# The Plasma Proteome Mediates the Association Between Workplace Noise Exposure and Psychiatric Disorder Risk

**DOI:** 10.1002/cns.71236

**Published:** 2026-11-07

**Authors:** Xuan Zhao, Yan Fu, Dan‐Dan Zhang, Wei Xu, Hui‐Fu Wang, Wei Zhang, Jin‐Tai Yu, Lan Tan

**Affiliations:** ^1^ Department of Neurology, Qingdao Municipal Hospital Qingdao University Qingdao China; ^2^ Department of Neurology, Qingdao Hospital University of Health and Rehabilitation Sciences (Qingdao Municipal Hospital) Qingdao China; ^3^ Department of Neurology and National Center for Neurological Disorders, Huashan Hospital, Shanghai Medical College Fudan University Shanghai China

**Keywords:** mediation analysis, psychiatric disorders, WNPRS, workplace noise exposure, workplace noise‐related proteomics

## Abstract

**Aims:**

Workplace noise exposure has been linked to adverse mental health outcomes, yet the underlying biological processes remain incompletely understood. We aimed to characterize workplace noise‐related plasma proteomic profiles, examine the mediating role of circulating proteins in the associations between workplace noise exposure and psychiatric disorders, and quantify individual susceptibility.

**Methods:**

Using UK Biobank data, we characterized workplace noise‐related proteomic profiles and examined the associations between workplace noise exposure and incident psychiatric disorders. Proteins associated with both exposure and disease risk were identified, followed by functional enrichment and mediation analyses. Workplace Noise‐related Protein Risk Scores (WNPRSs) were constructed from shared mediator proteins to assess individual susceptibility, joint risk patterns, and predictive performance.

**Results:**

We identified 229 workplace noise‐associated plasma proteins, with assay‐specific enrichment predominantly involving extracellular and membrane‐associated cellular components. Circulating proteins partially mediated the associations of workplace noise exposure with anxiety disorder and major depressive disorder (MDD), with mediation proportions ranging from 1.9% to 9.2% and 2.5% to 19.4%, respectively. WNPRS‐Anxiety and WNPRS‐MDD, both based on 44 shared mediator proteins, were associated with workplace noise exposure and the risks of the corresponding psychiatric disorders.

**Conclusions:**

This study identifies workplace noise‐related plasma proteomic signatures, suggests that circulating proteins partly mediate noise‐associated psychiatric risk, and highlights the potential utility of WNPRS for characterizing individual susceptibility.

## Graphical abstract

Workplace noise exposure was associated with adverse psychiatric outcomes and plasma proteomic alterations in the UK Biobank. Circulating proteins partially mediated associations with anxiety disorder and major depressive disorder (MDD), while workplace noise‐related protein risk scores provided proteomics‐based measures of individual susceptibility to psychiatric risk.

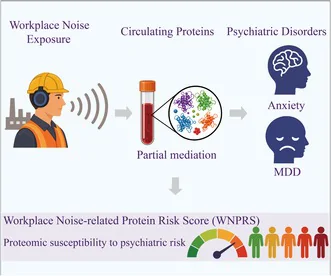

## 1 Introduction

In recent years, noise exposure has increasingly been recognized as a significant environmental factor affecting public health [1]. Beyond auditory effects, noise exposure has been linked to diverse non‐auditory outcomes, including cardiovascular disease, metabolic disorders, sleep disturbance, cognitive decline, and poor mental health [2, 3]. With the development of modern industrialization, workplace noise exposure has become an important environmental and public health concern. Meanwhile, mental disorders have emerged as a major global public health challenge [4], disrupting emotional, cognitive, sleep‐related, and behavioral functions [5, 6] and imposing substantial individual and societal burdens [7]. Although workplace noise exposure has been linked to increased risks of psychiatric disorders, the underlying molecular mechanisms remain insufficiently understood.

Previous research has identified mental stress, autonomic and endocrine activation, oxidative stress, inflammation, endothelial dysfunction, neuroinflammation, and immune‐cell dysregulation as shared pathogenic pathways in noise‐related psychiatric disorders [2, 8]. Recent proteomic studies have provided molecular evidence for the biological effects of noise exposure. For example, acute nocturnal train noise exposure induced substantial plasma protein changes mainly involving redox, pro‐thrombotic, and proinflammatory pathways in a controlled human study [9], while a mouse model of noise‐induced hearing loss showed marked cochlear proteomic alterations after high‐intensity noise exposure [10].

Proteins are key functional mediators of gene expression and provide molecular insights into disease processes [11]. Although psychiatric disorders mainly involve the central nervous system, brain tissue studies are limited by ethical and invasive constraints [12]. By contrast, blood‐based proteins are more accessible and may partly reflect brain‐related pathological changes [13]. With the availability of large‐scale plasma proteomic data, circulating proteins have emerged as valuable tools for disease risk prediction and mechanistic investigation, capturing molecular information from genetics, lifestyle, environmental exposures, and early pathophysiological changes [14]. Therefore, noise‐related proteomic analysis may help uncover molecular pathways linking workplace noise exposure to psychiatric disorders and provide biological evidence for individualized risk assessment [15].

With the release of UK Biobank (UKB) proteomic data, plasma proteins have been linked to multiple complex health outcomes. A recent UKB study suggested that plasma proteins may mediate the health effects of noise‐related environmental exposure patterns [16]. However, existing studies have not systematically linked workplace noise exposure‐related proteomic alterations to longitudinal psychiatric outcomes.

In this study, we addressed these gaps by investigating the role of circulating proteins in mediating the association between workplace noise exposure and psychiatric disorders. Using data from the UKB, we systematically examined the relationships among workplace noise exposure, plasma proteomic profiles, and incident psychiatric disorders. Based on the mediation analysis, we developed workplace noise‐related protein risk scores (WNPRS) to quantify individual proteomic susceptibility to workplace noise‐related psychiatric risk [17]. Together, this study aimed to characterize noise‐related proteomic alterations, evaluate their potential mediating roles, and develop proteomics‐informed scores to facilitate individual risk assessment and targeted prevention in occupational mental health. The study design is presented in Figure 1.

**FIGURE 1:**
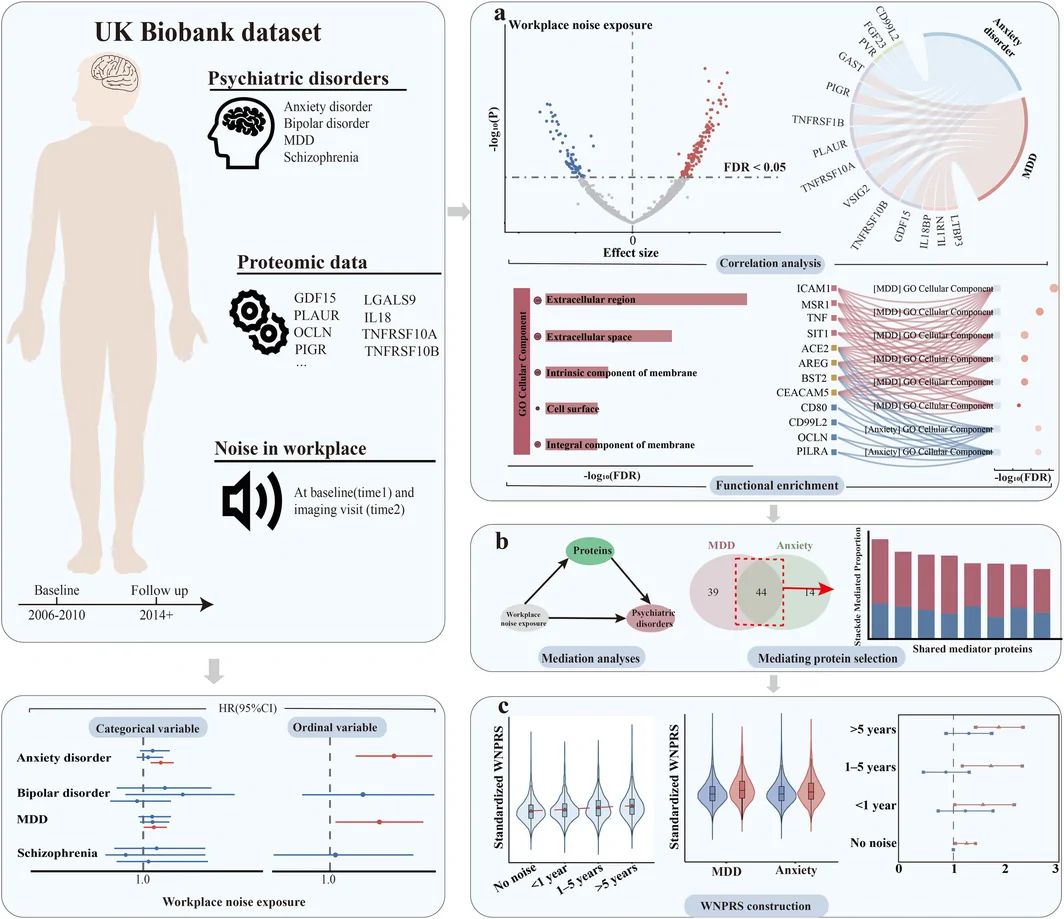
Guideline of the study. Top left, UK Biobank data used in this study included workplace noise exposure, plasma proteomic data, and psychiatric outcomes. Bottom left, associations between workplace noise exposure and incident psychiatric disorders assessed using Cox proportional hazards models. (a) Identification of workplace noise‐associated proteins, their overlap with psychiatric disorder‐associated proteins, and functional enrichment. Red and blue dots indicate significantly increased and decreased protein levels, respectively (FDR < 0.05). (b) Mediation analysis and mediator selection. The Venn diagram shows mediating proteins shared between anxiety disorder and MDD, and the stacked bar plot shows their mediation proportions. (c) Evaluation of WNPRS in relation to psychiatric disorder risk.

## 2 Materials and Methods

### 2.1 Participants

This study used data from the UKB, a large prospective cohort of more than 500,000 participants aged 40–69 years recruited from 22 assessment centers in England, Wales, and Scotland between 2006 and 2010. Data were collected through questionnaires, physical examinations, cognitive tests, biological samples, imaging, and genotyping. More details of the UKB cohort have been described previously [18, 19]. Information about the UK Biobank study design and assessment procedures is available at https://www.ukbiobank.ac.uk/.

In the present study, we first excluded participants with a diagnosis of anxiety disorder, major depressive disorder (MDD), bipolar disorder, or schizophrenia at baseline and then excluded those without available workplace noise exposure data or baseline plasma proteomic data. Separate analytic cohorts were subsequently constructed for each of the four psychiatric outcomes. For each cohort, the comparison group consisted of participants who did not develop any of the four psychiatric disorders during follow‐up. The primary analytic cohorts included 15,876 participants for anxiety disorder, 15,680 for MDD, 14,962 for bipolar disorder, and 14,956 for schizophrenia. The participant selection process is shown in Figure S1.

### 2.2 Assessment of Noise Exposure in the Workplace

Workplace noise exposure was assessed using touchscreen‐based questionnaire data (Field 4825) collected at the baseline assessment (2006–2010). Participants were classified according to the reported duration of working in a noisy place where they had to shout to be heard, including no exposure, < 1 year, 1–5 years, and > 5 years. This field provides information on the history and duration of workplace noise exposure but does not specify the exact timing or recency of the exposure (Methods S1).

### 2.3 Psychiatric Disorders

The studied psychiatric disorders, including anxiety disorder, bipolar disorder, MDD, and schizophrenia, were defined using International Classification of Diseases, 10th Revision (ICD‐10) diagnostic codes, with detailed definitions provided in Methods S2 and the corresponding codes listed in Table S1. Follow‐up began at the baseline assessment and ended at the first diagnosis of the corresponding disorder, death, loss to follow‐up, or the latest available linked health record, whichever occurred first.

### 2.4 Assessment of Covariates

Relevant covariates, including sociodemographic factors and physical measurements, were collected through questionnaires and registers at baseline. These included age, sex, ethnicity, body mass index (BMI), Townsend deprivation index (TDI), smoking status, hearing aid use, and the weighted average 24‐h traffic noise level (Lden). For Lden, a penalty of 5 dB and 10 dB was added to the evening and nighttime hours, respectively [20]. Additional variables included in the sensitivity analyses were lipid‐lowering and psychotropic medication use, hypertension, diabetes mellitus, cardiovascular disease (CVD), and chronic kidney disease (CKD). Detailed definitions are provided in Methods S3, with corresponding codes listed in Tables S2–S4.

### 2.5 Blood Proteomics

Plasma proteomic data (Category 1839) were obtained from the UK Biobank Pharma Proteomics Project (UKB‐PPP), with baseline proteomic measurements available for 53,026 participants. Plasma proteins were measured by Olink Analytical Services in Sweden using the Olink Explore 3072 platform. A total of 2923 unique proteins were quantified and released as normalized protein expression (NPX) values, which had undergone normalization within and across batches. Protein names were reported according to the annotations provided by the Olink Explore 3072 platform. Further details of sample selection and laboratory procedures have been reported previously [12, 21]. The proteomic data released by UK Biobank had undergone quality‐control procedures implemented by the UKB‐PPP, including the identification and removal of potential outlier samples based on principal component analysis (PCA) and sample‐level median and interquartile range (IQR) analyses of NPX values [22]. No additional outlier exclusion was performed in the present study.

In this study, proteins with more than 30% missing values were excluded [23]. Accordingly, three proteins (GLIPR1, NPM1, and PCOLCE) were excluded, leaving 2920 proteins for subsequent analyses. Remaining missing protein measurements were not imputed, and regression models were fitted using participants with complete data for the corresponding protein and covariates. NPX values were standardized to Z‐scores before regression analyses. Further details are provided in the Methods S4.

### 2.6 Statistical Analysis

Baseline characteristics were compared according to workplace noise exposure duration. Continuous variables were presented as mean ± standard deviation (SD) and were compared using analysis of variance (ANOVA), whereas categorical variables were presented as counts and percentages and were compared using *χ* ^2^ tests.

Multivariable linear regression models were used to identify plasma proteins associated with workplace noise exposure, adjusting for age, sex, ethnicity, smoking status, body mass index (BMI), Townsend deprivation index (TDI), Lden, and hearing aid use. Cox proportional hazards models with the same covariate adjustment were fitted using the survival package (version 3.7–0) [24] to evaluate the associations of workplace noise exposure with incident anxiety disorder, MDD, bipolar disorder, and schizophrenia, as well as the associations of plasma proteins with anxiety disorder and MDD. Cox model results were reported as hazard ratios (HRs) with 95% confidence intervals (CIs). To assess potential effect modification by sex, sex × workplace noise exposure group interaction terms were included in the fully adjusted Cox models without splitting the analytic sample by sex. The overall significance of the interaction was assessed using likelihood ratio tests comparing models with and without the interaction terms. Given the potential relationship between workplace noise exposure and hearing aid use, their association was additionally examined using *χ* ^2^ tests and logistic regression. Model assumptions and potential multicollinearity were evaluated, with detailed procedures described in Methods S5 and the corresponding results presented in Tables S11 and S18.

Additional robustness analyses were conducted to assess the consistency and stability of the Cox regression findings. For each psychiatric outcome, the analytic cohort was randomly divided into a training set (70%) and a testing set (30%) using stratified random sampling based on outcome status with the caret package (version 7.0–1) [25]. Cox proportional hazards analyses were repeated separately in both subsets using the same covariate adjustments as in the primary analyses. Previous studies have suggested that lipid‐lowering medications, particularly statins, may alter circulating protein profiles [26]. Given the psychiatric outcomes of this study, psychotropic medication use was also considered. Sensitivity analyses were therefore repeated according to lipid‐lowering and psychotropic medication use, and the corresponding proteomic models were additionally adjusted for hypertension, diabetes mellitus, CVD, and CKD [27]. Details are provided in Methods S6.

Mediation analysis was performed to investigate the potential mediating role of plasma proteins in the associations of workplace noise exposure with MDD and anxiety disorder. Candidate mediators were proteins significantly associated with both workplace noise exposure and the corresponding outcome in the preceding analyses. Mediation models were adjusted for age, sex, ethnicity, smoking status, BMI, TDI, Lden, and hearing aid use. Mediation effects were estimated using the mediation package (version 4.5.1) [28] (Methods S7). Functional enrichment analyses were performed using STRING for workplace noise‐associated proteins and mediator proteins identified for the relevant psychiatric outcomes [29]. To account for the targeted coverage of the Olink platform, the 2920 proteins available for association testing were used as the statistical background in the primary enrichment analyses. The enrichment analyses were additionally repeated using the default *Homo sapiens* genome‐wide background as sensitivity analyses (Methods S8). To prioritize mediation signals supported across outcomes, workplace noise‐related protein risk scores (WNPRSs) were further constructed using mediator proteins shared across the psychiatric outcomes included in the mediation analysis, with the corresponding mediation‐effect estimates used as weights for each outcome. The resulting scores were evaluated to assess individual susceptibility, joint risk patterns, and associations with the corresponding psychiatric disorder risk (Methods S9). Predictive performance was assessed using the area under the receiver operating characteristic curve (AUROC), calculated with the pROC package (version 1.19.0.1) [30].

All statistical tests were two‐sided. For the associations between workplace noise exposure and each psychiatric outcome, a *p* value < 0.05 was considered statistically significant. For proteomic analyses, *p* values were adjusted for multiple testing using the Benjamini–Hochberg (BH) procedure, with a false discovery rate (FDR) < 0.05 considered statistically significant. All statistical analyses were conducted using R software (version 4.4.2), and data visualization was performed using ggplot2 (version 4.0.3) [31].

## 3 Results

### 3.1 Baseline Characteristics of the Participants

In this study, the four analytic cohorts included 15,876 participants for anxiety disorder, 15,680 for MDD, 14,962 for bipolar disorder, and 14,956 for schizophrenia. Across the four cohorts, mean age ranged from 56.74 to 56.80 years, and women accounted for 51.6%–52.5% of participants. Baseline characteristics are presented in Table 1, with detailed characteristics according to workplace noise exposure duration provided in Tables S5–S8. Hearing aid use was positively associated with longer workplace noise exposure across all analytic cohorts (all *p* < 0.001; Table S9).

**TABLE 1:** Baseline characteristics of participants in the four analytic cohorts.

Characteristics	Anxiety disorder analytic cohort	MDD analytic cohort	Bipolar disorder analytic cohort	Schizophrenia analytic cohort
*n*	15,876	15,680	14,962	14,956
Age (mean ± SD)	56.78 (8.31)	56.80 (8.31)	56.74 (8.31)	56.74 (8.31)
Sex (%)				
Female	8337 (52.5)	8160 (52.0)	7734 (51.7)	7722 (51.6)
Male	7539 (47.5)	7520 (48.0)	7228 (48.3)	7234 (48.4)
Ethnicity (%)				
Non‐White	1647 (10.4)	1634 (10.5)	1569 (10.5)	1566 (10.5)
White	14,164 (89.6)	13,981 (89.5)	13,331 (89.5)	13,328 (89.5)
Smoking status (%)				
Never	8824 (55.7)	8723 (55.8)	8367 (56.1)	8366 (56.1)
Previous	5560 (35.1)	5479 (35.0)	5223 (35.0)	5222 (35.0)
Current	1452 (9.2)	1438 (9.2)	1335 (8.9)	1331 (8.9)
BMI (mean ± SD)	27.27 (4.70)	27.30 (4.71)	27.23 (4.65)	27.23 (4.65)
TDI (mean ± SD)	−1.08 (2.99)	−1.08 (3.00)	−1.11 (2.98)	−1.11 (2.98)
Lden (mean ± SD)	55.98 (4.24)	55.95 (4.20)	55.96 (4.21)	55.96 (4.21)
Hearing aid use (%)				
No	15,391 (97.0)	15,198 (97.0)	14,517 (97.1)	14,510 (97.1)
Yes	475 (3.0)	471 (3.0)	435 (2.9)	436 (2.9)

*Note:* The four analytic cohorts were not mutually exclusive. Continuous variables are presented as mean ± standard deviation (SD), and categorical variables as *n* (%).

Abbreviations: BMI, body mass index; Lden, the weighted average 24‐h traffic noise level; MDD, major depressive disorder; TDI, Townsend deprivation index.

The analytic cohorts were not mutually exclusive. Among participants who developed anxiety disorder, MDD, bipolar disorder, or schizophrenia during follow‐up, 206/944 (21.82%), 205/748 (27.41%), 18/30 (60.00%), and 5/24 (20.83%), respectively, also developed at least one of the other three psychiatric disorders (Table S10).

### 3.2 Associations Between Workplace Noise Exposure and Plasma Proteins

We first examined the associations between workplace noise exposure and plasma proteins among 18,359 participants with both workplace noise exposure and plasma proteomic data.

Workplace noise exposure was first modeled as an ordinal variable, identifying 229 significant workplace noise‐associated proteins (Figure 2a; Table S12). To assess the robustness of these findings, we additionally conducted categorical analyses using no workplace noise exposure as the reference group and comparing participants exposed for < 1 year, 1–5 years, and > 5 years (Figure 2a; Tables S13–S15). Proteins identified from these categorical comparisons were fully contained within the significant protein set derived from the ordinal exposure model, indicating consistent proteomic associations across different exposure modeling strategies.

**FIGURE 2:**
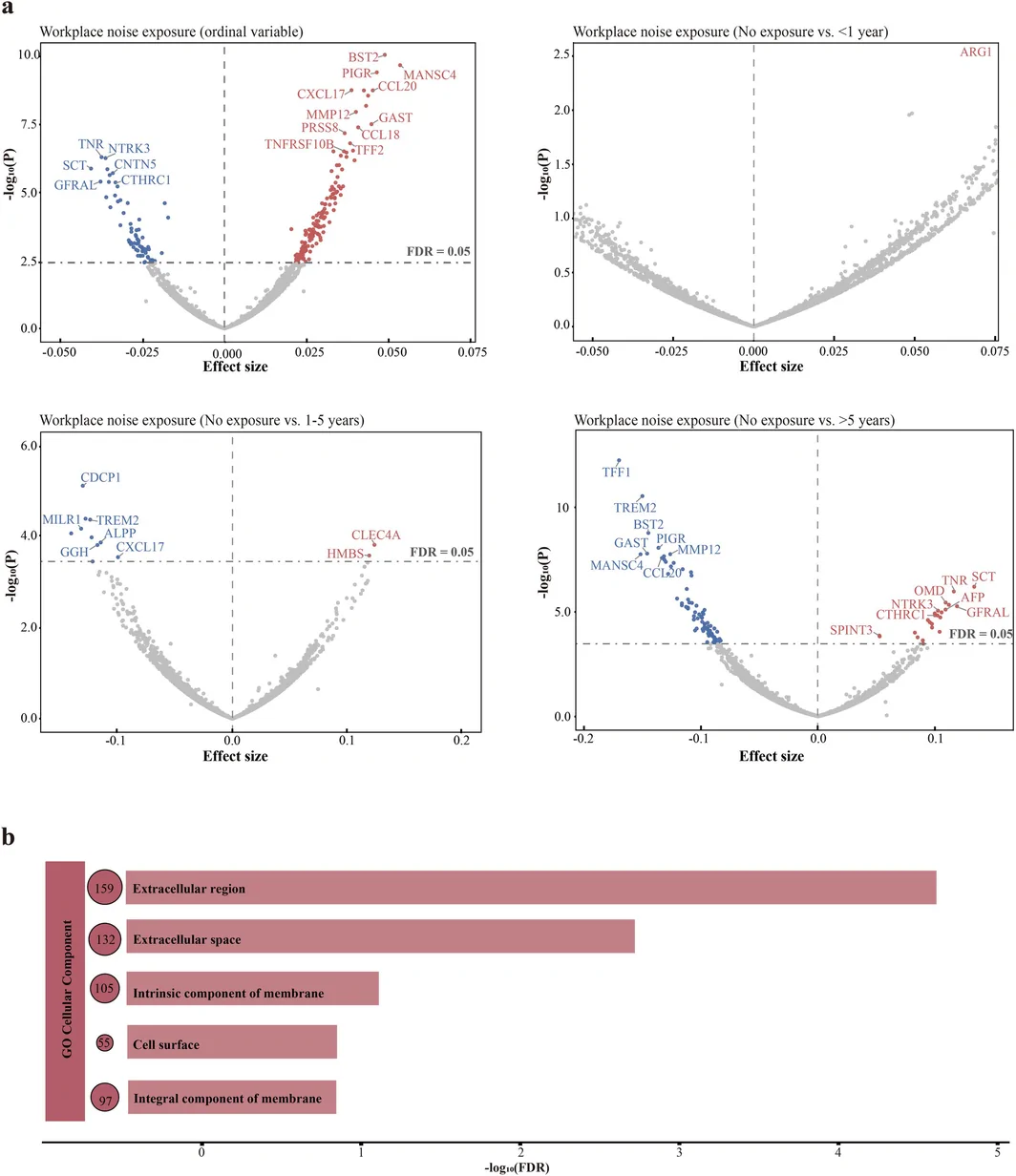
Associations between plasma proteins and workplace noise exposure. (a) Volcano plots showing associations between workplace noise exposure and plasma proteins using ordinal and categorical exposure models, with no exposure as the categorical reference. The y‐axis shows –log_10_(*p*); red and blue dots indicate significantly increased and decreased protein levels, respectively (FDR < 0.05). (b) Functional enrichment of workplace noise‐associated proteins using the 2920‐protein assay‐specific background. Significant enrichment was confined to GO Cellular Component terms. Bar length indicates −log_10_(FDR), and circles indicate the observed protein count.

We then performed functional enrichment analyses to characterize the biological characteristics of the workplace noise‐associated proteins. Using the 2920 proteins available for proteomic association testing as the assay‐specific statistical background, significant enrichment was confined to Gene Ontology (GO) Cellular Component terms, including extracellular region, extracellular space, membrane‐associated components, and cell‐surface terms (Figure 2b; Table S16). No significant GO Biological Process or Molecular Function terms, Kyoto Encyclopedia of Genes and Genomes (KEGG) pathways, or Reactome pathways were identified using this background. When the enrichment analyses were repeated using the default *Homo sapiens* genome‐wide background as sensitivity analyses, broader enrichment patterns emerged, including terms related to immune‐inflammatory and stress‐response processes, MAPK signaling, and cell adhesion/migration (Table S17).

### 3.3 Associations Between Workplace Noise Exposure and Psychiatric Disorders

Using Cox proportional hazards models, we evaluated the associations between workplace noise exposure and the risk of four psychiatric disorders (Figure 3a). Compared with participants without workplace noise exposure, those exposed for > 5 years had significantly higher risks of anxiety disorder (HR [95% CI]: 1.43 [1.17–1.75], *p* < 0.001) and MDD (1.26 [1.01–1.58], *p* = 0.041). Workplace noise exposure was not significantly associated with bipolar disorder or schizophrenia. To assess trends in psychiatric disorder risk with increasing workplace noise exposure duration, workplace noise exposure was modeled as an ordinal variable (Figure 3b). Significant positive trends were observed for anxiety disorder (1.12 [1.05–1.19], *p* < 0.001) and MDD (1.09 [1.01–1.17], *p* = 0.021), whereas no significant trends were observed for bipolar disorder or schizophrenia.

**FIGURE 3:**
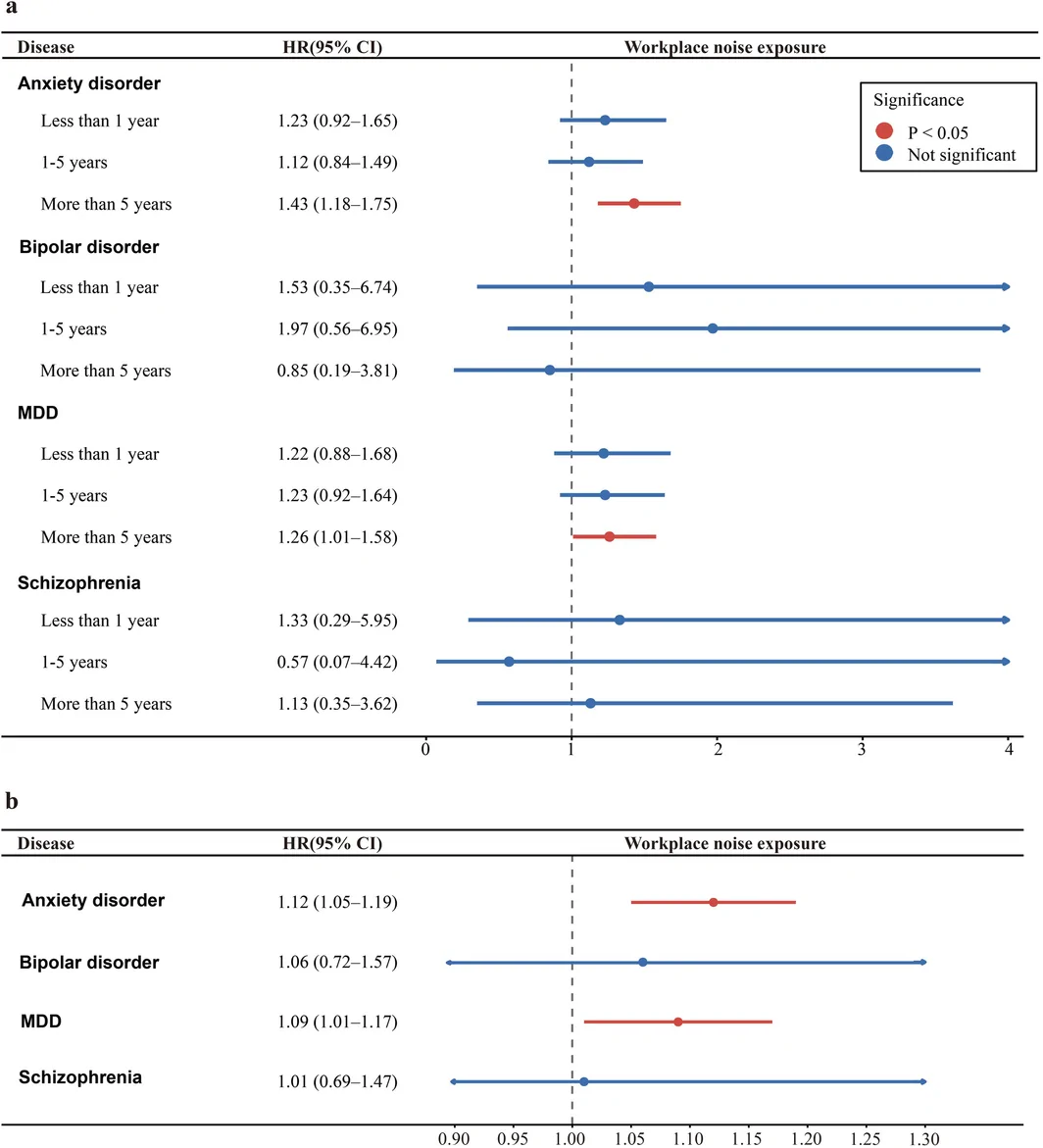
Associations between workplace noise exposure and psychiatric disorders. (a) Associations of workplace noise exposure with the risk of psychiatric disorders (categorical variable). (b) Associations of workplace noise exposure with the risk of psychiatric disorders (ordinal variable). Hazard ratios (HRs) and 95% confidence intervals (CIs) were estimated using Cox proportional hazards models. For the categorical analysis, no exposure was used as the reference group.

We further examined potential effect modification by sex. A significant sex × workplace noise exposure interaction was observed for MDD (P for interaction = 0.001), but not for the other three psychiatric outcomes (all P for interaction > 0.05). Sex‐specific estimates from the interaction model showed that workplace noise exposure for 1–5 years (1.47 [1.01–2.14], *p* = 0.043) and > 5 years (1.65 [1.26–2.16], *p* < 0.001) were associated with a higher risk of MDD in men, whereas no significant associations were observed in women (Table S19).

### 3.4 The Mediating Role of Proteins in the Association Between Workplace Noise Exposure and Psychiatric Disorders

To investigate the potential mediating role of plasma proteins in the associations of workplace noise exposure with anxiety disorder and MDD (Figure 4a), we first used Cox models to identify proteins associated with each disorder (FDR < 0.05). A total of 239 and 343 proteins were significantly associated with anxiety disorder and MDD, respectively (Figure 4b; Tables S20 and S21). By intersecting these disease‐associated proteins with the workplace noise exposure‐related proteins identified in our analysis, we identified 60 and 93 proteins that were simultaneously associated with workplace noise exposure and anxiety disorder or MDD, respectively.

**FIGURE 4:**
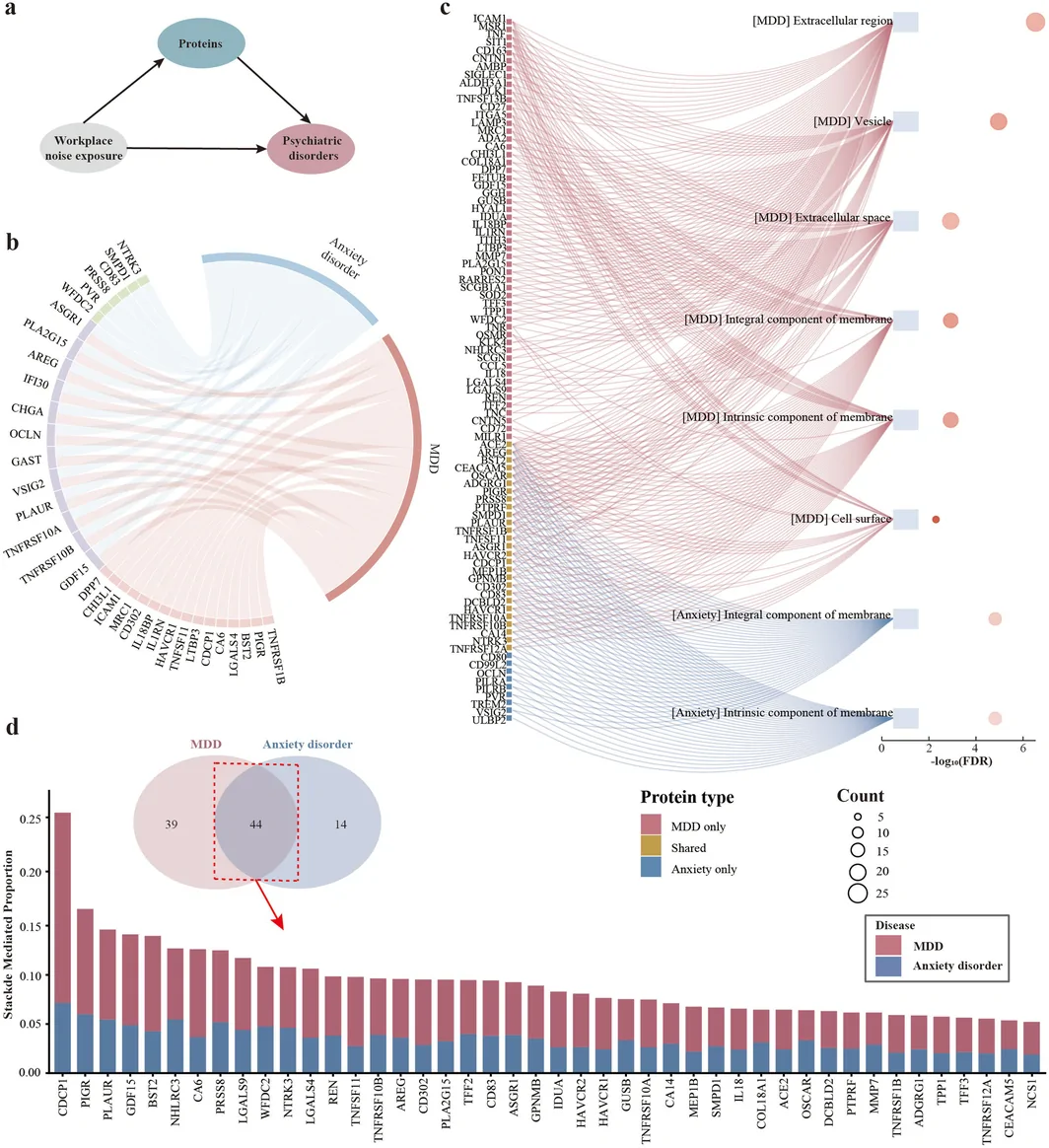
Mediation analysis linking workplace noise exposure, plasma proteins, and psychiatric disorders. (a) Schematic diagram illustrating the mediation analysis framework. (b) Plasma proteins associated with both workplace noise exposure and anxiety disorder or MDD. For visualization purposes, the top 30% of candidate proteins for each outcome were displayed based on FDR ranking. Proteins associated with anxiety disorder and MDD are shown in green and red, respectively. (c) Functional enrichment of mediator proteins for anxiety disorder and MDD using the 2920‐protein assay‐specific background. Curved links connect representative mediator proteins to significant GO Cellular Component terms. (d) Shared mediator proteins and mediation proportions. The Venn diagram shows the 44 mediator proteins shared between anxiety disorder and MDD; the dashed box highlights these proteins, which were used to construct WNPRS‐Anxiety and WNPRS‐MDD. The stacked bar plot shows their mediation proportions for the two disorders.

We then performed mediation analyses to assess whether plasma proteins mediated the associations of workplace noise exposure with anxiety disorder and MDD. This analysis identified 58 mediating proteins for anxiety disorder, including GAST, GDF15, PLAUR, TNFRSF10B, PIGR, and OCLN, and 83 for MDD, including GDF15, TNF, PLAUR, TNFRSF10B, PIGR, and LGALS9 (Tables S22 and S23).

To further characterize the functional features of the mediator proteins, we performed enrichment analyses using the 2920 proteins available for association testing as the assay‐specific statistical background (Figure 4c; Tables S24 and S25). For anxiety disorder, significant enrichment was confined to GO Cellular Component terms related to integral and intrinsic components of the membrane. For MDD, significant enrichment involved extracellular region, extracellular space, vesicular components, membrane‐associated components, and cell‐surface terms. No significant GO Biological Process or Molecular Function terms, KEGG pathways, or Reactome pathways were identified for either set of mediator proteins using the assay‐specific background. In sensitivity analyses using the default *Homo sapiens* genome‐wide background, broader enrichment patterns were observed (Tables S26 and S27). Anxiety disorder mediators showed enrichment in processes related to stress responses, protein phosphorylation, and immune‐inflammatory processes, whereas MDD mediators showed enrichment in immune‐inflammatory and stress‐response processes and MAPK/NF‐κB signaling.

### 3.5 Robustness and Sensitivity Analyses

In the robustness analyses, the primary Cox findings were generally consistent in the training and testing subsets. For anxiety disorder, workplace noise exposure for > 5 years remained significantly associated with increased risk in both the training (HR = 1.39, *p* = 0.007) and testing sets (HR = 1.51, *p* = 0.027), consistent with the primary analysis. For MDD, effect estimates remained directionally consistent across the primary, training, and testing analyses (HR = 1.26, 1.29, and 1.19, respectively), although statistical significance was observed only in the primary analysis. Estimates for bipolar disorder and schizophrenia were less precise across the training and testing sets. Among the proteins identified in the primary analyses, 27/239 (11.3%) and 112/239 (46.9%) for anxiety disorder, and 231/343 (67.3%) and 4/343 (1.2%) for MDD remained significant at FDR < 0.05 in the training and testing sets, respectively. Corresponding effect estimates remained strongly correlated with those from the primary analyses (*r* = 0.836–0.971), with directional concordance ranging from 96.2% to 100% (Tables S28–S30).

In further sensitivity analyses, the number of proteins remaining significant at FDR < 0.05 varied across medication‐use strata, with fewer proteins remaining significant in some stratified analyses, particularly among psychotropic medication users. Effect estimates were generally consistent with the primary analyses, with Pearson correlations ranging from 0.789 to 0.984 for lipid‐lowering medication strata and from 0.516 to 0.996 for psychotropic medication strata; directional concordance ranged from 89.1% to 100%. After additional adjustment for hypertension, diabetes mellitus, CVD, and CKD, 222/229 (96.9%), 130/239 (54.4%), and 237/343 (69.1%) of the workplace noise–protein, protein–anxiety disorder, and protein–MDD associations, respectively, remained significant at FDR < 0.05. The corresponding effect estimates were highly correlated with those from the primary analyses (*r* ≥ 0.992), with 100% directional concordance (Tables S31–S34).

### 3.6 WNPRS Represents Individual Susceptibility to Workplace Noise‐Related Psychiatric Disorder Risk

The mediation analysis was designed to quantify the role of individual plasma proteins in mediating the associations between workplace noise exposure and psychiatric disorders. Thus, to quantify individual susceptibility to workplace noise exposure, we developed WNPRS‐MDD and WNPRS‐Anxiety based on proteins identified as mediators in the anxiety disorder and MDD analyses. A total of 44 proteins that mediated the associations of workplace noise exposure with both anxiety disorder and MDD were selected to construct the two scores (Figure 4d). The coefficients used to construct each score were derived from the mediation‐effect estimates of each protein.

The WNPRS‐MDD and WNPRS‐Anxiety values showed right‐skewed distributions (Figure S2) and were positively correlated with workplace noise exposure (Figure 5a). We then compared WNPRS distributions between healthy individuals and those who developed the corresponding psychiatric disorder (Figure 5b). Consistently, WNPRS values were significantly higher in individuals with psychiatric disorders than in those who remained healthy. In addition, we examined the associations between WNPRS and observed event rates (Figure 5c). Higher WNPRS percentiles were positively correlated with higher observed event rates for both anxiety disorder and MDD (both *p* < 0.001).

**FIGURE 5:**
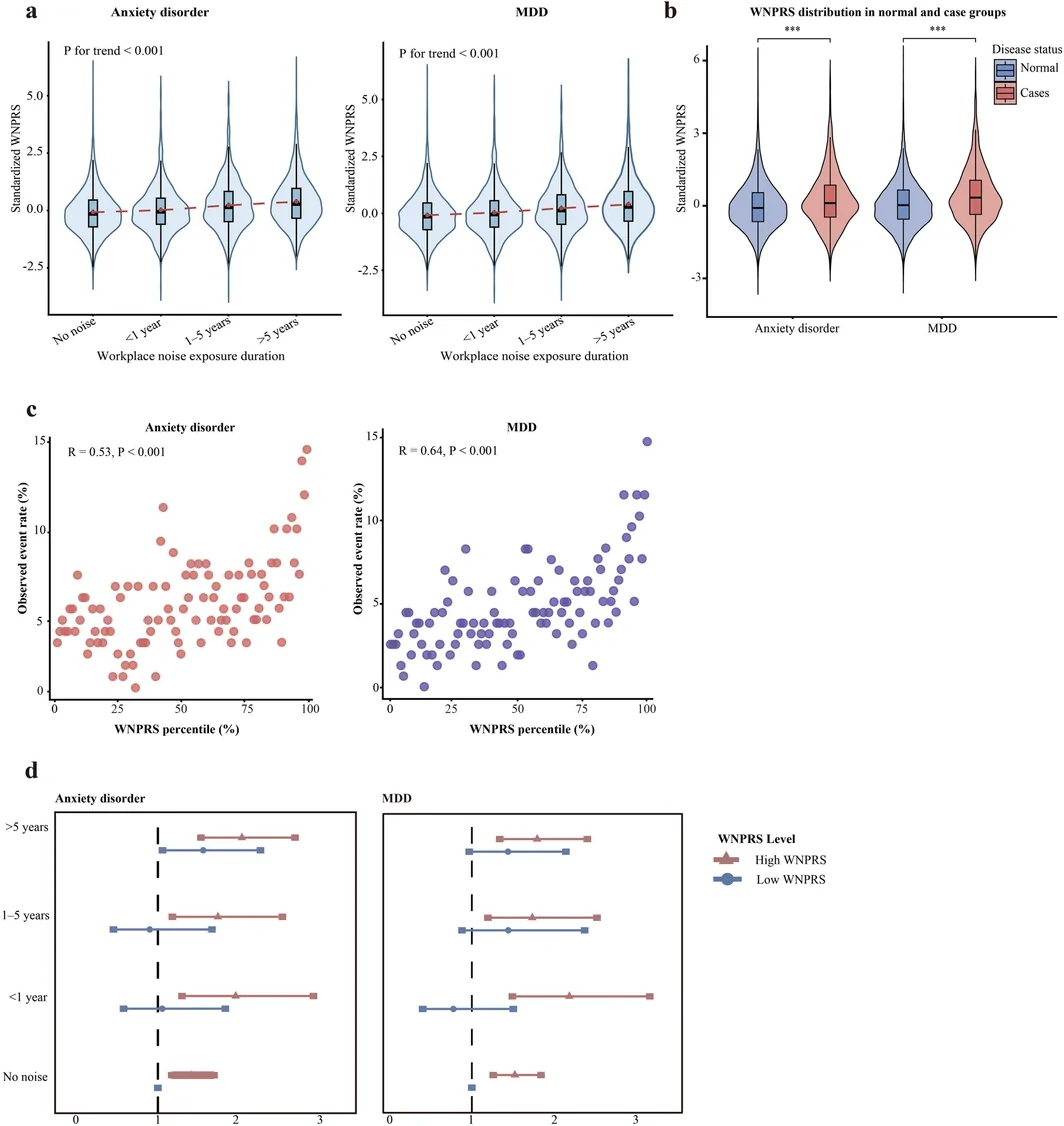
Evaluation of WNPRS in relation to psychiatric disorder risk. (a) Associations of WNPRS with workplace noise exposure duration. (b) Comparison of WNPRS between the normal and case groups. Differences were assessed using two‐sided Wilcoxon rank‐sum tests (***p < 0.001). (c) Associations of WNPRS with incidence rates of anxiety disorder and MDD. (d) Associations between workplace noise exposure and psychiatric disorder risk stratified by WNPRS level. Hazard ratios (HRs) and 95% confidence intervals (CIs) were estimated using Cox proportional hazards models; the dashed line indicates HR = 1.0.

Furthermore, to investigate the joint associations of workplace noise exposure and WNPRS with psychiatric disorder risk, we examined the association between WNPRS and disease incidence across different durations of workplace noise exposure (Figure 5d). Participants were stratified into four groups according to workplace noise exposure duration, and disease incidence rates were compared between individuals with high and low WNPRS within each exposure group. The results showed that, at the same level of workplace noise exposure, individuals with higher WNPRS consistently exhibited higher disease incidence rates.

### 3.7 WNPRS Improves Risk Prediction for Incident Psychiatric Disorders

Building on the observed positive associations of WNPRS‐MDD and WNPRS‐Anxiety with psychiatric disorder risk, we constructed prediction models incorporating WNPRS to assess the risk of incident psychiatric disorders. Basic models included age, sex, smoking status, ethnicity, BMI, TDI, Lden, hearing‐aid use, hypertension, and diabetes. Their predictive performance was compared with that of the corresponding models augmented with WNPRS. The results showed that adding WNPRS as an additional predictor modestly improved model performance. Compared with the basic models, the area under the receiver operating characteristic curve (AUROC) increased by 1.25 and 1.10 percentage points for MDD and anxiety disorder, respectively (*p* = 0.014 and *p* = 0.006, respectively). In both models, the coefficients for WNPRS were positive, indicating that higher WNPRS values were associated with higher predicted risks of MDD and anxiety disorder.

## 4 Discussion

In this study, using UK Biobank data, we investigated the potential mediating role of plasma proteins in the associations between workplace noise exposure and psychiatric disorders. Workplace noise exposure was associated with higher risks of anxiety disorder and MDD, with an increasing trend in risk observed with longer exposure duration, whereas no significant associations were observed for bipolar disorder or schizophrenia. However, given the limited number of bipolar disorder and schizophrenia cases, further validation in larger and more diverse populations is warranted. A total of 58 and 83 proteins were identified as potential mediators of the associations with anxiety disorder and MDD, respectively, with mediation proportions ranging from 1.9% to 9.2% and from 2.5% to 19.4%. We further developed WNPRS‐Anxiety and WNPRS‐MDD to quantify individual susceptibility to workplace noise‐related psychiatric risk and assess their joint associations with workplace noise exposure.

We further observed a significant sex × workplace noise exposure interaction for incident MDD, with increased risk observed in men but not in women. Previous studies have reported inconsistent sex‐specific associations between occupational noise exposure and depressive outcomes. Kwon et al. reported an association between severe occupational noise exposure and depression in men but not in women [32], whereas a study of young Finnish adults observed an association with depressive symptoms only in women [1]. These differences may partly reflect variation in study populations, exposure assessment, and outcome definitions. In addition, previous evidence suggests sex differences in stress‐related biological responses, including hypothalamic–pituitary–adrenal (HPA) axis function and glutathione redox homeostasis [33], while experimental evidence also supports sex differences in neural and behavioral responses to stress [34]. Together, these findings suggest that sex may modify susceptibility to the mental health effects of workplace noise exposure, although further confirmation is needed.

Previous studies have investigated the relationship between noise exposure and proteomic alterations. However, most have focused on transportation‐related noise, such as road traffic, railway, and aircraft noise. For example, a randomized, double‐blind crossover study showed that acute nocturnal road traffic noise altered plasma proteins related to interleukin signaling and chemotaxis, and these molecular signatures were linked to inter‐individual physiological responses to noise [35]. Workplace noise exposure, however, may represent a distinct type of noise exposure, as it is often characterized by greater intensity, longer duration, repeated exposure, and co‐occurring occupational stress [36, 37]. Here, we systematically assessed associations between workplace noise exposure and circulating proteins, providing further insight into potential biological mechanisms underlying workplace noise‐related psychiatric risk. Subsequently, we integrated workplace noise‐related proteomic alterations with proteome‐disease associations to investigate the potential mediating role of plasma proteins in the associations of workplace noise exposure with anxiety disorder and MDD.

Using the assay‐specific background, workplace noise‐associated proteins were predominantly enriched in extracellular and membrane‐associated cellular components. This localization pattern is broadly consistent with previous experimental proteomic studies of noise exposure. In a mouse model of noise‐induced hearing loss, differentially expressed cochlear proteins were localized to the extracellular region and extracellular matrix [10]. More recently, an integrated proteomic and transcriptomic study in rats similarly identified enrichment of cochlear proteins in membrane‐related cellular components, including membrane raft and endosome membrane [38]. Although differences in species, exposure paradigms, and tissue sources limit direct comparisons, these studies provide supportive biological context for the extracellular and membrane‐associated localization observed in our study. For anxiety disorder, mediator proteins were predominantly enriched in integral and intrinsic components of the membrane, whereas MDD mediator proteins showed a broader enrichment pattern encompassing extracellular, vesicular, and membrane‐associated components. Similar localization patterns have been reported in previous studies of depressive and anxiety disorders. In MDD, cerebrospinal fluid proteomic analysis identified enrichment of differentially regulated proteins in the extracellular region and intrinsic membrane components [39], while an Olink‐based plasma proteomic study of mild depression also identified plasma membrane and extracellular region among the main Cellular Component annotations [40]. For anxiety disorder, serum peptidomic profiling of generalized anxiety disorder similarly showed that differential peptide‐associated proteins were localized to secreted and plasma membrane compartments [41]. Although differences in biospecimen, analytical platform, and study population limit direct comparisons, these studies provide supportive context for the extracellular and membrane‐associated localization patterns observed in our study. However, GO Cellular Component enrichment primarily reflects protein localization and does not directly indicate the biological processes through which these proteins act. Sensitivity analyses using the genome‐wide background yielded broader functional annotations, including immune‐inflammatory and stress‐response processes. These broader patterns are generally consistent with previous evidence implicating inflammatory, oxidative‐stress, and stress‐response mechanisms in the biological effects of noise exposure [42], as well as immune‐inflammatory and stress‐related pathways in MDD and anxiety disorder [43, 44]. In contrast, the assay‐specific analysis produced a narrower enrichment profile dominated by extracellular and membrane‐associated cellular components. This difference suggests that the broader annotations were sensitive to the choice of statistical background and may partly reflect the targeted composition of the Olink panel. We therefore based our primary biological interpretation on the assay‐specific analysis and considered the genome‐wide results exploratory and hypothesis‐generating.

Beyond these enrichment findings, we further examined the 44 mediator proteins shared between anxiety disorder and MDD that were used to construct WNPRS‐Anxiety and WNPRS‐MDD. This overlap in mediator proteins may reflect shared biological processes between anxiety disorder and MDD. This observation is consistent with previous evidence supporting shared molecular and pathophysiological features between anxiety and depression [45]. We therefore focused on representative shared mediator proteins with well‐characterized biological functions or prior evidence of involvement in these disorders. GDF15 is a stress‐responsive cytokine and a recognized marker of mitochondrial dysfunction, with established relevance across multiple disease conditions [14]. In addition, GDF15 has been reported to function as a pro‐inflammatory stress signal involved in inflammatory responses [46]. Thus, our identification of GDF15 as a shared mediator suggests that inflammatory and stress‐related processes may be involved in the associations of workplace noise exposure with anxiety disorder and MDD. PLAUR, the urokinase plasminogen activator receptor, is a key component of the plasminogen activation system and participates in multiple biological processes, including inflammatory responses, vascular homeostasis, and immune regulation [27]. Given that inflammatory activation, endothelial dysfunction, and disrupted vascular homeostasis have been implicated in anxiety and depression [47, 48], the identification of PLAUR as a shared mediator is consistent with the potential involvement of inflammatory and vascular processes in these associations. In addition, several proteins, including ASGR1, CA14, MMP7, PIGR, TNFRSF10A, TNFRSF10B, OCLN, IL18, PRSS8, and LGALS9, have previously been linked to relevant biological pathways and psychiatric outcomes [45]. Beyond these known proteins, our study identified additional potential mediators, suggesting a broader range of proteomic alterations that may contribute to the associations between workplace noise exposure and psychiatric disorder risk. Collectively, these findings expand our understanding of the proteomic changes associated with these relationships and identify candidate proteins and biological processes for further mechanistic investigation.

WNPRS‐Anxiety and WNPRS‐MDD provide protein‐based indices for quantifying individual susceptibility to workplace noise‐related psychiatric risk. Adding WNPRS to the basic models resulted in modest improvements in AUROC for both anxiety disorder and MDD, suggesting that these scores capture additional proteomic information beyond traditional risk factors. Given the relatively modest improvements in predictive performance, the current value of WNPRS may lie primarily in characterizing individual susceptibility and joint risk patterns rather than substantially improving clinical prediction. The present WNPRSs were constructed using mediator proteins shared by anxiety disorder and MDD. Future studies could compare this shared‐mediator approach with WNPRS models based on all significant mediators identified separately for each disorder to evaluate whether their inclusion provides additional predictive value. External validation, together with calibration, reclassification, and decision‐curve analyses, is needed to determine their practical utility.

This study has several strengths. To our knowledge, it is among the first large‐scale cohort studies to systematically examine the relationships among workplace noise exposure, the plasma proteome, and multiple psychiatric disorders. By integrating proteomic profiling, mediation analysis, and risk‐score construction, our study provides new insights into potential biological pathways linking workplace noise exposure to psychiatric risk. By incorporating mediating proteins, the WNPRS provides a personalized tool for assessing individual susceptibility to noise‐related psychiatric risk. Furthermore, the development of WNPRS‐Anxiety and WNPRS‐MDD provides a protein‐based framework for assessing individual susceptibility to noise‐related psychiatric risk.

However, several limitations should be acknowledged. First, workplace noise exposure was self‐reported and lacked information on the exact timing or recency of exposure relative to baseline blood collection, which may have introduced recall bias or exposure misclassification and limited assessment of the temporal relationship between noise exposure and proteomic profiles. Second, plasma proteins were measured at baseline and may be influenced by recent internal and external factors. Although sensitivity analyses accounting for medication use and major somatic conditions yielded generally consistent effect estimates, the reduced number of significant proteins in some medication‐stratified analyses may partly reflect smaller subgroup sample sizes and reduced statistical power. Residual influences from other unmeasured or incompletely characterized factors cannot be entirely excluded. Third, despite the longitudinal design and the exclusion of participants with any of the four psychiatric disorders at baseline, residual confounding and the potential influence of psychiatric symptoms or undiagnosed conditions predating baseline cannot be fully ruled out. Fourth, the observed sex‐related heterogeneity in the association between workplace noise exposure and MDD requires further confirmation. Fifth, functional enrichment was sensitive to the choice of statistical background, and the broader annotations obtained using the genome‐wide background may partly reflect the targeted composition of the Olink panel. These findings should therefore be interpreted with consideration of the selected reference background. Finally, WNPRS‐Anxiety and WNPRS‐MDD were developed within the UK Biobank cohort, and their generalizability requires further evaluation.

## 5 Conclusion

In conclusion, this study characterized workplace noise‐related plasma proteomic profiles and provided insights into biological processes that may link workplace noise exposure to psychiatric risk. We found that workplace noise exposure was associated with increased risks of anxiety disorder and MDD, and that plasma proteins partly mediated these associations. These findings highlight the value of large‐scale proteomics for investigating the biological processes underlying workplace noise‐related psychiatric risk. WNPRS‐Anxiety and WNPRS‐MDD provide proteomics‐informed indices for characterizing individual susceptibility and may facilitate future mechanistic investigation and risk stratification. Overall, these findings underscore the importance of occupational noise control and further investigation of the biological consequences of workplace noise exposure.

## Author Contributions

Drs Yu and Tan had full access to all the data in the study and take responsibility for the integrity of the data and the accuracy of the data analysis. Concept and design: Yu, Tan. Acquisition, analysis, or interpretation of data: Zhao, Fu. Drafting of the manuscript: Zhao. Critical revision of the manuscript for important intellectual content: All authors. Statistical analysis: Zhao, Fu. Obtained funding: Yu.

## Funding

This study was supported by grants from Brain Science and Brain‐like Intelligence Technology ‐ National Science and Technology Major Project (2022ZD0211600), the National Natural Science Foundation of China (82530047, 82588301, 82271471, 82271475).

## Ethics Statement

The UKB study received approval from the National Health Service (NHS) North West Multicenter Research Ethics Committee (16/NW/0274).

## Consent

All participants provided written informed consent according to the Declaration of Helsinki. This study utilized the UKB resource, with application number 108930.

## Conflicts of Interest

The authors declare no conflicts of interest.

## Supporting information


**Table S1:** The codes used for psychiatric disorders.
**Table S2:** UK Biobank Showcase variables used as covariates in this study.
**Table S3:** List of lipid‐lowering medications and corresponding codes.
**Table S4:** List of psychotropic medications and corresponding codes.
**Table S5:** Baseline Characteristics of Participants in the Anxiety Disorder Analytic Cohort According to Workplace Noise Exposure Duration.
**Table S6:** Baseline Characteristics of Participants in the MDD Analytic Cohort According to Workplace Noise Exposure Duration.
**Table S7:** Baseline Characteristics of Participants in the Bipolar Disorder Analytic Cohort According to Workplace Noise Exposure Duration.
**Table S8:** Baseline Characteristics of Participants in the Schizophrenia Analytic Cohort According to Workplace Noise Exposure Duration.
**Table S9:** Association between hearing aid use and workplace noise exposure across analytic cohorts.
**Table S10:** Proportions of Comorbid Psychiatric Disorders Among Participants with Incident Psychiatric Disorders.
**Table S11:** Multicollinearity assessment across regression models.
**Table S12:** Results of the associations between workplace noise exposure and plasma proteins (ordinal variable, FDR < 0.05).
**Table S13:** Results of the associations between workplace noise exposure and plasma proteins (no noise vs. < 1 year, FDR < 0.05).
**Table S14:** Results of the associations between workplace noise exposure and plasma proteins (no noise vs. 1–5 years, FDR < 0.05).
**Table S15:** Results of the associations between workplace noise exposure and plasma proteins (no noise vs. > 5 years, FDR < 0.05).
**Table S16:** Functional enrichment analysis of plasma proteins associated with workplace noise exposure using the 2920 proteins available for proteomic association testing as the assay‐specific background.
**Table S17:** Functional enrichment analysis of plasma proteins associated with workplace noise exposure using the default *Homo sapiens* genome‐wide background as a sensitivity analysis.
**Table S18:** Schoenfeld residual tests for the proportional hazards assumption in Cox models of workplace noise exposure and psychiatric disorders.
**Table S19:** Sex‐specific associations between workplace noise exposure and risk of MDD derived from the interaction model.
**Table S20:** Results of the associations between plasma proteins and anxiety disorder (FDR < 0.05).
**Table S21:** Results of the associations between plasma proteins and MDD (FDR < 0.05).
**Table S22:** Significant plasma protein mediators of the association between workplace noise exposure and anxiety disorder.
**Table S23:** Significant plasma protein mediators of the association between workplace noise exposure and MDD.
**Table S24:** Functional enrichment analysis of mediator proteins for the association between workplace noise exposure and anxiety disorder using the 2920 proteins available for proteomic association testing as the assay‐specific background.
**Table S25:** Functional enrichment analysis of mediator proteins for the association between workplace noise exposure and MDD using the 2920 proteins available for proteomic association testing as the assay‐specific background.
**Table S26:** Functional enrichment analysis of mediator proteins for the association between workplace noise exposure and anxiety disorder using the default *Homo sapiens* genome‐wide background as a sensitivity analysis.
**Table S27:** Functional enrichment analysis of mediator proteins for the association between workplace noise exposure and MDD using the default *Homo sapiens* genome‐wide background as a sensitivity analysis.
**Table S28:** Consistency of protein effect estimates across primary, training, and testing analyses.
**Table S29:** Detailed protein associations with anxiety disorder in the primary, training, and testing analyses.
**Table S30:** Detailed protein associations with MDD in the primary, training, and testing analyses.
**Table S31:** Consistency of protein effect estimates across primary and sensitivity analyses.
**Table S32:** Detailed associations between workplace noise exposure and plasma proteins in the primary and sensitivity analyses.
**Table S33:** Detailed protein associations with anxiety disorder in the primary and sensitivity analyses.
**Table S34:** Detailed protein associations with MDD in the primary and sensitivity analyses.
**Methods S1:** Assessment of noise exposure in the workplace.
**Methods S2:** Psychiatric disorders.
**Methods S3:** Covariates.
**Methods S4:** Blood proteomics.
**Methods S5:** Model assumptions and multicollinearity assessment.
**Methods S6:** Robustness and sensitivity analyses.
**Methods S7:** Mediation analysis.
**Methods S8:** Functional enrichment analysis.
**Methods S9:** Development of WNPRS.
**Figure S1:** Flowchart of participant selection for the four psychiatric outcome analyses.
**Figure S2:** Distributions of WNPRS‐Anxiety and WNPRS‐MDD.

## Data Availability

The datasets are available from the UKB with an approved protocol. External investigators can request the data on application to the UK Biobank (www.ukbiobank.ac.uk/).
