# Supplementary material for: The Plasma Proteome Mediates the Association Between Workplace Noise Exposure and Psychiatric Disorder Risk
Source: CNS Neurosci Ther. 2026 Nov 7;32(11):e71236. doi: 10.1002/cns.71236 (PMC13743995; doi:10.1002/cns.71236)
Supplement: Supplementary file 1 [file CNS-32-e71236-s001.docx]

**Supplementary Information**

**Content**

Supplementary tables......................................................................................................................1

Supplementary Methods 1: Assessment of noise exposure in the workplace.................................4

Supplementary Methods 2: Psychiatric disorders...........................................................................4

Supplementary Methods 3: Covariates...........................................................................................5

Supplementary Methods 4: Blood proteomics................................................................................6

Supplementary Methods 5 Model assumptions and multicollinearity assessment.........................7

Supplementary Methods 6: Robustness and sensitivity analyses...................................................8

Supplementary Methods 7: Mediation analysis..............................................................................9

Supplementary Methods 8: Functional enrichment analysis.........................................................10

Supplementary Methods 9: Development of WNPRS....................................................................11

References.......................................................................................................................................13

Supplementary Table 1. The codes used for psychiatric disorders.................................................15

Supplementary Table 2. UK Biobank Showcase variables used as covariates in this study..........16

Supplementary Table 3. List of lipid-lowering medications and corresponding codes..................17

Supplementary Table 4. List of psychotropic medications and corresponding codes....................18

Supplementary Table 5. Baseline Characteristics of Participants in the Anxiety Disorder Analytic Cohort According to Workplace Noise Exposure Duration...........................................................26

Supplementary Table 6. Baseline Characteristics of Participants in the MDD Analytic Cohort According to Workplace Noise Exposure Duration.......................................................................27

Supplementary Table 7. Baseline Characteristics of Participants in the Bipolar Disorder Analytic Cohort According to Workplace Noise Exposure Duration...........................................................28

Supplementary Table 8. Baseline Characteristics of Participants in the Schizophrenia Analytic Cohort According to Workplace Noise Exposure Duration...........................................................29

Supplementary Table 9. Association between hearing aid use and workplace noise exposure across analytic cohorts..............................................................................................................................30

Supplementary Table 10. Proportions of Comorbid Psychiatric Disorders Among Participants with Incident Psychiatric Disorders.......................................................................................................30

Supplementary Table 11. Multicollinearity assessment across regression models........................31

Supplementary Table 12. Results of the associations between workplace noise exposure and plasma proteins. (ordinal variable, FDR < 0.05) ......................................................................32

Supplementary Table 13. Results of the associations between workplace noise exposure and plasma proteins（no noise vs < 1 year, FDR < 0.05）............................................................37

Supplementary Table 14. Results of the associations between workplace noise exposure and plasma proteins（no noise vs 1-5 years, FDR < 0.05）..........................................................37

Supplementary Table 15. Results of the associations between workplace noise exposure and plasma proteins（no noise vs > 5 years, FDR < 0.05）..........................................................38

Supplementary Table 16. Functional enrichment analysis of plasma proteins associated with workplace noise exposure using the 2,920 proteins available for proteomic association testing as the assay-specific background..................................................................................................41

Supplementary Table 17. Functional enrichment analysis of plasma proteins associated with workplace noise exposure using the default Homo sapiens genome-wide background as a sensitivity analysis....................................................................................................................42

Supplementary Table 18. Schoenfeld residual tests for the proportional hazards assumption in Cox models of workplace noise exposure and psychiatric disorders..............................................60

Supplementary Table 19. Sex-specific associations between workplace noise exposure and risk of MDD derived from the interaction model................................................................................61

Supplementary Table 20. Results of the associations between plasma proteins and anxiety disorder (FDR < 0.05) ............................................................................................................................62

Supplementary Table 21. Results of the associations between plasma proteins and MDD (FDR < 0.05) ..........................................................................................................................................68

Supplementary Table 22. Significant plasma protein mediators of the association between workplace noise exposure and anxiety disorder.........................................................................77

Supplementary Table 23. Significant plasma protein mediators of the association between workplace noise exposure and MDD ........................................................................................79

Supplementary Table 24. Functional enrichment analysis of mediator proteins for the association between workplace noise exposure and anxiety disorder using the 2,920 proteins available for proteomic association testing as the assay-specific background................................................81

Supplementary Table 25. Functional enrichment analysis of mediator proteins for the association between workplace noise exposure and MDD using the 2,920 proteins available for proteomic association testing as the assay-specific background.................................................................81

Supplementary Table 26. Functional enrichment analysis of mediator proteins for the association between workplace noise exposure and anxiety disorder using the default *Homo sapiens* genome-wide background as a sensitivity analysis.................................................................................87

Supplementary Table 27. Functional enrichment analysis of mediator proteins for the association between workplace noise exposure and MDD using the default *Homo sapiens* genome-wide background as a sensitivity analysis........................................................................................88

Supplementary Table 28. Consistency of protein effect estimates across primary, training, and testing analyses.........................................................................................................................99

Supplementary Table 29. Detailed protein associations with anxiety disorder in the primary, training, and testing analyses...................................................................................................100

Supplementary Table 30. Detailed protein associations with MDD in the primary, training, and testing analyses........................................................................................................................110

Supplementary Table 31. Consistency of protein effect estimates across primary and sensitivity analyses...................................................................................................................................124

Supplementary Table 32. Detailed associations between workplace noise exposure and plasma proteins in the primary and sensitivity analyses.....................................................................125

Supplementary Table 33. Detailed protein associations with anxiety disorder in the primary and sensitivity analyses.................................................................................................................134

Supplementary Table 34. Detailed protein associations with MDD in the primary and sensitivity analyses....................................................................................................................................144

Supplementary Fig. S1 Flowchart of participant selection for the four psychiatric outcome analyses....................................................................................................................................158

Supplementary Fig. S2 Distributions of WNPRS-Anxiety and WNPRS-MDD. ....................159

**Supplementary Methods 1: Assessment of noise exposure in the workplace**

Workplace noise exposure was assessed using UK Biobank (UKB) touchscreen-based questionnaire data (Field 4825) collected at the baseline assessment (2006–2010). Participants were asked, “Have you ever worked in a noisy place where you had to shout to be heard?” Responses were recorded for all participants except those who reported being completely deaf. Response options included “yes, for >5 years”, “yes, for around 1 to 5 years”, “yes, for <1 year”, “no”, “do not know”, and “prefer not to answer”. In the present analysis, workplace noise exposure was categorized as no exposure, <1 year, 1–5 years, and >5 years. Participants who answered “do not know” or “prefer not to answer” were excluded. Field 4825 provides information on the history and duration of workplace noise exposure but does not specify the exact timing or recency of the exposure.

**Supplementary Methods 2: Psychiatric disorders**

The studied psychiatric disorders included anxiety disorder, bipolar disorder, major depressive disorder (MDD), and schizophrenia. These disorders were defined using the corresponding 3-character International Classification of Diseases, 10th Revision (ICD-10) codes, with specific codes and definitions provided in **Supplementary Table 1**. The baseline date was defined as the date of initial enrollment at the assessment center (Field 53). The event date was defined as the earliest recorded diagnosis date. Participants were followed from baseline until the first occurrence of the outcome of interest, death (Field 40000), loss to follow-up, or the latest available date from linked hospital inpatient records (Fields 41280–41281) or primary care records (Field 42040), whichever occurred first.

**Supplementary Methods 3: Covariates**

Covariates assessed at baseline included age, sex, ethnicity, body mass index (BMI, treated as a continuous variable), Townsend deprivation index (TDI), smoking status (never, previous, or current), hearing aid use, and the weighted average 24-hour traffic noise level (Lden). For Lden, penalties of 5 dB and 10 dB were added to the evening and nighttime periods, respectively (1). Participants who selected “prefer not to answer” or “do not know” for the relevant variables were excluded. Medication use was ascertained from self-reported medications recorded at baseline. Information on the number of treatments/medications taken was additionally used to distinguish participants reporting no medication use from those with missing medication information when no specific medication code was recorded. Lipid-lowering medication use, including statins, fibrates, and cholesterol absorption inhibitors (2), was identified using the medication codes listed in Supplementary Table 3***.*** Psychotropic medication use, including antidepressants, antipsychotics, mood stabilizers, sedative-hypnotics, and other psychotropic medications, was identified using a predefined list of 154 medications (3)**.** The distribution across medication classes and the complete list of medication names and corresponding UKB codes are provided in **Supplementary Tables 2-4**. Comorbidities considered in sensitivity analyses included hypertension, diabetes mellitus, cardiovascular disease (CVD), and chronic kidney disease (CKD). Hypertension was identified based on self-reported history of hypertension, systolic blood pressure ≥140 mmHg, diastolic blood pressure ≥90 mmHg, or use of antihypertensive medication. Diabetes mellitus was identified based on International Classification of Diseases, 10th Revision (ICD-10) codes E10–E11, self-reported history of diabetes, or use of glucose-lowering medication. CVD was identified by self-reported heart attack or angina history or International Classification of Diseases, 10th Revision (ICD-10) codes I21–I25. CKD was identified based on ICD-10 code N18 (4).

**Supplementary Methods 4: Blood proteomics**

Plasma proteomic data were obtained from the UK Biobank Pharma Proteomics Project (UKB-PPP; Category 1839). Plasma proteins were measured by Olink Analytical Services in Sweden using the antibody-based Olink Explore 3072 Proximity Extension Assay platform. A total of 2,941 protein analytes, representing 2,923 unique proteins, were measured and reported as normalized protein expression (NPX) values. Further details of sample selection and laboratory procedures have been described previously (5, 6).

The proteomic data released by UK Biobank had undergone quality-control procedures implemented by the UKB-PPP (7). These procedures included removal of Olink control samples, individuals who had withdrawn consent or whose data had not been processed by Olink, potential outlier samples, measurements with QC or assay warnings, and likely sample swaps. Potential outlier samples were assessed using principal component analysis (PCA) and sample-level median and interquartile range (IQR) analyses of NPX values. Samples with PC1 or PC2 values more than 5 standard deviations (SDs) from the mean were identified as outliers. Samples were also identified as outliers if the median NPX value across proteins was more than 5 SDs from the mean median or if the IQR of NPX values across proteins was more than 5 SDs from the mean IQR; samples meeting these criteria were removed during the UKB-PPP quality-control process. As the present study used the quality-controlled proteomic data released by UK Biobank, no additional outlier exclusion was performed. In the present study, proteins with more than 30% missing values were excluded (8)**.** Among the 2,923 unique proteins, GLIPR1, NPM1, and PCOLCE had missing rates of 99.70%, 74.40%, and 63.63%, respectively, and were therefore excluded, leaving 2,920 proteins for subsequent analyses. Remaining missing protein measurements were not imputed. Because each protein was analyzed separately, regression models were fitted using participants with complete data for the corresponding protein and covariates. Thus, participants with a missing value for a particular protein were excluded only from analyses involving that protein, and the sample size could vary slightly across proteins. NPX values were standardized to Z-scores before regression analyses.

**Supplementary Methods 5: Model assumptions and multicollinearity assessment**

The proportional hazards assumption for Cox proportional hazards models was assessed using Schoenfeld residuals. Multicollinearity among covariates was assessed in both the multivariable linear regression and Cox proportional hazards models using generalized variance inflation factors (GVIFs) calculated with the *car* package (version 3.1-3) (9).

**Supplementary Methods 6: Robustness and sensitivity analyses**

Additional robustness analyses were conducted to assess the consistency and stability of the Cox regression findings. For each psychiatric outcome, the analytic cohort was randomly divided into a training set (70%) and a testing set (30%) using stratified random sampling based on outcome status to preserve the proportion of incident cases in both subsets. Cox proportional hazards analyses were then repeated separately in both subsets using the same covariate adjustments as in the primary analyses, and the consistency of the effect estimates in the training and testing sets with those from the primary analyses was evaluated. Additional sensitivity analyses were performed to evaluate the potential influence of medication use on the proteomic associations. Analyses of the associations between workplace noise exposure and proteins and between proteins and anxiety disorder or MDD were repeated after stratification by lipid-lowering and psychotropic medication use. To further assess potential confounding by major somatic conditions, the proteomic association models were additionally adjusted for hypertension, diabetes mellitus, CVD, and CKD. All 2,920 proteins were re-analyzed in each proteomic robustness and sensitivity analysis. To evaluate the consistency of the primary proteomic findings, we focused on proteins reaching FDR <0.05 in the primary analyses and assessed how many remained significant at FDR <0.05 in the corresponding robustness and sensitivity analyses. Their effect estimates were also compared with those from the primary analyses using Pearson correlation coefficients and directional concordance.

**Supplementary Methods 7: Mediation analysis**

Mediation analysis was conducted to investigate the potential role of plasma proteins in mediating the associations between workplace noise exposure and anxiety disorder or MDD. Proteins significantly associated with both workplace noise exposure and the corresponding outcome in the preceding analyses were considered candidate mediators and were evaluated individually using the mediation package (version 4.5.1) (10). The mediation framework was schematically represented as follows:

$$\left\{ \begin{aligned} M=a\times W+d\times C+\varepsilon_{1} \\ D=c^{'}\times W+b\times M+e\times C+\varepsilon_{2} \\ D=c\times W+f\times C+\varepsilon_{3} \end{aligned} \right.$$

where *W* represents workplace noise exposure, *M* represents the plasma protein mediator, *D* represents the corresponding psychiatric outcome, and *C* represents the covariates. The coefficient *a* represents the association between workplace noise exposure and the plasma protein mediator, *b* represents the association between the mediator and the psychiatric outcome conditional on workplace noise exposure and covariates, *c* represents the total association between workplace noise exposure and the psychiatric outcome, and *c′* represents the direct association after accounting for the mediator. The coefficients *d*, *e*, and *f* represent the associations of covariates in the corresponding models. Covariates included age, sex, ethnicity, smoking status, BMI, TDI, Lden, and hearing aid use.

For each candidate protein, the mediator model was fitted using multiple linear regression. For the time-to-event outcome, a Poisson regression model with a log link was fitted, with the natural logarithm of follow-up time included as an offset (11)*.* Mediation effects were estimated using the mediate() function in the R mediation package. The average causal mediation effect (ACME), average direct effect (ADE), total effect, and proportion mediated were estimated for each candidate protein, with statistical inference based on 5,000 nonparametric bootstrap resamples.

To account for multiple testing, ACME *P* values were adjusted across candidate proteins using the Benjamini–Hochberg procedure. A protein was considered a potential mediator if its ACME FDR was <0.05, the total-effect P value was <0.05, and the estimated average proportion mediated was >0.

**Supplementary Methods 8: Functional enrichment analysis**

Functional enrichment analyses were performed for workplace noise-associated proteins and mediator proteins identified for the relevant psychiatric outcomes using STRING v12.0. Gene Ontology (GO) Biological Process, Molecular Function, and Cellular Component terms, Kyoto Encyclopedia of Genes and Genomes (KEGG) pathways, and Reactome pathways were examined. Analyses were conducted for *Homo sapiens* using the default STRING settings, except for the specification of the statistical background, and terms with an FDR-adjusted P value < 0.05 were considered statistically significant.

The proteomic platform initially measured 2,923 unique proteins. GLIPR1, NPM1, and PCOLCE were excluded from the proteomic association analyses because their missingness exceeded 30%, leaving 2,920 proteins available for association testing. Accordingly, these 2,920 proteins were used as the assay-specific statistical background in the primary enrichment analyses, thereby restricting the reference universe to proteins that could potentially have been selected in the present study. To evaluate the influence of the selected reference universe, the enrichment analyses were repeated using the default *Homo sapiens* genome-wide background as a sensitivity analysis. The same input protein sets were used in the primary and sensitivity analyses, with only the statistical background being changed. As an additional robustness analysis, the enrichment analyses were repeated using all 2,923 unique proteins initially measured by the proteomic platform as the statistical background to assess whether the exclusion of the three proteins with high missingness influenced the platform-restricted enrichment results. The significant enrichment terms identified were identical to those obtained using the 2,920-protein background.

**Supplementary Methods 9: Development of WNPRS**

Workplace noise-related protein risk scores (WNPRSs) were developed by integrating proteins identified as mediators of the associations between workplace noise exposure and psychiatric outcomes. A total of 44 shared mediator proteins that met the mediation criteria in both the MDD and anxiety disorder mediation models were selected to construct WNPRS-MDD and WNPRS-Anxiety. Given the substantial comorbidity and potential biological overlap between these disorders, this cross-outcome selection strategy was intended to prioritize mediation signals supported across both outcomes and to provide a common proteomic basis for constructing the two scores. This approach was informed by the previously published APPRS framework, which similarly selected proteins showing significant mediation effects across multiple disease outcomes (12). The inclusion criteria were: ACME FDR <0.05, total-effect *P* <0.05, and a positive estimated average proportion mediated in both the MDD and anxiety disorder mediation models. The WNPRS was then calculated as a weighted sum of the protein expressions associated with workplace noise exposure, with the weights derived from ACME, which can be denoted as:

*WNPRS =*$\frac{\sum_{i}^{M} (ACMEᵢ \times Proteinᵢ）}{\sum_{i}^{M} ACMEᵢ}$

where *ACME_i_* is the coefficient of indirect effects for *i*th protein, Protein*_i_* the expression level of *i*th protein, *M* the total number of selected proteins. For MDD and anxiety disorder, WNPRS-MDD and WNPRS-Anxiety were calculated separately using the 44 shared mediator proteins, with protein levels weighted by the corresponding ACME estimates from the respective mediation models. Thus, although the same protein set was included in both scores, the different mediation-effect weights resulted in distinct WNPRS-MDD and WNPRS-Anxiety scores.

**References**

1.	Li W, Zhu J, Hao X, Chen Z, Wang D. Noise Exposure in the Workplace, Genetic Susceptibility, and Incidence of Atrial Fibrillation: A Prospective Cohort Study. J Am Heart Assoc. 2024;13(21):e036543.

2.	Lu T, Forgetta V, Richards JB, Greenwood CMT. Genetic determinants of polygenic prediction accuracy within a population. Genetics. 2022;222(4).

3.	Lee E, Lee D, Baek JH, Kim SY, Park WY. Transdiagnostic clustering and network analysis for questionnaire-based symptom profiling and drug recommendation in the UK Biobank and a Korean cohort. Sci Rep. 2024;14(1):4500.

4.	Yu HH, Chi HC, Gao PY, Zhang DD, Fu Y, Hu H, et al. The frequency of adding salt to food and incident neuropsychiatric disorders: a large prospective cohort study of 426,832 participants. J Affect Disord. 2025;391:119975.

5.	Sun BB, Chiou J, Traylor M, Benner C, Hsu YH, Richardson TG, et al. Plasma proteomic associations with genetics and health in the UK Biobank. Nature. 2023;622(7982):329-38.

6.	Zhang R, Luo J, Wang T, Wang W, Sun J, Zhang D. Identifying novel protein biomarkers with cross-psychiatric disorders effects and potential intervention targets: Evidence from proteomic-Mendelian randomization. Prog Neuropsychopharmacol Biol Psychiatry. 2025;139:111396.

7.	Sun BB, Ferber K, Lin T, Whelan CD. UK Biobank Pharma Proteomics Project: Olink quality control summary. UK Biobank. Available from: <https://biobank.ctsu.ox.ac.uk/ukb/ukb/docs/PPP_Phase_1_QC_dataset_companion_doc.pdf>.

8.	Wang Z, Ning Y, Gao P, Xu L, Cao S, Li Y, et al. Proteomic signature of dementia risk in type 2 diabetes. J Adv Res. 2026;83:727-40.

9.	Fox J, Weisberg S, Price B. car: Companion to Applied Regression. 3.1-3 ed: CRAN; 2024. p. R package.

10.	Tingley D, Yamamoto T, Hirose K, Keele L, Imai K. mediation: R Package for Causal Mediation Analysis. Journal of Statistical Software. 2014;59(5):1-38.

11.	Austin PC. A Tutorial on Multilevel Survival Analysis: Methods, Models and Applications. Int Stat Rev. 2017;85(2):185-203.

12.	Li W, Li K, Zhou P, Cheng Y, Zhao Y, Meng X, et al. Plasma proteome mediates the associations between air pollution exposure and disease risk. Nat Commun. 2026;17(1).

| **Supplementary Table 1. The codes used for psychiatric disorders.** | | | |
| --- | --- | --- | --- |
| **Psychiatric disorders** | **Source** | **Field ID** | **ICD-10 code** |
| Anxiety disorder | First occurrences | 130904 | F40 (Phobic anxiety disorders) |
|  |  | 130906 | F41 (Other anxiety disorders) |
| Bipolar disorder | First occurrences | 130892 | F31 (Bipolar affective disorder) |
| Major depressive disorder | First occurrences | 130894 | F32 (Depressive episode) |
|  |  | 130896 | F33 (Recurrent depressive disorder) |
| Schizophrenia | First occurrences | 130874 | F20 (Schizophrenia) |

| **Supplementary Table 2. UK Biobank Showcase variables used as covariates in this study.** | | |
| --- | --- | --- |
| **Covariates** | **Field ID** | **Description** |
| Age | 21022 | Age at recruitment |
| Sex | 31 | - |
| Ethnicity (white or non-white) | 21000 | What is your ethnic group? |
| Body mass index (kg/m^2^) | 21001 | BMI value here is constructed from height and weight measured during the initial Assessment Centre visit. |
| Townsend deprivation index | 22189 | Townsend deprivation index calculated immediately prior to participant joining UKB. Based on the preceding national census output areas. Each participant is assigned a score corresponding to the output area in which their postcode is located. |
| Smoking status | 20116 | The current/past smoking status of the participant. |
| Hearing aid use | 3393 | Self-reported hearing aid use recorded during the baseline touchscreen assessment. |
| the weighted average 24-hour traffic noise level | 24020  24021  24022 | Weighted average 24-hour traffic noise level derived from daytime, evening, and night-time sound levels, with penalties of 5 dB and 10 dB added to the evening and night-time periods, respectively. |
| Medication use | 20003 | Self-reported treatment or medication use recorded at baseline. |
|  | 137 | Number of self-reported treatments/medications taken at baseline. |
| Hypertension (yes, or no) | 6150,  4079, 4080,  93, 94, 6177 | Self-reported hypertension history or systolic blood pressure ≥ 140 mmHg or diastolic blood pressure ≥ 90 mm Hg or taking antihypertensive medication. |
| Cardiovascular disease (yes, or no) | 6150,131298,  131300, 131302  131304, 131606 | Self-reported heart attack or angina history, or diagnosis according to International Classification of Diseases, 10th Revision (ICD-10) (I21-I25). |
| Diabetes (yes, or no) | 2443, 6177,  130706, 130708 | Self-reported diabetes history or using medication for diabetes or diagnosis according to ICD-10 (E10, E11). |
| Chronic kidney disease (yes, or no) | 132032 | Diagnosis according to ICD-10 (N18) |

| **Supplementary Table 3. List of lipid-lowering medications and corresponding codes.** | | |
| --- | --- | --- |
| **Drug class** | **Drug name** | **UKB code** |
| Fibrate | Fenofibrate | 1140861954 |
| Statin | Simvastatin | 1140861958 |
|  | Fluvastatin | 1140888594 |
|  | Pravastatin | 1140888648 |
|  | Atorvastatin | 1141146234 |
|  | Rosuvastatin | 1141192410 |
| Cholesterol absorption inhibitor | Ezetimibe | 1141192736 |

| **Supplementary Table 4. List of psychotropic medications and corresponding codes.** | | |
| --- | --- | --- |
| **Drug class** | **Drug name** | **UKB code** |
| **Antidepressant** | citalopram | 1140921600 |
|  | fluoxetine | 1140879540 |
|  | amitriptyline | 1140879616 |
|  | sertraline | 1140867878 |
|  | venlafaxine | 1140916282 |
|  | paroxetine | 1140867888 |
|  | mirtazapine | 1141152732 |
|  | dosulepin | 1140909806 |
|  | escitalopram | 1141180212 |
|  | seroxat 20mg tablet | 1140882236 |
|  | prozac 20mg capsule | 1140867876 |
|  | trazodone | 1140879634 |
|  | st john's wort/hypericum [ctsu] | 1201 |
|  | duloxetine | 1141200564 |
|  | cipralex 5mg tablet | 1141190158 |
|  | lofepramine | 1140867726 |
|  | clomipramine | 1140879620 |
|  | efexor 37.5mg tablet | 1140916288 |
|  | nortriptyline | 1140867818 |
|  | imipramine | 1140879630 |
|  | cipramil 10mg tablet | 1141151946 |
|  | dothiepin | 1140879628 |
|  | prothiaden 25mg capsule | 1140867624 |
|  | reboxetine | 1141151978 |
|  | lustral 50mg tablet | 1140867884 |
|  | trimipramine | 1140867756 |
|  | zispin 30mg tablet | 1141152736 |
|  | amitriptyline hydrochloride+perphenazine 10mg/2mg tablet | 1140867948 |
|  | cymbalta 30mg gastro-resistant capsule | 1141201834 |
|  | anafranil 10mg capsule | 1140867690 |
|  | moclobemide | 1140867920 |
|  | phenelzine | 1140867850 |
|  | fluvoxamine | 1140879544 |
|  | tranylcypromine | 1140867914 |
|  | surmontil 10mg tablet | 1140867758 |
|  | doxepin | 1140867640 |
|  | triptafen tablet | 1140867934 |
|  | yentreve 20mg gastro-resistant capsule | 1141200570 |
|  | nardil 15mg tablet | 1140867852 |
|  | edronax 4mg tablet | 1141151982 |
|  | allegron 10mg tablet | 1140867820 |
|  | mianserin | 1140879556 |
|  | faverin 50mg tablet | 1140867860 |
|  | tranylcypromine+trifluoperazine 10mg/1mg tablet | 1140867944 |
|  | molipaxin 50mg capsule | 1140882244 |
|  | nefazodone | 1140917460 |
|  | gamanil 70mg tablet | 1140882310 |
|  | amitriptyline+chlordiazepoxide 12.5mg/5mg capsule | 1140867938 |
|  | manerix 150mg tablet | 1140867922 |
|  | maoi - tranylcypromine | 1140910820 |
|  | limbitrol 10 capsule | 1140856186 |
|  | norval 10mg tablet | 1140867812 |
|  | tofranil 10mg tablet | 1140867712 |
|  | amoxapine | 1140867774 |
|  | sinequan 10mg capsule | 1140882312 |
| **Antipsychotics** | olanzapine | 1140928916 |
|  | quetiapine | 1141152848 |
|  | risperidone | 1140867444 |
|  | chlorpromazine | 1140879658 |
|  | trifluoperazine | 1140868120 |
|  | amisulpride | 1141153490 |
|  | seroquel 25mg tablet | 1141152860 |
|  | sulpiride | 1140867304 |
|  | aripiprazole | 1141195974 |
|  | haloperidol | 1140867168 |
|  | stelazine 1mg tablet | 1140867244 |
|  | depixol 3mg tablet | 1140867152 |
|  | clozapine | 1140867420 |
|  | flupentixol | 1140909800 |
|  | promazine | 1140879746 |
|  | fluanxol 500micrograms tablet | 1140867952 |
|  | risperdal 0.5mg tablet | 1141177762 |
|  | modecate 12.5mg/0.5ml oily injection | 1140867456 |
|  | zyprexa 2.5mg tablet | 1141167976 |
|  | flupenthixol | 1140867150 |
|  | zuclopenthixol | 1140882100 |
|  | largactil 10mg tablet | 1140863416 |
|  | haldol 5mg tablet | 1140867184 |
|  | abilify 5mg tablet | 1141202024 |
|  | clopixol 2mg tablet | 1140867342 |
|  | clozaril 25mg tablet | 1140882320 |
|  | cpz - chlorpromazine | 1140910358 |
|  | fluphenazine | 1140882098 |
|  | pericyazine | 1140867134 |
|  | fentazin 2mg tablet | 1140867210 |
|  | fluphenazine decanoate | 1140867398 |
|  | dolmatil 200mg tablet | 1140867306 |
|  | levomepromazine | 1140909802 |
|  | perphenazine | 1140867208 |
|  | zaponex 25mg tablet | 1141201792 |
|  | neulactil 2.5mg tablet | 1140867136 |
|  | zotepine | 1141169714 |
|  | serenace 500micrograms capsule | 1140867092 |
|  | thioridazine | 1140879750 |
|  | denzapine 25mg tablet | 1141200458 |
|  | pimozide | 1140867218 |
|  | fluphenazine hydrochloride+nortriptyline 1.5mg/30mg tablet | 1140867940 |
|  | benperidol | 1140867078 |
|  | sertindole | 1140927956 |
| **Mood stabilizer** | lithium product | 1140867490 |
|  | priadel 200mg m/r tablet | 1140867504 |
|  | lamotrigine | 1140872290 |
|  | tegretol 100mg tablet | 1140872072 |
|  | sodium valproate | 1140872198 |
|  | carbamazepine | 2038459704 |
|  | epilim 100mg crushable tablet | 1140872200 |
|  | depakote 250mg e/c tablet | 1141172838 |
|  | topiramate | 1140923484 |
|  | valproic acid | 1140872214 |
|  | lamictal 25mg tablet | 1140872302 |
|  | camcolit 250 tablet | 1140867494 |
|  | carbamazepine product | 1140872064 |
| **Sedative hypnotics** | zopiclone | 1140863144 |
|  | diazepam | 1140863152 |
|  | temazepam | 1140863202 |
|  | zolpidem | 1140865016 |
|  | clonazepam | 1140872150 |
|  | nitrazepam | 1140863182 |
|  | lorazepam | 1140863302 |
|  | zimovane ls 3.75mg tablet | 1140928004 |
|  | valium 2mg tablet | 1140863244 |
|  | oxazepam | 1140863442 |
|  | chlordiazepoxide | 1140863328 |
|  | clobazam | 1140863268 |
|  | loprazolam | 1140863120 |
|  | stilnoct 5mg tablet | 1140864916 |
|  | xanax 250mcg tablet | 1140863310 |
|  | alprazolam | 1140863308 |
|  | diazepam product | 1141157496 |
|  | zaleplon | 1141171404 |
|  | sonata 5mg capsule | 1141171410 |
|  | bromazepam | 1140863318 |
|  | ativan 1mg tablet | 1140863364 |
|  | medazepam | 1140863372 |
|  | valium 10mg suppository | 1140855856 |
|  | mogadon 5mg tablet | 1140863194 |
|  | rohypnol 1mg tablet | 1140863106 |
|  | flurazepam | 1140863110 |
| **Etc** | propranolol | 1140879842 |
|  | gabapentin | 1140872228 |
|  | pregabalin | 1141200004 |
|  | procyclidine | 1140883476 |
|  | clonidine | 1140883468 |
|  | ropinirole | 1140928274 |
|  | nicotine product | 1140872492 |
|  | buspirone | 1140879730 |
|  | inderal 10mg tablet | 1140866804 |
|  | clonidine hydrochloride 25micrograms tablet | 1140871986 |
|  | trihexyphenidyl | 1140909816 |
|  | bupropion | 1141176854 |
|  | buspar 5mg tablet | 1140863454 |
|  | donepezil hydrochloride | 1141150834 |
|  | atomoxetine | 1141199446 |
|  | rivastigmine | 1141167690 |

| **Supplementary Table 5. Baseline Characteristics of Participants in the Anxiety Disorder Analytic Cohort According to Workplace Noise Exposure Duration.** | | | | | |
| --- | --- | --- | --- | --- | --- |
| **Characteristics** | **No exposure** | **< 1 year** | 1-5 **Years** | **>5 years** | **P value** |
|  | **(n= 12,229)** | **(n= 882)** | **(n=905)** | **(n= 1,860)** |  |
| Age (mean ± SD) | 56.54 (8.34) | 55.35 (8.21) | 56.96 (8.13) | 58.92 (7.84) | <0.001 |
| Sex (%) |  |  |  |  |  |
| Female | 7,463 (61.0) | 196 (22.2) | 253 (28.0) | 425 (22.8) | <0.001 |
| Male | 4,766 (39.0) | 686 (77.8) | 652 (72.0) | 1,435 (77.2) |  |
| Ethnicity (%) |  |  |  |  |  |
| Non-White | 1,327 (10.9) | 90 (10.3) | 90 (10.0) | 140 (7.6) | <0.001 |
| White | 10,855 (89.1) | 787 (89.7) | 812 (90.0) | 1,710 (92.4) |  |
| Smoking status (%) |  |  |  |  |  |
| Never | 7,198 (59.0) | 412 (46.8) | 384 (42.6) | 830 (44.9) | <0.001 |
| Previous | 4,046 (33.2) | 347 (39.4) | 367 (40.7) | 800 (43.2) |  |
| Current | 959 (7.9) | 122 (13.8) | 151 (16.7) | 220 (11.9) |  |
| BMI (mean ± SD) | 27.02 (4.66) | 27.60 (4.39) | 28.19 (5.24) | 28.31 (4.57) | <0.001 |
| TDI (mean ± SD) | -1.18 (2.94) | -0.98 (3.03) | -0.38 (3.27) | -0.76 (3.13) | <0.001 |
| Lden (mean ± SD) | 55.99 (4.24) | 56.13 (4.33) | 55.97 (4.28) | 55.85 (4.12) | 0.404 |
| Hearing aid use (%) |  |  |  |  |  |
| No | 11,941 (97.7) | 854 (96.9) | 861 (95.1) | 1,735 (93.4) | <0.001 |
| Yes | 281 (2.3) | 27 (3.1) | 44 (4.9) | 123 (6.6) |  |
| Note: Percentages were calculated based on non-missing values. Category counts may not sum to the total number of participants because of missing data. | | | | | |

| **Supplementary Table 6. Baseline Characteristics of Participants in the MDD Analytic Cohort According to Workplace Noise Exposure Duration.** | | | | | |
| --- | --- | --- | --- | --- | --- |
| **Characteristics** | **No exposure** | **<1 year** | **1-5 years** | **>5 years** | **P value** |
|  | **(n= 12,069)** | **(n= 875)** | **(n= 903)** | **(n= 1,833)** |  |
| Age (mean ± SD) | 56.58 (8.34) | 55.30 (8.26) | 56.85 (8.13) | 58.97 (7.85) | <0.001 |
| Sex (%) |  |  |  |  |  |
| Female | 7,330 (60.7) | 191 (21.8) | 244 (27.0) | 395 (21.5) | <0.001 |
| Male | 4,739 (39.3) | 684 (78.2) | 659 (73.0) | 1,438 (78.5) |  |
| Ethnicity (%) |  |  |  |  |  |
| Non-White | 1,311 (10.9) | 90 (10.3) | 90 (10.0) | 143 (7.8) | 0.001 |
| White | 10,710 (89.1) | 780 (89.7) | 810 (90.0) | 1,681 (92.2) |  |
| Smoking status (%) |  |  |  |  |  |
| Never | 7,116 (59.1) | 412 (47.1) | 386 (42.9) | 809 (44.4) | <0.001 |
| Previous | 3,986 (33.1) | 339 (38.8) | 363 (40.3) | 791 (43.4) |  |
| Current | 940 (7.8) | 123 (14.1) | 151 (16.8) | 224 (12.3) |  |
| BMI (mean ± SD) | 27.05 (4.68) | 27.72 (4.51) | 28.29 (5.26) | 28.30 (4.51) | <0.001 |
| TDI (mean ± SD) | -1.18 (2.94) | -0.96 (3.05) | -0.37 (3.27) | -0.77 (3.14) | <0.001 |
| Lden (mean ± SD) | 55.97 (4.21) | 56.09 (4.31) | 55.88 (4.19) | 55.82 (4.07) | 0.361 |
| Hearing aid use (%) |  |  |  |  |  |
| No | 11,787 (97.7) | 846 (96.8) | 860 (95.2) | 1,705 (93.2) | <0.001 |
| Yes | 275 (2.3) | 28 (3.2) | 43 (4.8) | 125 (6.8) |  |
| Note: Percentages were calculated based on non-missing values. Category counts may not sum to the total number of participants because of missing data. | | | | | |

| **Supplementary Table 7. Baseline Characteristics of Participants in the Bipolar Disorder Analytic Cohort According to Workplace Noise Exposure Duration.** | | | | | |
| --- | --- | --- | --- | --- | --- |
| **Characteristics** | **No exposure** | **<1 year** | **1-5 years** | **>5 years** | **P value** |
|  | **(n= 11,546)** | **(n= 833)** | **(n= 854)** | **(n= 1,729)** |  |
| Age (mean ± SD) | 56.50 (8.34) | 55.30 (8.21) | 56.93 (8.15) | 58.92 (7.87) | <0.001 |
| Sex (%) |  |  |  |  |  |
| Female | 6,954 (60.2) | 173 (20.8) | 228 (26.7) | 379 (21.9) | <0.001 |
| Male | 4,592 (39.8) | 660 (79.2) | 626 (73.3) | 1,350 (78.1) |  |
| Ethnicity (%) |  |  |  |  |  |
| Non-White | 1,262 (11.0) | 86 (10.4) | 87 (10.2) | 134 (7.8) | 0.001 |
| White | 10,238 (89.0) | 742 (89.6) | 764 (89.8) | 1,587 (92.2) |  |
| Smoking status (%) |  |  |  |  |  |
| Never | 6,837 (59.3) | 394 (47.4) | 366 (43.0) | 770 (44.7) | <0.001 |
| Previous | 3,802 (33.0) | 326 (39.2) | 346 (40.7) | 749 (43.5) |  |
| Current | 882 (7.7) | 112 (13.5) | 139 (16.3) | 202 (11.7) |  |
| BMI (mean ± SD) | 26.98 (4.61) | 27.58 (4.34) | 28.25 (5.24) | 28.24 (4.49) | <0.001 |
| TDI (mean ± SD) | -1.21 (2.92) | -1.01 (3.04) | -0.45 (3.25) | -0.85 (3.11) | <0.001 |
| Lden (mean ± SD) | 55.98 (4.22) | 56.08 (4.28) | 55.93 (4.24) | 55.83 (4.11) | 0.474 |
| Hearing aid use (%) |  |  |  |  |  |
| No | 11,278 (97.7) | 808 (97.1) | 814 (95.3) | 1,617 (93.6) | <0.001 |
| Yes | 261 (2.3) | 24 (2.9) | 40 (4.7) | 110 (6.4) |  |
| Note: Percentages were calculated based on non-missing values. Category counts may not sum to the total number of participants because of missing data. | | | | | |

| **Supplementary Table 8. Baseline Characteristics of Participants in the Schizophrenia Analytic Cohort According to Workplace Noise Exposure Duration.** | | | | | |
| --- | --- | --- | --- | --- | --- |
| **Characteristics** | **No exposure** | **<1 year** | **1-5 years** | **>5 years** | **P value** |
|  | **(n= 11,540)** | **(n= 834)** | **(n= 851)** | **(n= 1,731)** |  |
| Age (mean ± SD) | 56.50 (8.34) | 55.27 (8.22) | 56.96 (8.14) | 58.92 (7.87) | <0.001 |
| Sex (%) |  |  |  |  |  |
| Female | 6,945 (60.2) | 172 (20.6) | 225 (26.4) | 380 (22.0) | <0.001 |
| Male | 4,595 (39.8) | 662 (79.4) | 626 (73.6) | 1,351 (78.0) |  |
| Ethnicity (%) |  |  |  |  |  |
| Non-White | 1,259 (11.0) | 87 (10.5) | 86 (10.1) | 134 (7.8) | 0.001 |
| White | 10,235 (89.0) | 742 (89.5) | 762 (89.9) | 1,589 (92.2) |  |
| Smoking status (%) |  |  |  |  |  |
| Never | 6,834 (59.3) | 394 (47.4) | 366 (43.2) | 772 (44.8) | <0.001 |
| Previous | 3,800 (33.0) | 327 (39.3) | 346 (40.8) | 749 (43.5) |  |
| Current | 882 (7.7) | 111 (13.3) | 136 (16.0) | 202 (11.7) |  |
| BMI (mean ± SD) | 26.98 (4.61) | 27.57 (4.32) | 28.26 (5.25) | 28.24 (4.48) | <0.001 |
| TDI (mean ± SD) | -1.21 (2.92) | -1.00 (3.04) | -0.46 (3.25) | -0.85 (3.11) | <0.001 |
| Lden (mean ± SD) | 55.98 (4.22) | 56.10 (4.29) | 55.92 (4.21) | 55.83 (4.10) | 0.408 |
| Hearing aid use (%) |  |  |  |  |  |
| No | 11,272 (97.7) | 809 (97.1) | 811 (95.3) | 1,618 (93.6) | <0.001 |
| Yes | 261 (2.3) | 24 (2.9) | 40 (4.7) | 111 (6.4) |  |
| Note: Percentages were calculated based on non-missing values. Category counts may not sum to the total number of participants because of missing data. | | | | | |

| **Supplementary Table 9. Association between hearing aid use and workplace noise exposure across analytic cohorts.** | | | | | | | | |
| --- | --- | --- | --- | --- | --- | --- | --- | --- |
| **Analytic cohort** | **Hearing aid use (%)** | | | | **Group comparison** | | **Trend analysis** | |
|  | **No exposure** | **<1 year** | **1-5 years** | **>5 years** | **χ²** | **P** | **Trend OR** | **P for trend** |
| Workplace noise–protein analysis | 2.38 | 3.24 | 4.69 | 7.03 | 143.43 | <0.001 | 1.45 | <0.001 |
| Anxiety disorder analytic cohort | 2.30 | 3.06 | 4.86 | 6.62 | 115.32 | <0.001 | 1.45 | <0.001 |
| MDD analytic cohort | 2.28 | 3.20 | 4.76 | 6.83 | 123.29 | <0.001 | 1.46 | <0.001 |
| Bipolar disorder  analytic cohort | 2.26 | 2.88 | 4.68 | 6.37 | 99.84 | <0.001 | 1.44 | <0.001 |
| Schizophrenia analytic cohort | 2.26 | 2.88 | 4.70 | 6.42 | 101.89 | <0.001 | 1.44 | <0.001 |

| **Supplementary Table 10. Proportions of Comorbid Psychiatric Disorders Among Participants with Incident Psychiatric Disorders.** | | | | | | |
| --- | --- | --- | --- | --- | --- | --- |
| **Psychiatric disorder** | **Cases, n** | **Comorbid psychiatric disorder, n (%)** | | | | |
|  |  | **Anxiety** | **MDD** | **Bipolar disorder** | **Schizophrenia** | **Any of the other three disorders** |
| Anxiety disorder | 944 | — | 197 (20.87%) | 11 (1.17%) | 2 (0.21%) | 206 (21.82%) |
| MDD | 748 | 197 (26.34%) | — | 9 (1.20%) | 3 (0.40%) | 205 (27.41%) |
| Bipolar disorder | 30 | 11 (36.67%) | 9 (30.00%) | — | 1 (3.33%) | 18 (60.00%) |
| Schizophrenia | 24 | 2 (8.33%) | 3 (12.50%) | 1 (4.17%) | — | 5 (20.83%) |

| **Supplementary Table 11. Multicollinearity assessment across regression models.** | | | | | |
| --- | --- | --- | --- | --- | --- |
| **Predictor** | **Adjusted GVIF** | | | | |
|  | **Workplace noise–protein analysis** | **Anxiety disorder analytic cohort** | **MDD analytic cohort** | **Bipolar disorder analytic cohort** | **Schizophrenia analytic cohort** |
| Workplace noise | 1.064 | 1.028 | 1.030 | 1.027 | 1.027 |
| Age | 1.039 | 1.039 | 1.040 | 1.056 | 1.054 |
| Sex | 1.054 | 1.067 | 1.070 | 1.057 | 1.057 |
| Ethnicity | 1.061 | 1.054 | 1.056 | 1.097 | 1.053 |
| Smoking status | 1.023 | 1.022 | 1.025 | 1.031 | 1.025 |
| BMI | 1.013 | 1.015 | 1.015 | 1.017 | 1.012 |
| TDI | 1.067 | 1.060 | 1.063 | 1.089 | 1.061 |
| Lden | 1.007 | 1.007 | 1.005 | 1.004 | 1.006 |
| Hearing aid use | 1.010 | 1.013 | 1.015 | 1.015 | 1.031 |
| Note: Values are adjusted generalized variance inflation factors (GVIFs), calculated as GVIF^(1/(2×Df)). | | | | | |

| **Supplementary Table 12. Results of the associations between workplace noise exposure and plasma proteins. (ordinal variable, FDR < 0.05）** | | | | |
| --- | --- | --- | --- | --- |
| **Protein** | **UniProt ID** | **Beta** | **P.value** | **FDR** |
| AARSD1 | Q9BTE6 | -0.022 | **0.004** | **0.046** |
| ACE2 | Q9BYF1 | 0.024 | 6.74E-04 | 0.014 |
| ACHE | P22303 | -0.027 | 7.31E-04 | 0.015 |
| ACP1 | P24666 | -0.029 | 7.98E-04 | 0.016 |
| ACP5 | P13686 | 0.033 | 1.17E-05 | 0.001 |
| ACRV1 | P26436 | 0.02 | 2.23E-04 | 0.007 |
| ADA2 | Q9NZK5 | 0.026 | 5.91E-04 | 0.013 |
| ADAM22 | Q9P0K1 | -0.035 | 4.12E-06 | 3.33E-04 |
| ADGRG1 | Q9Y653 | 0.027 | 1.39E-04 | 0.005 |
| AFP | P02771 | -0.032 | 2.15E-05 | 0.001 |
| ALDH3A1 | P30838 | 0.032 | 1.46E-05 | 0.001 |
| ALPP | P05187 | 0.026 | 2.34E-04 | 0.007 |
| AMBP | P02760 | 0.028 | 7.00E-05 | 0.003 |
| AMPD3 | Q01432 | -0.029 | 6.87E-04 | 0.015 |
| ANGPTL4 | Q9BY76 | 0.024 | 0.002 | 0.024 |
| ANXA10 | Q9UJ72 | 0.035 | 5.94E-06 | 4.28E-04 |
| AREG | P15514 | 0.031 | 5.68E-05 | 0.002 |
| ARG1 | P05089 | -0.028 | 2.79E-04 | 0.008 |
| ARSA | P15289 | 0.03 | 7.11E-05 | 0.003 |
| ART3 | Q13508 | -0.029 | 2.46E-05 | 0.001 |
| ASGR1 | P07306 | 0.026 | 2.30E-04 | 0.007 |
| BGLAP | P02818 | 0.027 | 9.41E-04 | 0.018 |
| BOC | Q9BWV1 | -0.022 | 0.003 | 0.046 |
| BPIFA2 | Q96DR5 | -0.026 | 0.001 | 0.023 |
| BPIFB2 | Q8N4F0 | 0.023 | 0.002 | 0.031 |
| BST2 | Q10589 | 0.049 | 8.72E-11 | 6.35E-08 |
| C1R | P00736 | 0.03 | 2.90E-04 | 0.008 |
| C1RL | Q9NZP8 | 0.024 | 0.003 | 0.039 |
| C2 | P06681 | 0.023 | 0.002 | 0.03 |
| C3 | P01024 | 0.028 | 7.57E-04 | 0.015 |
| C9orf40 | Q8IXQ3 | -0.028 | 0.001 | 0.019 |
| CA14 | Q9ULX7 | -0.023 | 0.001 | 0.019 |
| CA6 | P23280 | -0.033 | 6.02E-06 | 4.28E-04 |
| CAT | P04040 | -0.026 | 0.002 | 0.031 |
| CCL17 | Q92583 | 0.023 | 0.002 | 0.034 |
| CCL18 | P55774 | 0.041 | 4.03E-08 | 8.39E-06 |
| CCL20 | P78556 | 0.045 | 1.76E-09 | 5.74E-07 |
| CCL21 | O00585 | 0.027 | 3.94E-04 | 0.009 |
| CCL22 | O00626 | 0.03 | 5.49E-05 | 0.002 |
| CCL5 | P13501 | 0.029 | 1.89E-04 | 0.006 |
| CD14 | P08571 | 0.033 | 1.10E-05 | 0.001 |
| CD163 | Q86VB7 | 0.024 | 0.001 | 0.021 |
| CD27 | P26842 | 0.024 | 0.001 | 0.024 |
| CD300LG | Q6UXG3 | -0.024 | 0.001 | 0.023 |
| CD302 | Q8IX05 | 0.029 | 5.87E-05 | 0.002 |
| CD72 | P21854 | 0.024 | 0.003 | 0.037 |
| CD80 | P33681 | 0.03 | 1.71E-04 | 0.005 |
| CD83 | Q01151 | 0.034 | 4.38E-06 | 3.36E-04 |
| CD99L2 | Q8TCZ2 | 0.037 | 4.91E-07 | 6.37E-05 |
| CDCP1 | Q9H5V8 | 0.086 | 1.02E-35 | 2.97E-32 |
| CDHR2 | Q9BYE9 | -0.019 | 0.002 | 0.026 |
| CEACAM5 | P06731 | 0.025 | 0.001 | 0.021 |
| CELA2A | P08217 | -0.026 | 0.002 | 0.031 |
| CFH | P08603 | 0.022 | 0.004 | 0.046 |
| CHGA | P10645 | 0.033 | 4.45E-05 | 0.002 |
| CHI3L1 | P36222 | 0.024 | 9.17E-04 | 0.018 |
| CHIT1 | Q13231 | 0.028 | 2.87E-04 | 0.008 |
| CILP | O75339 | -0.027 | 7.69E-04 | 0.016 |
| CLEC4A | Q9UMR7 | -0.033 | 1.30E-05 | 0.001 |
| CNTN1 | Q12860 | -0.023 | 0.001 | 0.022 |
| CNTN5 | O94779 | -0.034 | 1.94E-06 | 1.77E-04 |
| COL18A1 | P39060 | 0.022 | 0.002 | 0.031 |
| COL1A1 | P02452 | -0.023 | 0.002 | 0.035 |
| COL28A1 | Q2UY09 | -0.029 | 5.68E-04 | 0.012 |
| COX5B | P10606 | 0.024 | 0.002 | 0.034 |
| CRHBP | P24387 | 0.024 | 9.18E-04 | 0.018 |
| CTHRC1 | Q96CG8 | -0.033 | 4.28E-06 | 3.36E-04 |
| CTSD | P07339 | 0.031 | 4.01E-05 | 0.002 |
| CXCL12 | P48061 | 0.024 | 0.002 | 0.034 |
| CXCL14 | O95715 | 0.044 | 2.76E-09 | 8.03E-07 |
| CXCL17 | Q6UXB2 | 0.039 | 1.74E-09 | 5.74E-07 |
| DCBLD2 | Q96PD2 | 0.027 | 3.29E-04 | 0.008 |
| DLK1 | P80370 | -0.022 | 0.003 | 0.041 |
| DNAJA4 | Q8WW22 | -0.035 | 3.52E-05 | 0.002 |
| DPP7 | Q9UHL4 | 0.03 | 8.94E-05 | 0.003 |
| F7 | P08709 | 0.022 | 0.004 | 0.046 |
| FCAR | P24071 | 0.027 | 5.17E-04 | 0.012 |
| FETUB | Q9UGM5 | 0.029 | 1.12E-04 | 0.004 |
| FGF23 | Q9GZV9 | 0.033 | 6.23E-06 | 4.32E-04 |
| FRZB | Q92765 | 0.022 | 0.003 | 0.043 |
| GALNT2 | Q10471 | -0.026 | 5.85E-04 | 0.013 |
| GAST | P01350 | 0.045 | 3.08E-08 | 6.91E-06 |
| GDF15 | Q99988 | 0.033 | 3.09E-07 | 4.73E-05 |
| GDF2 | Q9UK05 | 0.026 | 2.46E-04 | 0.007 |
| GFRAL | Q6UXV0 | -0.038 | 4.00E-06 | 3.33E-04 |
| GGH | Q92820 | 0.024 | 0.001 | 0.021 |
| GIMAP7 | Q8NHV1 | -0.024 | 0.004 | 0.048 |
| GLA | P06280 | 0.029 | 3.61E-04 | 0.009 |
| GP2 | P55259 | -0.031 | 5.68E-05 | 0.002 |
| GPNMB | Q14956 | 0.034 | 9.28E-06 | 0.001 |
| GPR15L | Q6UWK7 | 0.035 | 7.13E-06 | 4.83E-04 |
| GPR37 | O15354 | 0.033 | 1.57E-05 | 0.001 |
| GUSB | P08236 | 0.027 | 8.39E-05 | 0.003 |
| GZMB | P10144 | -0.023 | 0.002 | 0.033 |
| HAGH | Q16775 | -0.027 | 4.05E-04 | 0.009 |
| HAVCR1 | Q96D42 | 0.025 | 3.60E-04 | 0.009 |
| HAVCR2 | Q8TDQ0 | 0.031 | 1.65E-05 | 0.001 |
| HBQ1 | P09105 | -0.025 | 0.001 | 0.019 |
| HHEX | Q03014 | 0.026 | 0.001 | 0.024 |
| HYAL1 | Q12794 | 0.034 | 1.02E-05 | 0.001 |
| ICAM1 | P05362 | 0.033 | 7.66E-06 | 0.001 |
| ICAM5 | Q9UMF0 | 0.029 | 8.19E-05 | 0.003 |
| IDUA | P35475 | 0.032 | 2.82E-05 | 0.001 |
| IFI30 | P13284 | 0.024 | 0.003 | 0.036 |
| IGDCC4 | Q8TDY8 | -0.025 | 0.001 | 0.024 |
| IL12RB1 | P42701 | 0.025 | 0.001 | 0.023 |
| IL18 | Q14116 | 0.024 | 0.002 | 0.026 |
| IL18BP | O95998 | 0.031 | 2.69E-05 | 0.001 |
| IL1RL2 | Q9HB29 | -0.024 | 0.001 | 0.021 |
| IL1RN | P18510 | 0.025 | 2.55E-04 | **0.007** |
| IL7R | P16871 | -0.023 | **0.003** | 0.041 |
| INSL3 | P51460 | -0.017 | 8.41E-05 | 0.003 |
| INSR | P06213 | 0.029 | 5.65E-04 | 0.012 |
| ITGA5 | P08648 | 0.026 | 3.70E-04 | 0.009 |
| ITIH3 | Q06033 | 0.025 | 7.07E-04 | 0.015 |
| ITIH4 | Q14624 | 0.04 | 6.61E-07 | 7.70E-05 |
| KEL | P23276 | -0.022 | 0.004 | 0.046 |
| KITLG | P21583 | -0.024 | 0.002 | 0.026 |
| KLK4 | Q9Y5K2 | 0.028 | 1.11E-04 | 0.004 |
| KYNU | Q16719 | 0.023 | 0.002 | 0.030 |
| L1CAM | P32004 | 0.023 | 0.002 | 0.033 |
| LAMP3 | Q9UQV4 | 0.035 | 4.43E-07 | 6.14E-05 |
| LEPR | P48357 | 0.030 | 5.35E-05 | 0.002 |
| LGALS3 | P17931 | 0.027 | 2.27E-04 | 0.007 |
| LGALS3BP | Q08380 | 0.036 | 5.96E-06 | 4.28E-04 |
| LGALS4 | P56470 | 0.026 | 4.38E-04 | 0.01 |
| LGALS9 | O00182 | 0.033 | 1.63E-06 | 1.54E-04 |
| LILRB4 | Q8NHJ6 | 0.031 | 2.73E-05 | 0.001 |
| LRRC25 | Q8N386 | 0.027 | 3.10E-04 | 0.008 |
| LTA4H | P09960 | 0.024 | 0.002 | 0.024 |
| LTBP3 | Q9NS15 | 0.024 | 0.001 | 0.023 |
| MANSC4 | A6NHS7 | 0.054 | 2.09E-10 | 1.22E-07 |
| MEGF9 | Q9H1U4 | 0.024 | 0.001 | 0.024 |
| MEP1B | Q16820 | -0.025 | 0.001 | 0.020 |
| MFAP4 | P55083 | -0.026 | 0.002 | 0.031 |
| FGA | P02671 | -0.026 | 2.33E-04 | 0.007 |
| MILR1 | Q7Z6M3 | 0.031 | 8.01E-05 | 0.003 |
| MMP12 | P39900 | 0.04 | 1.09E-08 | 2.65E-06 |
| MMP7 | P09237 | 0.025 | 0.001 | 0.020 |
| MRC1 | P22897 | 0.034 | 1.64E-05 | 0.001 |
| MSLN | Q13421 | 0.022 | 0.002 | 0.034 |
| MSR1 | P21757 | 0.042 | 1.77E-09 | 5.74E-07 |
| MSTN | O14793 | -0.025 | 4.54E-04 | 0.010 |
| MZB1 | Q8WU39 | 0.043 | 6.53E-09 | 1.73E-06 |
| NAPRT | Q6XQN6 | -0.027 | 0.001 | 0.022 |
| NCS1 | P62166 | 0.022 | 0.002 | 0.031 |
| NHLRC3 | Q5JS37 | 0.035 | 1.24E-05 | 0.001 |
| NOMO1 | Q15155 | 0.028 | 1.67E-04 | 0.005 |
| NOTCH3 | Q9UM47 | -0.021 | 0.003 | 0.044 |
| NPL | Q9BXD5 | 0.027 | 2.93E-04 | 0.008 |
| NTRK3 | Q16288 | -0.036 | 5.46E-07 | 6.63E-05 |
| OCLN | Q16625 | 0.028 | 3.25E-04 | 0.008 |
| OMD | Q99983 | -0.036 | 1.40E-06 | 1.38E-04 |
| OSCAR | Q8IYS5 | 0.027 | 4.04E-04 | 0.009 |
| OSMR | Q99650 | 0.036 | 1.42E-06 | 1.38E-04 |
| PI16 | Q6UXB8 | -0.025 | **0.002** | **0.031** |
| PIGR | P01833 | 0.046 | 3.84E-10 | 1.87E-07 |
| PILRA | Q9UKJ1 | 0.03 | 4.78E-05 | 0.002 |
| PILRB | Q9UKJ0 | 0.029 | 1.63E-04 | 0.005 |
| PKLR | P30613 | -0.026 | 9.52E-04 | 0.018 |
| PLA2G15 | Q8NCC3 | 0.027 | 2.87E-04 | 0.008 |
| PLA2G2A | P14555 | 0.024 | **0.001** | 0.020 |
| PLAUR | Q03405 | 0.035 | 9.89E-07 | 1.08E-04 |
| PLTP | P55058 | -0.027 | 2.44E-04 | 0.007 |
| PNLIPRP2 | P54317 | -0.025 | 0.001 | 0.024 |
| PON1 | P27169 | -0.024 | 0.004 | 0.046 |
| PON2 | Q15165 | 0.025 | 0.001 | 0.023 |
| PRCP | P42785 | 0.024 | 9.62E-04 | 0.018 |
| PRDX2 | P32119 | -0.032 | 1.61E-04 | 0.005 |
| PRKAR1A | P10644 | 0.023 | 0.004 | 0.048 |
| PRSS8 | Q16651 | 0.037 | 6.61E-08 | 1.28E-05 |
| PTPRF | P10586 | -0.032 | 1.92E-05 | 0.001 |
| PVR | P15151 | 0.023 | 0.003 | 0.036 |
| RARRES2 | Q99969 | 0.034 | 1.00E-06 | 1.08E-04 |
| REG3A | Q06141 | 0.028 | 1.49E-04 | 0.005 |
| REG4 | Q9BYZ8 | 0.027 | 3.05E-04 | 0.008 |
| REN | P00797 | 0.037 | 3.38E-07 | 4.93E-05 |
| RGMA | Q96B86 | -0.027 | 3.65E-04 | 0.009 |
| RGS8 | P57771 | 0.029 | 1.83E-04 | 0.006 |
| ROBO1 | Q9Y6N7 | 0.028 | 1.74E-04 | 0.005 |
| SCGB1A1 | P11684 | -0.035 | 2.29E-06 | 2.02E-04 |
| SCGN | O76038 | 0.024 | 0.002 | **0.030** |
| SCT | P09683 | -0.041 | 1.32E-06 | 1.38E-04 |
| SERPIND1 | P05546 | 0.029 | 3.37E-04 | **0.008** |
| SIGLEC1 | Q9BZZ2 | 0.023 | **0.002** | 0.024 |
| SIGLEC7 | Q9Y286 | 0.026 | 3.59E-04 | 0.009 |
| SIGLEC9 | Q9Y336 | 0.039 | 2.89E-07 | 4.73E-05 |
| SIRPB1 | O00241 | 0.024 | 0.002 | 0.026 |
| SIT1 | Q9Y3P8 | 0.023 | 0.002 | 0.031 |
| SLAMF7 | Q9NQ25 | 0.023 | 0.002 | 0.033 |
| SLITRK2 | Q9H156 | 0.023 | 0.002 | 0.030 |
| SMPD1 | P17405 | 0.023 | 0.003 | 0.040 |
| SOD2 | P04179 | 0.024 | 0.002 | 0.024 |
| SPINK6 | Q6UWN8 | 0.032 | 8.14E-06 | 0.001 |
| SPINT3 | P49223 | -0.018 | 2.48E-05 | 0.001 |
| SPTLC1 | O15269 | 0.025 | **0.003** | 0.046 |
| SSC4D | Q8WTU2 | -0.026 | 9.91E-05 | 0.003 |
| SUSD5 | O60279 | -0.027 | 5.21E-04 | 0.012 |
| TCN2 | P20062 | 0.027 | 3.91E-04 | 0.009 |
| TFF1 | P04155 | 0.057 | 9.92E-15 | 1.45E-11 |
| TFF2 | Q03403 | 0.038 | 1.56E-07 | 2.84E-05 |
| TFF3 | Q07654 | 0.022 | **0.004** | 0.047 |
| TG | P01266 | -0.036 | 1.50E-05 | 0.001 |
| TGFBR3 | Q03167 | -0.022 | 0.003 | 0.046 |
| TNC | P24821 | 0.024 | 0.001 | 0.024 |
| TNF | P01375 | 0.025 | 9.37E-04 | 0.018 |
| TNFRSF10A | O00220 | 0.024 | 7.14E-04 | 0.015 |
| TNFRSF10B | O14763 | 0.036 | 3.07E-07 | 4.73E-05 |
| TNFRSF12A | Q9NP84 | 0.022 | 0.002 | 0.030 |
| TNFRSF1B | P20333 | 0.022 | 0.003 | 0.039 |
| TNFSF11 | O14788 | -0.028 | 2.11E-04 | 0.006 |
| TNFSF13B | Q9Y275 | 0.026 | 3.87E-04 | 0.009 |
| TNFSF8 | P32971 | 0.029 | 4.54E-04 | 0.010 |
| TNR | Q92752 | -0.037 | 5.03E-07 | 6.37E-05 |
| TP53BP1 | Q12888 | -0.028 | 7.76E-04 | 0.016 |
| TPP1 | O14773 | 0.023 | **0.002** | 0.033 |
| TRAF3 | Q13114-2 | -0.029 | 6.61E-04 | 0.014 |
| TREM2 | Q9NZC2 | 0.052 | 2.76E-13 | 2.68E-10 |
| TXNDC15 | Q96J42 | 0.034 | 1.55E-05 | 0.001 |
| TYRP1 | P17643 | 0.032 | 1.24E-04 | 0.004 |
| UBAC1 | Q9BSL1 | -0.026 | 7.15E-04 | 0.015 |
| UHRF2 | Q96PU4 | 0.026 | **0.003** | 0.039 |
| ULBP2 | Q9BZM5 | 0.025 | 9.87E-04 | 0.019 |
| VIT | Q6UXI7 | -0.029 | 1.44E-04 | 0.005 |
| VSIG2 | Q96IQ7 | 0.034 | 2.53E-05 | 0.001 |
| VWC2 | Q2TAL6 | 0.034 | 2.70E-06 | 2.31E-04 |
| WFDC12 | Q8WWY7 | 0.025 | 3.07E-04 | 0.008 |
| WFDC2 | Q14508 | 0.03 | 2.51E-05 | 0.001 |
| XPNPEP2 | O43895 | -0.025 | **0.001** | 0.022 |

| **Supplementary Table 13. Results of the associations between workplace noise exposure and plasma proteins（no noise vs < 1 year, FDR < 0.05）** | | | | |
| --- | --- | --- | --- | --- |
| **Protein** | **UniProt ID** | **Beta** | **P.value** | **FDR** |
| ARG1 | P05089 | 0.141 | <0.001 | 0.009 |

| **Supplementary Table 14. Results of the associations between workplace noise exposure and plasma proteins（no noise vs 1-5 years, FDR < 0.05）** | | | | |
| --- | --- | --- | --- | --- |
| **Protein** | **UniProt ID** | **Beta** | **P.value** | **FDR** |
| CDCP1 | Q9H5V8 | -0.131 | 7.28E-06 | 0.004 |
| TREM2 | Q9NZC2 | -0.124 | 4.08E-05 | 0.011 |
| VWC2 | Q2TAL6 | -0.128 | 3.88E-05 | 0.011 |
| MILR1 | Q7Z6M3 | -0.132 | 6.42E-05 | 0.015 |
| MANSC4 | A6NHS7 | -0.141 | 8.18E-05 | 0.018 |
| TFF1 | P04155 | -0.123 | 1.00E-04 | 0.021 |
| GGH | Q92820 | -0.118 | 1.30E-04 | 0.025 |
| ALPP | P05187 | -0.115 | 1.46E-04 | 0.027 |
| CLEC4A | Q9UMR7 | 0.124 | 1.48E-04 | 0.027 |
| HMBS | P08397 | 0.120 | 2.52E-04 | 0.040 |
| CXCL17 | Q6UXB2 | -0.100 | 2.74E-04 | 0.044 |

| **Supplementary Table 15. Results of the associations between workplace noise exposure and plasma proteins（no noise vs > 5 years, FDR < 0.05）** | | | | |
| --- | --- | --- | --- | --- |
| **Protein** | **UniProt ID** | **Beta** | **P.value** | **FDR** |
| BST2 | Q10589 | -0.145 | 1.74E-09 | 7.63E-06 |
| CCL18 | P55774 | -0.130 | 4.22E-08 | 5.68E-05 |
| CCL20 | P78556 | -0.133 | 2.78E-08 | 4.43E-05 |
| CDCP1 | Q9H5V8 | -0.270 | 1.24E-34 | 2.17E-30 |
| CXCL14 | O95715 | -0.132 | 2.26E-08 | 3.96E-05 |
| CXCL17 | Q6UXB2 | -0.108 | 1.31E-07 | 1.35E-04 |
| GAST | P01350 | -0.146 | 1.68E-08 | 3.53E-05 |
| GDF15 | Q99988 | -0.108 | 1.90E-07 | 1.75E-04 |
| MANSC4 | A6NHS7 | -0.152 | 1.81E-08 | 3.53E-05 |
| MMP12 | P39900 | -0.126 | 1.80E-08 | 3.53E-05 |
| MSR1 | P21757 | -0.123 | 4.72E-08 | 5.90E-05 |
| MZB1 | Q8WU39 | -0.131 | 3.35E-08 | 4.88E-05 |
| PIGR | P01833 | -0.136 | 8.92E-09 | 2.60E-05 |
| PRSS8 | Q16651 | -0.116 | 9.41E-08 | 1.03E-04 |
| REN | P00797 | -0.126 | 6.93E-08 | 8.08E-05 |
| SIGLEC9 | Q9Y336 | -0.128 | 1.58E-07 | 1.54E-04 |
| TFF1 | P04155 | -0.170 | 5.87E-13 | 5.13E-09 |
| TREM2 | Q9NZC2 | -0.15 | 3.02E-11 | 1.76E-07 |
| SCT | P09683 | 0.134 | 6.47E-07 | 0.001 |
| TNFRSF10B | O14763 | -0.112 | 8.28E-07 | 0.001 |
| TNR | Q92752 | 0.116 | 1.09E-06 | 0.001 |
| CD99L2 | Q8TCZ2 | -0.111 | 2.64E-06 | 0.002 |
| LGALS3BP | Q08380 | -0.120 | 2.39E-06 | 0.002 |
| OMD | Q99983 | 0.109 | 3.70E-06 | 0.002 |
| OSMR | Q99650 | -0.11 | 3.70E-06 | 0.002 |
| ADAM22 | Q9P0K1 | 0.112 | 4.76E-06 | 0.003 |
| GFRAL | Q6UXV0 | 0.119 | 5.56E-06 | 0.003 |
| GPR15L | Q6UWK7 | -0.116 | 4.03E-06 | 0.003 |
| HYAL1 | Q12794 | -0.111 | 6.45E-06 | 0.003 |
| ITIH4 | Q14624 | -0.116 | 4.99E-06 | 0.003 |
| PLAUR | Q03405 | -0.104 | 5.23E-06 | 0.003 |
| TFF2 | Q03403 | -0.108 | 3.86E-06 | 0.003 |
| TXNDC15 | Q96J42 | -0.113 | 5.96E-06 | 0.003 |
| AFP | P02771 | 0.109 | 8.01E-06 | 0.004 |
| GPNMB | Q14956 | -0.111 | 7.16E-06 | 0.004 |
| LGALS9 | O00182 | -0.097 | 8.30E-06 | 0.004 |
| NTRK3 | Q16288 | 0.103 | 9.02E-06 | 0.004 |
| CNTN5 | O94779 | 0.100 | 1.23E-05 | 0.005 |
| ICAM1 | P05362 | -0.105 | 1.07E-05 | 0.005 |
| LAMP3 | Q9UQV4 | -0.098 | 1.30E-05 | 0.005 |
| LILRB4 | Q8NHJ6 | -0.101 | 1.35E-05 | 0.005 |
| RARRES2 | Q99969 | -0.098 | 1.10E-05 | 0.005 |
| VIT | Q6UXI7 | 0.106 | 1.09E-05 | 0.005 |
| ANXA10 | Q9UJ72 | -0.106 | 1.64E-05 | 0.006 |
| CTHRC1 | Q96CG8 | 0.100 | 1.49E-05 | 0.006 |
| SCGB1A1 | P11684 | 0.102 | 1.53E-05 | 0.006 |
| SPINK6 | Q6UWN8 | -0.100 | 1.73E-05 | 0.006 |
| CD14 | P08571 | -0.103 | 2.10E-05 | 0.007 |
| FGF23 | Q9GZV9 | -0.099 | 1.96E-05 | 0.007 |
| KLK4 | Q9Y5K2 | -0.099 | 1.98E-05 | 0.007 |
| SUSD5 | O60279 | 0.105 | 1.89E-05 | 0.007 |
| ART3 | Q13508 | 0.094 | 2.54E-05 | 0.008 |
| RGS8 | P57771 | -0.106 | 2.41E-05 | 0.008 |
| CTSD | P07339 | -0.099 | 3.07E-05 | 0.009 |
| ADGRG1 | Q9Y653 | -0.094 | 3.46E-05 | 0.01 |
| CA6 | P23280 | 0.096 | 3.22E-05 | 0.01 |
| CD83 | Q01151 | -0.097 | 3.98E-05 | 0.011 |
| LGALS4 | P56470 | -0.097 | 3.81E-05 | 0.011 |
| PTPRF | P10586 | 0.097 | 3.89E-05 | 0.011 |
| HAVCR2 | Q8TDQ0 | -0.094 | 5.25E-05 | 0.013 |
| WFDC2 | Q14508 | -0.092 | 4.86E-05 | 0.013 |
| ACP5 | P13686 | -0.097 | 5.47E-05 | 0.014 |
| RGMA | Q96B86 | 0.098 | 5.78E-05 | 0.014 |
| ALDH3A1 | P30838 | -0.095 | 6.48E-05 | 0.015 |
| HAVCR1 | Q96D42 | -0.089 | 6.40E-05 | 0.015 |
| NHLRC3 | Q5JS37 | -0.104 | 6.48E-05 | 0.015 |
| AREG | P15514 | -0.099 | 6.86E-05 | 0.016 |
| NPL | Q9BXD5 | -0.094 | 7.15E-05 | 0.016 |
| IL18BP | O95998 | -0.092 | 8.25E-05 | 0.018 |
| MRC1 | P22897 | -0.098 | 8.39E-05 | 0.018 |
| PILRA | Q9UKJ1 | -0.093 | 8.41E-05 | 0.018 |
| TYRP1 | P17643 | -0.105 | 8.00E-05 | 0.018 |
| GUSB | P08236 | -0.086 | 9.19E-05 | 0.019 |
| TG | P01266 | 0.104 | 9.19E-05 | 0.019 |
| SSC4D | Q8WTU2 | 0.083 | 1.01E-04 | 0.021 |
| CCL21 | O00585 | -0.094 | 1.11E-04 | 0.022 |
| LEPR | P48357 | -0.091 | 1.12E-04 | 0.022 |
| REG4 | Q9BYZ8 | -0.09 | 1.22E-04 | 0.024 |
| PILRB | Q9UKJ0 | -0.093 | 1.27E-04 | 0.025 |
| ICAM5 | Q9UMF0 | -0.091 | 1.39E-04 | 0.026 |
| SPINT3 | P49223 | 0.052 | 1.40E-04 | 0.026 |
| IDUA | P35475 | -0.093 | 1.43E-04 | 0.027 |
| INSL3 | P51460 | 0.053 | 1.53E-04 | 0.027 |
| FETUB | Q9UGM5 | -0.090 | 1.56E-04 | 0.028 |
| FGA | P02671 | 0.085 | 1.70E-04 | 0.029 |
| NOMO1 | Q15155 | -0.089 | 1.66E-04 | 0.029 |
| AMBP | P02760 | -0.084 | 2.02E-04 | 0.034 |
| TCN2 | P20062 | -0.088 | 2.16E-04 | 0.036 |
| VWC2 | Q2TAL6 | -0.087 | 2.12E-04 | 0.036 |
| GP2 | P55259 | 0.090 | 2.37E-04 | 0.039 |
| ARSA | P15289 | -0.087 | 2.50E-04 | 0.04 |
| ASGR1 | P07306 | -0.083 | 2.46E-04 | 0.04 |
| CHGA | P10645 | -0.095 | 2.88E-04 | 0.045 |
| LGALS3 | P17931 | -0.085 | 2.92E-04 | 0.045 |
| TNF | P01375 | -0.089 | 2.90E-04 | 0.045 |
| CCL5 | P13501 | -0.089 | 3.01E-04 | 0.046 |
| CHIT1 | Q13231 | -0.088 | 3.05E-04 | 0.046 |
| GPR37 | O15354 | -0.087 | 3.06E-04 | 0.046 |

| **Supplementary Table 16. Functional enrichment analysis of plasma proteins associated with workplace noise exposure using the 2,920 proteins available for proteomic association testing as the assay-specific background.** | | | | | | | |
| --- | --- | --- | --- | --- | --- | --- | --- |
| **Category** | **Term ID** | **Term description** | **Observed gene count** | **Background gene count** | **Strength** | **Signal** | **FDR** |
| GO Component | GO:0005576 | Extracellular region | 159 | 1447 | 0.14 | 0.50 | 8.05E-06 |
| GO Component | GO:0005615 | Extracellular space | 132 | 1195 | 0.14 | 0.42 | 0.001 |
| GO Component | GO:0031224 | Intrinsic component of membrane | 105 | 957 | 0.14 | 0.28 | 0.026 |
| GO Component | GO:0009986 | Cell surface | 55 | 421 | 0.22 | 0.26 | 0.048 |
| GO Component | GO:0016021 | Integral component of membrane | 97 | 890 | 0.14 | 0.25 | 0.049 |

| **Supplementary Table 17. Functional enrichment analysis of plasma proteins associated with workplace noise exposure using the default *Homo sapiens* genome-wide background as a sensitivity analysis.** | | | | | | | |
| --- | --- | --- | --- | --- | --- | --- | --- |
| **Category** | **Term ID** | **Term description** | **Observed gene count** | **Background gene count** | **Strength** | **Signal** | **FDR** |
| GO Process | GO:0009605 | Response to external stimulus | 87 | 2355 | 0.5 | 1.34 | 1.55E-19 |
| GO Process | GO:0044419 | Biological process involved in interspecies interaction between organisms | 66 | 1490 | 0.58 | 1.48 | 1.48E-17 |
| GO Process | GO:0006952 | Defense response | 63 | 1394 | 0.59 | 1.49 | 3.96E-17 |
| GO Process | GO:0006955 | Immune response | 60 | 1321 | 0.59 | 1.47 | 2.58E-16 |
| GO Process | GO:0009607 | Response to biotic stimulus | 61 | 1375 | 0.58 | 1.44 | 2.84E-16 |
| GO Process | GO:0051707 | Response to other organism | 59 | 1328 | 0.58 | 1.42 | 1.05E-15 |
| GO Process | GO:0002376 | Immune system process | 73 | 2121 | 0.47 | 1.17 | 1.59E-14 |
| GO Process | GO:0002682 | Regulation of immune system process | 59 | 1438 | 0.55 | 1.31 | 2.58E-14 |
| GO Process | GO:0098542 | Defense response to other organism | 48 | 989 | 0.62 | 1.43 | 9.57E-14 |
| GO Process | GO:0050896 | Response to stimulus | 153 | 7835 | 0.23 | 0.75 | 1.56E-13 |
| GO Process | GO:0007155 | Cell adhesion | 47 | 965 | 0.62 | 1.43 | 1.57E-13 |
| GO Process | GO:0048583 | Regulation of response to stimulus | 100 | 3931 | 0.34 | 0.91 | 4.97E-13 |
| GO Process | GO:0006950 | Response to stress | 91 | 3358 | 0.37 | 0.95 | 5.21E-13 |
| GO Process | GO:0034097 | Response to cytokine | 41 | 804 | 0.64 | 1.4 | 3.49E-12 |
| GO Process | GO:0048584 | Positive regulation of response to stimulus | 68 | 2131 | 0.44 | 1.04 | 6.56E-12 |
| GO Process | GO:0002684 | Positive regulation of immune system process | 41 | 874 | 0.61 | 1.28 | 4.45E-11 |
| GO Process | GO:0042221 | Response to chemical | 96 | 4010 | 0.32 | 0.82 | 1.01E-10 |
| GO Process | GO:0030155 | Regulation of cell adhesion | 38 | 784 | 0.62 | 1.27 | 1.47E-10 |
| GO Process | GO:0045087 | Innate immune response | 37 | 754 | 0.63 | 1.27 | 2.13E-10 |
| GO Process | GO:0006959 | Humoral immune response | 23 | 268 | 0.87 | 1.63 | 2.73E-10 |
| GO Process | GO:0007166 | Cell surface receptor signaling pathway | 63 | 2040 | 0.43 | 0.96 | 2.83E-10 |
| GO Process | GO:0009967 | Positive regulation of signal transduction | 53 | 1525 | 0.48 | 1.03 | 4.33E-10 |
| GO Process | GO:0009617 | Response to bacterium | 34 | 663 | 0.65 | 1.27 | 5.73E-10 |
| GO Process | GO:0022407 | Regulation of cell-cell adhesion | 29 | 480 | 0.72 | 1.37 | 7.03E-10 |
| GO Process | GO:0010033 | Response to organic substance | 73 | 2692 | 0.37 | 0.87 | 9.26E-10 |
| GO Process | GO:0002694 | Regulation of leukocyte activation | 32 | 601 | 0.66 | 1.28 | 9.97E-10 |
| GO Process | GO:0050865 | Regulation of cell activation | 33 | 658 | 0.64 | 1.22 | 1.96E-09 |
| GO Process | GO:0043408 | Regulation of MAPK cascade | 33 | 668 | 0.63 | 1.2 | 2.79E-09 |
| GO Process | GO:0043410 | Positive regulation of MAPK cascade | 28 | 481 | 0.7 | 1.29 | 3.40E-09 |
| GO Process | GO:0050863 | Regulation of T cell activation | 25 | 376 | 0.76 | 1.37 | 3.40E-09 |
| GO Process | GO:0034612 | Response to tumor necrosis factor | 19 | 199 | 0.92 | 1.56 | 3.96E-09 |
| GO Process | GO:0010647 | Positive regulation of cell communication | 54 | 1693 | 0.44 | 0.93 | 4.49E-09 |
| GO Process | GO:0023056 | Positive regulation of signaling | 54 | 1698 | 0.44 | 0.93 | 4.86E-09 |
| GO Process | GO:0006954 | Inflammatory response | 29 | 538 | 0.67 | 1.22 | 7.04E-09 |
| GO Process | GO:0009966 | Regulation of signal transduction | 75 | 2978 | 0.34 | 0.79 | 1.03E-08 |
| GO Process | GO:0071345 | Cellular response to cytokine stimulus | 33 | 711 | 0.6 | 1.12 | 1.07E-08 |
| GO Process | GO:1903037 | Regulation of leukocyte cell-cell adhesion | 24 | 368 | 0.75 | 1.31 | 1.07E-08 |
| GO Process | GO:0032101 | Regulation of response to external stimulus | 38 | 964 | 0.53 | 1.01 | 2.71E-08 |
| GO Process | GO:0071356 | Cellular response to tumor necrosis factor | 17 | 175 | 0.92 | 1.45 | 3.22E-08 |
| GO Process | GO:0002687 | Positive regulation of leukocyte migration | 16 | 151 | 0.96 | 1.49 | 3.52E-08 |
| GO Process | GO:0001906 | Cell killing | 15 | 129 | 1 | 1.51 | 4.11E-08 |
| GO Process | GO:0051249 | Regulation of lymphocyte activation | 27 | 508 | 0.66 | 1.15 | 4.11E-08 |
| GO Process | GO:0010646 | Regulation of cell communication | 79 | 3355 | 0.31 | 0.73 | 5.12E-08 |
| GO Process | GO:0023051 | Regulation of signaling | 79 | 3367 | 0.31 | 0.73 | 5.95E-08 |
| GO Process | GO:0070374 | Positive regulation of ERK1 and ERK2 cascade | 18 | 213 | 0.86 | 1.35 | 6.34E-08 |
| GO Process | GO:1902533 | Positive regulation of intracellular signal transduction | 37 | 997 | 0.51 | 0.92 | 2.10E-07 |
| GO Process | GO:0051239 | Regulation of multicellular organismal process | 68 | 2749 | 0.33 | 0.73 | 2.17E-07 |
| GO Process | GO:0019221 | Cytokine-mediated signaling pathway | 22 | 369 | 0.71 | 1.12 | 2.86E-07 |
| GO Process | GO:0045785 | Positive regulation of cell adhesion | 25 | 485 | 0.65 | 1.05 | 3.30E-07 |
| GO Process | GO:0070663 | Regulation of leukocyte proliferation | 19 | 271 | 0.78 | 1.18 | 3.34E-07 |
| GO Process | GO:0030334 | Regulation of cell migration | 35 | 927 | 0.51 | 0.91 | 3.81E-07 |
| GO Process | GO:1901700 | Response to oxygen-containing compound | 47 | 1547 | 0.42 | 0.81 | 4.01E-07 |
| GO Process | GO:0040011 | Locomotion | 26 | 537 | 0.62 | 1.01 | 4.84E-07 |
| GO Process | GO:0042129 | Regulation of T cell proliferation | 16 | 187 | 0.87 | 1.24 | 4.84E-07 |
| GO Process | GO:0080134 | Regulation of response to stress | 43 | 1373 | 0.43 | 0.8 | 9.21E-07 |
| GO Process | GO:0007165 | Signal transduction | 95 | 4714 | 0.24 | 0.62 | 9.29E-07 |
| GO Process | GO:0006935 | Chemotaxis | 25 | 516 | 0.62 | 0.98 | 9.46E-07 |
| GO Process | GO:0070887 | Cellular response to chemical stimulus | 64 | 2609 | 0.33 | 0.7 | 9.64E-07 |
| GO Process | GO:0002685 | Regulation of leukocyte migration | 17 | 231 | 0.8 | 1.14 | 1.10E-06 |
| GO Process | GO:0070372 | Regulation of ERK1 and ERK2 cascade | 19 | 299 | 0.74 | 1.08 | 1.26E-06 |
| GO Process | GO:0030335 | Positive regulation of cell migration | 25 | 529 | 0.61 | 0.96 | 1.40E-06 |
| GO Process | GO:0019730 | Antimicrobial humoral response | 14 | 149 | 0.91 | 1.2 | 1.45E-06 |
| GO Process | GO:0048518 | Positive regulation of biological process | 114 | 6207 | 0.2 | 0.58 | 1.49E-06 |
| GO Process | GO:0044092 | Negative regulation of molecular function | 38 | 1143 | 0.46 | 0.81 | 1.58E-06 |
| GO Process | GO:0071675 | Regulation of mononuclear cell migration | 13 | 125 | 0.95 | 1.23 | 1.58E-06 |
| GO Process | GO:0034341 | Response to interferon-gamma | 13 | 127 | 0.95 | 1.21 | 1.85E-06 |
| GO Process | GO:0050670 | Regulation of lymphocyte proliferation | 17 | 242 | 0.78 | 1.09 | 1.85E-06 |
| GO Process | GO:0002697 | Regulation of immune effector process | 21 | 383 | 0.68 | 1.00 | 1.96E-06 |
| GO Process | GO:0010466 | Negative regulation of peptidase activity | 17 | 249 | 0.77 | 1.06 | 2.63E-06 |
| GO Process | GO:0050900 | Leukocyte migration | 17 | 249 | 0.77 | 1.06 | 2.63E-06 |
| GO Process | GO:0051346 | Negative regulation of hydrolase activity | 20 | 354 | 0.69 | 1 | 2.66E-06 |
| GO Process | GO:0042127 | Regulation of cell population proliferation | 47 | 1669 | 0.39 | 0.73 | 2.89E-06 |
| GO Process | GO:0022409 | Positive regulation of cell-cell adhesion | 19 | 322 | 0.71 | 1 | 3.11E-06 |
| GO Process | GO:0007154 | Cell communication | 99 | 5165 | 0.22 | 0.58 | 3.58E-06 |
| GO Process | GO:0050793 | Regulation of developmental process | 60 | 2492 | 0.32 | 0.65 | 5.04E-06 |
| GO Process | GO:0023052 | Signaling | 97 | 5057 | 0.22 | 0.57 | 5.28E-06 |
| GO Process | GO:0032103 | Positive regulation of response to external stimulus | 22 | 453 | 0.62 | 0.9 | 5.77E-06 |
| GO Process | GO:0071310 | Cellular response to organic substance | 52 | 2019 | 0.35 | 0.67 | 6.71E-06 |
| GO Process | GO:0048519 | Negative regulation of biological process | 100 | 5313 | 0.21 | 0.56 | 6.98E-06 |
| GO Process | GO:0051094 | Positive regulation of developmental process | 40 | 1332 | 0.41 | 0.73 | 7.09E-06 |
| GO Process | GO:0002696 | Positive regulation of leukocyte activation | 20 | 384 | 0.65 | 0.91 | 8.09E-06 |
| GO Process | GO:0010951 | Negative regulation of endopeptidase activity | 16 | 240 | 0.76 | 0.99 | 8.09E-06 |
| GO Process | GO:0071677 | Positive regulation of mononuclear cell migration | 10 | 75 | 1.06 | 1.17 | 8.09E-06 |
| GO Process | GO:1903039 | Positive regulation of leukocyte cell-cell adhesion | 17 | 274 | 0.73 | 0.97 | 8.09E-06 |
| GO Process | GO:0002822 | Regulation of adaptive immune response based on somatic recombination of immune receptors built from immunoglobulin superfamily domains | 14 | 179 | 0.83 | 1.03 | 8.71E-06 |
| GO Process | GO:0051716 | Cellular response to stimulus | 113 | 6357 | 0.19 | 0.54 | 9.42E-06 |
| GO Process | GO:0032496 | Response to lipopolysaccharide | 18 | 314 | 0.69 | 0.93 | 9.48E-06 |
| GO Process | GO:0048522 | Positive regulation of cellular process | 103 | 5584 | 0.2 | 0.55 | 9.64E-06 |
| GO Process | GO:0002683 | Negative regulation of immune system process | 21 | 433 | 0.62 | 0.87 | 1.08E-05 |
| GO Process | GO:0050870 | Positive regulation of T cell activation | 16 | 251 | 0.74 | 0.95 | 1.31E-05 |
| GO Process | GO:0007162 | Negative regulation of cell adhesion | 17 | 289 | 0.71 | 0.92 | 1.50E-05 |
| GO Process | GO:0071346 | Cellular response to interferon-gamma | 11 | 105 | 0.96 | 1.07 | 1.50E-05 |
| GO Process | GO:1902531 | Regulation of intracellular signal transduction | 46 | 1726 | 0.36 | 0.66 | 1.60E-05 |
| GO Process | GO:0002548 | Monocyte chemotaxis | 8 | 43 | 1.21 | 1.17 | 1.67E-05 |
| GO Process | GO:0022408 | Negative regulation of cell-cell adhesion | 14 | 194 | 0.79 | 0.96 | 1.98E-05 |
| GO Process | GO:0002252 | Immune effector process | 19 | 375 | 0.64 | 0.85 | 2.24E-05 |
| GO Process | GO:0097529 | Myeloid leukocyte migration | 12 | 138 | 0.88 | 0.99 | 2.38E-05 |
| GO Process | GO:0033993 | Response to lipid | 29 | 827 | 0.48 | 0.73 | 2.54E-05 |
| GO Process | GO:1903038 | Negative regulation of leukocyte cell-cell adhesion | 12 | 140 | 0.87 | 0.98 | 2.70E-05 |
| GO Process | GO:0042742 | Defense response to bacterium | 17 | 306 | 0.68 | 0.86 | 2.92E-05 |
| GO Process | GO:0071674 | Mononuclear cell migration | 11 | 116 | 0.91 | 0.98 | 3.39E-05 |
| GO Process | GO:0002703 | Regulation of leukocyte mediated immunity | 15 | 239 | 0.73 | 0.88 | 3.53E-05 |
| GO Process | GO:0050776 | Regulation of immune response | 29 | 844 | 0.47 | 0.71 | 3.66E-05 |
| GO Process | GO:0001934 | Positive regulation of protein phosphorylation | 27 | 747 | 0.49 | 0.73 | 3.75E-05 |
| GO Process | GO:0002706 | Regulation of lymphocyte mediated immunity | 13 | 176 | 0.8 | 0.92 | 3.91E-05 |
| GO Process | GO:0016477 | Cell migration | 30 | 903 | 0.46 | 0.7 | 4.35E-05 |
| GO Process | GO:0031640 | Killing of cells of another organism | 9 | 71 | 1.04 | 1.02 | 4.37E-05 |
| GO Process | GO:2000026 | Regulation of multicellular organismal development | 39 | 1389 | 0.38 | 0.64 | 4.37E-05 |
| GO Process | GO:0002443 | Leukocyte mediated immunity | 14 | 211 | 0.76 | 0.88 | 4.48E-05 |
| GO Process | GO:0030595 | Leukocyte chemotaxis | 12 | 149 | 0.84 | 0.93 | 4.56E-05 |
| GO Process | GO:0048247 | Lymphocyte chemotaxis | 8 | 51 | 1.13 | 1.05 | 4.56E-05 |
| GO Process | GO:0051336 | Regulation of hydrolase activity | 32 | 1011 | 0.44 | 0.68 | 4.57E-05 |
| GO Process | GO:0002250 | Adaptive immune response | 18 | 359 | 0.64 | 0.81 | 4.80E-05 |
| GO Process | GO:1902107 | Positive regulation of leukocyte differentiation | 13 | 181 | 0.79 | 0.9 | 4.90E-05 |
| GO Process | GO:0045321 | Leukocyte activation | 23 | 574 | 0.54 | 0.74 | 5.05E-05 |
| GO Process | GO:0052547 | Regulation of peptidase activity | 20 | 446 | 0.59 | 0.77 | 5.60E-05 |
| GO Process | GO:0045595 | Regulation of cell differentiation | 42 | 1582 | 0.36 | 0.62 | 5.64E-05 |
| GO Process | GO:0070555 | Response to interleukin-1 | 11 | 125 | 0.88 | 0.93 | 5.84E-05 |
| GO Process | GO:0043086 | Negative regulation of catalytic activity | 27 | 771 | 0.48 | 0.7 | 5.98E-05 |
| GO Process | GO:0045597 | Positive regulation of cell differentiation | 29 | 876 | 0.46 | 0.68 | 6.62E-05 |
| GO Process | GO:0052548 | Regulation of endopeptidase activity | 19 | 414 | 0.6 | 0.76 | 7.59E-05 |
| GO Process | GO:0051251 | Positive regulation of lymphocyte activation | 17 | 336 | 0.64 | 0.78 | 8.39E-05 |
| GO Process | GO:0065009 | Regulation of molecular function | 65 | 3085 | 0.26 | 0.54 | 9.10E-05 |
| GO Process | GO:0008284 | Positive regulation of cell population proliferation | 30 | 945 | 0.44 | 0.65 | 9.30E-05 |
| GO Process | GO:1903034 | Regulation of response to wounding | 12 | 162 | 0.81 | 0.86 | 9.30E-05 |
| GO Process | GO:0072676 | Lymphocyte migration | 9 | 80 | 0.99 | 0.94 | 9.37E-05 |
| GO Process | GO:0051240 | Positive regulation of multicellular organismal process | 40 | 1505 | 0.36 | 0.6 | 9.99E-05 |
| GO Process | GO:0046718 | Viral entry into host cell | 10 | 107 | 0.91 | 0.89 | 1.10E-04 |
| GO Process | GO:0070098 | Chemokine-mediated signaling pathway | 9 | 82 | 0.98 | 0.92 | 1.10E-04 |
| GO Process | GO:0045937 | Positive regulation of phosphate metabolic process | 29 | 912 | 0.44 | 0.64 | 1.30E-04 |
| GO Process | GO:0009725 | Response to hormone | 26 | 762 | 0.47 | 0.65 | 1.40E-04 |
| GO Process | GO:1901652 | Response to peptide | 19 | 437 | 0.57 | 0.71 | 1.50E-04 |
| GO Process | GO:0061844 | Antimicrobial humoral immune response mediated by antimicrobial peptide | 10 | 113 | 0.88 | 0.85 | 1.60E-04 |
| GO Process | GO:0051241 | Negative regulation of multicellular organismal process | 31 | 1035 | 0.41 | 0.61 | 1.80E-04 |
| GO Process | GO:0048585 | Negative regulation of response to stimulus | 41 | 1612 | 0.34 | 0.56 | 2.00E-04 |
| GO Process | GO:0060326 | Cell chemotaxis | 13 | 210 | 0.73 | 0.77 | 2.00E-04 |
| GO Process | GO:0050921 | Positive regulation of chemotaxis | 11 | 147 | 0.81 | 0.8 | 2.20E-04 |
| GO Process | GO:0031347 | Regulation of defense response | 23 | 638 | 0.49 | 0.64 | 2.30E-04 |
| GO Process | GO:0098609 | Cell-cell adhesion | 21 | 542 | 0.52 | 0.66 | 2.30E-04 |
| GO Process | GO:2000272 | Negative regulation of signaling receptor activity | 8 | 67 | 1.01 | 0.87 | 2.30E-04 |
| GO Process | GO:0001817 | Regulation of cytokine production | 25 | 739 | 0.47 | 0.63 | 2.40E-04 |
| GO Process | GO:0001932 | Regulation of protein phosphorylation | 32 | 1108 | 0.4 | 0.59 | 2.40E-04 |
| GO Process | GO:0001775 | Cell activation | 24 | 693 | 0.48 | 0.63 | 2.60E-04 |
| GO Process | GO:0048870 | Cell motility | 31 | 1061 | 0.4 | 0.59 | 2.70E-04 |
| GO Process | GO:2000106 | Regulation of leukocyte apoptotic process | 9 | 95 | 0.91 | 0.81 | 3.00E-04 |
| GO Process | GO:0002690 | Positive regulation of leukocyte chemotaxis | 9 | 99 | 0.9 | 0.78 | 4.00E-04 |
| GO Process | GO:0002449 | Lymphocyte mediated immunity | 11 | 159 | 0.78 | 0.74 | 4.10E-04 |
| GO Process | GO:0050790 | Regulation of catalytic activity | 52 | 2370 | 0.28 | 0.5 | 4.40E-04 |
| GO Process | GO:0050868 | Negative regulation of T cell activation | 10 | 129 | 0.83 | 0.75 | 4.40E-04 |
| GO Process | GO:0042130 | Negative regulation of T cell proliferation | 8 | 75 | 0.96 | 0.79 | 4.60E-04 |
| GO Process | GO:0050830 | Defense response to Gram-positive bacterium | 10 | 130 | 0.82 | 0.75 | 4.60E-04 |
| GO Process | GO:0031341 | Regulation of cell killing | 9 | 102 | 0.88 | 0.76 | 4.90E-04 |
| GO Process | GO:0033273 | Response to vitamin | 8 | 78 | 0.95 | 0.77 | 0.001 |
| GO Process | GO:0048523 | Negative regulation of cellular process | 85 | 4736 | 0.19 | 0.44 | 0.001 |
| GO Process | GO:0051896 | Regulation of protein kinase B signaling | 11 | 166 | 0.76 | 0.71 | 0.001 |
| GO Process | GO:1902105 | Regulation of leukocyte differentiation | 15 | 315 | 0.61 | 0.65 | 0.001 |
| GO Process | GO:0030162 | Regulation of proteolysis | 24 | 739 | 0.45 | 0.57 | 0.001 |
| GO Process | GO:0010648 | Negative regulation of cell communication | 35 | 1348 | 0.35 | 0.52 | 0.001 |
| GO Process | GO:0023057 | Negative regulation of signaling | 35 | 1354 | 0.35 | 0.52 | 0.001 |
| GO Process | GO:0009719 | Response to endogenous stimulus | 35 | 1363 | 0.35 | 0.51 | 0.001 |
| GO Process | GO:0051897 | Positive regulation of protein kinase B signaling | 9 | 113 | 0.84 | 0.69 | 0.001 |
| GO Process | GO:0032720 | Negative regulation of tumor necrosis factor production | 7 | 61 | 1 | 0.72 | 0.001 |
| GO Process | GO:0007584 | Response to nutrient | 10 | 147 | 0.77 | 0.66 | 0.001 |
| GO Process | GO:0045582 | Positive regulation of T cell differentiation | 9 | 116 | 0.83 | 0.67 | 0.001 |
| GO Process | GO:0048731 | System development | 72 | 3867 | 0.21 | 0.43 | 0.001 |
| GO Process | GO:0050789 | Regulation of biological process | 166 | 11655 | 0.09 | 0.37 | 0.001 |
| GO Process | GO:0050866 | Negative regulation of cell activation | 12 | 218 | 0.68 | 0.63 | 0.001 |
| GO Process | GO:0035295 | Tube development | 26 | 880 | 0.41 | 0.52 | 0.001 |
| GO Process | GO:0051246 | Regulation of protein metabolic process | 54 | 2622 | 0.25 | 0.44 | 0.002 |
| GO Process | GO:1990266 | Neutrophil migration | 8 | 92 | 0.88 | 0.66 | 0.002 |
| GO Process | GO:2000403 | Positive regulation of lymphocyte migration | 6 | 43 | 1.08 | 0.7 | 0.002 |
| GO Process | GO:0002698 | Negative regulation of immune effector process | 9 | 123 | 0.8 | 0.64 | 0.002 |
| GO Process | GO:0050920 | Regulation of chemotaxis | 12 | 227 | 0.66 | 0.6 | 0.002 |
| GO Process | GO:1901224 | Positive regulation of NIK/NF-kappaB signaling | 7 | 67 | 0.96 | 0.67 | 0.002 |
| GO Process | GO:0031401 | Positive regulation of protein modification process | 28 | 1018 | 0.38 | 0.48 | 0.002 |
| GO Process | GO:0043434 | Response to peptide hormone | 15 | 355 | 0.56 | 0.55 | 0.002 |
| GO Process | GO:0045471 | Response to ethanol | 9 | 126 | 0.79 | 0.62 | 0.002 |
| GO Process | GO:0046006 | Regulation of activated T cell proliferation | 6 | 45 | 1.06 | 0.68 | 0.002 |
| GO Process | GO:2000401 | Regulation of lymphocyte migration | 7 | 69 | 0.94 | 0.66 | 0.002 |
| GO Process | GO:0002695 | Negative regulation of leukocyte activation | 11 | 196 | 0.69 | 0.59 | 0.002 |
| GO Process | GO:1905523 | Positive regulation of macrophage migration | 5 | 27 | 1.2 | 0.69 | 0.002 |
| GO Process | GO:0071347 | Cellular response to interleukin-1 | 8 | 98 | 0.85 | 0.62 | 0.002 |
| GO Process | GO:0050778 | Positive regulation of immune response | 18 | 502 | 0.49 | 0.52 | 0.002 |
| GO Process | GO:0061041 | Regulation of wound healing | 9 | 130 | 0.78 | 0.6 | 0.002 |
| GO Process | GO:0045662 | Negative regulation of myoblast differentiation | 5 | 28 | 1.19 | 0.67 | 0.003 |
| GO Process | GO:0070848 | Response to growth factor | 18 | 503 | 0.49 | 0.51 | 0.003 |
| GO Process | GO:2000404 | Regulation of T cell migration | 6 | 49 | 1.02 | 0.64 | 0.003 |
| GO Process | GO:0008285 | Negative regulation of cell population proliferation | 22 | 713 | 0.43 | 0.48 | 0.003 |
| GO Process | GO:0000302 | Response to reactive oxygen species | 10 | 169 | 0.71 | 0.57 | 0.003 |
| GO Process | GO:0002460 | Adaptive immune response based on somatic recombination of immune receptors built from immunoglobulin superfamily domains | 10 | 169 | 0.71 | 0.57 | 0.003 |
| GO Process | GO:0034103 | Regulation of tissue remodeling | 7 | 75 | 0.91 | 0.61 | 0.003 |
| GO Process | GO:0050727 | Regulation of inflammatory response | 15 | 371 | 0.54 | 0.52 | 0.003 |
| GO Process | GO:0071219 | Cellular response to molecule of bacterial origin | 11 | 205 | 0.67 | 0.56 | 0.003 |
| GO Process | GO:0019220 | Regulation of phosphate metabolic process | 34 | 1405 | 0.32 | 0.44 | 0.003 |
| GO Process | GO:1901136 | Carbohydrate derivative catabolic process | 10 | 170 | 0.71 | 0.56 | 0.003 |
| GO Process | GO:0046638 | Positive regulation of alpha-beta T cell differentiation | 6 | 51 | 1.01 | 0.62 | 0.003 |
| GO Process | GO:1901701 | Cellular response to oxygen-containing compound | 28 | 1057 | 0.36 | 0.45 | 0.003 |
| GO Process | GO:0080184 | Response to phenylpropanoid | 3 | 4 | 1.81 | 0.68 | 0.004 |
| GO Process | GO:0071363 | Cellular response to growth factor stimulus | 17 | 473 | 0.49 | 0.49 | 0.004 |
| GO Process | GO:0097305 | Response to alcohol | 12 | 252 | 0.61 | 0.52 | 0.004 |
| GO Process | GO:0014068 | Positive regulation of phosphatidylinositol 3-kinase signaling | 7 | 79 | 0.88 | 0.58 | 0.004 |
| GO Process | GO:0042102 | Positive regulation of T cell proliferation | 8 | 108 | 0.81 | 0.57 | 0.004 |
| GO Process | GO:0046007 | Negative regulation of activated T cell proliferation | 4 | 15 | 1.36 | 0.64 | 0.004 |
| GO Process | GO:0006026 | Aminoglycan catabolic process | 5 | 32 | 1.13 | 0.61 | 0.004 |
| GO Process | GO:0002526 | Acute inflammatory response | 7 | 80 | 0.88 | 0.58 | 0.004 |
| GO Process | GO:0030593 | Neutrophil chemotaxis | 7 | 80 | 0.88 | 0.58 | 0.004 |
| GO Process | GO:0045580 | Regulation of T cell differentiation | 10 | 178 | 0.69 | 0.54 | 0.004 |
| GO Process | GO:0002824 | Positive regulation of adaptive immune response based on somatic recombination of immune receptors built from immunoglobulin superfamily domains | 8 | 110 | 0.8 | 0.56 | 0.004 |
| GO Process | GO:0009968 | Negative regulation of signal transduction | 31 | 1252 | 0.33 | 0.43 | 0.004 |
| GO Process | GO:0010035 | Response to inorganic substance | 18 | 532 | 0.47 | 0.47 | 0.004 |
| GO Process | GO:0043372 | Positive regulation of CD4-positive, alpha-beta T cell differentiation | 5 | 33 | 1.12 | 0.6 | 0.005 |
| GO Process | GO:0048678 | Response to axon injury | 6 | 56 | 0.97 | 0.58 | 0.005 |
| GO Process | GO:2000107 | Negative regulation of leukocyte apoptotic process | 6 | 56 | 0.97 | 0.58 | 0.005 |
| GO Process | GO:0002699 | Positive regulation of immune effector process | 12 | 264 | 0.59 | 0.49 | 0.005 |
| GO Process | GO:0009611 | Response to wounding | 16 | 444 | 0.49 | 0.47 | 0.005 |
| GO Process | GO:0009991 | Response to extracellular stimulus | 17 | 492 | 0.47 | 0.46 | 0.005 |
| GO Process | GO:0002719 | Negative regulation of cytokine production involved in immune response | 5 | 35 | 1.09 | 0.58 | 0.006 |
| GO Process | GO:2000406 | Positive regulation of T cell migration | 5 | 35 | 1.09 | 0.58 | 0.006 |
| GO Process | GO:0051248 | Negative regulation of protein metabolic process | 27 | 1038 | 0.35 | 0.42 | 0.006 |
| GO Process | GO:0010941 | Regulation of cell death | 37 | 1651 | 0.29 | 0.4 | 0.006 |
| GO Process | GO:0001910 | Regulation of leukocyte mediated cytotoxicity | 7 | 86 | 0.85 | 0.54 | 0.006 |
| GO Process | GO:0002709 | Regulation of T cell mediated immunity | 7 | 86 | 0.85 | 0.54 | 0.006 |
| GO Process | GO:0071396 | Cellular response to lipid | 17 | 502 | 0.47 | 0.45 | 0.007 |
| GO Process | GO:0006956 | Complement activation | 6 | 60 | 0.94 | 0.54 | 0.007 |
| GO Process | GO:0032649 | Regulation of interferon-gamma production | 8 | 120 | 0.76 | 0.51 | 0.007 |
| GO Process | GO:0046649 | Lymphocyte activation | 16 | 457 | 0.48 | 0.44 | 0.007 |
| GO Process | GO:2001188 | Regulation of T cell activation via T cell receptor contact with antigen bound to MHC molecule on antigen presenting cell | 3 | 6 | 1.64 | 0.59 | 0.007 |
| GO Process | GO:0010759 | Positive regulation of macrophage chemotaxis | 4 | 19 | 1.26 | 0.56 | 0.008 |
| GO Process | GO:0031667 | Response to nutrient levels | 16 | 461 | 0.48 | 0.44 | 0.008 |
| GO Process | GO:0007275 | Multicellular organism development | 73 | 4209 | 0.18 | 0.35 | 0.008 |
| GO Process | GO:0071222 | Cellular response to lipopolysaccharide | 10 | 195 | 0.65 | 0.48 | 0.008 |
| GO Process | GO:0060548 | Negative regulation of cell death | 26 | 1016 | 0.34 | 0.39 | 0.010 |
| GO Process | GO:0001818 | Negative regulation of cytokine production | 12 | 284 | 0.56 | 0.44 | 0.010 |
| GO Process | GO:0007267 | Cell-cell signaling | 27 | 1079 | 0.33 | 0.38 | 0.010 |
| GO Process | GO:0010628 | Positive regulation of gene expression | 28 | 1146 | 0.32 | 0.38 | 0.011 |
| GO Process | GO:0006898 | Receptor-mediated endocytosis | 9 | 166 | 0.67 | 0.45 | 0.011 |
| GO Process | GO:0031960 | Response to corticosteroid | 9 | 167 | 0.67 | 0.45 | 0.012 |
| GO Process | GO:0070665 | Positive regulation of leukocyte proliferation | 9 | 167 | 0.67 | 0.45 | 0.012 |
| GO Process | GO:0002704 | Negative regulation of leukocyte mediated immunity | 6 | 68 | 0.88 | 0.48 | 0.012 |
| GO Process | GO:0042981 | Regulation of apoptotic process | 33 | 1462 | 0.29 | 0.36 | 0.012 |
| GO Process | GO:0048856 | Anatomical structure development | 84 | 5117 | 0.15 | 0.32 | 0.012 |
| GO Process | GO:0070232 | Regulation of T cell apoptotic process | 5 | 43 | 1 | 0.49 | 0.012 |
| GO Process | GO:0032102 | Negative regulation of response to external stimulus | 14 | 387 | 0.49 | 0.41 | 0.013 |
| GO Process | GO:0042592 | Homeostatic process | 32 | 1406 | 0.29 | 0.36 | 0.013 |
| GO Process | GO:2000427 | Positive regulation of apoptotic cell clearance | 3 | 8 | 1.51 | 0.52 | 0.013 |
| GO Process | GO:0031349 | Positive regulation of defense response | 12 | 296 | 0.54 | 0.42 | 0.013 |
| GO Process | GO:0097242 | Amyloid-beta clearance | 4 | 23 | 1.18 | 0.5 | 0.013 |
| GO Process | GO:0042110 | T cell activation | 12 | 298 | 0.54 | 0.41 | 0.014 |
| GO Process | GO:0044403 | Biological process involved in symbiotic interaction | 11 | 257 | 0.57 | 0.41 | 0.015 |
| GO Process | GO:0002705 | Positive regulation of leukocyte mediated immunity | 8 | 138 | 0.7 | 0.43 | 0.015 |
| GO Process | GO:0046849 | Bone remodeling | 5 | 46 | 0.97 | 0.46 | 0.016 |
| GO Process | GO:0010469 | Regulation of signaling receptor activity | 9 | 179 | 0.64 | 0.41 | 0.017 |
| GO Process | GO:0001960 | Negative regulation of cytokine-mediated signaling pathway | 6 | 75 | 0.84 | 0.44 | 0.018 |
| GO Process | GO:0032502 | Developmental process | 90 | 5657 | 0.14 | 0.3 | 0.018 |
| GO Process | GO:0051384 | Response to glucocorticoid | 8 | 144 | 0.68 | 0.41 | 0.020 |
| GO Process | GO:0098742 | Cell-cell adhesion via plasma-membrane adhesion molecules | 11 | 267 | 0.55 | 0.39 | 0.020 |
| GO Process | GO:0045661 | Regulation of myoblast differentiation | 6 | 77 | 0.83 | 0.42 | 0.020 |
| GO Process | GO:0065007 | Biological regulation | 168 | 12385 | 0.07 | 0.28 | 0.020 |
| GO Process | GO:0033197 | Response to vitamin E | 3 | 10 | 1.41 | 0.46 | 0.020 |
| GO Process | GO:0070099 | Regulation of chemokine-mediated signaling pathway | 3 | 10 | 1.41 | 0.46 | 0.020 |
| GO Process | GO:0002831 | Regulation of response to biotic stimulus | 13 | 361 | 0.49 | 0.37 | 0.020 |
| GO Process | GO:0032729 | Positive regulation of interferon-gamma production | 6 | 78 | 0.82 | 0.42 | 0.021 |
| GO Process | GO:0048513 | Animal organ development | 58 | 3246 | 0.19 | 0.31 | 0.021 |
| GO Process | GO:0002675 | Positive regulation of acute inflammatory response | 4 | 27 | 1.11 | 0.44 | 0.021 |
| GO Process | GO:0046850 | Regulation of bone remodeling | 5 | 50 | 0.94 | 0.43 | 0.021 |
| GO Process | GO:0050777 | Negative regulation of immune response | 9 | 185 | 0.62 | 0.39 | 0.021 |
| GO Process | GO:0009636 | Response to toxic substance | 10 | 229 | 0.58 | 0.38 | 0.023 |
| GO Process | GO:0045860 | Positive regulation of protein kinase activity | 14 | 416 | 0.46 | 0.36 | 0.023 |
| GO Process | GO:0097191 | Extrinsic apoptotic signaling pathway | 7 | 113 | 0.73 | 0.4 | 0.023 |
| GO Process | GO:0002700 | Regulation of production of molecular mediator of immune response | 9 | 189 | 0.61 | 0.38 | 0.024 |
| GO Process | GO:0002274 | Myeloid leukocyte activation | 8 | 150 | 0.66 | 0.39 | 0.024 |
| GO Process | GO:0071622 | Regulation of granulocyte chemotaxis | 5 | 52 | 0.92 | 0.41 | 0.024 |
| GO Process | GO:0045596 | Negative regulation of cell differentiation | 19 | 685 | 0.38 | 0.34 | 0.024 |
| GO Process | GO:0051050 | Positive regulation of transport | 23 | 915 | 0.34 | 0.33 | 0.024 |
| GO Process | GO:0030198 | Extracellular matrix organization | 11 | 278 | 0.53 | 0.36 | 0.025 |
| GO Process | GO:0001525 | Angiogenesis | 12 | 325 | 0.5 | 0.36 | 0.026 |
| GO Process | GO:0014070 | Response to organic cyclic compound | 22 | 861 | 0.34 | 0.33 | 0.026 |
| GO Process | GO:0002708 | Positive regulation of lymphocyte mediated immunity | 7 | 116 | 0.72 | 0.39 | 0.026 |
| GO Process | GO:0030154 | Cell differentiation | 61 | 3507 | 0.18 | 0.29 | 0.027 |
| GO Process | GO:0007157 | Heterophilic cell-cell adhesion via plasma membrane cell adhesion molecules | 5 | 54 | 0.9 | 0.4 | 0.027 |
| GO Process | GO:0031099 | Regeneration | 8 | 154 | 0.65 | 0.37 | 0.027 |
| GO Process | GO:0050731 | Positive regulation of peptidyl-tyrosine phosphorylation | 9 | 194 | 0.6 | 0.37 | 0.027 |
| GO Process | GO:0007167 | Enzyme-linked receptor protein signaling pathway | 18 | 641 | 0.38 | 0.33 | 0.029 |
| GO Process | GO:0046883 | Regulation of hormone secretion | 10 | 239 | 0.56 | 0.36 | 0.029 |
| GO Process | GO:0001819 | Positive regulation of cytokine production | 15 | 482 | 0.43 | 0.33 | 0.030 |
| GO Process | GO:0031399 | Regulation of protein modification process | 33 | 1560 | 0.26 | 0.3 | 0.030 |
| GO Process | GO:0051093 | Negative regulation of developmental process | 23 | 933 | 0.33 | 0.31 | 0.030 |
| GO Process | GO:0002718 | Regulation of cytokine production involved in immune response | 7 | 120 | 0.7 | 0.37 | 0.030 |
| GO Process | GO:0001909 | Leukocyte mediated cytotoxicity | 5 | 56 | 0.89 | 0.39 | 0.030 |
| GO Process | GO:0002707 | Negative regulation of lymphocyte mediated immunity | 5 | 56 | 0.89 | 0.39 | 0.030 |
| GO Process | GO:0002825 | Regulation of T-helper 1 type immune response | 4 | 31 | 1.05 | 0.4 | 0.030 |
| GO Process | GO:0033209 | Tumor necrosis factor-mediated signaling pathway | 5 | 56 | 0.89 | 0.39 | 0.030 |
| GO Process | GO:0048246 | Macrophage chemotaxis | 3 | 13 | 1.3 | 0.4 | 0.034 |
| GO Process | GO:2000668 | Regulation of dendritic cell apoptotic process | 3 | 13 | 1.3 | 0.4 | 0.034 |
| GO Process | GO:0032147 | Activation of protein kinase activity | 9 | 203 | 0.58 | 0.34 | 0.035 |
| GO Process | GO:0051247 | Positive regulation of protein metabolic process | 32 | 1512 | 0.26 | 0.29 | 0.035 |
| GO Process | GO:2000521 | Negative regulation of immunological synapse formation | 2 | 2 | 1.94 | 0.41 | 0.035 |
| GO Process | GO:0045926 | Negative regulation of growth | 10 | 247 | 0.54 | 0.34 | 0.035 |
| GO Process | GO:0033674 | Positive regulation of kinase activity | 15 | 494 | 0.42 | 0.32 | 0.036 |
| GO Process | GO:0042267 | Natural killer cell mediated cytotoxicity | 4 | 33 | 1.02 | 0.38 | 0.036 |
| GO Process | GO:0045428 | Regulation of nitric oxide biosynthetic process | 5 | 59 | 0.86 | 0.37 | 0.037 |
| GO Process | GO:0032680 | Regulation of tumor necrosis factor production | 8 | 164 | 0.62 | 0.34 | 0.037 |
| GO Process | GO:0051048 | Negative regulation of secretion | 8 | 164 | 0.62 | 0.34 | 0.037 |
| GO Process | GO:0006022 | Aminoglycan metabolic process | 7 | 126 | 0.68 | 0.35 | 0.038 |
| GO Process | GO:0045859 | Regulation of protein kinase activity | 18 | 663 | 0.37 | 0.3 | 0.039 |
| GO Process | GO:0043549 | Regulation of kinase activity | 20 | 778 | 0.35 | 0.3 | 0.039 |
| GO Process | GO:0090303 | Positive regulation of wound healing | 5 | 60 | 0.86 | 0.36 | 0.039 |
| GO Process | GO:0006897 | Endocytosis | 14 | 447 | 0.43 | 0.31 | 0.039 |
| GO Process | GO:0032673 | Regulation of interleukin-4 production | 4 | 34 | 1.01 | 0.37 | 0.039 |
| GO Process | GO:0150077 | Regulation of neuroinflammatory response | 4 | 34 | 1.01 | 0.37 | 0.039 |
| GO Process | GO:1905952 | Regulation of lipid localization | 8 | 167 | 0.62 | 0.33 | 0.041 |
| GO Process | GO:0007565 | Female pregnancy | 8 | 170 | 0.61 | 0.32 | 0.045 |
| GO Process | GO:0046348 | Amino sugar catabolic process | 3 | 15 | 1.24 | 0.36 | 0.045 |
| GO Process | GO:0150078 | Positive regulation of neuroinflammatory response | 3 | 15 | 1.24 | 0.36 | 0.045 |
| GO Process | GO:0050794 | Regulation of cellular process | 151 | 11025 | 0.07 | 0.24 | 0.046 |
| GO Process | GO:0038061 | NIK/NF-kappaB signaling | 4 | 36 | 0.98 | 0.35 | 0.047 |
| GO Process | GO:0050730 | Regulation of peptidyl-tyrosine phosphorylation | 10 | 259 | 0.52 | 0.31 | 0.047 |
| GO Process | GO:0032642 | Regulation of chemokine production | 6 | 96 | 0.73 | 0.33 | 0.048 |
| GO Process | GO:2001237 | Negative regulation of extrinsic apoptotic signaling pathway | 6 | 97 | 0.73 | 0.33 | 0.050 |
| GO Function | GO:0005102 | Signaling receptor binding | 59 | 1499 | 0.53 | 1.23 | 4.66E-13 |
| GO Function | GO:0030545 | Signaling receptor regulator activity | 32 | 552 | 0.7 | 1.35 | 4.85E-10 |
| GO Function | GO:0048018 | Receptor ligand activity | 29 | 499 | 0.7 | 1.28 | 4.55E-09 |
| GO Function | GO:0005125 | Cytokine activity | 20 | 234 | 0.87 | 1.44 | 1.42E-08 |
| GO Function | GO:0005126 | Cytokine receptor binding | 21 | 270 | 0.83 | 1.38 | 1.88E-08 |
| GO Function | GO:0030246 | Carbohydrate binding | 18 | 278 | 0.75 | 0.99 | 6.88E-06 |
| GO Function | GO:0098772 | Molecular function regulator activity | 52 | 1960 | 0.36 | 0.68 | 8.61E-06 |
| GO Function | GO:0008009 | Chemokine activity | 9 | 48 | 1.21 | 1.23 | 8.78E-06 |
| GO Function | GO:0042379 | Chemokine receptor binding | 10 | 71 | 1.09 | 1.13 | 1.44E-05 |
| GO Function | GO:0030414 | Peptidase inhibitor activity | 14 | 183 | 0.82 | 0.95 | 2.96E-05 |
| GO Function | GO:0017171 | Serine hydrolase activity | 14 | 198 | 0.79 | 0.87 | 6.75E-05 |
| GO Function | GO:0004857 | Enzyme inhibitor activity | 19 | 396 | 0.62 | 0.75 | 1.20E-04 |
| GO Function | GO:0004866 | Endopeptidase inhibitor activity | 13 | 177 | 0.8 | 0.84 | 1.20E-04 |
| GO Function | GO:0008236 | Serine-type peptidase activity | 13 | 194 | 0.76 | 0.77 | 2.40E-04 |
| GO Function | GO:0005539 | Glycosaminoglycan binding | 14 | 245 | 0.69 | 0.7 | 4.80E-04 |
| GO Function | GO:0038023 | Signaling receptor activity | 39 | 1489 | 0.35 | 0.54 | 4.80E-04 |
| GO Function | GO:0001664 | G protein-coupled receptor binding | 15 | 295 | 0.64 | 0.65 | 0.001 |
| GO Function | GO:0004867 | Serine-type endopeptidase inhibitor activity | 9 | 98 | 0.9 | 0.72 | 0.001 |
| GO Function | GO:0008233 | Peptidase activity | 22 | 617 | 0.49 | 0.57 | 0.001 |
| GO Function | GO:0004252 | Serine-type endopeptidase activity | 11 | 176 | 0.73 | 0.6 | 0.002 |
| GO Function | GO:0004175 | Endopeptidase activity | 17 | 437 | 0.53 | 0.5 | 0.004 |
| GO Function | GO:0048020 | CCR chemokine receptor binding | 6 | 47 | 1.04 | 0.57 | 0.006 |
| GO Function | GO:0016787 | Hydrolase activity | 49 | 2347 | 0.26 | 0.38 | 0.006 |
| GO Function | GO:0001618 | Virus receptor activity | 7 | 76 | 0.9 | 0.52 | 0.008 |
| GO Function | GO:0005164 | Tumor necrosis factor receptor binding | 5 | 32 | 1.13 | 0.52 | 0.010 |
| GO Function | GO:0019955 | Cytokine binding | 9 | 141 | 0.74 | 0.48 | 0.010 |
| GO Function | GO:0038024 | Cargo receptor activity | 7 | 82 | 0.87 | 0.48 | 0.011 |
| GO Function | GO:0005515 | Protein binding | 112 | 7242 | 0.13 | 0.3 | 0.015 |
| GO Function | GO:0043394 | Proteoglycan binding | 5 | 37 | 1.07 | 0.46 | 0.016 |
| GO Function | GO:0008083 | Growth factor activity | 9 | 162 | 0.68 | 0.39 | 0.023 |
| GO Function | GO:0019838 | Growth factor binding | 8 | 127 | 0.74 | 0.4 | 0.023 |
| GO Function | GO:0030547 | Signaling receptor inhibitor activity | 5 | 41 | 1.02 | 0.42 | 0.023 |
| GO Function | GO:0008238 | Exopeptidase activity | 7 | 97 | 0.79 | 0.4 | 0.025 |
| GO Function | GO:0008201 | Heparin binding | 9 | 173 | 0.65 | 0.36 | 0.032 |
| GO Function | GO:0016936 | Galactoside binding | 3 | 9 | 1.46 | 0.39 | 0.037 |
| GO Function | GO:0140375 | Immune receptor activity | 8 | 142 | 0.69 | 0.34 | 0.041 |
| GO Function | GO:0004888 | Transmembrane signaling receptor activity | 29 | 1268 | 0.3 | 0.28 | 0.048 |
| GO Component | GO:0005576 | Extracellular region | 159 | 4175 | 0.52 | 1.64 | 2.30E-51 |
| GO Component | GO:0005615 | Extracellular space | 132 | 3247 | 0.55 | 1.68 | 1.11E-41 |
| GO Component | GO:0009986 | Cell surface | 55 | 894 | 0.73 | 1.96 | 3.51E-21 |
| GO Component | GO:0031982 | Vesicle | 108 | 3957 | 0.37 | 1.03 | 2.32E-17 |
| GO Component | GO:0071944 | Cell periphery | 134 | 6015 | 0.28 | 0.86 | 8.06E-16 |
| GO Component | GO:0070062 | Extracellular exosome | 71 | 2096 | 0.47 | 1.15 | 2.14E-14 |
| GO Component | GO:0030141 | Secretory granule | 44 | 873 | 0.64 | 1.47 | 8.44E-14 |
| GO Component | GO:0062023 | Collagen-containing extracellular matrix | 28 | 407 | 0.77 | 1.57 | 2.73E-11 |
| GO Component | GO:0031012 | Extracellular matrix | 32 | 552 | 0.7 | 1.44 | 3.34E-11 |
| GO Component | GO:0009897 | External side of plasma membrane | 26 | 388 | 0.76 | 1.47 | 2.76E-10 |
| GO Component | GO:0005886 | Plasma membrane | 114 | 5544 | 0.25 | 0.72 | 4.77E-10 |
| GO Component | GO:0005775 | Vacuolar lumen | 18 | 175 | 0.95 | 1.67 | 1.13E-09 |
| GO Component | GO:0098552 | Side of membrane | 30 | 611 | 0.63 | 1.17 | 6.90E-09 |
| GO Component | GO:0031410 | Cytoplasmic vesicle | 66 | 2482 | 0.36 | 0.82 | 7.07E-09 |
| GO Component | GO:0034774 | Secretory granule lumen | 21 | 321 | 0.75 | 1.25 | 4.44E-08 |
| GO Component | GO:0005764 | Lysosome | 31 | 746 | 0.56 | 0.99 | 1.21E-07 |
| GO Component | GO:0031226 | Intrinsic component of plasma membrane | 51 | 1788 | 0.39 | 0.8 | 1.49E-07 |
| GO Component | GO:0031225 | Anchored component of membrane | 14 | 171 | 0.85 | 1.13 | 2.19E-06 |
| GO Component | GO:0043202 | Lysosomal lumen | 11 | 97 | 0.99 | 1.2 | 3.18E-06 |
| GO Component | GO:0042582 | Azurophil granule | 13 | 154 | 0.86 | 1.1 | 4.62E-06 |
| GO Component | GO:0012505 | Endomembrane system | 91 | 4721 | 0.22 | 0.57 | 5.45E-06 |
| GO Component | GO:0031224 | Intrinsic component of membrane | 105 | 5828 | 0.19 | 0.54 | 8.49E-06 |
| GO Component | GO:0005887 | Integral component of plasma membrane | 45 | 1706 | 0.36 | 0.66 | 1.18E-05 |
| GO Component | GO:0035578 | Azurophil granule lumen | 10 | 91 | 0.98 | 1.08 | 1.43E-05 |
| GO Component | GO:0005788 | Endoplasmic reticulum lumen | 17 | 312 | 0.67 | 0.89 | 1.51E-05 |
| GO Component | GO:0045121 | Membrane raft | 16 | 320 | 0.64 | 0.77 | 9.76E-05 |
| GO Component | GO:0072562 | Blood microparticle | 10 | 118 | 0.86 | 0.87 | 1.20E-04 |
| GO Component | GO:0016021 | Integral component of membrane | 97 | 5670 | 0.17 | 0.44 | 3.80E-04 |
| GO Component | GO:0110165 | Cellular anatomical entity | 226 | 18293 | 0.03 | 0.37 | 4.30E-04 |
| GO Component | GO:0031089 | Platelet dense granule lumen | 4 | 14 | 1.39 | 0.71 | 0.002 |
| GO Component | GO:0035580 | Specific granule lumen | 6 | 62 | 0.92 | 0.55 | 0.006 |
| GO Component | GO:0042581 | Specific granule | 9 | 159 | 0.69 | 0.5 | 0.007 |
| GO Component | GO:0043235 | Receptor complex | 15 | 418 | 0.49 | 0.45 | 0.007 |
| GO Component | GO:0016020 | Membrane | 138 | 9523 | 0.1 | 0.32 | 0.007 |
| GO Component | GO:0030667 | Secretory granule membrane | 12 | 308 | 0.53 | 0.41 | 0.014 |
| GO Component | GO:1905370 | Serine-type endopeptidase complex | 4 | 26 | 1.12 | 0.48 | 0.015 |
| GO Component | GO:0098636 | Protein complex involved in cell adhesion | 5 | 52 | 0.92 | 0.43 | 0.019 |
| GO Component | GO:0005614 | Interstitial matrix | 3 | 12 | 1.33 | 0.44 | 0.024 |
| GO Component | GO:1904724 | Tertiary granule lumen | 5 | 55 | 0.9 | 0.41 | 0.024 |
| GO Component | GO:0070820 | Tertiary granule | 8 | 164 | 0.62 | 0.36 | 0.032 |
| GO Component | GO:0098637 | Protein complex involved in cell-matrix adhesion | 3 | 14 | 1.27 | 0.4 | 0.033 |
| GO Component | GO:0046658 | Anchored component of plasma membrane | 5 | 63 | 0.84 | 0.36 | 0.040 |
| GO Component | GO:0005604 | Basement membrane | 6 | 98 | 0.72 | 0.33 | 0.045 |
| GO Component | GO:0090733 | Tenascin complex | 2 | 3 | 1.76 | 0.37 | 0.045 |
| KEGG | hsa04060 | Cytokine-cytokine receptor interaction | 28 | 282 | 0.93 | 2.17 | 8.90E-15 |
| KEGG | hsa04061 | Viral protein interaction with cytokine and cytokine receptor | 13 | 96 | 1.07 | 1.55 | 6.10E-08 |
| KEGG | hsa05323 | Rheumatoid arthritis | 10 | 83 | 1.02 | 1.11 | 1.25E-05 |
| KEGG | hsa04142 | Lysosome | 11 | 125 | 0.88 | 0.96 | 4.04E-05 |
| KEGG | hsa04610 | Complement and coagulation cascades | 8 | 82 | 0.93 | 0.76 | 0.001 |
| KEGG | hsa04064 | NF-kappa B signaling pathway | 8 | 101 | 0.84 | 0.64 | 0.002 |
| KEGG | hsa04974 | Protein digestion and absorption | 8 | 100 | 0.84 | 0.64 | 0.002 |
| KEGG | hsa04514 | Cell adhesion molecules | 8 | 138 | 0.7 | 0.45 | 0.012 |
| KEGG | hsa05133 | Pertussis | 6 | 73 | 0.85 | 0.47 | 0.012 |
| KEGG | hsa05150 | Staphylococcus aureus infection | 6 | 86 | 0.78 | 0.41 | 0.022 |
| KEGG | hsa04145 | Phagosome | 7 | 141 | 0.63 | 0.32 | 0.049 |
| Reactome | HSA-168256 | Immune System | 73 | 1979 | 0.5 | 1.26 | 4.34E-16 |
| Reactome | HSA-6798695 | Neutrophil degranulation | 27 | 476 | 0.69 | 1.19 | 3.20E-08 |
| Reactome | HSA-168249 | Innate Immune System | 39 | 1041 | 0.51 | 0.95 | 9.25E-08 |
| Reactome | HSA-198933 | Immunoregulatory interactions between a Lymphoid and a non-Lymphoid cell | 15 | 130 | 1 | 1.46 | 9.25E-08 |
| Reactome | HSA-6783783 | Interleukin-10 signaling | 9 | 45 | 1.24 | 1.3 | 4.46E-06 |
| Reactome | HSA-1280215 | Cytokine Signaling in Immune system | 25 | 706 | 0.49 | 0.61 | 4.20E-04 |
| Reactome | HSA-1474244 | Extracellular matrix organization | 14 | 300 | 0.61 | 0.5 | 0.005 |
| Reactome | HSA-163125 | Post-translational modification: synthesis of GPI-anchored proteins | 8 | 93 | 0.87 | 0.54 | 0.006 |
| Reactome | HSA-449147 | Signaling by Interleukins | 17 | 453 | 0.51 | 0.45 | 0.008 |
| Reactome | HSA-5668541 | TNFR2 non-canonical NF-kB pathway | 8 | 99 | 0.84 | 0.51 | 0.008 |
| Reactome | HSA-5669034 | TNFs bind their physiological receptors | 5 | 28 | 1.19 | 0.55 | 0.008 |
| Reactome | HSA-8957275 | Post-translational protein phosphorylation | 8 | 107 | 0.81 | 0.48 | 0.010 |
| Reactome | HSA-1280218 | Adaptive Immune System | 22 | 758 | 0.4 | 0.38 | 0.015 |
| Reactome | HSA-216083 | Integrin cell surface interactions | 7 | 85 | 0.85 | 0.46 | 0.015 |
| Reactome | HSA-380108 | Chemokine receptors bind chemokines | 6 | 57 | 0.96 | 0.47 | 0.015 |
| Reactome | HSA-5676594 | TNF receptor superfamily (TNFSF) members mediating non-canonical NF-kB pathway | 4 | 17 | 1.31 | 0.49 | 0.015 |
| Reactome | HSA-375276 | Peptide ligand-binding receptors | 10 | 1941 | 0.65 | 0.41 | 0.017 |
| Reactome | HSA-381426 | Regulation of Insulin-like Growth Factor (IGF) transport and uptake by Insulin-like Growth Factor Binding Proteins (IGFBPs) | 8 | 124 | 0.75 | 0.42 | 0.018 |

| **Supplementary Table 18. Schoenfeld residual tests for the proportional hazards assumption in Cox models of workplace noise exposure and psychiatric disorders.** | | |
| --- | --- | --- |
| **Psychiatric disorders** | **Noise P** | **GLOBAL P** |
| Anxiety disorder | 0.208 | 0.122 |
| MDD | 0.287 | 0.099 |
| Bipolar disorder | 0.684 | 0.154 |
| Schizophrenia | 0.330 | 0.540 |
| Note: Noise P and GLOBAL P represent the Schoenfeld residual test P values for workplace noise exposure and the overall model, respectively. | | |

| **Supplementary Table 19. Sex-specific associations between workplace noise exposure and risk of MDD derived from the interaction model.** | | | | |
| --- | --- | --- | --- | --- |
| **Sex** | **Workplace noise exposure** | **HR (95% CI)** | **P value** | **P interaction** |
| **Female** | <1 year | 1.601 (0.995–2.576) | 0.052 | **0.0013** |
|  | 1–5 years | 1.012 (0.628–1.631) | 0.960 |  |
|  | >5 years | 0.643 (0.389–1.061) | 0.084 |  |
| **Male** | <1 year | 1.107 (0.719–1.706) | 0.644 |  |
|  | 1–5 years | 1.471 (1.011–2.139) | 0.043 |  |
|  | >5 years | 1.653 (1.263–2.164) | <0.001 |  |

| **Supplementary Table 20. Results of the associations between plasma proteins and anxiety disorder (FDR < 0.05).** | | | | |
| --- | --- | --- | --- | --- |
| **Protein** | **UniProt ID** | **HR (95% CI)** | **P.value** | **FDR** |
| ASGR1 | P07306 | 1.185 (1.104-1.272) | 2.57E-06 | 3.57E-04 |
| B2M | P61769 | 1.131 (1.080-1.184) | 1.46E-07 | 5.31E-05 |
| CHGA | P10645 | 1.172 (1.101-1.247) | 7.09E-07 | 1.59E-04 |
| CXADR | P78310 | 1.182 (1.113-1.256) | 6.22E-08 | 3.63E-05 |
| GAST | P01350 | 1.177 (1.102-1.257) | 1.09E-06 | 2.17E-04 |
| GDF15 | Q99988 | 1.204 (1.135-1.277) | 6.48E-10 | 9.45E-07 |
| GRPEL1 | Q9HAV7 | 1.170 (1.097-1.247) | 1.48E-06 | 2.70E-04 |
| IFI30 | P13284 | 1.207 (1.117-1.304) | 1.79E-06 | 2.74E-04 |
| IGFBP4 | P22692 | 1.206 (1.126-1.292) | 1.04E-07 | 4.35E-05 |
| IMMT | Q16891 | 1.163 (1.094-1.236) | 1.11E-06 | 2.17E-04 |
| NCR3LG1 | Q68D85 | 1.194 (1.110-1.285) | 1.94E-06 | 2.83E-04 |
| NECTIN2 | Q92692 | 1.201 (1.127-1.281) | 1.89E-08 | 1.38E-05 |
| OCLN | Q16625 | 1.202 (1.119-1.291) | 5.09E-07 | 1.35E-04 |
| PLAUR | Q03405 | 1.204 (1.123-1.291) | 1.82E-07 | 5.89E-05 |
| RBFOX3 | A6NFN3 | 1.178 (1.101-1.259) | 1.61E-06 | 2.73E-04 |
| RNASE6 | Q93091 | 1.160 (1.096-1.227) | 2.47E-07 | 7.20E-05 |
| TNFRSF10B | O14763 | 1.153 (1.109-1.198) | 6.09E-13 | 1.78E-09 |
| TNFRSF14 | Q92956 | 1.180 (1.105-1.259) | 6.60E-07 | 1.60E-04 |
| VSIG2 | Q96IQ7 | 1.235 (1.154-1.322) | 1.06E-09 | 1.03E-06 |
| WFDC2 | Q14508 | 1.190 (1.108-1.278) | 1.69E-06 | 2.73E-04 |
| WFIKKN1 | Q96NZ8 | 0.823 (0.766-0.884) | 9.78E-08 | 4.35E-05 |
| AREG | P15514 | 1.145 (1.079-1.216) | 9.05E-06 | 0.001 |
| BTN3A2 | P78410 | 1.158 (1.083-1.239) | 1.97E-05 | 0.001 |
| CD274 | Q9NZQ7 | 1.146 (1.078-1.220) | 1.49E-05 | 0.001 |
| CD83 | Q01151 | 1.163 (1.087-1.244) | 1.15E-05 | 0.001 |
| CHCHD10 | Q8WYQ3 | 1.179 (1.098-1.267) | 6.49E-06 | 0.001 |
| DEFB4A_DEFB4B | O15263 | 1.167 (1.088-1.252) | 1.46E-05 | 0.001 |
| ECHDC3 | Q96DC8 | 1.160 (1.084-1.241) | 1.77E-05 | 0.001 |
| FGFR4 | P22455 | 1.171 (1.093-1.254) | 6.34E-06 | 0.001 |
| FSTL3 | O95633 | 1.178 (1.096-1.266) | 9.37E-06 | 0.001 |
| IGSF8 | Q969P0 | 1.162 (1.090-1.239) | 4.46E-06 | 0.001 |
| NPDC1 | Q9NQX5 | 1.152 (1.080-1.229) | 1.64E-05 | 0.001 |
| PI3 | P19957 | 1.158 (1.085-1.236) | 1.07E-05 | 0.001 |
| PLA2G15 | Q8NCC3 | 1.165 (1.089-1.245) | 8.46E-06 | 0.001 |
| PRSS8 | Q16651 | 1.182 (1.098-1.272) | 8.06E-06 | 0.001 |
| PVR | P15151 | 1.153 (1.084-1.227) | 5.60E-06 | 0.001 |
| RNASET2 | O00584 | 1.144 (1.076-1.216) | 1.67E-05 | 0.001 |
| SCARB2 | Q14108 | 1.170 (1.090-1.256) | 1.48E-05 | 0.001 |
| SHISA5 | Q8N114 | 1.145 (1.076-1.218) | 1.94E-05 | 0.001 |
| SMPD1 | P17405 | 1.156 (1.082-1.234) | 1.45E-05 | 0.001 |
| TNFRSF10A | O00220 | 1.162 (1.089-1.241) | 6.66E-06 | 0.001 |
| TNFRSF1A | P19438 | 1.166 (1.086-1.252) | 2.14E-05 | 0.001 |
| B4GALT1 | P15291 | 1.150 (1.077-1.228) | 2.73E-05 | 0.002 |
| CA14 | Q9ULX7 | 0.857 (0.797-0.922) | 3.53E-05 | 0.002 |
| COL18A1 | P39060 | 1.161 (1.081-1.246) | 4.28E-05 | 0.002 |
| CPM | P14384 | 1.162 (1.082-1.248) | 3.98E-05 | 0.002 |
| CSF1 | P09603 | 1.148 (1.074-1.227) | 4.96E-05 | 0.002 |
| EGFR | P00533 | 0.874 (0.819-0.933) | 4.87E-05 | 0.002 |
| ITGAM | P11215 | 0.864 (0.807-0.926) | 3.18E-05 | 0.002 |
| ITGAV | P06756 | 0.863 (0.806-0.925) | 2.83E-05 | 0.002 |
| LETM1 | O95202 | 1.154 (1.079-1.235) | 3.29E-05 | 0.002 |
| LMNB2 | Q03252 | 1.165 (1.083-1.253) | 4.02E-05 | 0.002 |
| NTRK3 | Q16288 | 0.862 (0.804-0.923) | 2.29E-05 | 0.002 |
| PALM2 | Q8IXS6 | 1.177 (1.091-1.269) | 2.43E-05 | 0.002 |
| PIGR | P01833 | 1.156 (1.081-1.237) | 2.49E-05 | 0.002 |
| PSAP | P07602 | 1.134 (1.070-1.203) | 2.58E-05 | 0.002 |
| REN | P00797 | 1.146 (1.074-1.224) | 4.35E-05 | 0.002 |
| RNASE1 | P07998 | 1.142 (1.073-1.215) | 2.62E-05 | 0.002 |
| TFF3 | Q07654 | 1.122 (1.062-1.186) | 4.46E-05 | 0.002 |
| ANGPT2 | O15123 | 1.147 (1.073-1.225) | 5.22E-05 | 0.003 |
| CRELD1 | Q96HD1 | 1.165 (1.081-1.257) | 6.65E-05 | 0.003 |
| MMP7 | P09237 | 1.159 (1.078-1.247) | 6.27E-05 | 0.003 |
| NPC2 | P61916 | 1.151 (1.074-1.234) | 6.71E-05 | 0.003 |
| OSCAR | Q8IYS5 | 1.148 (1.073-1.229) | 6.96E-05 | 0.003 |
| TFF2 | Q03403 | 1.146 (1.072-1.224) | 5.81E-05 | 0.003 |
| THBS2 | P35442 | 1.137 (1.067-1.210) | 6.35E-05 | 0.003 |
| TNFRSF1B | P20333 | 1.115 (1.056-1.176) | 7.60E-05 | 0.003 |
| CD99L2 | Q8TCZ2 | 1.144 (1.069-1.224) | 1.07E-04 | 0.004 |
| COL4A1 | P02462 | 1.139 (1.067-1.216) | 9.58E-05 | 0.004 |
| FABP3 | P05413 | 1.159 (1.076-1.248) | 1.03E-04 | 0.004 |
| FGF23 | Q9GZV9 | 1.150 (1.072-1.233) | 9.14E-05 | 0.004 |
| LGALS9 | O00182 | 1.159 (1.076-1.248) | 9.28E-05 | 0.004 |
| PGA4 | P0DJD7 | 1.159 (1.076-1.248) | 9.19E-05 | 0.004 |
| SORCS2 | Q96PQ0 | 1.146 (1.070-1.227) | 9.51E-05 | 0.004 |
| ATRAID | Q6UW56 | 1.130 (1.061-1.204) | 1.40E-04 | 0.005 |
| BST2 | Q10589 | 1.128 (1.061-1.200) | 1.30E-04 | 0.005 |
| CD276 | Q5ZPR3 | 1.142 (1.067-1.223) | 1.35E-04 | 0.005 |
| EFNA4 | P52798 | 1.144 (1.068-1.225) | 1.29E-04 | 0.005 |
| NHLRC3 | Q5JS37 | 1.161 (1.076-1.253) | 1.25E-04 | 0.005 |
| PENK | P01210 | 1.136 (1.064-1.213) | 1.28E-04 | 0.005 |
| PLIN3 | O60664 | 1.135 (1.064-1.210) | 1.23E-04 | 0.005 |
| SRPX | P78539 | 1.164 (1.077-1.258) | 1.37E-04 | 0.005 |
| FAP | Q12884 | 0.879 (0.822-0.941) | 1.81E-04 | 0.006 |
| ITGBL1 | O95965 | 1.149 (1.068-1.235) | 1.73E-04 | 0.006 |
| LGALS4 | P56470 | 1.140 (1.065-1.221) | 1.60E-04 | 0.006 |
| NT5C1A | Q9BXI3 | 1.129 (1.060-1.203) | 1.84E-04 | 0.006 |
| ADM | P35318 | 1.165 (1.075-1.263) | 2.13E-04 | 0.007 |
| CD80 | P33681 | 1.144 (1.065-1.229) | 2.16E-04 | 0.007 |
| RNASE4 | P34096 | 1.132 (1.060-1.208) | 2.06E-04 | 0.007 |
| ELN | P15502 | 1.186 (1.082-1.301) | 2.66E-04 | 0.008 |
| GUSB | P08236 | 1.145 (1.065-1.231) | 2.56E-04 | 0.008 |
| PRRT3 | Q5FWE3 | 1.140 (1.063-1.224) | 2.69E-04 | 0.008 |
| TNFRSF12A | Q9NP84 | 1.140 (1.063-1.222) | 2.44E-04 | 0.008 |
| ADAMTSL2 | Q86TH1 | 1.143 (1.063-1.230) | 3.24E-04 | 0.009 |
| BMPER | Q8N8U9 | 1.125 (1.055-1.199) | 3.00E-04 | 0.009 |
| CEACAM5 | P06731 | 1.132 (1.058-1.211) | 3.46E-04 | 0.009 |
| COL6A3 | P12111 | 1.140 (1.062-1.223) | 3.01E-04 | 0.009 |
| CRTAC1 | Q9NQ79 | 0.881 (0.823-0.944) | 3.12E-04 | 0.009 |
| CSTB | P04080 | 1.132 (1.058-1.210) | 2.96E-04 | 0.009 |
| EFCAB14 | O75071 | 1.110 (1.049-1.175) | 3.04E-04 | 0.009 |
| FAM3C | Q92520 | 1.136 (1.060-1.217) | 2.96E-04 | 0.009 |
| FSTL1 | Q12841 | 1.131 (1.059-1.209) | 2.78E-04 | 0.009 |
| ITGA11 | Q9UKX5 | 0.884 (0.826-0.945) | 3.17E-04 | 0.009 |
| LGALS1 | P09382 | 1.134 (1.059-1.214) | 3.20E-04 | 0.009 |
| SPINK1 | P00995 | 1.123 (1.054-1.196) | 3.17E-04 | 0.009 |
| SYT1 | P21579 | 1.112 (1.050-1.178) | 3.09E-04 | 0.009 |
| ULBP2 | Q9BZM5 | 1.131 (1.058-1.210) | 3.09E-04 | 0.009 |
| YAP1 | P46937 | 1.152 (1.067-1.245) | 2.99E-04 | 0.009 |
| EDA2R | Q9HAV5 | 1.159 (1.068-1.258) | 3.85E-04 | 0.010 |
| HPGDS | O60760 | 0.883 (0.825-0.946) | 3.69E-04 | 0.010 |
| TGFBR2 | P37173 | 1.131 (1.056-1.210) | 3.99E-04 | 0.010 |
| ADGRG1 | Q9Y653 | 1.125 (1.053-1.201) | 4.57E-04 | 0.011 |
| CEMIP2 | Q9UHN6 | 1.100 (1.043-1.161) | 4.35E-04 | 0.011 |
| CXCL17 | Q6UXB2 | 1.146 (1.062-1.236) | 4.18E-04 | 0.011 |
| ENPP7 | Q6UWV6 | 1.130 (1.056-1.210) | 4.11E-04 | 0.011 |
| HAVCR1 | Q96D42 | 1.137 (1.058-1.222) | 4.49E-04 | 0.011 |
| IL12RB2 | Q99665 | 1.124 (1.053-1.200) | 4.24E-04 | 0.011 |
| LAIR1 | Q6GTX8 | 1.117 (1.050-1.187) | 4.36E-04 | 0.011 |
| SIGLEC8 | Q9NYZ4 | 1.139 (1.059-1.225) | 4.66E-04 | 0.011 |
| ACE2 | Q9BYF1 | 1.136 (1.057-1.220) | 4.95E-04 | 0.012 |
| CA6 | P23280 | 0.883 (0.823-0.947) | 5.07E-04 | 0.012 |
| CTSF | Q9UBX1 | 1.128 (1.054-1.207) | 4.93E-04 | 0.012 |
| DTNB | O60941 | 1.131 (1.055-1.212) | 5.01E-04 | 0.012 |
| CTSL | P07711 | 1.127 (1.053-1.207) | 5.80E-04 | 0.013 |
| SUSD4 | Q5VX71 | 1.120 (1.050-1.194) | 5.72E-04 | 0.013 |
| VSIG4 | Q9Y279 | 1.136 (1.057-1.221) | 5.48E-04 | 0.013 |
| ADAM9 | Q13443 | 1.132 (1.054-1.216) | 6.29E-04 | 0.014 |
| MRC1 | P22897 | 1.136 (1.056-1.221) | 6.23E-04 | 0.014 |
| CHCHD6 | Q9BRQ6 | 1.101 (1.042-1.164) | 6.65E-04 | 0.015 |
| DPY30 | Q9C005 | 1.122 (1.050-1.199) | 6.85E-04 | 0.015 |
| IL1RL1 | Q01638 | 1.133 (1.054-1.218) | 6.97E-04 | 0.015 |
| JAM3 | Q9BX67 | 1.121 (1.050-1.196) | 6.52E-04 | 0.015 |
| MAD1L1 | Q9Y6D9 | 1.114 (1.047-1.186) | 6.91E-04 | 0.015 |
| MERTK | Q12866 | 1.124 (1.051-1.202) | 6.45E-04 | 0.015 |
| NAGPA | Q9UK23 | 1.138 (1.056-1.226) | 7.04E-04 | 0.015 |
| ASAH1 | Q13510 | 1.117 (1.047-1.192) | 7.74E-04 | 0.017 |
| OXCT1 | P55809 | 1.124 (1.050-1.203) | 8.09E-04 | 0.017 |
| TAFA5 | Q7Z5A7 | 1.120 (1.048-1.197) | 8.28E-04 | 0.017 |
| TCOF1 | Q13428 | 1.116 (1.047-1.189) | 7.86E-04 | 0.017 |
| CRIM1 | Q9NZV1 | 1.116 (1.046-1.191) | 8.60E-04 | 0.018 |
| SEPTIN8 | Q92599 | 1.121 (1.048-1.199) | 8.83E-04 | 0.018 |
| AGRN | O00468 | 1.120 (1.047-1.197) | 0.001 | 0.019 |
| GPNMB | Q14956 | 1.124 (1.049-1.204) | 0.001 | 0.019 |
| GUCA2A | Q02747 | 1.120 (1.047-1.198) | 0.001 | 0.019 |
| CD302 | Q8IX05 | 1.122 (1.048-1.202) | 0.001 | 0.020 |
| CHRDL1 | Q9BU40 | 1.134 (1.052-1.223) | 0.001 | 0.021 |
| IGFBP7 | Q16270 | 1.119 (1.046-1.197) | 0.001 | 0.021 |
| PRAP1 | Q96NZ9 | 1.147 (1.057-1.244) | 0.001 | 0.021 |
| PTPRF | P10586 | 0.892 (0.833-0.955) | 0.001 | 0.021 |
| CCER2 | I3L3R5 | 1.121 (1.046-1.202) | 0.001 | 0.022 |
| CCL19 | Q99731 | 1.102 (1.039-1.169) | 0.001 | 0.022 |
| LAMB1 | P07942 | 1.129 (1.049-1.214) | 0.001 | 0.022 |
| PILRB | Q9UKJ0 | 1.125 (1.048-1.207) | 0.001 | 0.022 |
| SLMAP | Q14BN4 | 1.119 (1.046-1.197) | 0.001 | 0.022 |
| SNAP25 | P60880 | 1.127 (1.048-1.211) | 0.001 | 0.022 |
| TPSG1 | Q9NRR2 | 1.106 (1.040-1.175) | 0.001 | 0.022 |
| UBE2L6 | O14933 | 1.126 (1.048-1.209) | 0.001 | 0.022 |
| NCS1 | P62166 | 1.126 (1.047-1.210) | 0.001 | 0.023 |
| COLEC12 | Q5KU26 | 1.131 (1.050-1.220) | 0.001 | 0.024 |
| GBP1 | P32455 | 1.127 (1.048-1.213) | 0.001 | 0.024 |
| LONP1 | P36776 | 1.125 (1.047-1.209) | 0.001 | 0.024 |
| EFNA1 | P20827 | 1.118 (1.044-1.196) | 0.001 | 0.025 |
| FKBP4 | Q02790 | 1.125 (1.046-1.209) | 0.001 | 0.025 |
| DCBLD2 | Q96PD2 | 1.117 (1.043-1.196) | 0.002 | 0.026 |
| IL10RB | Q08334 | 1.109 (1.041-1.183) | 0.001 | 0.026 |
| PTPRS | Q13332 | 0.898 (0.841-0.960) | 0.001 | 0.026 |
| THY1 | P04216 | 1.120 (1.044-1.201) | 0.002 | 0.026 |
| TREM2 | Q9NZC2 | 1.123 (1.046-1.206) | 0.001 | 0.026 |
| ACVRL1 | P37023 | 1.121 (1.045-1.204) | 0.002 | 0.027 |
| CLPP | Q16740 | 1.113 (1.041-1.189) | 0.002 | 0.027 |
| CXCL8 | P10145 | 1.108 (1.040-1.180) | 0.002 | 0.027 |
| GFRA1 | P56159 | 1.122 (1.045-1.206) | 0.002 | 0.027 |
| SPON2 | Q9BUD6 | 1.118 (1.044-1.199) | 0.002 | 0.027 |
| TNFRSF13B | O14836 | 1.105 (1.038-1.176) | 0.002 | 0.027 |
| CD40 | P25942 | 1.109 (1.040-1.182) | 0.002 | 0.028 |
| IL1R1 | P14778 | 1.108 (1.039-1.182) | 0.002 | 0.028 |
| LAYN | Q6UX15 | 1.117 (1.042-1.197) | 0.002 | 0.029 |
| IL15 | P40933 | 1.108 (1.039-1.182) | 0.002 | 0.030 |
| EDN1 | P05305 | 1.122 (1.043-1.207) | 0.002 | 0.031 |
| FN1 | P02751 | 1.130 (1.046-1.220) | 0.002 | 0.031 |
| HS6ST2 | Q96MM7 | 1.122 (1.043-1.207) | 0.002 | 0.031 |
| IL4R | P24394 | 1.107 (1.038-1.180) | 0.002 | 0.031 |
| TIMP1 | P01033 | 1.117 (1.041-1.197) | 0.002 | 0.031 |
| CKAP4 | Q07065 | 1.109 (1.038-1.185) | 0.002 | 0.032 |
| EPHA2 | P29317 | 1.114 (1.040-1.193) | 0.002 | 0.032 |
| FCAMR | Q8WWV6 | 1.128 (1.045-1.218) | 0.002 | 0.032 |
| GALNT10 | Q86SR1 | 1.110 (1.039-1.185) | 0.002 | 0.032 |
| IGFBPL1 | Q8WX77 | 1.115 (1.040-1.196) | 0.002 | 0.032 |
| PIK3IP1 | Q96FE7 | 1.114 (1.040-1.194) | 0.002 | 0.032 |
| RTBDN | Q9BSG5 | 1.109 (1.038-1.184) | 0.002 | 0.032 |
| TNFRSF6B | O95407 | 1.114 (1.040-1.193) | 0.002 | 0.032 |
| TNFSF11 | O14788 | 0.899 (0.840-0.962) | 0.002 | 0.032 |
| LRG1 | P02750 | 1.122 (1.042-1.208) | 0.002 | 0.033 |
| CD4 | P01730 | 1.088 (1.031-1.149) | 0.002 | 0.034 |
| SCARF1 | Q14162 | 1.106 (1.037-1.180) | 0.002 | 0.034 |
| STX8 | Q9UNK0 | 1.101 (1.035-1.171) | 0.002 | 0.035 |
| F11R | Q9Y624 | 1.105 (1.036-1.179) | 0.003 | 0.037 |
| TGFB1 | P01137 | 1.106 (1.036-1.180) | 0.003 | 0.037 |
| CD79B | P40259 | 1.108 (1.036-1.184) | 0.003 | 0.038 |
| FABP4 | P15090 | 1.143 (1.048-1.246) | 0.003 | 0.038 |
| LTBR | P36941 | 1.107 (1.036-1.184) | 0.003 | 0.038 |
| NTproBNP | NTproBNP | 1.114 (1.038-1.194) | 0.003 | 0.038 |
| SIGLEC10 | Q96LC7 | 1.108 (1.036-1.184) | 0.003 | 0.038 |
| HGF | P14210 | 1.119 (1.039-1.204) | 0.003 | 0.039 |
| CES3 | Q6UWW8 | 0.909 (0.854-0.968) | 0.003 | 0.04 |
| CREG1 | O75629 | 1.107 (1.035-1.183) | 0.003 | 0.041 |
| FGFBP3 | Q8TAT2 | 0.898 (0.836-0.964) | 0.003 | 0.041 |
| IL18 | Q14116 | 1.103 (1.034-1.176) | 0.003 | 0.041 |
| ODAM | A1E959 | 0.895 (0.832-0.963) | 0.003 | 0.041 |
| PALLD | Q8WX93 | 1.094 (1.031-1.161) | 0.003 | 0.041 |
| COL5A1 | P20908 | 1.112 (1.037-1.193) | 0.003 | 0.042 |
| GLA | P06280 | 1.120 (1.039-1.208) | 0.003 | 0.042 |
| ITGB2 | P05107 | 0.899 (0.838-0.965) | 0.003 | 0.042 |
| PILRA | Q9UKJ1 | 1.112 (1.036-1.193) | 0.003 | 0.042 |
| APOM | O95445 | 0.903 (0.843-0.966) | 0.003 | 0.043 |
| DDAH1 | O94760 | 1.106 (1.034-1.182) | 0.003 | 0.043 |
| PLSCR3 | Q9NRY6 | 1.110 (1.036-1.190) | 0.003 | 0.043 |
| QSOX1 | O00391 | 1.082 (1.027-1.141) | 0.003 | 0.043 |
| CCL15 | Q16663 | 1.100 (1.032-1.173) | 0.003 | 0.044 |
| CDCP1 | Q9H5V8 | 1.117 (1.038-1.203) | 0.003 | 0.044 |
| CHAD | O15335 | 0.900 (0.838-0.966) | 0.003 | 0.044 |
| ECHS1 | P30084 | 1.117 (1.037-1.202) | 0.003 | 0.044 |
| HAVCR2 | Q8TDQ0 | 1.110 (1.035-1.189) | 0.003 | 0.044 |
| ZNRD2 | O60232 | 1.109 (1.035-1.188) | 0.003 | 0.044 |
| C1QBP | Q07021 | 1.087 (1.028-1.150) | 0.004 | 0.045 |
| C7 | P10643 | 1.117 (1.037-1.203) | 0.003 | 0.045 |
| CTSZ | Q9UBR2 | 1.108 (1.034-1.187) | 0.003 | 0.045 |
| ESYT2 | A0FGR8 | 1.108 (1.034-1.187) | 0.004 | 0.045 |
| CLMP | Q9H6B4 | 1.123 (1.038-1.215) | 0.004 | 0.047 |
| IDUA | P35475 | 1.105 (1.033-1.182) | 0.004 | 0.047 |
| IGF2R | P11717 | 1.107 (1.033-1.185) | 0.004 | 0.047 |
| PLXNB2 | O15031 | 1.104 (1.032-1.180) | 0.004 | 0.047 |
| RAB6A | P20340 | 0.906 (0.848-0.969) | 0.004 | 0.047 |
| TPP1 | O14773 | 1.097 (1.030-1.168) | 0.004 | 0.047 |
| CST3 | P01034 | 1.113 (1.035-1.197) | 0.004 | 4.96E-02 |
| FOLR1 | P15328 | 1.108 (1.033-1.188) | 0.004 | 4.96E-02 |
| MEP1B | Q16820 | 0.909 (0.852-0.970) | 0.004 | 4.98E-02 |
| NPPC | P23582 | 1.117 (1.036-1.204) | 0.004 | 4.96E-02 |
| SCRG1 | O75711 | 1.114 (1.035-1.199) | 0.004 | 4.99E-02 |

| **Supplementary Table 21. Results of the associations between plasma proteins and MDD (FDR < 0.05).** | | | | |
| --- | --- | --- | --- | --- |
| **Protein** | **UniProt ID** | **HR (95% CI)** | **P.value** | **FDR** |
| ADAMTS8 | Q9UP79 | 0.841 (0.781-0.906) | 4.82E-06 | 3.60E-04 |
| AREG | P15514 | 1.179 (1.103-1.262) | 1.54E-06 | 1.54E-04 |
| BST2 | Q10589 | 1.189 (1.115-1.267) | 1.26E-07 | 3.06E-05 |
| BTN3A2 | P78410 | 1.187 (1.102-1.279) | 6.46E-06 | 4.09E-04 |
| CA6 | P23280 | 0.812 (0.750-0.878) | 2.13E-07 | 4.15E-05 |
| CD302 | Q8IX05 | 1.193 (1.108-1.283) | 2.47E-06 | 2.06E-04 |
| CDCP1 | Q9H5V8 | 1.226 (1.134-1.326) | 3.01E-07 | 5.48E-05 |
| CHGA | P10645 | 1.193 (1.113-1.279) | 6.24E-07 | 7.91E-05 |
| CHI3L1 | P36222 | 1.184 (1.099-1.275) | 7.71E-06 | 4.78E-04 |
| CLEC4D | Q8WXI8 | 1.240 (1.152-1.335) | 1.13E-08 | 5.51E-06 |
| CXCL9 | Q07325 | 1.187 (1.103-1.278) | 4.66E-06 | 3.58E-04 |
| EFNA4 | P52798 | 1.206 (1.121-1.297) | 4.95E-07 | 7.18E-05 |
| EGFR | P00533 | 0.834 (0.776-0.896) | 6.93E-07 | 8.42E-05 |
| FN1 | P02751 | 1.248 (1.144-1.361) | 6.13E-07 | 7.91E-05 |
| GAST | P01350 | 1.216 (1.131-1.306) | 9.97E-08 | 2.64E-05 |
| GDF15 | Q99988 | 1.261 (1.187-1.340) | 4.69E-14 | 1.37E-10 |
| GFRA1 | P56159 | 1.198 (1.108-1.295) | 5.27E-06 | 3.85E-04 |
| GRPEL1 | Q9HAV7 | 1.205 (1.123-1.293) | 2.01E-07 | 4.15E-05 |
| HAVCR1 | Q96D42 | 1.207 (1.118-1.304) | 1.59E-06 | 1.54E-04 |
| HGF | P14210 | 1.251 (1.156-1.354) | 2.48E-08 | 9.04E-06 |
| ICAM1 | P05362 | 1.183 (1.100-1.272) | 6.43E-06 | 4.09E-04 |
| IGFBP4 | P22692 | 1.204 (1.116-1.299) | 1.77E-06 | 1.60E-04 |
| IGSF8 | Q969P0 | 1.179 (1.098-1.265) | 5.79E-06 | 4.02E-04 |
| IL18BP | O95998 | 1.192 (1.109-1.282) | 2.14E-06 | 1.84E-04 |
| IL18R1 | Q13478 | 1.206 (1.119-1.300) | 9.53E-07 | 1.11E-04 |
| IL1RN | P18510 | 1.195 (1.111-1.286) | 1.68E-06 | 1.58E-04 |
| IL4R | P24394 | 1.217 (1.137-1.303) | 1.37E-08 | 5.71E-06 |
| KRT19 | P08727 | 1.186 (1.103-1.275) | 3.58E-06 | 2.82E-04 |
| LGALS4 | P56470 | 1.220 (1.133-1.314) | 1.41E-07 | 3.17E-05 |
| LTBP3 | Q9NS15 | 1.177 (1.105-1.254) | 4.30E-07 | 6.60E-05 |
| MRC1 | P22897 | 1.213 (1.118-1.315) | 3.18E-06 | 2.58E-04 |
| OCLN | Q16625 | 1.210 (1.114-1.313) | 5.52E-06 | 3.93E-04 |
| PIGR | P01833 | 1.223 (1.136-1.316) | 7.92E-08 | 2.31E-05 |
| PLA2G15 | Q8NCC3 | 1.210 (1.125-1.303) | 3.59E-07 | 5.93E-05 |
| PLAUR | Q03405 | 1.264 (1.171-1.364) | 2.04E-09 | 1.41E-06 |
| SCARB2 | Q14108 | 1.206 (1.118-1.302) | 1.54E-06 | 1.54E-04 |
| TGFA | P01135 | 1.243 (1.158-1.334) | 1.74E-09 | 1.41E-06 |
| TIMP1 | P01033 | 1.199 (1.113-1.292) | 1.82E-06 | 1.60E-04 |
| TNFRSF10A | O00220 | 1.224 (1.145-1.308) | 2.42E-09 | 1.41E-06 |
| TNFRSF10B | O14763 | 1.161 (1.113-1.211) | 5.60E-12 | 8.17E-09 |
| TNFRSF1A | P19438 | 1.220 (1.130-1.317) | 3.66E-07 | 5.93E-05 |
| TNFRSF1B | P20333 | 1.152 (1.095-1.213) | 6.22E-08 | 2.01E-05 |
| TNFSF11 | O14788 | 0.827 (0.766-0.892) | 9.93E-07 | 1.11E-04 |
| TNFSF13 | O75888 | 1.191 (1.104-1.285) | 6.21E-06 | 4.09E-04 |
| UMOD | P07911 | 0.837 (0.779-0.900) | 1.34E-06 | 1.45E-04 |
| VSIG2 | Q96IQ7 | 1.217 (1.127-1.314) | 5.17E-07 | 7.18E-05 |
| VSIG4 | Q9Y279 | 1.198 (1.108-1.296) | 6.16E-06 | 4.09E-04 |
| ADA2 | Q9NZK5 | 1.169 (1.085-1.260) | 3.78E-05 | 0.001 |
| ASGR1 | P07306 | 1.193 (1.103-1.291) | 9.63E-06 | 0.001 |
| BAIAP2 | Q9UQB8 | 1.169 (1.086-1.259) | 3.20E-05 | 0.001 |
| BCAN | Q96GW7 | 0.835 (0.770-0.904) | 9.33E-06 | 0.001 |
| CCL7 | P80098 | 1.162 (1.087-1.242) | 9.82E-06 | 0.001 |
| CD276 | Q5ZPR3 | 1.176 (1.089-1.269) | 3.26E-05 | 0.001 |
| CD300E | Q496F6 | 1.186 (1.097-1.283) | 1.81E-05 | 0.001 |
| CD80 | P33681 | 1.187 (1.097-1.284) | 2.04E-05 | 0.001 |
| CD83 | Q01151 | 1.173 (1.088-1.264) | 2.93E-05 | 0.001 |
| CSF1 | P09603 | 1.171 (1.086-1.261) | 3.63E-05 | 0.001 |
| CTSL | P07711 | 1.172 (1.086-1.264) | 4.02E-05 | 0.001 |
| CXADR | P78310 | 1.161 (1.082-1.245) | 3.12E-05 | 0.001 |
| DPP7 | Q9UHL4 | 1.178 (1.093-1.269) | 1.68E-05 | 0.001 |
| FSTL3 | O95633 | 1.188 (1.096-1.287) | 2.51E-05 | 0.001 |
| IFI30 | P13284 | 1.209 (1.108-1.318) | 1.96E-05 | 0.001 |
| IL10 | P22301 | 1.151 (1.082-1.224) | 9.02E-06 | 0.001 |
| IL17F | Q96PD4 | 1.145 (1.075-1.219) | 2.48E-05 | 0.001 |
| IL2RA | P01589 | 1.166 (1.085-1.254) | 3.29E-05 | 0.001 |
| IMMT | Q16891 | 1.168 (1.089-1.253) | 1.40E-05 | 0.001 |
| ITGAV | P06756 | 0.844 (0.781-0.911) | 1.44E-05 | 0.001 |
| LGALS9 | O00182 | 1.196 (1.101-1.299) | 2.10E-05 | 0.001 |
| LILRA5 | A6NI73 | 1.193 (1.102-1.291) | 1.26E-05 | 0.001 |
| NECTIN2 | Q92692 | 1.177 (1.095-1.264) | 8.98E-06 | 0.001 |
| NHLRC3 | Q5JS37 | 1.202 (1.102-1.312) | 3.51E-05 | 0.001 |
| PRSS8 | Q16651 | 1.190 (1.096-1.292) | 3.59E-05 | 0.001 |
| PRTN3 | P24158 | 1.144 (1.076-1.216) | 1.81E-05 | 0.001 |
| RBFOX3 | A6NFN3 | 1.199 (1.105-1.300) | 1.20E-05 | 0.001 |
| RNASE6 | Q93091 | 1.157 (1.083-1.235) | 1.37E-05 | 0.001 |
| SCGB1A1 | P11684 | 0.853 (0.793-0.918) | 2.38E-05 | 0.001 |
| SIGLEC1 | Q9BZZ2 | 1.179 (1.091-1.275) | 3.54E-05 | 0.001 |
| TFF3 | Q07654 | 1.143 (1.073-1.218) | 3.62E-05 | 0.001 |
| TNF | P01375 | 1.120 (1.063-1.180) | 2.08E-05 | 0.001 |
| TNFRSF14 | Q92956 | 1.178 (1.096-1.267) | 8.82E-06 | 0.001 |
| WFDC2 | Q14508 | 1.181 (1.092-1.277) | 3.03E-05 | 0.001 |
| DDAH1 | O94760 | 1.161 (1.079-1.249) | 6.11E-05 | 0.002 |
| ECHDC3 | Q96DC8 | 1.168 (1.083-1.260) | 6.12E-05 | 0.002 |
| EPHA2 | P29317 | 1.164 (1.081-1.252) | 5.33E-05 | 0.002 |
| LRRN1 | Q6UXK5 | 0.857 (0.794-0.925) | 7.37E-05 | 0.002 |
| MAD1L1 | Q9Y6D9 | 1.140 (1.068-1.216) | 7.37E-05 | 0.002 |
| MEP1B | Q16820 | 0.861 (0.801-0.926) | 5.05E-05 | 0.002 |
| MVK | Q03426 | 1.167 (1.081-1.258) | 6.80E-05 | 0.002 |
| OSMR | Q99650 | 1.165 (1.082-1.253) | 4.55E-05 | 0.002 |
| RNASET2 | O00584 | 1.151 (1.074-1.234) | 7.25E-05 | 0.002 |
| SIGLEC10 | Q96LC7 | 1.164 (1.082-1.252) | 4.34E-05 | 0.002 |
| ACVRL1 | P37023 | 1.164 (1.078-1.258) | 1.11E-04 | 0.003 |
| CD274 | Q9NZQ7 | 1.150 (1.073-1.233) | 8.11E-05 | 0.003 |
| CEACAM6 | P40199 | 1.170 (1.080-1.268) | 1.24E-04 | 0.003 |
| DEFB4A_DEFB4B | O15263 | 1.166 (1.078-1.261) | 1.24E-04 | 0.003 |
| GKN1 | Q9NS71 | 1.146 (1.070-1.228) | 1.06E-04 | 0.003 |
| HAVCR2 | Q8TDQ0 | 1.165 (1.080-1.257) | 8.41E-05 | 0.003 |
| IL15 | P40933 | 1.152 (1.073-1.237) | 8.91E-05 | 0.003 |
| IL18 | Q14116 | 1.153 (1.073-1.238) | 1.03E-04 | 0.003 |
| LEP | P41159 | 1.311 (1.145-1.501) | 8.68E-05 | 0.003 |
| OSM | P13725 | 1.162 (1.077-1.253) | 1.12E-04 | 0.003 |
| PON1 | P27169 | 0.878 (0.822-0.938) | 1.03E-04 | 0.003 |
| TGFB1 | P01137 | 1.150 (1.072-1.234) | 9.88E-05 | 0.003 |
| TYMP | P19971 | 1.164 (1.079-1.255) | 8.47E-05 | 0.003 |
| VNN2 | O95498 | 1.168 (1.079-1.265) | 1.21E-04 | 0.003 |
| C9 | P02748 | 1.181 (1.083-1.288) | 1.69E-04 | 0.004 |
| CEACAM5 | P06731 | 1.158 (1.074-1.249) | 1.40E-04 | 0.004 |
| CNTN5 | O94779 | 0.851 (0.784-0.925) | 1.35E-04 | 0.004 |
| FABP1 | P07148 | 1.163 (1.077-1.257) | 1.29E-04 | 0.004 |
| IL6 | P05231 | 1.143 (1.066-1.226) | 1.71E-04 | 0.004 |
| PAG1 | Q9NWQ8 | 1.146 (1.068-1.229) | 1.49E-04 | 0.004 |
| RARRES2 | Q99969 | 1.165 (1.076-1.262) | 1.57E-04 | 0.004 |
| SERPINA3 | P01011 | 1.137 (1.064-1.214) | 1.37E-04 | 0.004 |
| THBS2 | P35442 | 1.142 (1.066-1.223) | 1.46E-04 | 0.004 |
| A1BG | P04217 | 1.178 (1.081-1.285) | 2.04E-04 | 0.005 |
| CFH | P08603 | 1.198 (1.088-1.318) | 2.22E-04 | 0.005 |
| GIPC2 | Q8TF65 | 1.150 (1.068-1.239) | 2.33E-04 | 0.005 |
| IL19 | Q9UHD0 | 1.146 (1.066-1.232) | 2.30E-04 | 0.005 |
| ITIH3 | Q06033 | 1.165 (1.074-1.263) | 2.23E-04 | 0.005 |
| MDK | P21741 | 1.143 (1.065-1.227) | 2.08E-04 | 0.005 |
| PALM2 | Q8IXS6 | 1.177 (1.080-1.282) | 1.98E-04 | 0.005 |
| REN | P00797 | 1.143 (1.065-1.227) | 2.19E-04 | 0.005 |
| SMPD1 | P17405 | 1.149 (1.068-1.236) | 2.02E-04 | 0.005 |
| TFF2 | Q03403 | 1.151 (1.069-1.240) | 2.13E-04 | 0.005 |
| TNFRSF11A | Q9Y6Q6 | 1.154 (1.071-1.244) | 1.82E-04 | 0.005 |
| TNFRSF11B | O00300 | 1.160 (1.072-1.255) | 2.28E-04 | 0.005 |
| VCAM1 | P19320 | 1.144 (1.065-1.228) | 2.33E-04 | 0.005 |
| ACE2 | Q9BYF1 | 1.156 (1.069-1.249) | 2.55E-04 | 0.006 |
| AIFM1 | O95831 | 1.149 (1.065-1.239) | 3.11E-04 | 0.006 |
| C1RL | Q9NZP8 | 1.172 (1.076-1.276) | 2.54E-04 | 0.006 |
| CD74 | P04233 | 1.155 (1.069-1.248) | 2.59E-04 | 0.006 |
| COL4A1 | P02462 | 1.145 (1.064-1.231) | 2.72E-04 | 0.006 |
| CPM | P14384 | 1.162 (1.072-1.260) | 2.58E-04 | 0.006 |
| FSTL1 | Q12841 | 1.149 (1.065-1.238) | 3.03E-04 | 0.006 |
| GPKOW | Q92917 | 1.111 (1.049-1.176) | 2.91E-04 | 0.006 |
| IL1RL1 | Q01638 | 1.161 (1.071-1.260) | 3.13E-04 | 0.006 |
| ITGBL1 | O95965 | 1.159 (1.071-1.255) | 2.63E-04 | 0.006 |
| LAIR1 | Q6GTX8 | 1.132 (1.059-1.211) | 2.82E-04 | 0.006 |
| LRIG1 | Q96JA1 | 1.148 (1.066-1.237) | 2.65E-04 | 0.006 |
| NID1 | P14543 | 1.147 (1.065-1.234) | 2.76E-04 | 0.006 |
| NTRK3 | Q16288 | 0.866 (0.802-0.936) | 3.02E-04 | 0.006 |
| OLR1 | P78380 | 1.131 (1.058-1.210) | 3.05E-04 | 0.006 |
| TNFRSF6B | O95407 | 1.151 (1.068-1.241) | 2.47E-04 | 0.006 |
| ADAMTSL2 | Q86TH1 | 1.157 (1.068-1.254) | 3.51E-04 | 0.007 |
| CA12 | O43570 | 1.140 (1.061-1.226) | 3.76E-04 | 0.007 |
| FGFR4 | P22455 | 1.148 (1.064-1.239) | 3.63E-04 | 0.007 |
| FOXO3 | O43524 | 1.142 (1.062-1.229) | 3.51E-04 | 0.007 |
| IDUA | P35475 | 1.147 (1.064-1.238) | 3.83E-04 | 0.007 |
| PI3 | P19957 | 1.143 (1.062-1.229) | 3.62E-04 | 0.007 |
| TMSB10 | P63313 | 1.151 (1.065-1.245) | 3.79E-04 | 0.007 |
| WFIKKN1 | Q96NZ8 | 0.863 (0.796-0.935) | 3.35E-04 | 0.007 |
| AGR2 | O95994 | 1.139 (1.059-1.226) | 4.69E-04 | 0.008 |
| CD4 | P01730 | 1.111 (1.048-1.178) | 4.39E-04 | 0.008 |
| CD79B | P40259 | 1.141 (1.061-1.228) | 4.06E-04 | 0.008 |
| CKAP4 | Q07065 | 1.138 (1.059-1.222) | 4.20E-04 | 0.008 |
| COL6A3 | P12111 | 1.151 (1.064-1.244) | 4.09E-04 | 0.008 |
| CTSZ | Q9UBR2 | 1.147 (1.063-1.238) | 4.28E-04 | 0.008 |
| KLRB1 | Q12918 | 1.140 (1.060-1.228) | 4.66E-04 | 0.008 |
| LMNB2 | Q03252 | 1.159 (1.067-1.259) | 4.53E-04 | 0.008 |
| RNF149 | Q8NC42 | 1.155 (1.066-1.251) | 4.19E-04 | 0.008 |
| TNFRSF8 | P28908 | 1.138 (1.059-1.223) | 4.56E-04 | 0.008 |
| TNFRSF9 | Q07011 | 1.140 (1.060-1.226) | 4.22E-04 | 0.008 |
| XCL1 | P47992 | 1.130 (1.056-1.210) | 4.31E-04 | 0.008 |
| ITGA11 | Q9UKX5 | 0.875 (0.812-0.943) | 4.98E-04 | 0.009 |
| CFHR5 | Q9BXR6 | 1.163 (1.068-1.267) | 0.001 | 0.009 |
| NPC2 | P61916 | 1.150 (1.062-1.245) | 0.001 | 0.009 |
| PDZK1 | Q5T2W1 | 1.142 (1.059-1.231) | 0.001 | 0.009 |
| PSAP | P07602 | 1.128 (1.054-1.207) | 0.001 | 0.009 |
| TNFRSF4 | P43489 | 1.138 (1.058-1.224) | 0.001 | 0.009 |
| YAP1 | P46937 | 1.166 (1.069-1.271) | 0.001 | 0.009 |
| CHCHD10 | Q8WYQ3 | 1.156 (1.064-1.256) | 0.001 | 0.01 |
| GGH | Q92820 | 1.147 (1.061-1.240) | 0.001 | 0.01 |
| LRG1 | P02750 | 1.158 (1.065-1.259) | 0.001 | 0.01 |
| NTproBNP | NTproBNP | 1.144 (1.060-1.235) | 0.001 | 0.01 |
| POLR2F | P61218 | 1.123 (1.051-1.200) | 0.001 | 0.01 |
| B2M | P61769 | 1.111 (1.046-1.180) | 0.001 | 0.011 |
| TP53INP1 | Q96A56 | 0.852 (0.776-0.934) | 0.001 | 0.011 |
| CEACAM8 | P31997 | 1.131 (1.053-1.215) | 0.001 | 0.012 |
| CNTN1 | Q12860 | 0.872 (0.805-0.945) | 0.001 | 0.012 |
| CTSO | P43234 | 1.134 (1.054-1.220) | 0.001 | 0.012 |
| DPY30 | Q9C005 | 1.134 (1.054-1.221) | 0.001 | 0.012 |
| FAP | Q12884 | 0.879 (0.815-0.948) | 0.001 | 0.012 |
| FGFBP3 | Q8TAT2 | 0.870 (0.802-0.943) | 0.001 | 0.012 |
| GSN | P06396 | 0.915 (0.869-0.963) | 0.001 | 0.012 |
| KLK4 | Q9Y5K2 | 1.138 (1.055-1.226) | 0.001 | 0.012 |
| LAMP3 | Q9UQV4 | 1.147 (1.059-1.242) | 0.001 | 0.012 |
| SELENOP | P49908 | 0.869 (0.800-0.943) | 0.001 | 0.012 |
| SIAE | Q9HAT2 | 1.137 (1.055-1.225) | 0.001 | 0.012 |
| TNFRSF12A | Q9NP84 | 1.144 (1.058-1.237) | 0.001 | 0.012 |
| TNR | Q92752 | 0.874 (0.808-0.945) | 0.001 | 0.012 |
| ALDH3A1 | P30838 | 1.119 (1.047-1.196) | 0.001 | 0.013 |
| ANXA10 | Q9UJ72 | 1.129 (1.051-1.213) | 0.001 | 0.013 |
| CA14 | Q9ULX7 | 0.871 (0.802-0.945) | 0.001 | 0.013 |
| CD72 | P21854 | 1.154 (1.061-1.256) | 0.001 | 0.013 |
| FCRLB | Q6BAA4 | 1.123 (1.049-1.202) | 0.001 | 0.013 |
| HDGF | P51858 | 1.138 (1.055-1.228) | 0.001 | 0.013 |
| HPGDS | O60760 | 0.878 (0.813-0.948) | 0.001 | 0.013 |
| MPO | P05164 | 1.123 (1.049-1.202) | 0.001 | 0.013 |
| NCS1 | P62166 | 1.147 (1.058-1.243) | 0.001 | 0.013 |
| NPDC1 | Q9NQX5 | 1.131 (1.052-1.215) | 0.001 | 0.013 |
| OMG | P23515 | 0.878 (0.814-0.948) | 0.001 | 0.013 |
| PRAP1 | Q96NZ9 | 1.173 (1.068-1.289) | 0.001 | 0.013 |
| PTX3 | P26022 | 1.151 (1.060-1.250) | 0.001 | 0.013 |
| ADAM12 | O43184 | 1.105 (1.041-1.172) | 0.001 | 0.014 |
| DLL1 | O00548 | 1.136 (1.053-1.225) | 0.001 | 0.014 |
| ELN | P15502 | 1.190 (1.073-1.321) | 0.001 | 0.014 |
| FGF12 | P61328 | 1.156 (1.061-1.261) | 0.001 | 0.014 |
| INSL3 | P51460 | 0.778 (0.670-0.903) | 0.001 | 0.014 |
| MILR1 | Q7Z6M3 | 1.131 (1.051-1.217) | 0.001 | 0.014 |
| OXCT1 | P55809 | 1.138 (1.054-1.230) | 0.001 | 0.014 |
| SLAMF8 | Q9P0V8 | 1.143 (1.055-1.237) | 0.001 | 0.014 |
| AMBP | P02760 | 1.141 (1.054-1.236) | 0.001 | 0.015 |
| IL10RB | Q08334 | 1.126 (1.049-1.208) | 0.001 | 0.015 |
| MMP9 | P14780 | 1.137 (1.053-1.227) | 0.001 | 0.015 |
| MYBPC2 | Q14324 | 1.126 (1.049-1.210) | 0.001 | 0.015 |
| NT5C1A | Q9BXI3 | 1.124 (1.048-1.205) | 0.001 | 0.015 |
| RBP5 | P82980 | 1.142 (1.055-1.237) | 0.001 | 0.015 |
| RRM2 | P31350 | 1.105 (1.041-1.173) | 0.001 | 0.015 |
| SELP | P16109 | 1.130 (1.050-1.215) | 0.001 | 0.015 |
| GCNT1 | Q02742 | 1.132 (1.050-1.220) | 0.001 | 0.016 |
| MERTK | Q12866 | 1.132 (1.050-1.221) | 0.001 | 0.016 |
| MME | P08473 | 1.130 (1.050-1.217) | 0.001 | 0.016 |
| PLIN3 | O60664 | 1.128 (1.049-1.214) | 0.001 | 0.016 |
| RNASE4 | P34096 | 1.131 (1.050-1.219) | 0.001 | 0.016 |
| SCGN | O76038 | 1.129 (1.049-1.215) | 0.001 | 0.016 |
| APLP1 | P51693 | 0.875 (0.807-0.949) | 0.001 | 0.017 |
| APOM | O95445 | 0.884 (0.820-0.953) | 0.001 | 0.017 |
| EPPK1 | P58107 | 1.131 (1.049-1.220) | 0.001 | 0.017 |
| MARCO | Q9UEW3 | 1.135 (1.050-1.227) | 0.001 | 0.017 |
| MMP7 | P09237 | 1.142 (1.052-1.239) | 0.001 | 0.018 |
| RNASE1 | P07998 | 1.128 (1.047-1.214) | 0.001 | 0.018 |
| RPS10 | P46783 | 0.854 (0.775-0.941) | 0.001 | 0.018 |
| ADGRF5 | Q8IZF2 | 1.144 (1.053-1.243) | 0.001 | 0.019 |
| CEACAM1 | P13688 | 1.133 (1.049-1.224) | 0.002 | 0.019 |
| TF | P02787 | 1.137 (1.050-1.232) | 0.002 | 0.019 |
| TPP1 | O14773 | 1.118 (1.043-1.197) | 0.002 | 0.019 |
| ADM | P35318 | 1.158 (1.057-1.269) | 0.002 | 0.02 |
| NCR3LG1 | Q68D85 | 1.145 (1.052-1.246) | 0.002 | 0.02 |
| SOD2 | P04179 | 1.121 (1.044-1.203) | 0.002 | 0.02 |
| CCL15 | Q16663 | 1.120 (1.043-1.203) | 0.002 | 0.021 |
| CLEC6A | Q6EIG7 | 1.126 (1.045-1.213) | 0.002 | 0.021 |
| GFER | P55789 | 1.129 (1.046-1.218) | 0.002 | 0.021 |
| IGF2R | P11717 | 1.131 (1.047-1.221) | 0.002 | 0.021 |
| POF1B | Q8WVV4 | 1.120 (1.043-1.202) | 0.002 | 0.021 |
| EDN1 | P05305 | 1.140 (1.050-1.238) | 0.002 | 0.022 |
| FETUB | Q9UGM5 | 1.138 (1.049-1.234) | 0.002 | 0.022 |
| PTPRF | P10586 | 0.885 (0.820-0.956) | 0.002 | 0.022 |
| SCARF1 | Q14162 | 1.123 (1.044-1.207) | 0.002 | 0.022 |
| TNC | P24821 | 1.122 (1.044-1.207) | 0.002 | 0.022 |
| ADAMTS13 | Q76LX8 | 0.890 (0.827-0.958) | 0.002 | 0.023 |
| ARSB | P15848 | 1.123 (1.043-1.208) | 0.002 | 0.023 |
| CD27 | P26842 | 1.125 (1.044-1.212) | 0.002 | 0.023 |
| GGT1 | P19440 | 1.132 (1.046-1.225) | 0.002 | 0.023 |
| GPNMB | Q14956 | 1.128 (1.045-1.219) | 0.002 | 0.024 |
| SIT1 | Q9Y3P8 | 1.131 (1.046-1.223) | 0.002 | 0.024 |
| ADGRG1 | Q9Y653 | 1.114 (1.040-1.194) | 0.002 | 0.025 |
| AMIGO2 | Q86SJ2 | 1.122 (1.042-1.208) | 0.002 | 0.025 |
| ASGR2 | P07307 | 1.130 (1.045-1.222) | 0.002 | 0.025 |
| C7 | P10643 | 1.140 (1.048-1.239) | 0.002 | 0.025 |
| CBLN4 | Q9NTU7 | 0.891 (0.827-0.960) | 0.002 | 0.025 |
| COL5A1 | P20908 | 1.129 (1.045-1.221) | 0.002 | 0.025 |
| HPSE | Q9Y251 | 1.141 (1.049-1.241) | 0.002 | 0.025 |
| LETM1 | O95202 | 1.129 (1.044-1.221) | 0.002 | 0.025 |
| MMP3 | P08254 | 1.152 (1.052-1.261) | 0.002 | 0.025 |
| MSR1 | P21757 | 1.136 (1.047-1.233) | 0.002 | 0.025 |
| PLXNB2 | O15031 | 1.121 (1.042-1.207) | 0.002 | 0.025 |
| SERPINA11 | Q86U17 | 1.138 (1.048-1.236) | 0.002 | 0.025 |
| MAP1LC3A | Q9H492 | 0.881 (0.812-0.956) | 0.002 | 0.026 |
| COL18A1 | P39060 | 1.132 (1.044-1.226) | 0.003 | 0.027 |
| CCL5 | P13501 | 1.120 (1.040-1.205) | 0.003 | 0.028 |
| TNFSF13B | Q9Y275 | 1.123 (1.041-1.212) | 0.003 | 0.028 |
| CXCL11 | O14625 | 1.116 (1.039-1.200) | 0.003 | 0.029 |
| SIGLEC6 | O43699 | 1.113 (1.038-1.194) | 0.003 | 0.029 |
| TMED4 | Q7Z7H5 | 0.860 (0.780-0.949) | 0.003 | 0.029 |
| CXCL8 | P10145 | 1.112 (1.037-1.191) | 0.003 | 0.03 |
| FAM3C | Q92520 | 1.124 (1.041-1.214) | 0.003 | 0.03 |
| QSOX1 | O00391 | 1.095 (1.032-1.162) | 0.003 | 0.03 |
| BAX | Q07812 | 1.121 (1.039-1.208) | 0.003 | 0.032 |
| DLK1 | P80370 | 0.890 (0.823-0.961) | 0.003 | 0.032 |
| SPINK1 | P00995 | 1.113 (1.037-1.195) | 0.003 | 0.032 |
| SPON2 | Q9BUD6 | 1.124 (1.040-1.214) | 0.003 | 0.032 |
| VEGFA | P15692 | 1.117 (1.038-1.202) | 0.003 | 0.032 |
| ZNRD2 | O60232 | 1.124 (1.040-1.215) | 0.003 | 0.032 |
| CD5 | P06127 | 1.111 (1.036-1.192) | 0.003 | 0.033 |
| PKD1 | P98161 | 1.141 (1.045-1.246) | 0.003 | 0.033 |
| TNFSF14 | O43557 | 1.119 (1.038-1.206) | 0.003 | 0.033 |
| EGFL7 | Q9UHF1 | 1.120 (1.038-1.208) | 0.003 | 0.034 |
| ANGPT2 | O15123 | 1.119 (1.038-1.206) | 0.004 | 0.035 |
| CD163 | Q86VB7 | 1.119 (1.038-1.207) | 0.003 | 0.035 |
| CLEC7A | Q9BXN2 | 1.135 (1.042-1.236) | 0.004 | 0.035 |
| GRN | P28799 | 1.118 (1.037-1.205) | 0.003 | 0.035 |
| ILKAP | Q9H0C8 | 1.111 (1.035-1.192) | 0.003 | 0.035 |
| PGF | P49763 | 1.115 (1.037-1.200) | 0.003 | 0.035 |
| SCLY | Q96I15 | 1.122 (1.039-1.212) | 0.003 | 0.035 |
| ACAA1 | P09110 | 1.117 (1.036-1.204) | 0.004 | 0.036 |
| DSC2 | Q02487 | 1.119 (1.037-1.207) | 0.004 | 0.036 |
| NAGPA | Q9UK23 | 1.133 (1.041-1.232) | 0.004 | 0.036 |
| NDUFA5 | Q16718 | 1.128 (1.040-1.223) | 0.004 | 0.036 |
| SPINK4 | O60575 | 1.116 (1.037-1.202) | 0.004 | 0.036 |
| VWA1 | Q6PCB0 | 1.121 (1.038-1.210) | 0.004 | 0.036 |
| FAS | P25445 | 1.101 (1.031-1.175) | 0.004 | 0.037 |
| FGR | P09769 | 1.106 (1.033-1.183) | 0.004 | 0.037 |
| SORT1 | Q99523 | 1.116 (1.036-1.202) | 0.004 | 0.037 |
| WNT9A | O14904 | 1.122 (1.038-1.214) | 0.004 | 0.037 |
| CCL3 | P10147 | 1.084 (1.026-1.145) | 0.004 | 0.038 |
| CST3 | P01034 | 1.126 (1.039-1.221) | 0.004 | 0.038 |
| QPCT | Q16769 | 1.117 (1.036-1.204) | 0.004 | 0.038 |
| RAB6A | P20340 | 0.896 (0.831-0.966) | 0.004 | 0.038 |
| UBE2L6 | O14933 | 1.127 (1.039-1.223) | 0.004 | 0.038 |
| ECHS1 | P30084 | 1.131 (1.040-1.230) | 0.004 | 0.039 |
| HYAL1 | Q12794 | 1.111 (1.034-1.195) | 0.004 | 0.039 |
| OSCAR | Q8IYS5 | 1.118 (1.036-1.207) | 0.004 | 0.039 |
| CANT1 | Q8WVQ1 | 1.118 (1.036-1.208) | 0.004 | 0.04 |
| CRYBB2 | P43320 | 1.092 (1.028-1.160) | 0.004 | 0.04 |
| VWF | P04275 | 1.115 (1.035-1.201) | 0.004 | 0.04 |
| BCAT1 | P54687 | 1.117 (1.035-1.205) | 0.005 | 0.042 |
| CD34 | P28906 | 0.897 (0.832-0.967) | 0.005 | 0.042 |
| CD36 | P16671 | 1.123 (1.037-1.217) | 0.005 | 0.042 |
| DBI | P07108 | 1.118 (1.035-1.207) | 0.005 | 0.042 |
| DCBLD2 | Q96PD2 | 1.116 (1.034-1.204) | 0.005 | 0.042 |
| GOLM2 | Q6P4E1 | 1.107 (1.032-1.188) | 0.005 | 0.042 |
| HMOX2 | P30519 | 1.104 (1.031-1.182) | 0.005 | 0.042 |
| VSIR | Q9H7M9 | 1.108 (1.032-1.189) | 0.005 | 0.042 |
| APBB1IP | Q7Z5R6 | 1.106 (1.031-1.187) | 0.005 | 0.043 |
| ASAH1 | Q13510 | 1.112 (1.033-1.198) | 0.005 | 0.043 |
| CFB | P00751 | 1.137 (1.040-1.244) | 0.005 | 0.043 |
| CLEC5A | Q9NY25 | 1.113 (1.033-1.199) | 0.005 | 0.043 |
| FCN1 | O00602 | 1.138 (1.040-1.245) | 0.005 | 0.043 |
| HHEX | Q03014 | 1.128 (1.038-1.226) | 0.005 | 0.043 |
| SORCS2 | Q96PQ0 | 1.117 (1.034-1.206) | 0.005 | 0.043 |
| CALCA | P01258 | 1.136 (1.039-1.242) | 0.005 | 0.044 |
| METAP1D | Q6UB28 | 1.115 (1.034-1.204) | 0.005 | 0.044 |
| TMED10 | P49755 | 0.848 (0.756-0.952) | 0.005 | 0.044 |
| KRT18 | P05783 | 1.117 (1.034-1.208) | 0.005 | 0.045 |
| EFNA1 | P20827 | 1.114 (1.033-1.202) | 0.005 | 0.046 |
| NUCB2 | P80303 | 1.112 (1.032-1.199) | 0.005 | 0.046 |
| ITGA5 | P08648 | 1.116 (1.033-1.206) | 0.005 | 0.047 |
| HEG1 | Q9ULI3 | 1.130 (1.037-1.232) | 0.006 | 0.048 |
| CDH2 | P19022 | 1.121 (1.034-1.215) | 0.006 | 0.049 |
| ESAM | Q96AP7 | 1.108 (1.030-1.191) | 0.006 | 0.049 |
| GUSB | P08236 | 1.121 (1.034-1.215) | 0.006 | 0.049 |
| HEPACAM2 | A8MVW5 | 0.891 (0.821-0.967) | 0.006 | 0.049 |
| KIR3DL1 | P43629 | 1.109 (1.031-1.192) | 0.006 | 0.049 |

| **Supplementary Table 22. Significant plasma protein mediators of the association between workplace noise exposure and anxiety disorder.** | | | | | | | |
| --- | --- | --- | --- | --- | --- | --- | --- |
| **Mediator** | **Prop_Mediated** | **P. ACME** | **P.ADE** | **P.Total_Effect** | **ACME_FDR** | **ADE_FDR** | **Total_FDR** |
| ACE2 | 0.024 | 0.006 | 0.001 | 0.001 | 0.009 | 0.003 | 0.002 |
| ADGRG1 | 0.024 | 0.007 | 0.002 | 0.002 | 0.009 | 0.004 | 0.003 |
| AREG | 0.036 | 4.00E-04 | 0.003 | 0.001 | 0.001 | 0.004 | 0.002 |
| ASGR1 | 0.039 | <0.001 | 8.00E-04 | 4.00E-04 | <0.001 | 0.003 | 0.002 |
| BST2 | 0.043 | 4.00E-04 | 8.00E-04 | 4.00E-04 | 0.001 | 0.003 | 0.002 |
| CA14 | 0.030 | 0.0032 | <0.001 | <0.001 | 0.005 | <0.001 | <0.001 |
| CA6 | 0.037 | 4.00E-04 | 0.003 | 0.002 | 0.001 | 0.004 | 0.003 |
| CD302 | 0.029 | 0.006 | 0.002 | 0.002 | 0.008 | 0.004 | 0.003 |
| CD80 | 0.041 | 0.011 | 0.045 | 0.037 | 0.012 | 0.050 | 0.041 |
| CD83 | 0.038 | 4.00E-04 | 0.002 | 0.002 | 0.001 | 0.004 | 0.003 |
| CD99L2 | 0.047 | 4.00E-04 | 0.003 | 0.002 | 0.001 | 0.004 | 0.003 |
| CDCP1 | 0.071 | 0.013 | 0.002 | 8.00E-04 | 0.013 | 0.004 | 0.002 |
| CEACAM5 | 0.025 | 0.004 | 0.004 | 0.003 | 0.006 | 0.005 | 0.004 |
| CHGA | 0.068 | <0.001 | 0.060 | 0.045 | <0.001 | 0.063 | 0.047 |
| COL18A1 | 0.031 | 0.003 | 0.002 | 4.00E-04 | 0.005 | 0.004 | 0.002 |
| CXCL17 | 0.042 | 0.002 | 0.002 | 0.001 | 0.003 | 0.004 | 0.002 |
| DCBLD2 | 0.026 | 0.004 | 4.00E-04 | <0.001 | 0.006 | 0.003 | <0.001 |
| FGF23 | 0.032 | 0.002 | 0.001 | 8.00E-04 | 0.003 | 0.003 | 0.002 |
| GAST | 0.092 | <0.001 | 0.075 | 0.046 | <0.001 | 0.076 | 0.047 |
| GDF15 | 0.049 | <0.001 | 8.00E-04 | 4.00E-04 | <0.001 | 0.003 | 0.002 |
| GLA | 0.043 | 0.010 | 0.052 | 0.040 | 0.011 | 0.056 | 0.043 |
| GPNMB | 0.035 | 8.00E-04 | 0.002 | 0.002 | 0.002 | 0.004 | 0.003 |
| GUSB | 0.033 | 0.002 | 0.002 | 0.002 | 0.004 | 0.004 | 0.003 |
| HAVCR1 | 0.024 | 0.004 | <0.001 | <0.001 | 0.006 | <0.001 | <0.001 |
| HAVCR2 | 0.027 | 0.009 | 0.004 | 0.003 | 0.011 | 0.005 | 0.004 |
| IDUA | 0.026 | 0.005 | <0.001 | <0.001 | 0.007 | <0.001 | <0.001 |
| IL18 | 0.024 | 0.004 | 0.002 | 0.001 | 0.006 | 0.004 | 0.002 |
| LGALS4 | 0.036 | 4.00E-04 | 0.004 | 0.002 | 0.001 | 0.005 | 0.003 |
| LGALS9 | 0.044 | <0.001 | 0.002 | 0.002 | <0.001 | 0.004 | 0.003 |
| MEP1B | 0.022 | 0.003 | 8.00E-04 | 8.00E-04 | 0.005 | 0.003 | 0.002 |
| MMP7 | 0.029 | 0.002 | 0.001 | 8.00E-04 | 0.003 | 0.003 | 0.002 |
| NCS1 | 0.019 | 0.014 | 0.002 | 0.002 | 0.014 | 0.004 | 0.003 |
| NHLRC3 | 0.055 | 8.00E-04 | 0.045 | 0.034 | 0.002 | 0.050 | 0.038 |
| NTRK3 | 0.046 | <0.001 | 4.00E-04 | <0.001 | <0.001 | 0.003 | <0.001 |
| OCLN | 0.050 | 0.009 | 0.041 | 0.032 | 0.011 | 0.047 | 0.036 |
| OSCAR | 0.033 | 0.002 | 0.003 | 0.002 | 0.003 | 0.004 | 0.003 |
| PIGR | 0.060 | 4.00E-04 | 0.005 | 0.004 | 0.001 | 0.006 | 0.005 |
| PILRA | 0.026 | 0.007 | 0.002 | 0.001 | 0.009 | 0.004 | 0.002 |
| PILRB | 0.026 | 0.003 | 4.00E-04 | 4.00E-04 | 0.005 | 0.003 | 0.002 |
| PLA2G15 | 0.033 | 0.002 | 0.001 | 8.00E-04 | 0.003 | 0.003 | 0.002 |
| PLAUR | 0.054 | <0.001 | 0.006 | 0.004 | <0.001 | 0.007 | 0.004 |
| PRSS8 | 0.052 | <0.001 | 0.002 | 0.001 | <0.001 | 0.004 | 0.002 |
| PTPRF | 0.025 | 0.002 | 8.00E-04 | 8.00E-04 | 0.005 | 0.003 | 0.002 |
| PVR | 0.026 | 0.012 | 4.00E-04 | 4.00E-04 | 0.013 | 0.003 | 0.002 |
| REN | 0.038 | 4.00E-04 | 8.00E-04 | 8.00E-04 | 0.001 | 0.003 | 0.002 |
| SMPD1 | 0.027 | 0.002 | 8.00E-04 | 4.00E-04 | 0.004 | 0.003 | 0.002 |
| TFF2 | 0.040 | 8.00E-04 | 0.001 | 0.001 | 0.002 | 0.003 | 0.002 |
| TFF3 | 0.021 | 0.002 | 0.003 | 0.002 | 0.004 | 0.004 | 0.003 |
| TNFRSF10A | 0.026 | 0.007 | 0.001 | 4.00E-04 | 0.009 | 0.003 | 0.002 |
| TNFRSF10B | 0.039 | <0.001 | 0.002 | 0.002 | <0.001 | 0.004 | 0.003 |
| TNFRSF12A | 0.020 | 0.021 | 4.00E-04 | 4.00E-04 | 0.021 | 0.003 | 0.002 |
| TNFRSF1B | 0.021 | 0.01 | 0.002 | 0.002 | 0.011 | 0.004 | 0.003 |
| TNFSF11 | 0.027 | 0.005 | 8.00E-04 | 4.00E-04 | 0.007 | 0.003 | 0.002 |
| TPP1 | 0.021 | 0.008 | 0.003 | 0.003 | 0.010 | 0.004 | 0.004 |
| TREM2 | 0.052 | 0.004 | 0.004 | 0.002 | 0.006 | 0.005 | 0.003 |
| ULBP2 | 0.023 | 0.009 | 4.00E-04 | 4.00E-04 | 0.011 | 0.003 | 0.002 |
| VSIG2 | 0.081 | 0.002 | 0.069 | 0.044 | 0.003 | 0.071 | 0.046 |
| WFDC2 | 0.047 | <0.001 | 0.004 | 0.003 | <0.001 | 0.005 | 0.004 |

| **Supplementary Table 23. Significant plasma protein mediators of the association between workplace noise exposure and MDD.** | | | | | | | |
| --- | --- | --- | --- | --- | --- | --- | --- |
| **Mediator** | **Prop_Mediated** | **P. ACME** | **P.ADE** | **P.Total_Effect** | **ACME_FDR** | **ADE_FDR** | **Total_FDR** |
| ACE2 | 0.040 | 0.003 | 0.028 | 0.021 | 0.006 | 0.057 | 0.044 |
| ADA2 | 0.055 | 0.002 | 0.037 | 0.026 | 0.004 | 0.057 | 0.044 |
| ADGRG1 | 0.034 | 0.011 | 0.026 | 0.022 | 0.013 | 0.057 | 0.044 |
| ALDH3A1 | 0.046 | 0.002 | 0.026 | 0.020 | 0.005 | 0.057 | 0.044 |
| AMBP | 0.043 | 0.003 | 0.035 | 0.026 | 0.006 | 0.057 | 0.044 |
| ANXA10 | 0.054 | 0.003 | 0.037 | 0.029 | 0.006 | 0.057 | 0.044 |
| AREG | 0.060 | <0.001 | 0.037 | 0.027 | <0.001 | 0.057 | 0.044 |
| ASGR1 | 0.054 | <0.001 | 0.028 | 0.020 | <0.001 | 0.057 | 0.044 |
| BST2 | 0.097 | <0.001 | 0.042 | 0.022 | <0.001 | 0.060 | 0.044 |
| CA14 | 0.041 | 0.003 | 0.034 | 0.027 | 0.006 | 0.057 | 0.044 |
| CA6 | 0.089 | <0.001 | 0.066 | 0.042 | <0.001 | 0.073 | 0.048 |
| CCL5 | 0.038 | 0.004 | 0.025 | 0.018 | 0.007 | 0.057 | 0.044 |
| CD163 | 0.027 | 0.022 | 0.017 | 0.014 | 0.023 | 0.057 | 0.044 |
| CD27 | 0.032 | 0.016 | 0.034 | 0.028 | 0.018 | 0.057 | 0.044 |
| CD302 | 0.066 | <0.001 | 0.051 | 0.036 | <0.001 | 0.063 | 0.044 |
| CD72 | 0.037 | 0.022 | 0.036 | 0.032 | 0.023 | 0.057 | 0.044 |
| CD83 | 0.056 | <0.001 | 0.029 | 0.021 | <0.001 | 0.057 | 0.044 |
| CDCP1 | 0.194 | <0.001 | 0.057 | 0.022 | <0.001 | 0.067 | 0.044 |
| CEACAM5 | 0.029 | 0.018 | 0.022 | 0.019 | 0.019 | 0.057 | 0.044 |
| CHI3L1 | 0.045 | 0.006 | 0.022 | 0.016 | 0.008 | 0.057 | 0.044 |
| CNTN1 | 0.037 | 0.003 | 0.024 | 0.018 | 0.006 | 0.057 | 0.044 |
| CNTN5 | 0.073 | 8.00E-04 | 0.036 | 0.024 | 0.002 | 0.057 | 0.044 |
| COL18A1 | 0.033 | 0.006 | 0.032 | 0.027 | 0.008 | 0.057 | 0.044 |
| DCBLD2 | 0.037 | 0.006 | 0.038 | 0.032 | 0.009 | 0.057 | 0.044 |
| DLK1 | 0.028 | 0.018 | 0.020 | 0.016 | 0.019 | 0.057 | 0.044 |
| DPP7 | 0.064 | <0.001 | 0.037 | 0.026 | <0.001 | 0.057 | 0.044 |
| FETUB | 0.048 | 0.002 | 0.024 | 0.018 | 0.004 | 0.057 | 0.044 |
| GDF15 | 0.092 | <0.001 | 0.030 | 0.015 | <0.001 | 0.057 | 0.044 |
| GGH | 0.041 | 0.004 | 0.044 | 0.037 | 0.006 | 0.060 | 0.044 |
| GPNMB | 0.054 | 0.005 | 0.032 | 0.025 | 0.008 | 0.057 | 0.044 |
| GUSB | 0.042 | 0.015 | 0.029 | 0.023 | 0.017 | 0.057 | 0.044 |
| HAVCR1 | 0.052 | 0.004 | 0.032 | 0.021 | 0.006 | 0.057 | 0.044 |
| HAVCR2 | 0.054 | 8.00E-04 | 0.049 | 0.038 | 0.002 | 0.061 | 0.044 |
| HYAL1 | 0.043 | 0.009 | 0.034 | 0.025 | 0.012 | 0.057 | 0.044 |
| ICAM1 | 0.057 | 0.001 | 0.034 | 0.026 | 0.003 | 0.057 | 0.044 |
| IDUA | 0.056 | 0.002 | 0.035 | 0.024 | 0.004 | 0.057 | 0.044 |
| IL18 | 0.042 | 0.006 | 0.036 | 0.028 | 0.008 | 0.057 | 0.044 |
| IL18BP | 0.061 | 8.00E-04 | 0.020 | 0.013 | 0.002 | 0.057 | 0.044 |
| IL1RN | 0.052 | 4.00E-04 | 0.044 | 0.034 | 0.001 | 0.060 | 0.044 |
| ITGA5 | 0.033 | 0.011 | 0.036 | 0.029 | 0.013 | 0.057 | 0.044 |
| ITIH3 | 0.039 | 0.008 | 0.018 | 0.012 | 0.010 | 0.057 | 0.044 |
| KLK4 | 0.040 | 0.004 | 0.034 | 0.026 | 0.006 | 0.057 | 0.044 |
| LAMP3 | 0.042 | 0.009 | 0.022 | 0.018 | 0.012 | 0.057 | 0.044 |
| LGALS4 | 0.070 | 4.00E-04 | 0.049 | 0.031 | 0.001 | 0.061 | 0.044 |
| LGALS9 | 0.073 | <0.001 | 0.046 | 0.034 | <0.001 | 0.060 | 0.044 |
| LTBP3 | 0.049 | 0.002 | 0.044 | 0.035 | 0.005 | 0.060 | 0.044 |
| MEP1B | 0.046 | 0.001 | 0.026 | 0.020 | 0.003 | 0.057 | 0.044 |
| MILR1 | 0.042 | 0.003 | 0.034 | 0.030 | 0.006 | 0.057 | 0.044 |
| MMP7 | 0.032 | 0.011 | 0.020 | 0.017 | 0.013 | 0.057 | 0.044 |
| MRC1 | 0.073 | 0.001 | 0.069 | 0.048 | 0.003 | 0.074 | 0.053 |
| MSR1 | 0.067 | 0.007 | 0.030 | 0.020 | 0.009 | 0.057 | 0.044 |
| NCS1 | 0.033 |  | 0.032 | 0.029 | 0.013 | 0.057 | 0.044 |
| NHLRC3 | 0.072 | <0.001 | 0.046 | 0.031 | <0.001 | 0.060 | 0.044 |
| NTRK3 | 0.062 | 8.00E-04 | 0.031 | 0.024 | 0.002 | 0.057 | 0.044 |
| OSCAR | 0.031 | 0.020 | 0.036 | 0.030 | 0.021 | 0.057 | 0.044 |
| OSMR | 0.067 | 4.00E-04 | 0.018 | 0.010 | 0.001 | 0.057 | 0.044 |
| PIGR | 0.107 | <0.001 | 0.068 | 0.037 | <0.001 | 0.074 | 0.044 |
| PLA2G15 | 0.062 | 0.002 | 0.034 | 0.027 | 0.005 | 0.057 | 0.044 |
| PLAUR | 0.092 | <0.001 | 0.058 | 0.037 | <0.001 | 0.067 | 0.044 |
| PON1 | 0.036 | 0.006 | 0.052 | 0.046 | 0.008 | 0.063 | 0.052 |
| PRSS8 | 0.073 | <0.001 | 0.045 | 0.030 | <0.001 | 0.060 | 0.044 |
| PTPRF | 0.037 | 0.003 | 0.019 | 0.015 | 0.006 | 0.057 | 0.044 |
| RARRES2 | 0.056 | 4.00E-04 | 0.029 | 0.020 | 0.001 | 0.057 | 0.044 |
| REN | 0.060 | 4.00E-04 | 0.034 | 0.024 | 0.001 | 0.057 | 0.044 |
| SCGB1A1 | 0.063 | 8.00E-04 | 0.047 | 0.036 | 0.002 | 0.060 | 0.044 |
| SCGN | 0.037 | 0.004 | 0.026 | 0.024 | 0.006 | 0.057 | 0.044 |
| SIGLEC1 | 0.045 | 0.004 | 0.040 | 0.032 | 0.007 | 0.058 | 0.044 |
| SIT1 | 0.025 | 0.027 | 0.024 | 0.021 | 0.027 | 0.057 | 0.044 |
| SMPD1 | 0.039 | 0.003 | 0.019 | 0.015 | 0.006 | 0.057 | 0.044 |
| SOD2 | 0.035 | 0.009 | 0.037 | 0.033 | 0.012 | 0.057 | 0.044 |
| TFF2 | 0.055 | 0.004 | 0.033 | 0.024 | 0.006 | 0.057 | 0.044 |
| TFF3 | 0.035 | 0.003 | 0.023 | 0.020 | 0.006 | 0.057 | 0.044 |
| TNC | 0.035 | 0.005 | 0.028 | 0.023 | 0.007 | 0.057 | 0.044 |
| TNF | 0.038 | 4.00E-04 | 0.026 | 0.022 | 0.001 | 0.057 | 0.044 |
| TNFRSF10A | 0.048 | 0.013 | 0.036 | 0.028 | 0.016 | 0.057 | 0.044 |
| TNFRSF10B | 0.057 | <0.001 | 0.042 | 0.032 | <0.001 | 0.060 | 0.044 |
| TNFRSF12A | 0.035 | 0.008 | 0.033 | 0.029 | 0.011 | 0.057 | 0.044 |
| TNFRSF1B | 0.038 | 0.015 | 0.039 | 0.032 | 0.017 | 0.057 | 0.044 |
| TNFSF11 | 0.070 | 4.00E-04 | 0.026 | 0.017 | 0.001 | 0.057 | 0.044 |
| TNFSF13B | 0.030 | 0.016 | 0.021 | 0.019 | 0.018 | 0.057 | 0.044 |
| TNR | 0.050 | 0.003 | 0.034 | 0.025 | 0.006 | 0.057 | 0.044 |
| TPP1 | 0.037 | 0.002 | 0.035 | 0.029 | 0.005 | 0.057 | 0.044 |
| WFDC2 | 0.061 | 8.00E-04 | 0.054 | 0.040 | 0.002 | 0.064 | 0.047 |

| **Supplementary Table 24. Functional enrichment analysis of mediator proteins for the association between workplace noise exposure and anxiety disorder using the 2,920 proteins available for proteomic association testing as the assay-specific background.** | | | | | | | |
| --- | --- | --- | --- | --- | --- | --- | --- |
| **Category** | **Term ID** | **Term description** | **Observed gene count** | **Background gene count** | **Strength** | **Signal** | **FDR** |
| GO Component | GO:0016021 | Integral component of membrane | 35 | 890 | 0.29 | 0.34 | 0.004 |
| GO Component | GO:0031224 | Intrinsic component of membrane | 36 | 957 | 0.27 | 0.34 | 0.004 |

| **Supplementary Table 25. Functional enrichment analysis of mediator proteins for the association between workplace noise exposure and MDD using the 2,920 proteins available for proteomic association testing as the assay-specific background.** | | | | | | | |
| --- | --- | --- | --- | --- | --- | --- | --- |
| **Category** | **Term ID** | **Term description** | **Observed gene count** | **Background gene count** | **Strength** | **Signal** | **FDR** |
| GO Component | GO:0005576 | Extracellular region | 64 | 1447 | 0.19 | 0.37 | 0.001 |
| GO Component | GO:0031982 | Vesicle | 52 | 1100 | 0.22 | 0.34 | 0.003 |
| GO Component | GO:0005615 | Extracellular space | 52 | 1195 | 0.18 | 0.27 | 0.022 |
| GO Component | GO:0016021 | Integral component of membrane | 43 | 890 | 0.23 | 0.28 | 0.022 |
| GO Component | GO:0031224 | Intrinsic component of membrane | 45 | 957 | 0.21 | 0.28 | 0.022 |
| GO Component | GO:0009986 | Cell surface | 25 | 421 | 0.32 | 0.27 | 0.046 |

| **Supplementary Table 26. Functional enrichment analysis of mediator proteins for the association between workplace noise exposure and anxiety disorder using the default *Homo sapiens* genome-wide background as a sensitivity analysis.** | | | | | | | |
| --- | --- | --- | --- | --- | --- | --- | --- |
| **Category** | **Term ID** | **Term description** | **Observed gene count** | **Background gene count** | **Strength** | **Signal** | **FDR** |
| GO Process | GO:0009605 | Response to external stimulus | 23 | 2355 | 0.52 | 0.52 | 0.001 |
| GO Process | GO:0045937 | Positive regulation of phosphate metabolic process | 15 | 912 | 0.75 | 0.66 | 0.001 |
| GO Process | GO:0001934 | Positive regulation of protein phosphorylation | 13 | 747 | 0.77 | 0.66 | 0.001 |
| GO Process | GO:0009967 | Positive regulation of signal transduction | 18 | 1525 | 0.6 | 0.56 | 0.001 |
| GO Process | GO:0001932 | Regulation of protein phosphorylation | 15 | 1108 | 0.66 | 0.58 | 0.001 |
| GO Process | GO:0043408 | Regulation of MAPK cascade | 12 | 668 | 0.79 | 0.64 | 0.001 |
| GO Process | GO:0031401 | Positive regulation of protein modification process | 14 | 1018 | 0.67 | 0.56 | 0.002 |
| GO Process | GO:0048584 | Positive regulation of response to stimulus | 20 | 2131 | 0.5 | 0.47 | 0.002 |
| GO Process | GO:0019220 | Regulation of phosphate metabolic process | 16 | 1405 | 0.59 | 0.51 | 0.003 |
| GO Process | GO:0002682 | Regulation of immune system process | 16 | 1438 | 0.58 | 0.5 | 0.003 |
| GO Process | GO:0070372 | Regulation of ERK1 and ERK2 cascade | 8 | 299 | 0.96 | 0.67 | 0.003 |
| GO Process | GO:0070374 | Positive regulation of ERK1 and ERK2 cascade | 7 | 213 | 1.05 | 0.69 | 0.003 |
| GO Process | GO:0043372 | Positive regulation of CD4-positive, alpha-beta T cell differentiation | 4 | 33 | 1.61 | 0.8 | 0.003 |
| GO Process | GO:0044419 | Biological process involved in interspecies interaction between organisms | 16 | 1490 | 0.56 | 0.48 | 0.004 |
| GO Process | GO:0006955 | Immune response | 15 | 1321 | 0.59 | 0.49 | 0.004 |
| GO Process | GO:0048583 | Regulation of response to stimulus | 27 | 3931 | 0.37 | 0.38 | 0.004 |
| GO Process | GO:0051247 | Positive regulation of protein metabolic process | 16 | 1512 | 0.56 | 0.47 | 0.004 |
| GO Process | GO:1902533 | Positive regulation of intracellular signal transduction | 13 | 997 | 0.65 | 0.52 | 0.004 |
| GO Process | GO:1902531 | Regulation of intracellular signal transduction | 17 | 1726 | 0.52 | 0.45 | 0.004 |
| GO Process | GO:0002703 | Regulation of leukocyte mediated immunity | 7 | 239 | 1 | 0.64 | 0.005 |
| GO Process | GO:0001817 | Regulation of cytokine production | 11 | 739 | 0.7 | 0.52 | 0.006 |
| GO Process | GO:0002694 | Regulation of leukocyte activation | 10 | 601 | 0.75 | 0.53 | 0.006 |
| GO Process | GO:0043410 | Positive regulation of MAPK cascade | 9 | 481 | 0.8 | 0.54 | 0.006 |
| GO Process | GO:0007166 | Cell surface receptor signaling pathway | 18 | 2040 | 0.48 | 0.41 | 0.007 |
| GO Process | GO:0002706 | Regulation of lymphocyte mediated immunity | 6 | 176 | 1.06 | 0.61 | 0.008 |
| GO Process | GO:0050863 | Regulation of T cell activation | 8 | 376 | 0.86 | 0.55 | 0.008 |
| GO Process | GO:0002822 | Regulation of adaptive immune response based on somatic recombination of immune receptors built from immunoglobulin superfamily domains | 6 | 179 | 1.06 | 0.6 | 0.008 |
| GO Process | GO:0070663 | Regulation of leukocyte proliferation | 7 | 271 | 0.94 | 0.57 | 0.008 |
| GO Process | GO:0002696 | Positive regulation of leukocyte activation | 8 | 384 | 0.85 | 0.54 | 0.008 |
| GO Process | GO:0002697 | Regulation of immune effector process | 8 | 383 | 0.85 | 0.54 | 0.008 |
| GO Process | GO:0046718 | Viral entry into host cell | 5 | 107 | 1.2 | 0.63 | 0.008 |
| GO Process | GO:1902107 | Positive regulation of leukocyte differentiation | 6 | 181 | 1.05 | 0.6 | 0.008 |
| GO Process | GO:0032101 | Regulation of response to external stimulus | 12 | 964 | 0.63 | 0.46 | 0.008 |
| GO Process | GO:0042129 | Regulation of T cell proliferation | 6 | 187 | 1.04 | 0.59 | 0.008 |
| GO Process | GO:0009617 | Response to bacterium | 10 | 663 | 0.71 | 0.49 | 0.008 |
| GO Process | GO:0002376 | Immune system process | 18 | 2121 | 0.46 | 0.39 | 0.008 |
| GO Process | GO:0032147 | Activation of protein kinase activity | 6 | 203 | 1 | 0.56 | 0.010 |
| GO Process | GO:0009607 | Response to biotic stimulus | 14 | 1375 | 0.54 | 0.41 | 0.011 |
| GO Process | GO:0032720 | Negative regulation of tumor necrosis factor production | 4 | 61 | 1.35 | 0.61 | 0.012 |
| GO Process | GO:0032496 | Response to lipopolysaccharide | 7 | 314 | 0.88 | 0.51 | 0.012 |
| GO Process | GO:0006952 | Defense response | 14 | 1394 | 0.53 | 0.4 | 0.012 |
| GO Process | GO:0002684 | Positive regulation of immune system process | 11 | 874 | 0.63 | 0.44 | 0.012 |
| GO Process | GO:0007165 | Signal transduction | 28 | 4714 | 0.3 | 0.31 | 0.013 |
| GO Process | GO:0022409 | Positive regulation of cell-cell adhesion | 7 | 322 | 0.87 | 0.5 | 0.013 |
| GO Process | GO:0032103 | Positive regulation of response to external stimulus | 8 | 453 | 0.78 | 0.47 | 0.014 |
| GO Process | GO:1901224 | Positive regulation of NIK/NF-kappaB signaling | 4 | 67 | 1.31 | 0.57 | 0.014 |
| GO Process | GO:0009966 | Regulation of signal transduction | 21 | 2978 | 0.38 | 0.33 | 0.016 |
| GO Process | GO:0051239 | Regulation of multicellular organismal process | 20 | 2749 | 0.39 | 0.34 | 0.017 |
| GO Process | GO:0001894 | Tissue homeostasis | 6 | 238 | 0.93 | 0.49 | 0.018 |
| GO Process | GO:0002519 | Natural killer cell tolerance induction | 2 | 3 | 2.35 | 0.59 | 0.019 |
| GO Process | GO:0022407 | Regulation of cell-cell adhesion | 8 | 480 | 0.75 | 0.44 | 0.019 |
| GO Process | GO:0034103 | Regulation of tissue remodeling | 4 | 75 | 1.26 | 0.54 | 0.019 |
| GO Process | GO:0042130 | Negative regulation of T cell proliferation | 4 | 75 | 1.26 | 0.54 | 0.019 |
| GO Process | GO:0045785 | Positive regulation of cell adhesion | 8 | 485 | 0.75 | 0.44 | 0.019 |
| GO Process | GO:0007154 | Cell communication | 29 | 5165 | 0.28 | 0.29 | 0.019 |
| GO Process | GO:0031347 | Regulation of defense response | 9 | 638 | 0.68 | 0.41 | 0.021 |
| GO Process | GO:0033674 | Positive regulation of kinase activity | 8 | 494 | 0.74 | 0.43 | 0.021 |
| GO Process | GO:0050870 | Positive regulation of T cell activation | 6 | 251 | 0.91 | 0.47 | 0.021 |
| GO Process | GO:0051707 | Response to other organism | 13 | 1328 | 0.52 | 0.37 | 0.021 |
| GO Process | GO:1903037 | Regulation of leukocyte cell-cell adhesion | 7 | 368 | 0.81 | 0.45 | 0.021 |
| GO Process | GO:0048871 | Multicellular organismal homeostasis | 7 | 372 | 0.81 | 0.44 | 0.021 |
| GO Process | GO:0051246 | Regulation of protein metabolic process | 19 | 2622 | 0.39 | 0.32 | 0.022 |
| GO Process | GO:0002825 | Regulation of T-helper 1 type immune response | 3 | 31 | 1.52 | 0.53 | 0.024 |
| GO Process | GO:0051048 | Negative regulation of secretion | 5 | 164 | 1.02 | 0.47 | 0.024 |
| GO Process | GO:0033993 | Response to lipid | 10 | 827 | 0.61 | 0.38 | 0.025 |
| GO Process | GO:0070665 | Positive regulation of leukocyte proliferation | 5 | 167 | 1.01 | 0.47 | 0.025 |
| GO Process | GO:0048522 | Positive regulation of cellular process | 30 | 5584 | 0.26 | 0.27 | 0.026 |
| GO Process | GO:0080134 | Regulation of response to stress | 13 | 1373 | 0.51 | 0.35 | 0.026 |
| GO Process | GO:0048518 | Positive regulation of biological process | 32 | 6207 | 0.24 | 0.27 | 0.027 |
| GO Process | GO:0032673 | Regulation of interleukin-4 production | 3 | 34 | 1.48 | 0.5 | 0.028 |
| GO Process | GO:0150077 | Regulation of neuroinflammatory response | 3 | 34 | 1.48 | 0.5 | 0.028 |
| GO Process | GO:0001818 | Negative regulation of cytokine production | 6 | 284 | 0.86 | 0.42 | 0.031 |
| GO Process | GO:0038061 | NIK/NF-kappaB signaling | 3 | 36 | 1.45 | 0.49 | 0.031 |
| GO Process | GO:0051241 | Negative regulation of multicellular organismal process | 11 | 1035 | 0.56 | 0.35 | 0.031 |
| GO Process | GO:0032642 | Regulation of chemokine production | 4 | 96 | 1.15 | 0.46 | 0.031 |
| GO Process | GO:0045860 | Positive regulation of protein kinase activity | 7 | 416 | 0.76 | 0.39 | 0.034 |
| GO Process | GO:2001188 | Regulation of T cell activation via T cell receptor contact with antigen bound to MHC molecule on antigen presenting cell | 2 | 6 | 2.05 | 0.5 | 0.034 |
| GO Process | GO:0032733 | Positive regulation of interleukin-10 production | 3 | 40 | 1.41 | 0.46 | 0.038 |
|  |  |  |  |  |  |  |  |
| GO Process | GO:0042127 | Regulation of cell population proliferation | 14 | 1669 | 0.45 | 0.31 | 0.039 |
| GO Process | GO:0036462 | TRAIL-activated apoptotic signaling pathway | 2 | 7 | 1.99 | 0.47 | 0.041 |
| GO Process | GO:0042742 | Defense response to bacterium | 6 | 306 | 0.82 | 0.39 | 0.041 |
| GO Process | GO:0042102 | Positive regulation of T cell proliferation | 4 | 108 | 1.1 | 0.42 | 0.044 |
| GO Process | GO:0030334 | Regulation of cell migration | 10 | 927 | 0.56 | 0.32 | 0.048 |
| GO Process | GO:0002858 | Regulation of natural killer cell mediated cytotoxicity directed against tumor cell target | 2 | 8 | 1.93 | 0.44 | 0.049 |
| GO Process | GO:0006950 | Response to stress | 21 | 3358 | 0.33 | 0.27 | 0.049 |
| GO Process | GO:0016139 | Glycoside catabolic process | 2 | 8 | 1.93 | 0.44 | 0.049 |
| GO Process | GO:0071219 | Cellular response to molecule of bacterial origin | 5 | 205 | 0.92 | 0.38 | 0.049 |
| GO Function | GO:0005035 | Death receptor activity | 3 | 13 | 1.89 | 0.55 | 0.024 |
| GO Function | GO:0005102 | Signaling receptor binding | 16 | 1499 | 0.56 | 0.37 | 0.024 |
| GO Function | GO:0016936 | Galactoside binding | 3 | 9 | 2.05 | 0.55 | 0.024 |
| GO Component | GO:0005576 | Extracellular region | 41 | 4175 | 0.52 | 0.8 | 2.13E-12 |
| GO Component | GO:0005615 | Extracellular space | 34 | 3247 | 0.55 | 0.8 | 5.40E-10 |
| GO Component | GO:0009986 | Cell surface | 18 | 894 | 0.83 | 1.14 | 4.11E-08 |
| GO Component | GO:0071944 | Cell periphery | 41 | 6015 | 0.36 | 0.54 | 2.01E-07 |
| GO Component | GO:0005886 | Plasma membrane | 38 | 5544 | 0.37 | 0.52 | 1.61E-06 |
| GO Component | GO:0031224 | Intrinsic component of membrane | 36 | 5828 | 0.32 | 0.43 | < 0.001 |
| GO Component | GO:0031982 | Vesicle | 29 | 3957 | 0.4 | 0.48 | < 0.001 |
| GO Component | GO:0016021 | Integral component of membrane | 35 | 5670 | 0.32 | 0.43 | < 0.001 |
| GO Component | GO:0070062 | Extracellular exosome | 20 | 2096 | 0.51 | 0.54 | < 0.001 |
| GO Component | GO:0043202 | Lysosomal lumen | 5 | 97 | 1.24 | 0.8 | 0.002 |
| GO Component | GO:0005775 | Vacuolar lumen | 6 | 175 | 1.07 | 0.73 | 0.002 |
| GO Component | GO:0030141 | Secretory granule | 11 | 873 | 0.63 | 0.48 | 0.006 |
| GO Component | GO:0031226 | Intrinsic component of plasma membrane | 16 | 1788 | 0.48 | 0.42 | 0.006 |
| GO Component | GO:0045121 | Membrane raft | 7 | 320 | 0.87 | 0.57 | 0.006 |
| GO Component | GO:0005764 | Lysosome | 10 | 746 | 0.66 | 0.48 | 0.007 |
| GO Component | GO:0005887 | Integral component of plasma membrane | 15 | 1706 | 0.48 | 0.39 | 0.009 |
| GO Component | GO:0042582 | Azurophil granule | 5 | 154 | 1.04 | 0.57 | 0.010 |
| GO Component | GO:0016020 | Membrane | 42 | 9523 | 0.18 | 0.26 | 0.014 |
| GO Component | GO:0031410 | Cytoplasmic vesicle | 18 | 2482 | 0.39 | 0.34 | 0.015 |
| KEGG | hsa04060 | Cytokine-cytokine receptor interaction | 8 | 282 | 0.98 | 0.79 | 0.001 |
| KEGG | hsa04142 | Lysosome | 6 | 125 | 1.21 | 0.89 | 0.001 |
| KEGG | hsa04061 | Viral protein interaction with cytokine and cytokine receptor | 4 | 96 | 1.15 | 0.49 | 0.024 |
| Reactome | HSA-168256 | Immune System | 19 | 1979 | 0.51 | 0.44 | 0.005 |

| **Supplementary Table 27. Functional enrichment analysis of mediator proteins for the association between workplace noise exposure and MDD using the default *Homo sapiens* genome-wide background as a sensitivity analysis.** | | | | | | | |
| --- | --- | --- | --- | --- | --- | --- | --- |
| **Category** | **Term ID** | **Term description** | **Observed gene count** | **Background gene count** | **Strength** | **Signal** | **FDR** |
| GO Process | GO:0006955 | Immune response | 23 | 1321 | 0.62 | 0.71 | 6.31E-05 |
| GO Process | GO:0030155 | Regulation of cell adhesion | 18 | 784 | 0.74 | 0.8 | 6.31E-05 |
| GO Process | GO:0034097 | Response to cytokine | 18 | 804 | 0.73 | 0.79 | 6.31E-05 |
| GO Process | GO:0007155 | Cell adhesion | 19 | 965 | 0.67 | 0.75 | 6.55E-05 |
| GO Process | GO:0022407 | Regulation of cell-cell adhesion | 14 | 480 | 0.84 | 0.87 | 6.55E-05 |
| GO Process | GO:0002694 | Regulation of leukocyte activation | 15 | 601 | 0.77 | 0.8 | 9.51E-05 |
| GO Process | GO:0009605 | Response to external stimulus | 29 | 2355 | 0.47 | 0.58 | 1.10E-04 |
| GO Process | GO:0042221 | Response to chemical | 39 | 4010 | 0.36 | 0.51 | 1.10E-04 |
| GO Process | GO:0050863 | Regulation of T cell activation | 12 | 376 | 0.88 | 0.85 | 1.30E-04 |
| GO Process | GO:0032496 | Response to lipopolysaccharide | 11 | 314 | 0.92 | 0.86 | 1.60E-04 |
| GO Process | GO:0050896 | Response to stimulus | 57 | 7835 | 0.24 | 0.42 | 1.60E-04 |
| GO Process | GO:0043408 | Regulation of MAPK cascade | 15 | 668 | 0.73 | 0.74 | 1.70E-04 |
| GO Process | GO:0045785 | Positive regulation of cell adhesion | 13 | 485 | 0.8 | 0.79 | 1.70E-04 |
| GO Process | GO:0044419 | Biological process involved in interspecies interaction between organisms | 22 | 1490 | 0.54 | 0.62 | 1.80E-04 |
| GO Process | GO:0034612 | Response to tumor necrosis factor | 9 | 199 | 1.03 | 0.9 | 2.00E-04 |
| GO Process | GO:0006950 | Response to stress | 34 | 3358 | 0.38 | 0.5 | 2.20E-04 |
| GO Process | GO:0010033 | Response to organic substance | 30 | 2692 | 0.42 | 0.53 | 2.20E-04 |
| GO Process | GO:0045937 | Positive regulation of phosphate metabolic process | 17 | 912 | 0.65 | 0.68 | 2.20E-04 |
| GO Process | GO:0002376 | Immune system process | 26 | 2121 | 0.46 | 0.55 | 2.60E-04 |
| GO Process | GO:0048583 | Regulation of response to stimulus | 37 | 3931 | 0.35 | 0.48 | 2.60E-04 |
| GO Process | GO:0002682 | Regulation of immune system process | 21 | 1438 | 0.54 | 0.6 | 2.80E-04 |
| GO Process | GO:0006954 | Inflammatory response | 13 | 538 | 0.76 | 0.73 | 2.90E-04 |
| GO Process | GO:0046718 | Viral entry into host cell | 7 | 107 | 1.19 | 0.93 | 3.10E-04 |
| GO Process | GO:1903037 | Regulation of leukocyte cell-cell adhesion | 11 | 368 | 0.85 | 0.78 | 3.10E-04 |
| GO Process | GO:0001934 | Positive regulation of protein phosphorylation | 15 | 747 | 0.68 | 0.68 | 3.20E-04 |
| GO Process | GO:0009617 | Response to bacterium | 14 | 663 | 0.7 | 0.68 | 4.10E-04 |
| GO Process | GO:0010951 | Negative regulation of endopeptidase activity | 9 | 240 | 0.95 | 0.79 | 0.001 |
| GO Process | GO:0071356 | Cellular response to tumor necrosis factor | 8 | 175 | 1.04 | 0.82 | 0.001 |
| GO Process | GO:0006952 | Defense response | 20 | 1394 | 0.53 | 0.57 | 0.001 |
| GO Process | GO:0022409 | Positive regulation of cell-cell adhesion | 10 | 322 | 0.87 | 0.74 | 0.001 |
| GO Process | GO:0042129 | Regulation of T cell proliferation | 8 | 187 | 1.01 | 0.79 | 0.001 |
| GO Process | GO:0071345 | Cellular response to cytokine stimulus | 14 | 711 | 0.67 | 0.63 | 0.001 |
| GO Process | GO:0033993 | Response to lipid | 15 | 827 | 0.63 | 0.61 | 0.001 |
| GO Process | GO:0007166 | Cell surface receptor signaling pathway | 24 | 2040 | 0.45 | 0.5 | 0.001 |
| GO Process | GO:0032101 | Regulation of response to external stimulus | 16 | 964 | 0.6 | 0.58 | 0.001 |
| GO Process | GO:0070663 | Regulation of leukocyte proliferation | 9 | 271 | 0.9 | 0.72 | 0.001 |
| GO Process | GO:1903039 | Positive regulation of leukocyte cell-cell adhesion | 9 | 274 | 0.89 | 0.71 | 0.001 |
| GO Process | GO:0070374 | Positive regulation of ERK1 and ERK2 cascade | 8 | 213 | 0.95 | 0.71 | 0.001 |
| GO Process | GO:0051716 | Cellular response to stimulus | 47 | 6357 | 0.24 | 0.38 | 0.002 |
| GO Process | GO:0031401 | Positive regulation of protein modification process | 16 | 1018 | 0.57 | 0.54 | 0.002 |
| GO Process | GO:0044092 | Negative regulation of molecular function | 17 | 1143 | 0.55 | 0.52 | 0.002 |
| GO Process | GO:0002696 | Positive regulation of leukocyte activation | 10 | 384 | 0.79 | 0.63 | 0.002 |
| GO Process | GO:0070372 | Regulation of ERK1 and ERK2 cascade | 9 | 299 | 0.85 | 0.65 | 0.002 |
| GO Process | GO:0043410 | Positive regulation of MAPK cascade | 11 | 481 | 0.73 | 0.6 | 0.002 |
| GO Process | GO:0030334 | Regulation of cell migration | 15 | 927 | 0.58 | 0.53 | 0.002 |
| GO Process | GO:1902531 | Regulation of intracellular signal transduction | 21 | 1726 | 0.46 | 0.47 | 0.002 |
| GO Process | GO:0052548 | Regulation of endopeptidase activity | 10 | 414 | 0.76 | 0.58 | 0.003 |
| GO Process | GO:0050870 | Positive regulation of T cell activation | 8 | 251 | 0.88 | 0.61 | 0.003 |
| GO Process | GO:1901224 | Positive regulation of NIK/NF-kappaB signaling | 5 | 67 | 1.25 | 0.7 | 0.003 |
| GO Process | GO:2000272 | Negative regulation of signaling receptor activity | 5 | 67 | 1.25 | 0.7 | 0.003 |
| GO Process | GO:0001932 | Regulation of protein phosphorylation | 16 | 1108 | 0.53 | 0.48 | 0.004 |
| GO Process | GO:0006026 | Aminoglycan catabolic process | 4 | 32 | 1.47 | 0.72 | 0.004 |
| GO Process | GO:0007154 | Cell communication | 40 | 5165 | 0.26 | 0.36 | 0.004 |
| GO Process | GO:0009607 | Response to biotic stimulus | 18 | 1375 | 0.49 | 0.46 | 0.004 |
| GO Process | GO:0009967 | Positive regulation of signal transduction | 19 | 1525 | 0.47 | 0.45 | 0.004 |
| GO Process | GO:0030335 | Positive regulation of cell migration | 11 | 529 | 0.69 | 0.54 | 0.004 |
| GO Process | GO:0042127 | Regulation of cell population proliferation | 20 | 1669 | 0.45 | 0.45 | 0.004 |
| GO Process | GO:0044403 | Biological process involved in symbiotic interaction | 8 | 257 | 0.87 | 0.6 | 0.004 |
| GO Process | GO:0080134 | Regulation of response to stress | 18 | 1373 | 0.49 | 0.46 | 0.004 |
| GO Process | GO:1902533 | Positive regulation of intracellular signal transduction | 15 | 997 | 0.55 | 0.48 | 0.004 |
| GO Process | GO:0032673 | Regulation of interleukin-4 production | 4 | 34 | 1.45 | 0.71 | 0.004 |
| GO Process | GO:0098609 | Cell-cell adhesion | 11 | 542 | 0.68 | 0.53 | 0.004 |
| GO Process | GO:0010647 | Positive regulation of cell communication | 20 | 1693 | 0.45 | 0.44 | 0.004 |
| GO Process | GO:1901700 | Response to oxygen-containing compound | 19 | 1547 | 0.46 | 0.45 | 0.004 |
| GO Process | GO:0023056 | Positive regulation of signaling | 20 | 1698 | 0.45 | 0.44 | 0.004 |
| GO Process | GO:0034103 | Regulation of tissue remodeling | 5 | 75 | 1.2 | 0.67 | 0.004 |
| GO Process | GO:0023052 | Signaling | 39 | 5057 | 0.26 | 0.36 | 0.004 |
| GO Process | GO:0038061 | NIK/NF-kappaB signaling | 4 | 36 | 1.42 | 0.69 | 0.004 |
| GO Process | GO:0071310 | Cellular response to organic substance | 22 | 2019 | 0.41 | 0.42 | 0.004 |
| GO Process | GO:0032147 | Activation of protein kinase activity | 7 | 203 | 0.91 | 0.58 | 0.005 |
| GO Process | GO:0007165 | Signal transduction | 37 | 4714 | 0.27 | 0.35 | 0.005 |
| GO Process | GO:0050727 | Regulation of inflammatory response | 9 | 371 | 0.76 | 0.54 | 0.005 |
| GO Process | GO:0051707 | Response to other organism | 17 | 1328 | 0.48 | 0.43 | 0.006 |
| GO Process | GO:0065009 | Regulation of molecular function | 28 | 3085 | 0.33 | 0.37 | 0.007 |
| GO Process | GO:0008284 | Positive regulation of cell population proliferation | 14 | 945 | 0.55 | 0.45 | 0.007 |
| GO Process | GO:0070887 | Cellular response to chemical stimulus | 25 | 2609 | 0.36 | 0.37 | 0.008 |
| GO Process | GO:0051247 | Positive regulation of protein metabolic process | 18 | 1512 | 0.45 | 0.4 | 0.008 |
| GO Process | GO:0048584 | Positive regulation of response to stimulus | 22 | 2131 | 0.39 | 0.38 | 0.009 |
| GO Process | GO:0051246 | Regulation of protein metabolic process | 25 | 2622 | 0.35 | 0.37 | 0.009 |
| GO Process | GO:0009966 | Regulation of signal transduction | 27 | 2978 | 0.33 | 0.36 | 0.009 |
| GO Process | GO:0010646 | Regulation of cell communication | 29 | 3355 | 0.31 | 0.34 | 0.010 |
| GO Process | GO:0023051 | Regulation of signaling | 29 | 3367 | 0.31 | 0.34 | 0.011 |
| GO Process | GO:0045860 | Positive regulation of protein kinase activity | 9 | 416 | 0.71 | 0.47 | 0.011 |
| GO Process | GO:0034112 | Positive regulation of homotypic cell-cell adhesion | 3 | 17 | 1.62 | 0.59 | 0.011 |
| GO Process | GO:0002703 | Regulation of leukocyte mediated immunity | 7 | 239 | 0.84 | 0.5 | 0.011 |
| GO Process | GO:0031347 | Regulation of defense response | 11 | 638 | 0.61 | 0.43 | 0.012 |
| GO Process | GO:0051896 | Regulation of protein kinase B signaling | 6 | 166 | 0.93 | 0.51 | 0.012 |
| GO Process | GO:0007250 | Activation of NF-kappaB-inducing kinase activity | 3 | 18 | 1.6 | 0.58 | 0.012 |
| GO Process | GO:0070665 | Positive regulation of leukocyte proliferation | 6 | 167 | 0.93 | 0.51 | 0.012 |
| GO Process | GO:0000302 | Response to reactive oxygen species | 6 | 169 | 0.93 | 0.5 | 0.013 |
| GO Process | GO:0007565 | Female pregnancy | 6 | 170 | 0.92 | 0.5 | 0.013 |
| GO Process | GO:0045780 | Positive regulation of bone resorption | 3 | 19 | 1.57 | 0.56 | 0.014 |
| GO Process | GO:0042102 | Positive regulation of T cell proliferation | 5 | 108 | 1.04 | 0.5 | 0.015 |
| GO Process | GO:0007159 | Leukocyte cell-cell adhesion | 4 | 56 | 1.23 | 0.52 | 0.015 |
| GO Process | GO:0033209 | Tumor necrosis factor-mediated signaling pathway | 4 | 56 | 1.23 | 0.52 | 0.015 |
| GO Process | GO:0048678 | Response to axon injury | 4 | 56 | 1.23 | 0.52 | 0.015 |
| GO Process | GO:0010469 | Regulation of signaling receptor activity | 6 | 179 | 0.9 | 0.47 | 0.016 |
| GO Process | GO:0051094 | Positive regulation of developmental process | 16 | 1332 | 0.45 | 0.37 | 0.017 |
| GO Process | GO:0032103 | Positive regulation of response to external stimulus | 9 | 453 | 0.67 | 0.42 | 0.017 |
| GO Process | GO:0032656 | Regulation of interleukin-13 production | 3 | 21 | 1.53 | 0.53 | 0.017 |
| GO Process | GO:0051897 | Positive regulation of protein kinase B signaling | 5 | 113 | 1.02 | 0.49 | 0.017 |
| GO Process | GO:0097191 | Extrinsic apoptotic signaling pathway | 5 | 113 | 1.02 | 0.49 | 0.017 |
| GO Process | GO:1902107 | Positive regulation of leukocyte differentiation | 6 | 181 | 0.9 | 0.47 | 0.017 |
| GO Process | GO:0002250 | Adaptive immune response | 8 | 359 | 0.72 | 0.43 | 0.018 |
| GO Process | GO:0045321 | Leukocyte activation | 10 | 574 | 0.62 | 0.4 | 0.019 |
| GO Process | GO:0002519 | Natural killer cell tolerance induction | 2 | 3 | 2.2 | 0.53 | 0.020 |
| GO Process | GO:0030207 | Chondroitin sulfate catabolic process | 2 | 3 | 2.2 | 0.53 | 0.020 |
| GO Process | GO:0034021 | Response to silicon dioxide | 2 | 3 | 2.2 | 0.53 | 0.020 |
| GO Process | GO:0019221 | Cytokine-mediated signaling pathway | 8 | 369 | 0.71 | 0.42 | 0.021 |
| GO Process | GO:0032649 | Regulation of interferon-gamma production | 5 | 120 | 1 | 0.46 | 0.021 |
| GO Process | GO:0048519 | Negative regulation of biological process | 38 | 5313 | 0.23 | 0.29 | 0.021 |
| GO Process | GO:0022408 | Negative regulation of cell-cell adhesion | 6 | 194 | 0.87 | 0.44 | 0.022 |
| GO Process | GO:0050731 | Positive regulation of peptidyl-tyrosine phosphorylation | 6 | 194 | 0.87 | 0.44 | 0.022 |
| GO Process | GO:0071222 | Cellular response to lipopolysaccharide | 6 | 195 | 0.86 | 0.44 | 0.022 |
| GO Process | GO:0070555 | Response to interleukin-1 | 5 | 125 | 0.98 | 0.45 | 0.024 |
| GO Process | GO:0001818 | Negative regulation of cytokine production | 7 | 284 | 0.77 | 0.41 | 0.024 |
| GO Process | GO:0006022 | Aminoglycan metabolic process | 5 | 126 | 0.97 | 0.44 | 0.024 |
| GO Process | GO:0034341 | Response to interferon-gamma | 5 | 127 | 0.97 | 0.44 | 0.025 |
| GO Process | GO:0006027 | Glycosaminoglycan catabolic process | 3 | 26 | 1.44 | 0.48 | 0.025 |
| GO Process | GO:0050790 | Regulation of catalytic activity | 22 | 2370 | 0.34 | 0.31 | 0.027 |
| GO Process | GO:0002675 | Positive regulation of acute inflammatory response | 3 | 27 | 1.42 | 0.47 | 0.027 |
| GO Process | GO:0014070 | Response to organic cyclic compound | 12 | 861 | 0.52 | 0.35 | 0.028 |
| GO Process | GO:0032722 | Positive regulation of chemokine production | 4 | 71 | 1.13 | 0.44 | 0.028 |
| GO Process | GO:0001817 | Regulation of cytokine production | 11 | 739 | 0.55 | 0.35 | 0.029 |
| GO Process | GO:0030162 | Regulation of proteolysis | 11 | 739 | 0.55 | 0.35 | 0.029 |
| GO Process | GO:0043065 | Positive regulation of apoptotic process | 9 | 507 | 0.62 | 0.37 | 0.031 |
| GO Process | GO:0002684 | Positive regulation of immune system process | 12 | 874 | 0.51 | 0.34 | 0.031 |
| GO Process | GO:0042130 | Negative regulation of T cell proliferation | 4 | 75 | 1.1 | 0.42 | 0.033 |
| GO Process | GO:0043154 | Negative regulation of cysteine-type endopeptidase activity involved in apoptotic process | 4 | 75 | 1.1 | 0.42 | 0.033 |
| GO Process | GO:0006935 | Chemotaxis | 9 | 516 | 0.62 | 0.36 | 0.034 |
| GO Process | GO:1903900 | Regulation of viral life cycle | 5 | 139 | 0.93 | 0.4 | 0.034 |
| GO Process | GO:0031102 | Neuron projection regeneration | 3 | 31 | 1.36 | 0.43 | 0.036 |
| GO Process | GO:0048660 | Regulation of smooth muscle cell proliferation | 5 | 142 | 0.92 | 0.4 | 0.036 |
| GO Process | GO:0032729 | Positive regulation of interferon-gamma production | 4 | 78 | 1.09 | 0.41 | 0.037 |
| GO Process | GO:0051241 | Negative regulation of multicellular organismal process | 13 | 1035 | 0.47 | 0.32 | 0.037 |
| GO Process | GO:0014068 | Positive regulation of phosphatidylinositol 3-kinase signaling | 4 | 79 | 1.08 | 0.41 | 0.038 |
| GO Process | GO:0043086 | Negative regulation of catalytic activity | 11 | 771 | 0.53 | 0.33 | 0.038 |
| GO Process | GO:0051384 | Response to glucocorticoid | 5 | 144 | 0.92 | 0.39 | 0.038 |
| GO Process | GO:0043372 | Positive regulation of CD4-positive, alpha-beta T cell differentiation | 3 | 33 | 1.33 | 0.41 | 0.042 |
| GO Process | GO:2001188 | Regulation of T cell activation via T cell receptor contact with antigen bound to MHC molecule on antigen presenting cell | 2 | 6 | 1.9 | 0.42 | 0.043 |
| GO Process | GO:0010941 | Regulation of cell death | 17 | 1651 | 0.39 | 0.29 | 0.043 |
| GO Process | GO:0002274 | Myeloid leukocyte activation | 5 | 150 | 0.9 | 0.37 | 0.043 |
| GO Process | GO:0150077 | Regulation of neuroinflammatory response | 3 | 34 | 1.32 | 0.41 | 0.043 |
| GO Process | GO:0002687 | Positive regulation of leukocyte migration | 5 | 151 | 0.9 | 0.37 | 0.044 |
| GO Process | GO:0045859 | Regulation of protein kinase activity | 10 | 663 | 0.55 | 0.32 | 0.044 |
| GO Process | GO:0002683 | Negative regulation of immune system process | 8 | 433 | 0.64 | 0.34 | 0.045 |
| GO Process | GO:0048518 | Positive regulation of biological process | 41 | 6207 | 0.2 | 0.25 | 0.045 |
| GO Process | GO:0050793 | Regulation of developmental process | 22 | 2492 | 0.32 | 0.28 | 0.045 |
| GO Function | GO:0005102 | Signaling receptor binding | 22 | 1499 | 0.54 | 0.55 | 0.001 |
| GO Function | GO:0030246 | Carbohydrate binding | 10 | 278 | 0.93 | 0.73 | 0.001 |
| GO Function | GO:0005126 | Cytokine receptor binding | 9 | 270 | 0.9 | 0.6 | 0.004 |
| GO Function | GO:0005125 | Cytokine activity | 8 | 234 | 0.91 | 0.52 | 0.010 |
| GO Function | GO:0001618 | Virus receptor activity | 5 | 76 | 1.19 | 0.48 | 0.022 |
| GO Function | GO:0005035 | Death receptor activity | 3 | 13 | 1.74 | 0.51 | 0.022 |
| GO Function | GO:0030545 | Signaling receptor regulator activity | 11 | 552 | 0.67 | 0.4 | 0.022 |
| GO Function | GO:0043394 | Proteoglycan binding | 4 | 37 | 1.41 | 0.49 | 0.022 |
| GO Function | GO:0048018 | Receptor ligand activity | 10 | 499 | 0.68 | 0.39 | 0.026 |
| GO Function | GO:0004866 | Endopeptidase inhibitor activity | 6 | 177 | 0.91 | 0.36 | 0.050 |
| GO Component | GO:0005576 | Extracellular region | 64 | 4175 | 0.56 | 1.14 | 6.46E-24 |
| GO Component | GO:0005615 | Extracellular space | 52 | 3247 | 0.58 | 1.12 | 6.58E-18 |
| GO Component | GO:0031982 | Vesicle | 52 | 3957 | 0.49 | 0.91 | 3.14E-14 |
| GO Component | GO:0009986 | Cell surface | 25 | 894 | 0.82 | 1.43 | 1.46E-11 |
| GO Component | GO:0070062 | Extracellular exosome | 34 | 2096 | 0.59 | 0.97 | 4.27E-10 |
| GO Component | GO:0071944 | Cell periphery | 56 | 6015 | 0.34 | 0.64 | 1.23E-09 |
| GO Component | GO:0031410 | Cytoplasmic vesicle | 32 | 2482 | 0.49 | 0.72 | 4.80E-07 |
| GO Component | GO:0005886 | Plasma membrane | 48 | 5544 | 0.31 | 0.53 | 2.76E-06 |
| GO Component | GO:0030141 | Secretory granule | 18 | 873 | 0.69 | 0.88 | 3.63E-06 |
| GO Component | GO:0031226 | Intrinsic component of plasma membrane | 25 | 1788 | 0.52 | 0.7 | 7.97E-06 |
| GO Component | GO:0005775 | Vacuolar lumen | 9 | 175 | 1.09 | 1.16 | 1.06E-05 |
| GO Component | GO:0005887 | Integral component of plasma membrane | 23 | 1706 | 0.51 | 0.63 | 5.01E-05 |
| GO Component | GO:0045121 | Membrane raft | 10 | 320 | 0.87 | 0.84 | 1.40E-04 |
| GO Component | GO:0031224 | Intrinsic component of membrane | 45 | 5828 | 0.26 | 0.43 | 2.60E-04 |
| GO Component | GO:0042582 | Azurophil granule | 7 | 154 | 1.03 | 0.83 | 5.00E-04 |
| GO Component | GO:0009897 | External side of plasma membrane | 10 | 388 | 0.79 | 0.71 | 0.001 |
| GO Component | GO:0016021 | Integral component of membrane | 43 | 5670 | 0.26 | 0.4 | 0.001 |
| GO Component | GO:0034774 | Secretory granule lumen | 9 | 321 | 0.82 | 0.7 | 0.001 |
| GO Component | GO:0005764 | Lysosome | 13 | 746 | 0.62 | 0.58 | 0.001 |
| GO Component | GO:0031012 | Extracellular matrix | 11 | 552 | 0.67 | 0.59 | 0.002 |
| GO Component | GO:0098637 | Protein complex involved in cell-matrix adhesion | 3 | 14 | 1.71 | 0.76 | 0.003 |
| GO Component | GO:0035578 | Azurophil granule lumen | 5 | 91 | 1.12 | 0.68 | 0.003 |
| GO Component | GO:0062023 | Collagen-containing extracellular matrix | 9 | 407 | 0.72 | 0.55 | 0.004 |
| GO Component | GO:0043202 | Lysosomal lumen | 5 | 97 | 1.09 | 0.65 | 0.004 |
| GO Component | GO:0098636 | Protein complex involved in cell adhesion | 4 | 52 | 1.26 | 0.65 | 0.005 |
| GO Component | GO:0090733 | Tenascin complex | 2 | 3 | 2.2 | 0.63 | 0.009 |
| GO Component | GO:0012505 | Endomembrane system | 35 | 4721 | 0.25 | 0.32 | 0.011 |
| GO Component | GO:0016020 | Membrane | 56 | 9523 | 0.14 | 0.27 | 0.017 |
| GO Component | GO:0098966 | Perisynaptic extracellular matrix | 2 | 6 | 1.9 | 0.5 | 0.023 |
| GO Component | GO:0099026 | Anchored component of presynaptic membrane | 2 | 7 | 1.83 | 0.48 | 0.029 |
| GO Component | GO:0042581 | Specific granule | 5 | 159 | 0.87 | 0.41 | 0.029 |
| GO Component | GO:0031225 | Anchored component of membrane | 5 | 171 | 0.84 | 0.38 | 0.039 |
| KEGG | hsa04060 | Cytokine-cytokine receptor interaction | 13 | 282 | 1.04 | 1.43 | 9.69E-08 |
| KEGG | hsa04142 | Lysosome | 7 | 125 | 1.12 | 0.93 | < 0.001 |
| KEGG | hsa05323 | Rheumatoid arthritis | 6 | 83 | 1.23 | 0.97 | < 0.001 |
| KEGG | hsa04061 | Viral protein interaction with cytokine and cytokine receptor | 6 | 96 | 1.17 | 0.9 | < 0.001 |
| KEGG | hsa05164 | Influenza A | 6 | 163 | 0.94 | 0.59 | 0.005 |
| KEGG | hsa00531 | Glycosaminoglycan degradation | 3 | 19 | 1.57 | 0.67 | 0.006 |
| KEGG | hsa05143 | African trypanosomiasis | 3 | 36 | 1.3 | 0.46 | 0.028 |
| KEGG | hsa04064 | NF-kappa B signaling pathway | 4 | 101 | 0.97 | 0.39 | 0.042 |
| KEGG | hsa05144 | Malaria | 3 | 46 | 1.19 | 0.4 | 0.043 |
| KEGG | hsa04668 | TNF signaling pathway | 4 | 111 | 0.93 | 0.37 | 0.047 |
| Reactome | HSA-168256 | Immune System | 27 | 1979 | 0.51 | 0.64 | 4.99E-05 |
| Reactome | HSA-1280215 | Cytokine Signaling in Immune system | 16 | 706 | 0.73 | 0.81 | 5.07E-05 |
| Reactome | HSA-6783783 | Interleukin-10 signaling | 6 | 45 | 1.5 | 1.21 | 5.35E-05 |
| Reactome | HSA-449147 | Signaling by Interleukins | 11 | 453 | 0.76 | 0.61 | 0.002 |
| Reactome | HSA-5668541 | TNFR2 non-canonical NF-kB pathway | 6 | 99 | 1.16 | 0.71 | 0.002 |
| Reactome | HSA-6798695 | Neutrophil degranulation | 11 | 476 | 0.74 | 0.59 | 0.002 |
| Reactome | HSA-5669034 | TNFs bind their physiological receptors | 4 | 28 | 1.53 | 0.74 | 0.003 |
| Reactome | HSA-2206281 | Mucopolysaccharidoses | 3 | 11 | 1.81 | 0.66 | 0.007 |
| Reactome | HSA-5676594 | TNF receptor superfamily (TNFSF) members mediating non-canonical NF-kB pathway | 3 | 17 | 1.62 | 0.52 | 0.020 |

| **Supplementary Table 28. Consistency of protein associations across primary, training, and testing analyses.** | | | | | |
| --- | --- | --- | --- | --- | --- |
| **Association** | **Analysis** | **N** | **Pearson r** | **Directional concordance (%)** | **Proteins remaining significant at FDR <0.05** |
| Protein-anxiety disorder | Training set (70%) | 11100 | 0.956 | 100 | 27/239 (11.3%) |
|  | Testing set (30%) | 4776 | 0.862 | 99.2 | 112/239 (46.9%) |
| Protein-MDD | Training set (70%) | 10959 | 0.971 | 100 | 231/343 (67.3%) |
|  | Testing set (30%) | 4721 | 0.836 | 96.2 | 4/343 (1.2%) |

| **Supplementary Table 29. Detailed protein associations with anxiety disorder in the primary, training, and testing analyses.** | | | | | | | | | |
| --- | --- | --- | --- | --- | --- | --- | --- | --- | --- |
| **Protein** | **Primary analysis** | | | **Training set** | | | **Testing set** | | |
|  | **HR (95% CI)** | **P** | **FDR** | **HR (95% CI)** | **P** | **FDR** | **HR (95% CI)** | **P** | **FDR** |
| ACE2 | 1.136 (1.057-1.220) | 4.95E-04 | 0.012 | 1.092 (1.000-1.193) | 0.051 | 0.460 | 1.230 (1.089-1.389) | 0.001 | 0.025 |
| ACVRL1 | 1.121 (1.045-1.204) | 0.002 | 0.027 | 1.107 (1.015-1.208) | 0.022 | 0.333 | 1.154 (1.021-1.304) | 0.022 | 0.146 |
| ADAM9 | 1.132 (1.054-1.216) | 0.001 | 0.014 | 1.083 (0.992-1.183) | 0.074 | 0.502 | 1.241 (1.097-1.403) | 0.001 | 0.020 |
| ADAMTSL2 | 1.143 (1.063-1.230) | 3.24E-04 | 0.009 | 1.093 (0.998-1.197) | 0.055 | 0.473 | 1.245 (1.102-1.406) | 4.10E-04 | 0.017 |
| ADGRG1 | 1.125 (1.053-1.201) | 4.57E-04 | 0.011 | 1.099 (1.014-1.191) | 0.022 | 0.332 | 1.170 (1.045-1.310) | 0.007 | 0.082 |
| ADM | 1.165 (1.075-1.263) | 2.13E-04 | 0.007 | 1.238 (1.121-1.367) | 2.55E-05 | 0.009 | 1.032 (0.898-1.186) | 0.658 | 0.807 |
| AGRN | 1.120 (1.047-1.197) | 0.001 | 0.019 | 1.068 (0.982-1.161) | 0.125 | 0.595 | 1.222 (1.094-1.366) | 3.92E-04 | 0.017 |
| ANGPT2 | 1.147 (1.073-1.225) | 5.22E-05 | 0.003 | 1.093 (1.008-1.186) | 0.032 | 0.388 | 1.263 (1.128-1.414) | 5.19E-05 | 0.008 |
| APOM | 0.903 (0.843-0.966) | 0.003 | 0.043 | 0.910 (0.838-0.987) | 0.023 | 0.336 | 0.889 (0.787-1.004) | 0.059 | 0.231 |
| AREG | 1.145 (1.079-1.216) | 9.05E-06 | 0.001 | 1.135 (1.055-1.222) | 0.001 | 0.067 | 1.169 (1.054-1.297) | 0.003 | 0.052 |
| ASAH1 | 1.117 (1.047-1.192) | 0.001 | 0.017 | 1.081 (0.995-1.175) | 0.066 | 0.495 | 1.172 (1.065-1.289) | 0.001 | 0.031 |
| ASGR1 | 1.185 (1.104-1.272) | 2.57E-06 | 3.57E-04 | 1.168 (1.071-1.273) | 4.40E-04 | 0.046 | 1.216 (1.074-1.376) | 0.002 | 0.042 |
| ATRAID | 1.130 (1.061-1.204) | 1.40E-04 | 0.005 | 1.135 (1.051-1.227) | 0.001 | 0.094 | 1.118 (1.002-1.248) | 0.046 | 0.211 |
| B2M | 1.131 (1.080-1.184) | 1.46E-07 | 5.31E-05 | 1.080 (1.010-1.155) | 0.024 | 0.337 | 1.283 (1.182-1.393) | 2.63E-09 | 3.84E-06 |
| B4GALT1 | 1.150 (1.077-1.228) | 2.73E-05 | 0.002 | 1.122 (1.036-1.214) | 0.005 | 0.164 | 1.216 (1.083-1.364) | 0.001 | 0.027 |
| BMPER | 1.125 (1.055-1.199) | 3.00E-04 | 0.009 | 1.081 (0.997-1.171) | 0.058 | 0.481 | 1.251 (1.108-1.411) | 2.82E-04 | 0.016 |
| BST2 | 1.128 (1.061-1.200) | 1.30E-04 | 0.005 | 1.092 (1.010-1.181) | 0.027 | 0.354 | 1.198 (1.084-1.323) | 3.74E-04 | 0.017 |
| BTN3A2 | 1.158 (1.083-1.239) | 1.97E-05 | 0.001 | 1.143 (1.053-1.240) | 0.001 | 0.094 | 1.179 (1.044-1.331) | 0.008 | 0.089 |
| C1QBP | 1.087 (1.028-1.150) | 0.004 | 0.045 | 1.108 (1.037-1.185) | 0.003 | 0.120 | 1.049 (0.945-1.164) | 0.368 | 0.587 |
| C7 | 1.117 (1.037-1.203) | 0.003 | 0.045 | 1.109 (1.013-1.214) | 0.025 | 0.350 | 1.133 (0.995-1.290) | 0.059 | 0.231 |
| CA14 | 0.857 (0.797-0.922) | 3.53E-05 | 0.002 | 0.852 (0.779-0.931) | 4.36E-04 | 0.046 | 0.857 (0.754-0.974) | 0.018 | 0.134 |
| CA6 | 0.883 (0.823-0.947) | 0.001 | 0.012 | 0.871 (0.799-0.949) | 0.002 | 0.095 | 0.900 (0.796-1.017) | 0.092 | 0.284 |
| CCER2 | 1.121 (1.046-1.202) | 0.001 | 0.022 | 1.137 (1.045-1.237) | 0.003 | 0.122 | 1.085 (0.961-1.225) | 0.188 | 0.408 |
| CCL15 | 1.100 (1.032-1.173) | 0.003 | 0.044 | 1.077 (0.995-1.165) | 0.065 | 0.493 | 1.145 (1.025-1.278) | 0.016 | 0.126 |
| CCL19 | 1.102 (1.039-1.169) | 0.001 | 0.022 | 1.072 (0.994-1.156) | 0.071 | 0.502 | 1.151 (1.049-1.263) | 0.003 | 0.050 |
| CD274 | 1.146 (1.078-1.220) | 1.49E-05 | 0.001 | 1.117 (1.035-1.206) | 0.005 | 0.163 | 1.214 (1.093-1.348) | 3.01E-04 | 0.016 |
| CD276 | 1.142 (1.067-1.223) | 1.35E-04 | 0.005 | 1.188 (1.092-1.292) | 5.93E-05 | 0.015 | 1.055 (0.939-1.186) | 0.366 | 0.586 |
| CD302 | 1.122 (1.048-1.202) | 0.001 | 0.020 | 1.103 (1.013-1.202) | 0.024 | 0.340 | 1.156 (1.031-1.295) | 0.013 | 0.113 |
| CD4 | 1.088 (1.031-1.149) | 0.002 | 0.034 | 1.077 (1.009-1.150) | 0.026 | 0.354 | 1.120 (1.006-1.247) | 0.038 | 0.192 |
| CD40 | 1.109 (1.040-1.182) | 0.002 | 0.028 | 1.085 (1.003-1.175) | 0.043 | 0.439 | 1.159 (1.039-1.293) | 0.008 | 0.091 |
| CD79B | 1.108 (1.036-1.184) | 0.003 | 0.038 | 1.057 (0.973-1.149) | 0.192 | 0.695 | 1.210 (1.087-1.347) | 4.84E-04 | 0.018 |
| CD80 | 1.144 (1.065-1.229) | 2.16E-04 | 0.007 | 1.114 (1.020-1.217) | 0.017 | 0.302 | 1.201 (1.066-1.352) | 0.003 | 0.046 |
| CD83 | 1.163 (1.087-1.244) | 1.15E-05 | 0.001 | 1.117 (1.027-1.215) | 0.010 | 0.228 | 1.251 (1.117-1.401) | 1.06E-04 | 0.010 |
| CD99L2 | 1.144 (1.069-1.224) | 1.07E-04 | 0.004 | 1.121 (1.032-1.218) | 0.007 | 0.202 | 1.193 (1.059-1.345) | 0.004 | 0.060 |
| CDCP1 | 1.117 (1.038-1.203) | 0.003 | 0.044 | 1.109 (1.014-1.214) | 0.023 | 0.336 | 1.134 (0.995-1.292) | 0.059 | 0.231 |
| CEACAM5 | 1.132 (1.058-1.211) | 3.46E-04 | 0.009 | 1.084 (0.998-1.177) | 0.054 | 0.473 | 1.253 (1.109-1.417) | 3.10E-04 | 0.016 |
| CEMIP2 | 1.100 (1.043-1.161) | 4.35E-04 | 0.011 | 1.082 (1.006-1.164) | 0.034 | 0.391 | 1.124 (1.042-1.214) | 0.003 | 0.047 |
| CES3 | 0.909 (0.854-0.968) | 0.003 | 0.040 | 0.883 (0.816-0.955) | 0.002 | 0.100 | 0.963 (0.868-1.069) | 0.481 | 0.677 |
| CHAD | 0.900 (0.838-0.966) | 0.003 | 0.044 | 0.849 (0.780-0.925) | 1.71E-04 | 0.033 | 1.009 (0.889-1.145) | 0.891 | 0.944 |
| CHCHD10 | 1.179 (1.098-1.267) | 6.49E-06 | 0.001 | 1.160 (1.061-1.268) | 0.001 | 0.084 | 1.218 (1.080-1.373) | 0.001 | 0.033 |
| CHCHD6 | 1.101 (1.042-1.164) | 0.001 | 0.015 | 1.074 (0.995-1.159) | 0.068 | 0.501 | 1.136 (1.053-1.226) | 0.001 | 0.028 |
| CHGA | 1.172 (1.101-1.247) | 7.09E-07 | < 0.001 | 1.194 (1.109-1.286) | 2.85E-06 | 0.001 | 1.126 (1.002-1.266) | 0.046 | 0.211 |
| CHRDL1 | 1.134 (1.052-1.223) | 0.001 | 0.021 | 1.132 (1.033-1.240) | 0.008 | 0.217 | 1.141 (0.997-1.305) | 0.056 | 0.226 |
| CKAP4 | 1.109 (1.038-1.185) | 0.002 | 0.032 | 1.110 (1.022-1.206) | 0.013 | 0.271 | 1.103 (0.990-1.230) | 0.076 | 0.262 |
| CLMP | 1.123 (1.038-1.215) | 0.004 | 0.047 | 1.120 (1.018-1.233) | 0.020 | 0.331 | 1.126 (0.982-1.292) | 0.089 | 0.279 |
| CLPP | 1.113 (1.041-1.189) | 0.002 | 0.027 | 1.096 (1.011-1.188) | 0.026 | 0.354 | 1.150 (1.023-1.291) | 0.019 | 0.136 |
| COL18A1 | 1.161 (1.081-1.246) | 4.28E-05 | 0.002 | 1.142 (1.047-1.246) | 0.003 | 0.122 | 1.190 (1.051-1.346) | 0.006 | 0.076 |
| COL4A1 | 1.139 (1.067-1.216) | 9.58E-05 | 0.004 | 1.127 (1.041-1.221) | 0.003 | 0.131 | 1.167 (1.042-1.307) | 0.008 | 0.087 |
| COL5A1 | 1.112 (1.037-1.193) | 0.003 | 0.042 | 1.040 (0.952-1.136) | 0.384 | 0.825 | 1.251 (1.119-1.398) | 7.81E-05 | 0.009 |
| COL6A3 | 1.140 (1.062-1.223) | 3.01E-04 | 0.009 | 1.089 (0.997-1.188) | 0.057 | 0.477 | 1.244 (1.105-1.400) | 2.96E-04 | 0.016 |
| COLEC12 | 1.131 (1.050-1.220) | 0.001 | 0.024 | 1.100 (1.003-1.207) | 0.043 | 0.439 | 1.193 (1.049-1.357) | 0.007 | 0.084 |
| CPM | 1.162 (1.082-1.248) | 3.98E-05 | 0.002 | 1.118 (1.025-1.219) | 0.012 | 0.256 | 1.263 (1.112-1.434) | 3.15E-04 | 0.016 |
| CREG1 | 1.107 (1.035-1.183) | 0.003 | 0.041 | 1.078 (0.993-1.171) | 0.071 | 0.502 | 1.177 (1.050-1.320) | 0.005 | 0.072 |
| CRELD1 | 1.165 (1.081-1.257) | 6.65E-05 | 0.003 | 1.148 (1.048-1.258) | 0.003 | 0.126 | 1.204 (1.055-1.374) | 0.006 | 0.076 |
| CRIM1 | 1.116 (1.046-1.191) | 0.001 | 0.018 | 1.095 (1.010-1.186) | 0.027 | 0.354 | 1.161 (1.039-1.296) | 0.008 | 0.091 |
| CRTAC1 | 0.881 (0.823-0.944) | 3.12E-04 | 0.009 | 0.876 (0.806-0.952) | 0.002 | 0.102 | 0.899 (0.797-1.013) | 0.081 | 0.268 |
| CSF1 | 1.148 (1.074-1.227) | 4.96E-05 | 0.002 | 1.101 (1.013-1.196) | 0.023 | 0.336 | 1.239 (1.110-1.382) | 1.31E-04 | 0.011 |
| CST3 | 1.113 (1.035-1.197) | 0.004 | 0.050 | 1.085 (0.992-1.186) | 0.075 | 0.502 | 1.171 (1.032-1.328) | 0.014 | 0.120 |
| CSTB | 1.132 (1.058-1.210) | 2.96E-04 | 0.009 | 1.102 (1.015-1.196) | 0.021 | 0.331 | 1.196 (1.064-1.343) | 0.003 | 0.046 |
| CTSF | 1.128 (1.054-1.207) | 4.93E-04 | 0.012 | 1.138 (1.048-1.236) | 0.002 | 0.104 | 1.109 (0.984-1.251) | 0.091 | 0.282 |
| CTSL | 1.127 (1.053-1.207) | 0.001 | 0.013 | 1.072 (0.986-1.167) | 0.104 | 0.553 | 1.232 (1.096-1.384) | 4.56E-04 | 0.017 |
| CTSZ | 1.108 (1.034-1.187) | 0.003 | 0.045 | 1.107 (1.018-1.204) | 0.017 | 0.306 | 1.111 (0.984-1.255) | 0.089 | 0.279 |
| CXADR | 1.182 (1.113-1.256) | 6.22E-08 | < 0.001 | 1.095 (1.013-1.183) | 0.022 | 0.335 | 1.342 (1.220-1.475) | 1.38E-09 | 3.84E-06 |
| CXCL17 | 1.146 (1.062-1.236) | 4.18E-04 | 0.011 | 1.104 (1.006-1.211) | 0.038 | 0.411 | 1.239 (1.085-1.414) | 0.002 | 0.036 |
| CXCL8 | 1.108 (1.040-1.180) | 0.002 | 0.027 | 1.058 (0.974-1.148) | 0.182 | 0.687 | 1.189 (1.081-1.308) | 3.60E-04 | 0.017 |
| DCBLD2 | 1.117 (1.043-1.196) | 0.002 | 0.026 | 1.043 (0.960-1.133) | 0.321 | 0.792 | 1.299 (1.151-1.466) | 2.17E-05 | 0.004 |
| DDAH1 | 1.106 (1.034-1.182) | 0.003 | 0.043 | 1.047 (0.963-1.137) | 0.281 | 0.773 | 1.224 (1.096-1.367) | 3.50E-04 | 0.017 |
| DEFB4A_DEFB4B | 1.167 (1.088-1.252) | 1.46E-05 | 0.001 | 1.147 (1.054-1.249) | 0.002 | 0.095 | 1.204 (1.065-1.362) | 0.003 | 0.051 |
| DPY30 | 1.122 (1.050-1.199) | 0.001 | 0.015 | 1.103 (1.017-1.196) | 0.018 | 0.313 | 1.164 (1.036-1.307) | 0.011 | 0.103 |
| DTNB | 1.131 (1.055-1.212) | 0.001 | 0.012 | 1.102 (1.010-1.203) | 0.029 | 0.371 | 1.188 (1.061-1.331) | 0.003 | 0.050 |
| ECHDC3 | 1.160 (1.084-1.241) | 1.77E-05 | 0.001 | 1.159 (1.068-1.257) | 4.10E-04 | 0.046 | 1.153 (1.022-1.301) | 0.021 | 0.144 |
| ECHS1 | 1.117 (1.037-1.202) | 0.003 | 0.044 | 1.087 (0.994-1.189) | 0.068 | 0.501 | 1.172 (1.030-1.332) | 0.016 | 0.126 |
| EDA2R | 1.159 (1.068-1.258) | 3.85E-04 | 0.010 | 1.124 (1.018-1.242) | 0.021 | 0.332 | 1.230 (1.069-1.417) | 0.004 | 0.061 |
| EDN1 | 1.122 (1.043-1.207) | 0.002 | 0.031 | 1.073 (0.979-1.175) | 0.130 | 0.601 | 1.210 (1.078-1.359) | 0.001 | 0.032 |
| EFCAB14 | 1.110 (1.049-1.175) | 3.04E-04 | 0.009 | 1.074 (0.997-1.157) | 0.060 | 0.481 | 1.201 (1.088-1.325) | 2.81E-04 | 0.016 |
| EFNA1 | 1.118 (1.044-1.196) | 0.001 | 0.025 | 1.079 (0.992-1.173) | 0.075 | 0.502 | 1.189 (1.057-1.338) | 0.004 | 0.060 |
| EFNA4 | 1.144 (1.068-1.225) | 1.29E-04 | 0.005 | 1.120 (1.030-1.218) | 0.008 | 0.220 | 1.191 (1.054-1.345) | 0.005 | 0.070 |
| EGFR | 0.874 (0.819-0.933) | 4.87E-05 | 0.002 | 0.891 (0.824-0.965) | 0.004 | 0.161 | 0.835 (0.743-0.937) | 0.002 | 0.043 |
| ELN | 1.186 (1.082-1.301) | 2.66E-04 | 0.008 | 1.168 (1.045-1.305) | 0.006 | 0.199 | 1.227 (1.040-1.447) | 0.015 | 0.124 |
| ENPP7 | 1.130 (1.056-1.210) | 4.11E-04 | 0.011 | 1.069 (0.984-1.162) | 0.112 | 0.572 | 1.269 (1.126-1.430) | 9.73E-05 | 0.009 |
| EPHA2 | 1.114 (1.040-1.193) | 0.002 | 0.032 | 1.078 (0.989-1.174) | 0.088 | 0.528 | 1.179 (1.055-1.318) | 0.004 | 0.059 |
| ESYT2 | 1.108 (1.034-1.187) | 0.004 | 0.045 | 1.083 (0.995-1.179) | 0.066 | 0.496 | 1.161 (1.031-1.306) | 0.013 | 0.116 |
| F11R | 1.105 (1.036-1.179) | 0.003 | 0.037 | 1.089 (1.006-1.179) | 0.036 | 0.403 | 1.142 (1.022-1.277) | 0.019 | 0.137 |
| FABP3 | 1.159 (1.076-1.248) | 1.03E-04 | 0.004 | 1.127 (1.028-1.235) | 0.011 | 0.246 | 1.236 (1.086-1.407) | 0.001 | 0.033 |
| FABP4 | 1.143 (1.048-1.246) | 0.003 | 0.038 | 1.104 (0.992-1.229) | 0.070 | 0.502 | 1.219 (1.051-1.413) | 0.009 | 0.093 |
| FAM3C | 1.136 (1.060-1.217) | 2.96E-04 | 0.009 | 1.109 (1.020-1.206) | 0.016 | 0.292 | 1.185 (1.050-1.336) | 0.006 | 0.076 |
| FAP | 0.879 (0.822-0.941) | 1.81E-04 | 0.006 | 0.817 (0.753-0.886) | 9.69E-07 | 0.001 | 1.032 (0.916-1.163) | 0.607 | 0.771 |
| FCAMR | 1.128 (1.045-1.218) | 0.002 | 0.032 | 1.049 (0.955-1.153) | 0.317 | 0.790 | 1.303 (1.142-1.486) | 8.38E-05 | 0.009 |
| FGF23 | 1.150 (1.072-1.233) | 9.14E-05 | 0.004 | 1.142 (1.048-1.243) | 0.002 | 0.110 | 1.166 (1.032-1.318) | 0.014 | 0.117 |
| FGFBP3 | 0.898 (0.836-0.964) | 0.003 | 0.041 | 0.870 (0.798-0.948) | 0.002 | 0.095 | 0.964 (0.851-1.092) | 0.564 | 0.740 |
| FGFR4 | 1.171 (1.093-1.254) | 6.34E-06 | 0.001 | 1.090 (1.000-1.187) | 0.049 | 0.453 | 1.331 (1.194-1.483) | 2.35E-07 | 1.37E-04 |
| FKBP4 | 1.125 (1.046-1.209) | 0.001 | 0.025 | 1.102 (1.009-1.203) | 0.031 | 0.380 | 1.176 (1.036-1.335) | 0.012 | 0.110 |
| FN1 | 1.130 (1.046-1.220) | 0.002 | 0.031 | 1.124 (1.024-1.234) | 0.014 | 0.277 | 1.140 (0.995-1.307) | 0.059 | 0.231 |
| FOLR1 | 1.108 (1.033-1.188) | 0.004 | 0.050 | 1.062 (0.974-1.158) | 0.172 | 0.682 | 1.214 (1.077-1.368) | 0.001 | 0.036 |
| FSTL1 | 1.131 (1.059-1.209) | 2.78E-04 | 0.009 | 1.080 (0.993-1.175) | 0.071 | 0.502 | 1.268 (1.121-1.434) | 1.55E-04 | 0.011 |
| FSTL3 | 1.178 (1.096-1.266) | 9.37E-06 | 0.001 | 1.175 (1.075-1.285) | 3.81E-04 | 0.046 | 1.181 (1.044-1.336) | 0.008 | 0.091 |
| GALNT10 | 1.110 (1.039-1.185) | 0.002 | 0.032 | 1.061 (0.979-1.151) | 0.151 | 0.644 | 1.204 (1.081-1.340) | 0.001 | 0.023 |
| GAST | 1.177 (1.102-1.257) | 1.09E-06 | < 0.001 | 1.160 (1.072-1.256) | 2.36E-04 | 0.038 | 1.231 (1.093-1.385) | 0.001 | 0.020 |
| GBP1 | 1.127 (1.048-1.213) | 0.001 | 0.024 | 1.062 (0.969-1.163) | 0.198 | 0.699 | 1.254 (1.111-1.417) | 2.67E-04 | 0.016 |
| GDF15 | 1.204 (1.135-1.277) | 6.48E-10 | < 0.001 | 1.190 (1.109-1.277) | 1.31E-06 | 0.001 | 1.235 (1.107-1.378) | 1.57E-04 | 0.011 |
| GFRA1 | 1.122 (1.045-1.206) | 0.002 | 0.027 | 1.085 (0.994-1.184) | 0.068 | 0.501 | 1.194 (1.053-1.353) | 0.006 | 0.074 |
| GLA | 1.120 (1.039-1.208) | 0.003 | 0.042 | 1.055 (0.963-1.156) | 0.248 | 0.744 | 1.276 (1.117-1.458) | 3.25E-04 | 0.017 |
| GPNMB | 1.124 (1.049-1.204) | 0.001 | 0.019 | 1.067 (0.982-1.160) | 0.128 | 0.596 | 1.238 (1.099-1.395) | 4.44E-04 | 0.017 |
| GRPEL1 | 1.170 (1.097-1.247) | 1.48E-06 | < 0.001 | 1.112 (1.026-1.205) | 0.010 | 0.238 | 1.269 (1.148-1.404) | 3.48E-06 | 0.001 |
| GUCA2A | 1.120 (1.047-1.198) | 0.001 | 0.019 | 1.117 (1.029-1.212) | 0.008 | 0.220 | 1.122 (0.998-1.262) | 0.054 | 0.221 |
| GUSB | 1.145 (1.065-1.231) | 2.56E-04 | 0.008 | 1.112 (1.017-1.215) | 0.020 | 0.327 | 1.214 (1.073-1.373) | 0.002 | 0.043 |
| HAVCR1 | 1.137 (1.058-1.222) | 4.49E-04 | 0.011 | 1.117 (1.023-1.220) | 0.013 | 0.271 | 1.188 (1.050-1.345) | 0.006 | 0.080 |
| HAVCR2 | 1.110 (1.035-1.189) | 0.003 | 0.044 | 1.067 (0.979-1.162) | 0.138 | 0.617 | 1.202 (1.067-1.354) | 0.002 | 0.045 |
| HGF | 1.119 (1.039-1.204) | 0.003 | 0.039 | 1.059 (0.968-1.159) | 0.213 | 0.707 | 1.256 (1.107-1.426) | 3.95E-04 | 0.017 |
| HPGDS | 0.883 (0.825-0.946) | 3.69E-04 | 0.010 | 0.836 (0.769-0.909) | 2.78E-05 | 0.009 | 0.987 (0.878-1.110) | 0.826 | 0.910 |
| HS6ST2 | 1.122 (1.043-1.207) | 0.002 | 0.031 | 1.174 (1.076-1.281) | 3.10E-04 | 0.045 | 1.022 (0.894-1.168) | 0.749 | 0.871 |
| IDUA | 1.105 (1.033-1.182) | 0.004 | 0.047 | 1.086 (1.000-1.179) | 0.049 | 0.453 | 1.146 (1.018-1.290) | 0.024 | 0.152 |
| IFI30 | 1.207 (1.117-1.304) | 1.79E-06 | < 0.001 | 1.136 (1.032-1.250) | 0.009 | 0.224 | 1.346 (1.185-1.530) | 5.06E-06 | 0.002 |
| IGF2R | 1.107 (1.033-1.185) | 0.004 | 0.047 | 1.095 (1.008-1.189) | 0.031 | 0.380 | 1.141 (1.009-1.289) | 0.035 | 0.185 |
| IGFBP4 | 1.206 (1.126-1.292) | 1.04E-07 | < 0.001 | 1.170 (1.075-1.273) | 2.79E-04 | 0.043 | 1.283 (1.137-1.449) | 5.50E-05 | 0.008 |
| IGFBP7 | 1.119 (1.046-1.197) | 0.001 | 0.021 | 1.062 (0.974-1.158) | 0.173 | 0.682 | 1.208 (1.096-1.331) | 1.36E-04 | 0.011 |
| IGFBPL1 | 1.115 (1.040-1.196) | 0.002 | 0.032 | 1.028 (0.942-1.122) | 0.539 | 0.893 | 1.289 (1.153-1.442) | 8.12E-06 | 0.002 |
| IGSF8 | 1.162 (1.090-1.239) | 4.46E-06 | 0.001 | 1.142 (1.057-1.234) | 0.001 | 0.067 | 1.200 (1.068-1.349) | 0.002 | 0.043 |
| IL10RB | 1.109 (1.041-1.183) | 0.001 | 0.026 | 1.063 (0.980-1.153) | 0.140 | 0.621 | 1.193 (1.081-1.316) | 4.44E-04 | 0.017 |
| IL12RB2 | 1.124 (1.053-1.200) | 4.24E-04 | 0.011 | 1.144 (1.059-1.237) | 0.001 | 0.066 | 1.081 (0.960-1.218) | 0.199 | 0.417 |
| IL15 | 1.108 (1.039-1.182) | 0.002 | 0.030 | 1.100 (1.016-1.192) | 0.018 | 0.313 | 1.136 (1.014-1.273) | 0.028 | 0.167 |
| IL18 | 1.103 (1.034-1.176) | 0.003 | 0.041 | 1.090 (1.007-1.181) | 0.033 | 0.391 | 1.128 (1.011-1.259) | 0.031 | 0.175 |
| IL1R1 | 1.108 (1.039-1.182) | 0.002 | 0.028 | 1.083 (1.001-1.172) | 0.048 | 0.453 | 1.160 (1.045-1.288) | 0.005 | 0.074 |
| IL1RL1 | 1.133 (1.054-1.218) | 0.001 | 0.015 | 1.100 (1.007-1.201) | 0.034 | 0.392 | 1.211 (1.066-1.374) | 0.003 | 0.052 |
| IL4R | 1.107 (1.038-1.180) | 0.002 | 0.031 | 1.053 (0.973-1.139) | 0.203 | 0.700 | 1.212 (1.089-1.348) | 4.36E-04 | 0.017 |
| IMMT | 1.163 (1.094-1.236) | 1.11E-06 | < 0.001 | 1.142 (1.056-1.236) | 0.001 | 0.076 | 1.203 (1.094-1.322) | 1.40E-04 | 0.011 |
| ITGA11 | 0.884 (0.826-0.945) | 3.17E-04 | 0.009 | 0.877 (0.808-0.951) | 0.001 | 0.095 | 0.908 (0.806-1.023) | 0.113 | 0.313 |
| ITGAM | 0.864 (0.807-0.926) | 3.18E-05 | 0.002 | 0.844 (0.777-0.917) | 6.08E-05 | 0.015 | 0.910 (0.804-1.028) | 0.131 | 0.338 |
| ITGAV | 0.863 (0.806-0.925) | 2.83E-05 | 0.002 | 0.819 (0.754-0.890) | 2.52E-06 | 0.001 | 0.967 (0.856-1.094) | 0.598 | 0.764 |
| ITGB2 | 0.899 (0.838-0.965) | 0.003 | 0.042 | 0.872 (0.801-0.950) | 0.002 | 0.098 | 0.955 (0.842-1.084) | 0.477 | 0.675 |
| ITGBL1 | 1.149 (1.068-1.235) | 1.73E-04 | 0.006 | 1.090 (0.996-1.192) | 0.060 | 0.481 | 1.285 (1.134-1.456) | 8.52E-05 | 0.009 |
| JAM3 | 1.121 (1.050-1.196) | 0.001 | 0.015 | 1.109 (1.024-1.202) | 0.011 | 0.246 | 1.138 (1.015-1.277) | 0.027 | 0.165 |
| LAIR1 | 1.117 (1.050-1.187) | 4.36E-04 | 0.011 | 1.135 (1.053-1.223) | 0.001 | 0.076 | 1.089 (0.977-1.214) | 0.124 | 0.329 |
| LAMB1 | 1.129 (1.049-1.214) | 0.001 | 0.022 | 1.074 (0.984-1.172) | 0.110 | 0.568 | 1.265 (1.107-1.445) | 0.001 | 0.020 |
| LAYN | 1.117 (1.042-1.197) | 0.002 | 0.029 | 1.079 (0.991-1.174) | 0.081 | 0.516 | 1.195 (1.061-1.347) | 0.003 | 0.054 |
| LETM1 | 1.154 (1.079-1.235) | 3.29E-05 | 0.002 | 1.110 (1.020-1.208) | 0.015 | 0.290 | 1.229 (1.100-1.372) | 2.64E-04 | 0.016 |
| LGALS1 | 1.134 (1.059-1.214) | 3.20E-04 | 0.009 | 1.105 (1.016-1.201) | 0.020 | 0.327 | 1.193 (1.059-1.344) | 0.004 | 0.059 |
| LGALS4 | 1.140 (1.065-1.221) | 1.60E-04 | 0.006 | 1.111 (1.023-1.208) | 0.013 | 0.267 | 1.202 (1.066-1.355) | 0.003 | 0.047 |
| LGALS9 | 1.159 (1.076-1.248) | 9.28E-05 | 0.004 | 1.106 (1.009-1.211) | 0.031 | 0.380 | 1.279 (1.127-1.450) | 1.30E-04 | 0.011 |
| LMNB2 | 1.165 (1.083-1.253) | 4.02E-05 | 0.002 | 1.154 (1.056-1.260) | 0.001 | 0.095 | 1.189 (1.042-1.357) | 0.010 | 0.102 |
| LONP1 | 1.125 (1.047-1.209) | 0.001 | 0.024 | 1.097 (1.005-1.197) | 0.038 | 0.411 | 1.179 (1.039-1.338) | 0.011 | 0.104 |
| LRG1 | 1.122 (1.042-1.208) | 0.002 | 0.033 | 1.096 (1.002-1.198) | 0.045 | 0.439 | 1.172 (1.031-1.332) | 0.015 | 0.123 |
| LTBR | 1.107 (1.036-1.184) | 0.003 | 0.038 | 1.085 (0.998-1.178) | 0.054 | 0.473 | 1.151 (1.031-1.285) | 0.012 | 0.109 |
| MAD1L1 | 1.114 (1.047-1.186) | 0.001 | 0.015 | 1.085 (1.001-1.176) | 0.047 | 0.446 | 1.179 (1.066-1.303) | 0.001 | 0.033 |
| MEP1B | 0.909 (0.852-0.970) | 0.004 | 0.050 | 0.881 (0.814-0.953) | 0.002 | 0.095 | 0.966 (0.861-1.084) | 0.554 | 0.735 |
| MERTK | 1.124 (1.051-1.202) | 0.001 | 0.015 | 1.087 (1.001-1.180) | 0.047 | 0.446 | 1.202 (1.072-1.349) | 0.002 | 0.037 |
| MMP7 | 1.159 (1.078-1.247) | 6.27E-05 | 0.003 | 1.132 (1.037-1.237) | 0.006 | 0.185 | 1.213 (1.068-1.378) | 0.003 | 0.050 |
| MRC1 | 1.136 (1.056-1.221) | 0.001 | 0.014 | 1.059 (0.966-1.161) | 0.223 | 0.720 | 1.270 (1.139-1.417) | 1.84E-05 | 0.004 |
| NAGPA | 1.138 (1.056-1.226) | 0.001 | 0.015 | 1.046 (0.957-1.144) | 0.321 | 0.791 | 1.372 (1.200-1.569) | 3.63E-06 | 0.001 |
| NCR3LG1 | 1.194 (1.110-1.285) | 1.94E-06 | < 0.001 | 1.153 (1.054-1.261) | 0.002 | 0.102 | 1.288 (1.136-1.462) | 8.45E-05 | 0.009 |
| NCS1 | 1.126 (1.047-1.210) | 0.001 | 0.023 | 1.078 (0.987-1.177) | 0.095 | 0.537 | 1.225 (1.079-1.390) | 0.002 | 0.037 |
| NECTIN2 | 1.201 (1.127-1.281) | 1.89E-08 | < 0.001 | 1.132 (1.044-1.228) | 0.003 | 0.121 | 1.329 (1.202-1.470) | 3.25E-08 | 3.16E-05 |
| NHLRC3 | 1.161 (1.076-1.253) | 1.25E-04 | 0.005 | 1.070 (0.976-1.172) | 0.149 | 0.638 | 1.399 (1.222-1.603) | 1.24E-06 | 0.001 |
| NPC2 | 1.151 (1.074-1.234) | 6.71E-05 | 0.003 | 1.138 (1.042-1.243) | 0.004 | 0.153 | 1.161 (1.042-1.294) | 0.007 | 0.084 |
| NPDC1 | 1.152 (1.080-1.229) | 1.64E-05 | 0.001 | 1.110 (1.023-1.205) | 0.012 | 0.263 | 1.229 (1.109-1.361) | 8.19E-05 | 0.009 |
| NPPC | 1.117 (1.036-1.204) | 0.004 | 0.050 | 1.095 (0.998-1.201) | 0.055 | 0.473 | 1.156 (1.014-1.317) | 0.030 | 0.172 |
| NT5C1A | 1.129 (1.060-1.203) | 1.84E-04 | 0.006 | 1.086 (1.003-1.175) | 0.043 | 0.439 | 1.243 (1.107-1.396) | 2.39E-04 | 0.016 |
| NTproBNP | 1.114 (1.038-1.194) | 0.003 | 0.038 | 1.104 (1.013-1.202) | 0.024 | 0.338 | 1.133 (1.003-1.281) | 0.045 | 0.208 |
| NTRK3 | 0.862 (0.804-0.923) | 2.29E-05 | 0.002 | 0.867 (0.797-0.942) | 0.001 | 0.067 | 0.851 (0.753-0.962) | 0.010 | 0.100 |
| OCLN | 1.202 (1.119-1.291) | 5.09E-07 | < 0.001 | 1.152 (1.054-1.260) | 0.002 | 0.102 | 1.301 (1.154-1.467) | 1.65E-05 | 0.004 |
| ODAM | 0.895 (0.832-0.963) | 0.003 | 0.041 | 0.870 (0.795-0.952) | 0.002 | 0.112 | 0.947 (0.838-1.071) | 0.386 | 0.600 |
| OSCAR | 1.148 (1.073-1.229) | 6.96E-05 | 0.003 | 1.144 (1.053-1.242) | 0.001 | 0.095 | 1.162 (1.030-1.312) | 0.015 | 0.122 |
| OXCT1 | 1.124 (1.050-1.203) | 0.001 | 0.017 | 1.096 (1.009-1.191) | 0.029 | 0.373 | 1.181 (1.046-1.333) | 0.007 | 0.084 |
| PALLD | 1.094 (1.031-1.161) | 0.003 | 0.041 | 1.102 (1.025-1.185) | 0.009 | 0.223 | 1.087 (0.981-1.204) | 0.110 | 0.310 |
| PALM2 | 1.177 (1.091-1.269) | 2.43E-05 | 0.002 | 1.139 (1.036-1.251) | 0.007 | 0.204 | 1.237 (1.094-1.399) | 0.001 | 0.023 |
| PENK | 1.136 (1.064-1.213) | 1.28E-04 | 0.005 | 1.081 (0.994-1.175) | 0.069 | 0.501 | 1.238 (1.121-1.369) | 2.76E-05 | 0.005 |
| PGA4 | 1.159 (1.076-1.248) | 9.19E-05 | 0.004 | 1.177 (1.076-1.287) | 3.56E-04 | 0.046 | 1.120 (0.980-1.279) | 0.097 | 0.293 |
| PI3 | 1.158 (1.085-1.236) | 1.07E-05 | 0.001 | 1.167 (1.077-1.264) | 1.58E-04 | 0.033 | 1.135 (1.014-1.271) | 0.028 | 0.167 |
| PIGR | 1.156 (1.081-1.237) | 2.49E-05 | 0.002 | 1.135 (1.047-1.231) | 0.002 | 0.105 | 1.208 (1.067-1.369) | 0.003 | 0.050 |
| PIK3IP1 | 1.114 (1.040-1.194) | 0.002 | 0.032 | 1.083 (0.995-1.178) | 0.064 | 0.492 | 1.179 (1.047-1.328) | 0.007 | 0.082 |
| PILRA | 1.112 (1.036-1.193) | 0.003 | 0.042 | 1.124 (1.032-1.224) | 0.007 | 0.212 | 1.091 (0.964-1.235) | 0.166 | 0.382 |
| PILRB | 1.125 (1.048-1.207) | 0.001 | 0.022 | 1.139 (1.044-1.242) | 0.003 | 0.131 | 1.103 (0.974-1.248) | 0.121 | 0.325 |
| PLA2G15 | 1.165 (1.089-1.245) | 8.46E-06 | 0.001 | 1.108 (1.021-1.202) | 0.013 | 0.271 | 1.296 (1.153-1.456) | 1.31E-05 | 0.003 |
| PLAUR | 1.204 (1.123-1.291) | 1.82E-07 | < 0.001 | 1.177 (1.080-1.282) | 2.05E-04 | 0.035 | 1.262 (1.119-1.423) | 1.42E-04 | 0.011 |
| PLIN3 | 1.135 (1.064-1.210) | 1.23E-04 | 0.005 | 1.124 (1.039-1.216) | 0.004 | 0.142 | 1.159 (1.036-1.297) | 0.010 | 0.102 |
| PLSCR3 | 1.110 (1.036-1.190) | 0.003 | 0.043 | 1.100 (1.010-1.198) | 0.029 | 0.372 | 1.131 (1.003-1.275) | 0.045 | 0.208 |
| PLXNB2 | 1.104 (1.032-1.180) | 0.004 | 0.047 | 1.059 (0.977-1.149) | 0.165 | 0.671 | 1.201 (1.068-1.350) | 0.002 | 0.043 |
| PRAP1 | 1.147 (1.057-1.244) | 0.001 | 0.021 | 1.139 (1.031-1.258) | 0.011 | 0.244 | 1.157 (1.002-1.335) | 0.047 | 0.211 |
| PRRT3 | 1.140 (1.063-1.224) | 2.69E-04 | 0.008 | 1.100 (1.008-1.200) | 0.032 | 0.388 | 1.216 (1.081-1.368) | 0.001 | 0.030 |
| PRSS8 | 1.182 (1.098-1.272) | 8.06E-06 | 0.001 | 1.119 (1.023-1.225) | 0.014 | 0.281 | 1.318 (1.162-1.496) | 1.86E-05 | 0.004 |
| PSAP | 1.134 (1.070-1.203) | 2.58E-05 | 0.002 | 1.132 (1.049-1.223) | 0.002 | 0.095 | 1.130 (1.032-1.237) | 0.008 | 0.091 |
| PTPRF | 0.892 (0.833-0.955) | 0.001 | 0.021 | 0.875 (0.805-0.951) | 0.002 | 0.095 | 0.925 (0.820-1.044) | 0.206 | 0.424 |
| PTPRS | 0.898 (0.841-0.960) | 0.001 | 0.026 | 0.900 (0.831-0.975) | 0.010 | 0.234 | 0.887 (0.789-0.996) | 0.043 | 0.205 |
| PVR | 1.153 (1.084-1.227) | 5.60E-06 | 0.001 | 1.080 (1.000-1.166) | 0.050 | 0.457 | 1.315 (1.186-1.456) | 1.72E-07 | 1.25E-04 |
| QSOX1 | 1.082 (1.027-1.141) | 0.003 | 0.043 | 1.074 (1.006-1.146) | 0.032 | 0.388 | 1.097 (1.003-1.200) | 0.043 | 0.206 |
| RAB6A | 0.906 (0.848-0.969) | 0.004 | 0.047 | 0.889 (0.819-0.965) | 0.005 | 0.166 | 0.944 (0.841-1.059) | 0.325 | 0.551 |
| RBFOX3 | 1.178 (1.101-1.259) | 1.61E-06 | < 0.001 | 1.174 (1.084-1.271) | 8.09E-05 | 0.018 | 1.192 (1.053-1.350) | 0.006 | 0.075 |
| REN | 1.146 (1.074-1.224) | 4.35E-05 | 0.002 | 1.121 (1.036-1.213) | 0.005 | 0.165 | 1.205 (1.073-1.352) | 0.002 | 0.036 |
| RNASE1 | 1.142 (1.073-1.215) | 2.62E-05 | 0.002 | 1.127 (1.031-1.233) | 0.008 | 0.220 | 1.146 (1.056-1.243) | 0.001 | 0.030 |
| RNASE4 | 1.132 (1.060-1.208) | 2.06E-04 | 0.007 | 1.108 (1.013-1.213) | 0.026 | 0.350 | 1.154 (1.053-1.265) | 0.002 | 0.043 |
| RNASE6 | 1.160 (1.096-1.227) | 2.47E-07 | < 0.001 | 1.159 (1.081-1.243) | 3.18E-05 | 0.009 | 1.158 (1.052-1.274) | 0.003 | 0.047 |
| RNASET2 | 1.144 (1.076-1.216) | 1.67E-05 | 0.001 | 1.147 (1.064-1.236) | 3.37E-04 | 0.046 | 1.145 (1.027-1.275) | 0.014 | 0.120 |
| RTBDN | 1.109 (1.038-1.184) | 0.002 | 0.032 | 1.070 (0.985-1.163) | 0.109 | 0.565 | 1.162 (1.055-1.281) | 0.002 | 0.045 |
| SCARB2 | 1.170 (1.090-1.256) | 1.48E-05 | 0.001 | 1.168 (1.071-1.273) | 4.14E-04 | 0.046 | 1.174 (1.034-1.333) | 0.013 | 0.114 |
| SCARF1 | 1.106 (1.037-1.180) | 0.002 | 0.034 | 1.074 (0.992-1.163) | 0.079 | 0.512 | 1.177 (1.052-1.316) | 0.004 | 0.065 |
| SCRG1 | 1.114 (1.035-1.199) | 0.004 | 0.050 | 1.119 (1.024-1.222) | 0.013 | 0.267 | 1.102 (0.961-1.264) | 0.165 | 0.381 |
| SEPTIN8 | 1.121 (1.048-1.199) | 0.001 | 0.018 | 1.132 (1.046-1.226) | 0.002 | 0.104 | 1.096 (0.966-1.243) | 0.155 | 0.366 |
| SHISA5 | 1.145 (1.076-1.218) | 1.94E-05 | 0.001 | 1.142 (1.054-1.238) | 0.001 | 0.087 | 1.141 (1.037-1.256) | 0.007 | 0.082 |
| SIGLEC10 | 1.108 (1.036-1.184) | 0.003 | 0.038 | 1.072 (0.988-1.164) | 0.095 | 0.537 | 1.180 (1.052-1.325) | 0.005 | 0.069 |
| SIGLEC8 | 1.139 (1.059-1.225) | 4.66E-04 | 0.011 | 1.123 (1.028-1.227) | 0.010 | 0.241 | 1.169 (1.027-1.331) | 0.018 | 0.136 |
| SLMAP | 1.119 (1.046-1.197) | 0.001 | 0.022 | 1.087 (1.001-1.181) | 0.049 | 0.453 | 1.188 (1.056-1.337) | 0.004 | 0.061 |
| SMPD1 | 1.156 (1.082-1.234) | 1.45E-05 | 0.001 | 1.155 (1.066-1.251) | 4.10E-04 | 0.046 | 1.161 (1.036-1.301) | 0.010 | 0.102 |
| SNAP25 | 1.127 (1.048-1.211) | 0.001 | 0.022 | 1.078 (0.988-1.177) | 0.092 | 0.534 | 1.236 (1.090-1.401) | 0.001 | 0.027 |
| SORCS2 | 1.146 (1.070-1.227) | 9.51E-05 | 0.004 | 1.118 (1.029-1.215) | 0.009 | 0.221 | 1.200 (1.067-1.351) | 0.002 | 0.045 |
| SPINK1 | 1.123 (1.054-1.196) | 3.17E-04 | 0.009 | 1.131 (1.047-1.222) | 0.002 | 0.100 | 1.101 (0.987-1.229) | 0.086 | 0.275 |
| SPON2 | 1.118 (1.044-1.199) | 0.002 | 0.027 | 1.083 (0.996-1.179) | 0.063 | 0.489 | 1.191 (1.056-1.343) | 0.004 | 0.066 |
| SRPX | 1.164 (1.077-1.258) | 1.37E-04 | 0.005 | 1.151 (1.046-1.266) | 0.004 | 0.143 | 1.187 (1.036-1.361) | 0.014 | 0.116 |
| STX8 | 1.101 (1.035-1.171) | 0.002 | 0.035 | 1.106 (1.025-1.193) | 0.009 | 0.224 | 1.095 (0.983-1.220) | 0.098 | 0.294 |
| SUSD4 | 1.120 (1.050-1.194) | 0.001 | 0.013 | 1.120 (1.037-1.210) | 0.004 | 0.152 | 1.116 (0.994-1.253) | 0.064 | 0.241 |
| SYT1 | 1.112 (1.050-1.178) | 3.09E-04 | 0.009 | 1.082 (1.005-1.165) | 0.035 | 0.401 | 1.164 (1.061-1.276) | 0.001 | 0.033 |
| TAFA5 | 1.120 (1.048-1.197) | 0.001 | 0.017 | 1.101 (1.015-1.193) | 0.020 | 0.327 | 1.148 (1.021-1.291) | 0.021 | 0.145 |
| TCOF1 | 1.116 (1.047-1.189) | 0.001 | 0.017 | 1.113 (1.029-1.204) | 0.007 | 0.212 | 1.118 (0.999-1.250) | 0.051 | 0.217 |
| TFF2 | 1.146 (1.072-1.224) | 5.81E-05 | 0.003 | 1.105 (1.018-1.199) | 0.017 | 0.302 | 1.231 (1.099-1.380) | 3.50E-04 | 0.017 |
| TFF3 | 1.122 (1.062-1.186) | 4.46E-05 | 0.002 | 1.122 (1.049-1.201) | 0.001 | 0.070 | 1.118 (1.016-1.230) | 0.022 | 0.146 |
| TGFB1 | 1.106 (1.036-1.180) | 0.003 | 0.037 | 1.065 (0.982-1.155) | 0.128 | 0.596 | 1.192 (1.069-1.329) | 0.002 | 0.036 |
| TGFBR2 | 1.131 (1.056-1.210) | 3.99E-04 | 0.010 | 1.089 (1.001-1.186) | 0.048 | 0.453 | 1.218 (1.091-1.360) | 4.44E-04 | 0.017 |
| THBS2 | 1.137 (1.067-1.210) | 6.35E-05 | 0.003 | 1.103 (1.021-1.192) | 0.013 | 0.271 | 1.202 (1.082-1.335) | 0.001 | 0.020 |
| THY1 | 1.120 (1.044-1.201) | 0.002 | 0.026 | 1.068 (0.980-1.164) | 0.131 | 0.602 | 1.226 (1.090-1.379) | 0.001 | 0.021 |
| TIMP1 | 1.117 (1.041-1.197) | 0.002 | 0.031 | 1.066 (0.978-1.162) | 0.144 | 0.628 | 1.226 (1.089-1.381) | 0.001 | 0.024 |
| TNFRSF10A | 1.162 (1.089-1.241) | 6.66E-06 | 0.001 | 1.128 (1.041-1.223) | 0.003 | 0.131 | 1.236 (1.102-1.385) | 2.80E-04 | 0.016 |
| TNFRSF10B | 1.153 (1.109-1.198) | 6.09E-13 | < 0.001 | 1.160 (1.106-1.216) | 7.91E-10 | 2.31E-06 | 1.145 (1.068-1.227) | 1.25E-04 | 0.011 |
| TNFRSF12A | 1.140 (1.063-1.222) | 2.44E-04 | 0.008 | 1.087 (0.998-1.185) | 0.056 | 0.473 | 1.243 (1.102-1.402) | 4.14E-04 | 0.017 |
| TNFRSF13B | 1.105 (1.038-1.176) | 0.002 | 0.027 | 1.070 (0.988-1.159) | 0.098 | 0.545 | 1.154 (1.050-1.268) | 0.003 | 0.050 |
| TNFRSF14 | 1.180 (1.105-1.259) | 6.60E-07 | < 0.001 | 1.152 (1.063-1.248) | 0.001 | 0.058 | 1.230 (1.103-1.371) | 1.85E-04 | 0.013 |
| TNFRSF1A | 1.166 (1.086-1.252) | 2.14E-05 | 0.001 | 1.109 (1.015-1.212) | 0.022 | 0.332 | 1.281 (1.138-1.441) | 3.86E-05 | 0.006 |
| TNFRSF1B | 1.115 (1.056-1.176) | 7.60E-05 | 0.003 | 1.076 (1.001-1.156) | 0.046 | 0.439 | 1.164 (1.078-1.256) | 1.03E-04 | 0.010 |
| TNFRSF6B | 1.114 (1.040-1.193) | 0.002 | 0.032 | 1.039 (0.953-1.133) | 0.384 | 0.825 | 1.267 (1.135-1.416) | 2.74E-05 | 0.005 |
| TNFSF11 | 0.899 (0.840-0.962) | 0.002 | 0.032 | 0.878 (0.808-0.954) | 0.002 | 0.104 | 0.940 (0.836-1.058) | 0.306 | 0.533 |
| TPP1 | 1.097 (1.030-1.168) | 0.004 | 0.047 | 1.067 (0.986-1.154) | 0.106 | 0.122 | 1.155 (1.041-1.282) | 0.006 | 0.082 |
| TPSG1 | 1.106 (1.040-1.175) | 0.001 | 0.022 | 1.122 (1.041-1.209) | 0.003 | 0.469 | 1.075 (0.969-1.193) | 0.172 | 0.388 |
| TREM2 | 1.123 (1.046-1.206) | 0.001 | 0.026 | 1.090 (0.999-1.190) | 0.053 | 0.519 | 1.198 (1.060-1.354) | 0.004 | 0.060 |
| UBE2L6 | 1.126 (1.048-1.209) | 0.001 | 0.022 | 1.080 (0.990-1.179) | 0.083 | 0.354 | 1.224 (1.080-1.388) | 0.002 | 0.036 |
| ULBP2 | 1.131 (1.058-1.210) | 3.09E-04 | 0.009 | 1.097 (1.011-1.191) | 0.027 | 0.001 | 1.207 (1.076-1.354) | 0.001 | 0.033 |
| VSIG2 | 1.235 (1.154-1.322) | 1.06E-09 | < 0.001 | 1.232 (1.134-1.337) | 7.12E-07 | 0.473 | 1.241 (1.100-1.399) | 4.42E-04 | 0.017 |
| VSIG4 | 1.136 (1.057-1.221) | 0.001 | 0.013 | 1.090 (0.998-1.192) | 0.056 | 0.122 | 1.237 (1.093-1.401) | 0.001 | 0.024 |
| WFDC2 | 1.190 (1.108-1.278) | 1.69E-06 | < 0.001 | 1.146 (1.048-1.254) | 0.003 | 0.009 | 1.277 (1.136-1.436) | 4.18E-05 | 0.006 |
| WFIKKN1 | 0.823 (0.766-0.884) | 9.78E-08 | < 0.001 | 0.826 (0.757-0.902) | 2.10E-05 | 0.221 | 0.814 (0.718-0.922) | 0.001 | 0.033 |
| YAP1 | 1.152 (1.067-1.245) | 2.99E-04 | 0.009 | 1.135 (1.033-1.247) | 0.009 | 0.502 | 1.184 (1.035-1.353) | 0.014 | 0.116 |
| ZNRD2 | 1.109 (1.035-1.188) | 0.003 | 0.044 | 1.081 (0.993-1.177) | 0.072 | 0.122 | 1.173 (1.042-1.321) | 0.008 | 0.091 |

| **Supplementary Table 30. Detailed protein associations with MDD in the primary, training, and testing analyses.** | | | | | | | | | |
| --- | --- | --- | --- | --- | --- | --- | --- | --- | --- |
| **Protein** | **Primary analysis** | | | **Training set** | | | **Testing set** | | |
|  | **HR (95% CI)** | **P** | **FDR** | **HR (95% CI)** | **P** | **FDR** | **HR (95% CI)** | **P** | **FDR** |
| A1BG | 1.178 (1.081-1.285) | 2.04E-04 | 0.005 | 1.178 (1.062-1.307) | 0.002 | 0.029 | 1.180 (1.009-1.380) | 0.038 | 0.644 |
| ACAA1 | 1.117 (1.036-1.204) | 0.004 | 0.036 | 1.113 (1.018-1.216) | 0.018 | 0.120 | 1.125 (0.979-1.293) | 0.098 | 0.838 |
| ACE2 | 1.156 (1.069-1.249) | 2.55E-04 | 0.006 | 1.095 (0.996-1.204) | 0.062 | 0.234 | 1.291 (1.131-1.472) | 1.49E-04 | 0.062 |
| ACVRL1 | 1.164 (1.078-1.258) | 1.11E-04 | 0.003 | 1.148 (1.047-1.260) | 0.003 | 0.041 | 1.201 (1.045-1.381) | 0.010 | 0.431 |
| ADA2 | 1.169 (1.085-1.260) | 3.78E-05 | 0.001 | 1.167 (1.069-1.275) | 0.001 | 0.012 | 1.176 (1.023-1.351) | 0.023 | 0.567 |
| ADAM12 | 1.105 (1.041-1.172) | 0.001 | 0.014 | 1.108 (1.033-1.188) | 0.004 | 0.048 | 1.099 (0.980-1.232) | 0.105 | 0.838 |
| ADAMTS13 | 0.890 (0.827-0.958) | 0.002 | 0.023 | 0.917 (0.840-1.001) | 0.052 | 0.213 | 0.827 (0.723-0.948) | 0.006 | 0.394 |
| ADAMTS8 | 0.841 (0.781-0.906) | 4.82E-06 | 3.60E-04 | 0.881 (0.806-0.962) | 4.88E-03 | 0.052 | 0.756 (0.661-0.864) | 3.96E-05 | 0.029 |
| ADAMTSL2 | 1.157 (1.068-1.254) | 3.51E-04 | 0.007 | 1.195 (1.087-1.314) | 0.000 | 0.006 | 1.068 (0.918-1.243) | 0.393 | 0.992 |
| ADGRF5 | 1.144 (1.053-1.243) | 0.001 | 0.019 | 1.132 (1.025-1.251) | 0.015 | 0.108 | 1.167 (1.006-1.355) | 0.042 | 0.671 |
| ADGRG1 | 1.114 (1.040-1.194) | 0.002 | 0.025 | 1.102 (1.014-1.196) | 0.021 | 0.134 | 1.142 (1.005-1.298) | 0.042 | 0.671 |
| ADM | 1.158 (1.057-1.269) | 0.002 | 0.02 | 1.165 (1.045-1.299) | 0.006 | 0.060 | 1.137 (0.962-1.344) | 0.133 | 0.879 |
| AGR2 | 1.139 (1.059-1.226) | 4.69E-04 | 0.008 | 1.152 (1.057-1.256) | 0.001 | 0.023 | 1.108 (0.967-1.271) | 0.141 | 0.883 |
| AIFM1 | 1.149 (1.065-1.239) | 3.11E-04 | 0.006 | 1.147 (1.048-1.255) | 0.003 | 0.036 | 1.152 (1.003-1.323) | 0.045 | 0.690 |
| ALDH3A1 | 1.119 (1.047-1.196) | 0.001 | 0.013 | 1.104 (1.017-1.199) | 0.019 | 0.123 | 1.147 (1.026-1.282) | 0.016 | 0.508 |
| AMBP | 1.141 (1.054-1.236) | 0.001 | 0.015 | 1.153 (1.049-1.268) | 0.003 | 0.040 | 1.112 (0.960-1.287) | 0.156 | 0.897 |
| AMIGO2 | 1.122 (1.042-1.208) | 0.002 | 0.025 | 1.164 (1.066-1.270) | 0.001 | 0.014 | 1.026 (0.896-1.175) | 0.713 | 0.992 |
| ANGPT2 | 1.119 (1.038-1.206) | 0.004 | 0.035 | 1.118 (1.022-1.222) | 0.014 | 0.108 | 1.120 (0.972-1.290) | 0.118 | 0.859 |
| ANXA10 | 1.129 (1.051-1.213) | 0.001 | 0.013 | 1.112 (1.020-1.212) | 0.016 | 0.113 | 1.169 (1.028-1.331) | 0.018 | 0.542 |
| APBB1IP | 1.106 (1.031-1.187) | 0.005 | 0.043 | 1.141 (1.051-1.237) | 0.002 | 0.025 | 1.023 (0.891-1.174) | 0.749 | 0.992 |
| APLP1 | 0.875 (0.807-0.949) | 0.001 | 0.017 | 0.882 (0.800-0.972) | 0.012 | 0.096 | 0.857 (0.741-0.992) | 0.039 | 0.644 |
| APOM | 0.884 (0.820-0.953) | 0.001 | 0.017 | 0.865 (0.791-0.945) | 0.001 | 0.023 | 0.934 (0.811-1.075) | 0.340 | 0.992 |
| AREG | 1.179 (1.103-1.262) | 1.54E-06 | 1.54E-04 | 1.180 (1.089-1.279) | 5.63E-05 | 0.003 | 1.175 (1.038-1.329) | 0.010 | 0.433 |
| ARSB | 1.123 (1.043-1.208) | 0.002 | 0.023 | 1.151 (1.055-1.255) | 0.002 | 0.025 | 1.054 (0.918-1.211) | 0.454 | 0.992 |
| ASAH1 | 1.112 (1.033-1.198) | 0.005 | 0.043 | 1.148 (1.050-1.255) | 0.002 | 0.033 | 1.043 (0.909-1.198) | 0.549 | 0.992 |
| ASGR1 | 1.193 (1.103-1.291) | 9.63E-06 | 0.001 | 1.188 (1.083-1.303) | 0.000 | 0.007 | 1.206 (1.040-1.398) | 0.013 | 0.468 |
| ASGR2 | 1.130 (1.045-1.222) | 0.002 | 0.025 | 1.156 (1.051-1.271) | 0.003 | 0.036 | 1.074 (0.932-1.238) | 0.325 | 0.992 |
| B2M | 1.111 (1.046-1.180) | 0.001 | 0.011 | 1.103 (1.029-1.182) | 0.006 | 0.059 | 1.144 (0.998-1.311) | 0.054 | 0.693 |
| BAIAP2 | 1.169 (1.086-1.259) | 3.20E-05 | 0.001 | 1.165 (1.068-1.271) | 0.001 | 0.012 | 1.179 (1.026-1.354) | 0.020 | 0.566 |
| BAX | 1.121 (1.039-1.208) | 0.003 | 0.032 | 1.144 (1.046-1.251) | 0.003 | 0.040 | 1.068 (0.929-1.229) | 0.354 | 0.992 |
| BCAN | 0.835 (0.770-0.904) | 9.33E-06 | 0.001 | 0.820 (0.746-0.902) | 0.000 | 0.002 | 0.871 (0.749-1.012) | 0.071 | 0.800 |
| BCAT1 | 1.117 (1.035-1.205) | 0.005 | 0.042 | 1.117 (1.019-1.224) | 0.018 | 0.120 | 1.112 (0.969-1.276) | 0.130 | 0.873 |
| BST2 | 1.189 (1.115-1.267) | 1.26E-07 | 3.06E-05 | 1.186 (1.098-1.281) | 1.42E-05 | 0.001 | 1.193 (1.063-1.340) | 0.003 | 0.272 |
| BTN3A2 | 1.187 (1.102-1.279) | 6.46E-06 | 4.09E-04 | 1.193 (1.093-1.303) | 8.31E-05 | 0.003 | 1.167 (1.014-1.343) | 0.031 | 0.620 |
| C1RL | 1.172 (1.076-1.276) | 2.54E-04 | 0.006 | 1.197 (1.081-1.324) | 0.001 | 0.011 | 1.117 (0.956-1.307) | 0.165 | 0.899 |
| C7 | 1.140 (1.048-1.239) | 0.002 | 0.025 | 1.185 (1.072-1.310) | 0.001 | 0.018 | 1.043 (0.897-1.212) | 0.583 | 0.992 |
| C9 | 1.181 (1.083-1.288) | 1.69E-04 | 0.004 | 1.181 (1.065-1.311) | 0.002 | 0.027 | 1.182 (1.011-1.383) | 0.037 | 0.639 |
| CA12 | 1.140 (1.061-1.226) | 3.76E-04 | 0.007 | 1.152 (1.055-1.257) | 0.002 | 0.026 | 1.112 (0.977-1.266) | 0.107 | 0.838 |
| CA14 | 0.871 (0.802-0.945) | 0.001 | 0.013 | 0.882 (0.800-0.972) | 0.011 | 0.091 | 0.842 (0.724-0.979) | 0.025 | 0.587 |
| CA6 | 0.812 (0.750-0.878) | 2.13E-07 | 4.15E-05 | 0.812 (0.738-0.892) | 1.64E-05 | 0.001 | 0.812 (0.705-0.936) | 0.004 | 0.327 |
| CALCA | 1.136 (1.039-1.242) | 0.005 | 0.044 | 1.173 (1.058-1.301) | 0.002 | 0.033 | 1.039 (0.873-1.237) | 0.665 | 0.992 |
| CANT1 | 1.118 (1.036-1.208) | 0.004 | 0.04 | 1.136 (1.037-1.245) | 0.006 | 0.062 | 1.075 (0.933-1.240) | 0.316 | 0.992 |
| CBLN4 | 0.891 (0.827-0.960) | 0.002 | 0.025 | 0.959 (0.878-1.047) | 0.351 | 0.592 | 0.746 (0.652-0.854) | 2.15E-05 | 0.025 |
| CCL15 | 1.120 (1.043-1.203) | 0.002 | 0.021 | 1.114 (1.023-1.213) | 0.013 | 0.101 | 1.132 (0.994-1.289) | 0.062 | 0.736 |
| CCL3 | 1.084 (1.026-1.145) | 0.004 | 0.038 | 1.088 (1.019-1.162) | 0.012 | 0.098 | 1.070 (0.969-1.182) | 0.181 | 0.916 |
| CCL5 | 1.120 (1.040-1.205) | 0.003 | 0.028 | 1.118 (1.024-1.220) | 0.013 | 0.102 | 1.120 (0.978-1.284) | 0.102 | 0.838 |
| CCL7 | 1.162 (1.087-1.242) | 9.82E-06 | 0.001 | 1.178 (1.089-1.273) | 0.000 | 0.002 | 1.115 (0.983-1.264) | 0.090 | 0.820 |
| CD163 | 1.119 (1.038-1.207) | 0.003 | 0.035 | 1.102 (1.007-1.206) | 0.034 | 0.167 | 1.159 (1.009-1.332) | 0.037 | 0.639 |
| CD27 | 1.125 (1.044-1.212) | 0.002 | 0.023 | 1.178 (1.078-1.286) | 0.000 | 0.007 | 1.007 (0.874-1.159) | 0.926 | 0.993 |
| CD274 | 1.150 (1.073-1.233) | 8.11E-05 | 0.003 | 1.166 (1.075-1.264) | 0.000 | 0.006 | 1.107 (0.965-1.271) | 0.146 | 0.887 |
| CD276 | 1.176 (1.089-1.269) | 3.26E-05 | 0.001 | 1.207 (1.103-1.322) | 0.000 | 0.002 | 1.101 (0.956-1.269) | 0.182 | 0.916 |
| CD300E | 1.186 (1.097-1.283) | 1.81E-05 | 0.001 | 1.233 (1.124-1.353) | 0.000 | 0.001 | 1.082 (0.934-1.254) | 0.294 | 0.992 |
| CD302 | 1.193 (1.108-1.283) | 2.47E-06 | 2.06E-04 | 1.237 (1.135-1.347) | 1.17E-06 | 2.31E-04 | 1.090 (0.948-1.253) | 0.227 | 0.959 |
| CD34 | 0.897 (0.832-0.967) | 0.005 | 0.042 | 0.897 (0.820-0.981) | 0.017 | 0.117 | 0.898 (0.782-1.030) | 0.124 | 0.866 |
| CD36 | 1.123 (1.037-1.217) | 0.005 | 0.042 | 1.097 (0.995-1.210) | 0.062 | 0.236 | 1.180 (1.024-1.359) | 0.022 | 0.567 |
| CD4 | 1.111 (1.048-1.178) | 4.39E-04 | 0.008 | 1.123 (1.054-1.197) | 0.000 | 0.008 | 1.058 (0.928-1.207) | 0.399 | 0.992 |
| CD5 | 1.111 (1.036-1.192) | 0.003 | 0.033 | 1.196 (1.094-1.306) | 0.000 | 0.003 | 0.960 (0.836-1.103) | 0.567 | 0.992 |
| CD72 | 1.154 (1.061-1.256) | 0.001 | 0.013 | 1.248 (1.132-1.376) | 0.000 | 0.001 | 0.940 (0.799-1.105) | 0.453 | 0.992 |
| CD74 | 1.155 (1.069-1.248) | 2.59E-04 | 0.006 | 1.178 (1.074-1.291) | 0.000 | 0.011 | 1.100 (0.953-1.270) | 0.191 | 0.926 |
| CD79B | 1.141 (1.061-1.228) | 4.06E-04 | 0.008 | 1.183 (1.084-1.290) | 0.000 | 0.005 | 1.051 (0.916-1.205) | 0.481 | 0.992 |
| CD80 | 1.187 (1.097-1.284) | 2.04E-05 | 0.001 | 1.205 (1.098-1.323) | 0.000 | 0.003 | 1.139 (0.982-1.322) | 0.086 | 0.820 |
| CD83 | 1.173 (1.088-1.264) | 2.93E-05 | 0.001 | 1.180 (1.080-1.291) | 0.000 | 0.007 | 1.154 (1.006-1.323) | 0.041 | 0.664 |
| CDCP1 | 1.226 (1.134-1.326) | 3.01E-07 | 5.48E-05 | 1.195 (1.088-1.311) | 1.79E-04 | 0.005 | 1.310 (1.135-1.511) | 2.19E-04 | 0.066 |
| CDH2 | 1.121 (1.034-1.215) | 0.006 | 0.049 | 1.128 (1.025-1.242) | 0.014 | 0.106 | 1.102 (0.949-1.279) | 0.204 | 0.936 |
| CEACAM1 | 1.133 (1.049-1.224) | 0.002 | 0.019 | 1.125 (1.026-1.233) | 0.012 | 0.096 | 1.152 (0.998-1.329) | 0.054 | 0.693 |
| CEACAM5 | 1.158 (1.074-1.249) | 1.40E-04 | 0.004 | 1.115 (1.019-1.221) | 0.018 | 0.120 | 1.272 (1.107-1.461) | 0.001 | 0.121 |
| CEACAM6 | 1.170 (1.080-1.268) | 1.24E-04 | 0.003 | 1.114 (1.009-1.231) | 0.033 | 0.165 | 1.286 (1.127-1.467) | 1.90E-04 | 0.066 |
| CEACAM8 | 1.131 (1.053-1.215) | 0.001 | 0.012 | 1.121 (1.026-1.224) | 0.011 | 0.093 | 1.158 (1.024-1.309) | 0.019 | 0.559 |
| CFB | 1.137 (1.040-1.244) | 0.005 | 0.043 | 1.150 (1.033-1.281) | 0.011 | 0.092 | 1.108 (0.943-1.301) | 0.213 | 0.938 |
| CFH | 1.198 (1.088-1.318) | 2.22E-04 | 0.005 | 1.187 (1.059-1.331) | 0.003 | 0.040 | 1.226 (1.028-1.462) | 0.023 | 0.567 |
| CFHR5 | 1.163 (1.068-1.267) | 0.001 | 0.009 | 1.164 (1.050-1.291) | 0.004 | 0.046 | 1.164 (0.999-1.357) | 0.051 | 0.693 |
| CHCHD10 | 1.156 (1.064-1.256) | 0.001 | 0.01 | 1.156 (1.046-1.279) | 0.005 | 0.050 | 1.155 (0.997-1.338) | 0.056 | 0.693 |
| CHGA | 1.193 (1.113-1.279) | 6.24E-07 | 7.91E-05 | 1.187 (1.092-1.291) | 5.56E-05 | 0.003 | 1.203 (1.061-1.364) | 0.004 | 0.317 |
| CHI3L1 | 1.184 (1.099-1.275) | 7.71E-06 | 4.78E-04 | 1.178 (1.077-1.288) | 3.32E-04 | 0.008 | 1.202 (1.053-1.372) | 0.007 | 0.396 |
| CKAP4 | 1.138 (1.059-1.222) | 4.20E-04 | 0.008 | 1.170 (1.072-1.277) | 0.000 | 0.010 | 1.075 (0.944-1.225) | 0.274 | 0.992 |
| CLEC4D | 1.240 (1.152-1.335) | 1.13E-08 | 5.51E-06 | 1.274 (1.167-1.392) | 6.27E-08 | 3.66E-05 | 1.166 (1.018-1.336) | 0.027 | 0.597 |
| CLEC5A | 1.113 (1.033-1.199) | 0.005 | 0.043 | 1.097 (1.003-1.200) | 0.043 | 0.192 | 1.154 (1.009-1.319) | 0.036 | 0.639 |
| CLEC6A | 1.126 (1.045-1.213) | 0.002 | 0.021 | 1.136 (1.041-1.241) | 0.004 | 0.049 | 1.101 (0.956-1.267) | 0.181 | 0.916 |
| CLEC7A | 1.135 (1.042-1.236) | 0.004 | 0.035 | 1.191 (1.071-1.325) | 0.001 | 0.022 | 1.030 (0.898-1.182) | 0.672 | 0.992 |
| CNTN1 | 0.872 (0.805-0.945) | 0.001 | 0.012 | 0.886 (0.805-0.975) | 0.013 | 0.103 | 0.841 (0.728-0.972) | 0.019 | 0.559 |
| CNTN5 | 0.851 (0.784-0.925) | 1.35E-04 | 0.004 | 0.870 (0.788-0.960) | 0.006 | 0.059 | 0.812 (0.699-0.943) | 0.006 | 0.394 |
| COL18A1 | 1.132 (1.044-1.226) | 0.003 | 0.027 | 1.152 (1.047-1.268) | 0.004 | 0.043 | 1.083 (0.935-1.254) | 0.289 | 0.992 |
| COL4A1 | 1.145 (1.064-1.231) | 2.72E-04 | 0.006 | 1.141 (1.047-1.243) | 0.003 | 0.034 | 1.149 (1.001-1.319) | 0.048 | 0.693 |
| COL5A1 | 1.129 (1.045-1.221) | 0.002 | 0.025 | 1.192 (1.087-1.307) | 0.000 | 0.005 | 0.994 (0.858-1.153) | 0.940 | 0.993 |
| COL6A3 | 1.151 (1.064-1.244) | 4.09E-04 | 0.008 | 1.179 (1.076-1.292) | 0.000 | 0.010 | 1.085 (0.936-1.257) | 0.281 | 0.992 |
| CPM | 1.162 (1.072-1.260) | 2.58E-04 | 0.006 | 1.127 (1.024-1.241) | 0.015 | 0.109 | 1.252 (1.079-1.452) | 0.003 | 0.279 |
| CRYBB2 | 1.092 (1.028-1.160) | 0.004 | 0.04 | 1.108 (1.034-1.188) | 0.004 | 0.043 | 1.045 (0.924-1.182) | 0.486 | 0.992 |
| CSF1 | 1.171 (1.086-1.261) | 3.63E-05 | 0.001 | 1.194 (1.092-1.305) | 0.000 | 0.003 | 1.118 (0.974-1.284) | 0.113 | 0.854 |
| CST3 | 1.126 (1.039-1.221) | 0.004 | 0.038 | 1.118 (1.015-1.232) | 0.024 | 0.139 | 1.145 (0.988-1.325) | 0.071 | 0.800 |
| CTSL | 1.172 (1.086-1.264) | 4.02E-05 | 0.001 | 1.215 (1.110-1.329) | 0.000 | 0.002 | 1.071 (0.932-1.231) | 0.333 | 0.992 |
| CTSO | 1.134 (1.054-1.220) | 0.001 | 0.012 | 1.145 (1.049-1.249) | 0.002 | 0.032 | 1.109 (0.967-1.272) | 0.140 | 0.882 |
| CTSZ | 1.147 (1.063-1.238) | 4.28E-04 | 0.008 | 1.189 (1.086-1.302) | 0.000 | 0.005 | 1.052 (0.912-1.213) | 0.489 | 0.992 |
| CXADR | 1.161 (1.082-1.245) | 3.12E-05 | 0.001 | 1.196 (1.101-1.300) | 0.000 | 0.002 | 1.079 (0.946-1.231) | 0.258 | 0.992 |
| CXCL11 | 1.116 (1.039-1.200) | 0.003 | 0.029 | 1.138 (1.046-1.238) | 0.003 | 0.035 | 1.058 (0.921-1.215) | 0.427 | 0.992 |
| CXCL8 | 1.112 (1.037-1.191) | 0.003 | 0.03 | 1.143 (1.054-1.239) | 0.001 | 0.022 | 1.036 (0.905-1.187) | 0.605 | 0.992 |
| CXCL9 | 1.187 (1.103-1.278) | 4.66E-06 | 3.58E-04 | 1.205 (1.107-1.311) | 1.56E-05 | 0.001 | 1.134 (0.978-1.314) | 0.096 | 0.838 |
| DBI | 1.118 (1.035-1.207) | 0.005 | 0.042 | 1.138 (1.037-1.248) | 0.006 | 0.062 | 1.074 (0.935-1.233) | 0.315 | 0.992 |
| DCBLD2 | 1.116 (1.034-1.204) | 0.005 | 0.042 | 1.176 (1.074-1.288) | 0.000 | 0.010 | 0.986 (0.858-1.134) | 0.847 | 0.993 |
| DDAH1 | 1.161 (1.079-1.249) | 6.11E-05 | 0.002 | 1.167 (1.069-1.273) | 0.001 | 0.011 | 1.145 (1.002-1.309) | 0.047 | 0.693 |
| DEFB4A_DEFB4B | 1.166 (1.078-1.261) | 1.24E-04 | 0.003 | 1.205 (1.097-1.323) | 0.000 | 0.003 | 1.075 (0.929-1.243) | 0.333 | 0.992 |
| DLK1 | 0.890 (0.823-0.961) | 0.003 | 0.032 | 0.965 (0.879-1.059) | 0.449 | 0.670 | 0.742 (0.646-0.853) | 2.62E-05 | 0.025 |
| DLL1 | 1.136 (1.053-1.225) | 0.001 | 0.014 | 1.190 (1.088-1.301) | 0.000 | 0.005 | 1.018 (0.884-1.172) | 0.806 | 0.992 |
| DPP7 | 1.178 (1.093-1.269) | 1.68E-05 | 0.001 | 1.217 (1.114-1.330) | 0.000 | 0.001 | 1.084 (0.946-1.243) | 0.244 | 0.979 |
| DPY30 | 1.134 (1.054-1.221) | 0.001 | 0.012 | 1.172 (1.076-1.278) | 0.000 | 0.007 | 1.045 (0.906-1.205) | 0.546 | 0.992 |
| DSC2 | 1.119 (1.037-1.207) | 0.004 | 0.036 | 1.143 (1.045-1.250) | 0.003 | 0.040 | 1.058 (0.916-1.221) | 0.445 | 0.992 |
| ECHDC3 | 1.168 (1.083-1.260) | 6.12E-05 | 0.002 | 1.161 (1.061-1.270) | 0.001 | 0.022 | 1.180 (1.024-1.360) | 0.022 | 0.567 |
| ECHS1 | 1.131 (1.040-1.230) | 0.004 | 0.039 | 1.149 (1.040-1.270) | 0.006 | 0.062 | 1.083 (0.927-1.265) | 0.314 | 0.992 |
| EDN1 | 1.140 (1.050-1.238) | 0.002 | 0.022 | 1.127 (1.020-1.245) | 0.019 | 0.124 | 1.164 (1.007-1.346) | 0.040 | 0.660 |
| EFNA1 | 1.114 (1.033-1.202) | 0.005 | 0.046 | 1.119 (1.023-1.224) | 0.014 | 0.106 | 1.098 (0.953-1.266) | 0.196 | 0.936 |
| EFNA4 | 1.206 (1.121-1.297) | 4.95E-07 | 7.18E-05 | 1.209 (1.111-1.316) | 1.15E-05 | 0.001 | 1.198 (1.039-1.381) | 0.013 | 0.468 |
| EGFL7 | 1.120 (1.038-1.208) | 0.003 | 0.034 | 1.109 (1.012-1.216) | 0.027 | 0.149 | 1.143 (1.001-1.305) | 0.048 | 0.693 |
| EGFR | 0.834 (0.776-0.896) | 6.93E-07 | 8.42E-05 | 0.833 (0.765-0.906) | 2.13E-05 | 0.002 | 0.836 (0.730-0.958) | 0.010 | 0.431 |
| ELN | 1.190 (1.073-1.321) | 0.001 | 0.014 | 1.287 (1.140-1.454) | 0.000 | 0.002 | 0.964 (0.789-1.177) | 0.719 | 0.992 |
| EPHA2 | 1.164 (1.081-1.252) | 5.33E-05 | 0.002 | 1.211 (1.108-1.322) | 0.000 | 0.002 | 1.064 (0.928-1.221) | 0.374 | 0.992 |
| EPPK1 | 1.131 (1.049-1.220) | 0.001 | 0.017 | 1.193 (1.092-1.304) | 0.000 | 0.003 | 0.991 (0.859-1.145) | 0.907 | 0.993 |
| ESAM | 1.108 (1.030-1.191) | 0.006 | 0.049 | 1.143 (1.048-1.247) | 0.003 | 0.034 | 1.032 (0.904-1.179) | 0.641 | 0.992 |
| FABP1 | 1.163 (1.077-1.257) | 1.29E-04 | 0.004 | 1.159 (1.057-1.271) | 0.002 | 0.027 | 1.174 (1.017-1.355) | 0.028 | 0.599 |
| FAM3C | 1.124 (1.041-1.214) | 0.003 | 0.03 | 1.141 (1.041-1.250) | 0.005 | 0.050 | 1.087 (0.941-1.255) | 0.257 | 0.992 |
| FAP | 0.879 (0.815-0.948) | 0.001 | 0.012 | 0.905 (0.826-0.991) | 0.030 | 0.159 | 0.821 (0.716-0.942) | 0.005 | 0.358 |
| FAS | 1.101 (1.031-1.175) | 0.004 | 0.037 | 1.143 (1.058-1.234) | 0.001 | 0.013 | 1.018 (0.894-1.158) | 0.792 | 0.992 |
| FCN1 | 1.138 (1.040-1.245) | 0.005 | 0.043 | 1.177 (1.056-1.312) | 0.003 | 0.039 | 1.055 (0.902-1.234) | 0.506 | 0.992 |
| FCRLB | 1.123 (1.049-1.202) | 0.001 | 0.013 | 1.130 (1.043-1.224) | 0.003 | 0.035 | 1.103 (0.968-1.256) | 0.143 | 0.887 |
| FETUB | 1.138 (1.049-1.234) | 0.002 | 0.022 | 1.135 (1.031-1.250) | 0.010 | 0.083 | 1.145 (0.982-1.333) | 0.083 | 0.820 |
| FGF12 | 1.156 (1.061-1.261) | 0.001 | 0.014 | 1.161 (1.047-1.288) | 0.005 | 0.050 | 1.146 (0.979-1.340) | 0.089 | 0.820 |
| FGFBP3 | 0.870 (0.802-0.943) | 0.001 | 0.012 | 0.856 (0.777-0.943) | 0.002 | 0.027 | 0.903 (0.778-1.047) | 0.176 | 0.916 |
| FGFR4 | 1.148 (1.064-1.239) | 3.63E-04 | 0.007 | 1.180 (1.078-1.292) | 0.000 | 0.008 | 1.078 (0.937-1.241) | 0.294 | 0.992 |
| FGR | 1.106 (1.033-1.183) | 0.004 | 0.037 | 1.108 (1.020-1.204) | 0.015 | 0.108 | 1.108 (0.981-1.252) | 0.100 | 0.838 |
| FN1 | 1.248 (1.144-1.361) | 6.13E-07 | 7.91E-05 | 1.251 (1.128-1.387) | 2.23E-05 | 0.002 | 1.237 (1.053-1.453) | 0.010 | 0.423 |
| FOXO3 | 1.142 (1.062-1.229) | 3.51E-04 | 0.007 | 1.172 (1.076-1.276) | 0.000 | 0.007 | 1.067 (0.928-1.228) | 0.360 | 0.992 |
| FSTL1 | 1.149 (1.065-1.238) | 3.03E-04 | 0.006 | 1.180 (1.085-1.284) | 0.000 | 0.004 | 1.062 (0.917-1.230) | 0.424 | 0.992 |
| FSTL3 | 1.188 (1.096-1.287) | 2.51E-05 | 0.001 | 1.201 (1.092-1.322) | 0.000 | 0.005 | 1.155 (0.997-1.338) | 0.055 | 0.693 |
| GAST | 1.216 (1.131-1.306) | 9.97E-08 | 2.64E-05 | 1.205 (1.106-1.313) | 2.11E-05 | 0.002 | 1.243 (1.090-1.417) | 0.001 | 0.163 |
| GCNT1 | 1.132 (1.050-1.220) | 0.001 | 0.016 | 1.192 (1.091-1.301) | 0.000 | 0.003 | 0.987 (0.855-1.140) | 0.862 | 0.993 |
| GDF15 | 1.261 (1.187-1.340) | 4.69E-14 | 1.37E-10 | 1.283 (1.192-1.381) | 3.50E-11 | 1.02E-07 | 1.228 (1.101-1.370) | 2.33E-04 | 0.066 |
| GFER | 1.129 (1.046-1.218) | 0.002 | 0.021 | 1.138 (1.039-1.246) | 0.005 | 0.054 | 1.109 (0.965-1.275) | 0.145 | 0.887 |
| GFRA1 | 1.198 (1.108-1.295) | 5.27E-06 | 3.85E-04 | 1.283 (1.171-1.405) | 8.66E-08 | 4.21E-05 | 1.013 (0.873-1.174) | 0.869 | 0.993 |
| GGH | 1.147 (1.061-1.240) | 0.001 | 0.01 | 1.192 (1.089-1.305) | 0.000 | 0.005 | 1.039 (0.896-1.205) | 0.611 | 0.992 |
| GGT1 | 1.132 (1.046-1.225) | 0.002 | 0.023 | 1.115 (1.015-1.226) | 0.024 | 0.139 | 1.171 (1.015-1.352) | 0.031 | 0.620 |
| GIPC2 | 1.150 (1.068-1.239) | 2.33E-04 | 0.005 | 1.106 (1.008-1.213) | 0.033 | 0.166 | 1.236 (1.096-1.394) | 0.001 | 0.110 |
| GKN1 | 1.146 (1.070-1.228) | 1.06E-04 | 0.003 | 1.180 (1.092-1.277) | 0.000 | 0.002 | 1.055 (0.918-1.212) | 0.450 | 0.992 |
| GOLM2 | 1.107 (1.032-1.188) | 0.005 | 0.042 | 1.119 (1.032-1.214) | 0.007 | 0.064 | 1.077 (0.939-1.236) | 0.291 | 0.992 |
| GPKOW | 1.111 (1.049-1.176) | 2.91E-04 | 0.006 | 1.132 (1.058-1.210) | 0.000 | 0.007 | 1.075 (0.961-1.202) | 0.204 | 0.936 |
| GPNMB | 1.128 (1.045-1.219) | 0.002 | 0.024 | 1.151 (1.051-1.261) | 0.003 | 0.034 | 1.071 (0.928-1.236) | 0.350 | 0.992 |
| GRN | 1.118 (1.037-1.205) | 0.003 | 0.035 | 1.123 (1.028-1.226) | 0.010 | 0.086 | 1.104 (0.959-1.271) | 0.169 | 0.912 |
| GRPEL1 | 1.205 (1.123-1.293) | 2.01E-07 | 4.15E-05 | 1.207 (1.111-1.312) | 8.70E-06 | 0.001 | 1.199 (1.049-1.371) | 0.008 | 0.410 |
| GSN | 0.915 (0.869-0.963) | 0.001 | 0.012 | 0.862 (0.786-0.947) | 0.002 | 0.028 | 0.949 (0.867-1.039) | 0.260 | 0.992 |
| GUSB | 1.121 (1.034-1.215) | 0.006 | 0.049 | 1.135 (1.030-1.250) | 0.011 | 0.090 | 1.093 (0.943-1.268) | 0.238 | 0.975 |
| HAVCR1 | 1.207 (1.118-1.304) | 1.59E-06 | 1.54E-04 | 1.249 (1.139-1.370) | 2.46E-06 | 3.98E-04 | 1.119 (0.972-1.289) | 0.117 | 0.858 |
| HAVCR2 | 1.165 (1.080-1.257) | 8.41E-05 | 0.003 | 1.154 (1.054-1.263) | 0.002 | 0.029 | 1.194 (1.036-1.376) | 0.014 | 0.470 |
| HDGF | 1.138 (1.055-1.228) | 0.001 | 0.013 | 1.159 (1.059-1.269) | 0.001 | 0.023 | 1.089 (0.947-1.251) | 0.232 | 0.970 |
| HEG1 | 1.130 (1.037-1.232) | 0.006 | 0.048 | 1.094 (0.986-1.213) | 0.090 | 0.289 | 1.219 (1.042-1.425) | 0.013 | 0.468 |
| HEPACAM2 | 0.891 (0.821-0.967) | 0.006 | 0.049 | 0.898 (0.815-0.990) | 0.030 | 0.157 | 0.877 (0.755-1.019) | 0.087 | 0.820 |
| HGF | 1.251 (1.156-1.354) | 2.48E-08 | 9.04E-06 | 1.262 (1.149-1.386) | 1.10E-06 | 2.31E-04 | 1.230 (1.062-1.423) | 0.006 | 0.382 |
| HHEX | 1.128 (1.038-1.226) | 0.005 | 0.043 | 1.136 (1.027-1.256) | 0.013 | 0.102 | 1.109 (0.954-1.289) | 0.179 | 0.916 |
| HMOX2 | 1.104 (1.031-1.182) | 0.005 | 0.042 | 1.120 (1.035-1.212) | 0.005 | 0.054 | 1.062 (0.928-1.216) | 0.382 | 0.992 |
| HPGDS | 0.878 (0.813-0.948) | 0.001 | 0.013 | 0.818 (0.746-0.896) | 0.000 | 0.001 | 1.029 (0.902-1.175) | 0.667 | 0.992 |
| HPSE | 1.141 (1.049-1.241) | 0.002 | 0.025 | 1.133 (1.025-1.254) | 0.015 | 0.109 | 1.153 (0.990-1.343) | 0.067 | 0.769 |
| HYAL1 | 1.111 (1.034-1.195) | 0.004 | 0.039 | 1.093 (1.003-1.191) | 0.043 | 0.192 | 1.155 (1.010-1.321) | 0.035 | 0.628 |
| ICAM1 | 1.183 (1.100-1.272) | 6.43E-06 | 4.09E-04 | 1.208 (1.108-1.316) | 1.71E-05 | 0.001 | 1.127 (0.983-1.293) | 0.087 | 0.820 |
| IDUA | 1.147 (1.064-1.238) | 3.83E-04 | 0.007 | 1.204 (1.099-1.320) | 0.000 | 0.003 | 1.029 (0.899-1.177) | 0.681 | 0.992 |
| IFI30 | 1.209 (1.108-1.318) | 1.96E-05 | 0.001 | 1.228 (1.108-1.361) | 0.000 | 0.003 | 1.158 (0.983-1.365) | 0.079 | 0.820 |
| IGF2R | 1.131 (1.047-1.221) | 0.002 | 0.021 | 1.155 (1.055-1.265) | 0.002 | 0.028 | 1.068 (0.926-1.232) | 0.368 | 0.992 |
| IGFBP4 | 1.204 (1.116-1.299) | 1.77E-06 | 1.60E-04 | 1.210 (1.108-1.323) | 2.48E-05 | 0.002 | 1.185 (1.023-1.373) | 0.024 | 0.573 |
| IGSF8 | 1.179 (1.098-1.265) | 5.79E-06 | 4.02E-04 | 1.221 (1.128-1.322) | 7.73E-07 | 1.94E-04 | 1.055 (0.916-1.216) | 0.454 | 0.992 |
| IL10 | 1.151 (1.082-1.224) | 9.02E-06 | 0.001 | 1.149 (1.067-1.238) | 0.000 | 0.006 | 1.155 (1.030-1.294) | 0.014 | 0.470 |
| IL10RB | 1.126 (1.049-1.208) | 0.001 | 0.015 | 1.140 (1.045-1.242) | 0.003 | 0.037 | 1.098 (0.967-1.248) | 0.149 | 0.890 |
| IL15 | 1.152 (1.073-1.237) | 8.91E-05 | 0.003 | 1.126 (1.034-1.227) | 0.006 | 0.062 | 1.211 (1.067-1.376) | 0.003 | 0.281 |
| IL17F | 1.145 (1.075-1.219) | 2.48E-05 | 0.001 | 1.124 (1.040-1.214) | 0.003 | 0.039 | 1.191 (1.069-1.327) | 0.002 | 0.179 |
| IL18 | 1.153 (1.073-1.238) | 1.03E-04 | 0.003 | 1.175 (1.079-1.280) | 0.000 | 0.006 | 1.102 (0.966-1.257) | 0.149 | 0.889 |
| IL18BP | 1.192 (1.109-1.282) | 2.14E-06 | 1.84E-04 | 1.194 (1.095-1.301) | 5.89E-05 | 0.003 | 1.192 (1.040-1.366) | 0.012 | 0.467 |
| IL18R1 | 1.206 (1.119-1.300) | 9.53E-07 | 1.11E-04 | 1.223 (1.119-1.336) | 9.45E-06 | 0.001 | 1.168 (1.016-1.342) | 0.029 | 0.599 |
| IL19 | 1.146 (1.066-1.232) | 2.30E-04 | 0.005 | 1.171 (1.075-1.276) | 0.000 | 0.008 | 1.082 (0.944-1.240) | 0.256 | 0.992 |
| IL1RL1 | 1.161 (1.071-1.260) | 3.13E-04 | 0.006 | 1.088 (0.987-1.199) | 0.090 | 0.290 | 1.350 (1.163-1.566) | 7.64E-05 | 0.045 |
| IL1RN | 1.195 (1.111-1.286) | 1.68E-06 | 1.58E-04 | 1.229 (1.128-1.338) | 2.19E-06 | 3.76E-04 | 1.118 (0.970-1.287) | 0.123 | 0.866 |
| IL2RA | 1.166 (1.085-1.254) | 3.29E-05 | 0.001 | 1.142 (1.047-1.246) | 0.003 | 0.036 | 1.227 (1.073-1.403) | 0.003 | 0.272 |
| IL4R | 1.217 (1.137-1.303) | 1.37E-08 | 5.71E-06 | 1.239 (1.144-1.342) | 1.37E-07 | 5.69E-05 | 1.163 (1.023-1.322) | 0.021 | 0.567 |
| IL6 | 1.143 (1.066-1.226) | 1.71E-04 | 0.004 | 1.144 (1.056-1.241) | 0.001 | 0.020 | 1.146 (0.997-1.317) | 0.054 | 0.693 |
| ILKAP | 1.111 (1.035-1.192) | 0.003 | 0.035 | 1.134 (1.044-1.232) | 0.003 | 0.037 | 1.059 (0.926-1.210) | 0.401 | 0.992 |
| IMMT | 1.168 (1.089-1.253) | 1.40E-05 | 0.001 | 1.175 (1.084-1.273) | 0.000 | 0.003 | 1.150 (0.998-1.324) | 0.053 | 0.693 |
| INSL3 | 0.778 (0.670-0.903) | 0.001 | 0.014 | 0.760 (0.635-0.909) | 0.003 | 0.035 | 0.818 (0.624-1.073) | 0.147 | 0.887 |
| ITGA11 | 0.875 (0.812-0.943) | 4.98E-04 | 0.009 | 0.864 (0.790-0.945) | 0.001 | 0.023 | 0.901 (0.784-1.035) | 0.140 | 0.882 |
| ITGA5 | 1.116 (1.033-1.206) | 0.005 | 0.047 | 1.135 (1.036-1.245) | 0.007 | 0.064 | 1.068 (0.923-1.235) | 0.376 | 0.992 |
| ITGAV | 0.844 (0.781-0.911) | 1.44E-05 | 0.001 | 0.861 (0.785-0.944) | 0.001 | 0.024 | 0.806 (0.702-0.927) | 0.002 | 0.271 |
| ITGBL1 | 1.159 (1.071-1.255) | 2.63E-04 | 0.006 | 1.194 (1.088-1.311) | 0.000 | 0.005 | 1.075 (0.924-1.251) | 0.349 | 0.992 |
| ITIH3 | 1.165 (1.074-1.263) | 2.23E-04 | 0.005 | 1.180 (1.070-1.300) | 0.001 | 0.017 | 1.132 (0.978-1.309) | 0.097 | 0.838 |
| KIR3DL1 | 1.109 (1.031-1.192) | 0.006 | 0.049 | 1.081 (0.991-1.179) | 0.078 | 0.269 | 1.178 (1.028-1.351) | 0.018 | 0.550 |
| KLK4 | 1.138 (1.055-1.226) | 0.001 | 0.012 | 1.146 (1.049-1.252) | 0.003 | 0.034 | 1.112 (0.965-1.282) | 0.142 | 0.887 |
| KLRB1 | 1.140 (1.060-1.228) | 4.66E-04 | 0.008 | 1.201 (1.101-1.310) | 0.000 | 0.002 | 1.006 (0.875-1.156) | 0.938 | 0.993 |
| KRT18 | 1.117 (1.034-1.208) | 0.005 | 0.045 | 1.134 (1.033-1.244) | 0.008 | 0.073 | 1.086 (0.941-1.253) | 0.259 | 0.992 |
| KRT19 | 1.186 (1.103-1.275) | 3.58E-06 | 2.82E-04 | 1.202 (1.103-1.310) | 2.87E-05 | 0.002 | 1.153 (1.010-1.315) | 0.035 | 0.628 |
| LAIR1 | 1.132 (1.059-1.211) | 2.82E-04 | 0.006 | 1.154 (1.068-1.247) | 0.000 | 0.007 | 1.071 (0.935-1.226) | 0.321 | 0.992 |
| LAMP3 | 1.147 (1.059-1.242) | 0.001 | 0.012 | 1.148 (1.043-1.262) | 0.005 | 0.050 | 1.146 (0.992-1.324) | 0.065 | 0.747 |
| LEP | 1.311 (1.145-1.501) | 8.68E-05 | 0.003 | 1.285 (1.096-1.508) | 0.002 | 0.029 | 1.376 (1.067-1.775) | 0.014 | 0.470 |
| LETM1 | 1.129 (1.044-1.221) | 0.002 | 0.025 | 1.114 (1.014-1.223) | 0.024 | 0.139 | 1.164 (1.008-1.344) | 0.038 | 0.644 |
| LGALS4 | 1.220 (1.133-1.314) | 1.41E-07 | 3.17E-05 | 1.242 (1.138-1.356) | 1.19E-06 | 2.31E-04 | 1.168 (1.015-1.343) | 0.030 | 0.619 |
| LGALS9 | 1.196 (1.101-1.299) | 2.10E-05 | 0.001 | 1.219 (1.105-1.345) | 0.000 | 0.003 | 1.141 (0.980-1.329) | 0.089 | 0.820 |
| LILRA5 | 1.193 (1.102-1.291) | 1.26E-05 | 0.001 | 1.211 (1.103-1.331) | 0.000 | 0.003 | 1.144 (0.989-1.324) | 0.070 | 0.794 |
| LMNB2 | 1.159 (1.067-1.259) | 4.53E-04 | 0.008 | 1.170 (1.061-1.290) | 0.002 | 0.026 | 1.128 (0.965-1.318) | 0.129 | 0.870 |
| LRG1 | 1.158 (1.065-1.259) | 0.001 | 0.01 | 1.164 (1.052-1.288) | 0.003 | 0.039 | 1.144 (0.987-1.327) | 0.075 | 0.803 |
| LRIG1 | 1.148 (1.066-1.237) | 2.65E-04 | 0.006 | 1.152 (1.054-1.260) | 0.002 | 0.028 | 1.143 (0.998-1.309) | 0.053 | 0.693 |
| LRRN1 | 0.857 (0.794-0.925) | 7.37E-05 | 0.002 | 0.861 (0.786-0.944) | 0.001 | 0.023 | 0.846 (0.735-0.974) | 0.020 | 0.559 |
| LTBP3 | 1.177 (1.105-1.254) | 4.30E-07 | 6.60E-05 | 1.156 (1.072-1.247) | 1.79E-04 | 0.005 | 1.235 (1.100-1.386) | 3.37E-04 | 0.076 |
| MAD1L1 | 1.140 (1.068-1.216) | 7.37E-05 | 0.002 | 1.174 (1.090-1.265) | 0.000 | 0.002 | 1.059 (0.925-1.212) | 0.409 | 0.992 |
| MAP1LC3A | 0.881 (0.812-0.956) | 0.002 | 0.026 | 0.872 (0.790-0.962) | 0.006 | 0.062 | 0.903 (0.779-1.046) | 0.173 | 0.915 |
| MARCO | 1.135 (1.050-1.227) | 0.001 | 0.017 | 1.151 (1.049-1.263) | 0.003 | 0.037 | 1.097 (0.952-1.265) | 0.201 | 0.936 |
| MDK | 1.143 (1.065-1.227) | 2.08E-04 | 0.005 | 1.167 (1.075-1.266) | 0.000 | 0.006 | 1.083 (0.943-1.244) | 0.261 | 0.992 |
| MEP1B | 0.861 (0.801-0.926) | 5.05E-05 | 0.002 | 0.890 (0.816-0.971) | 0.009 | 0.077 | 0.800 (0.702-0.911) | 0.001 | 0.124 |
| MERTK | 1.132 (1.050-1.221) | 0.001 | 0.016 | 1.123 (1.026-1.230) | 0.012 | 0.096 | 1.156 (1.008-1.325) | 0.038 | 0.644 |
| METAP1D | 1.115 (1.034-1.204) | 0.005 | 0.044 | 1.118 (1.021-1.224) | 0.016 | 0.113 | 1.107 (0.963-1.274) | 0.154 | 0.897 |
| MILR1 | 1.131 (1.051-1.217) | 0.001 | 0.014 | 1.115 (1.023-1.215) | 0.013 | 0.104 | 1.172 (1.019-1.347) | 0.026 | 0.590 |
| MME | 1.130 (1.050-1.217) | 0.001 | 0.016 | 1.115 (1.021-1.218) | 0.016 | 0.112 | 1.165 (1.017-1.334) | 0.028 | 0.599 |
| MMP3 | 1.152 (1.052-1.261) | 0.002 | 0.025 | 1.114 (0.999-1.242) | 0.053 | 0.215 | 1.238 (1.056-1.452) | 0.009 | 0.410 |
| MMP7 | 1.142 (1.052-1.239) | 0.001 | 0.018 | 1.203 (1.092-1.327) | 0.000 | 0.006 | 1.012 (0.874-1.173) | 0.869 | 0.993 |
| MMP9 | 1.137 (1.053-1.227) | 0.001 | 0.015 | 1.116 (1.018-1.223) | 0.019 | 0.125 | 1.191 (1.036-1.370) | 0.014 | 0.470 |
| MPO | 1.123 (1.049-1.202) | 0.001 | 0.013 | 1.119 (1.030-1.216) | 0.008 | 0.072 | 1.137 (1.007-1.284) | 0.039 | 0.644 |
| MRC1 | 1.213 (1.118-1.315) | 3.18E-06 | 2.58E-04 | 1.198 (1.085-1.323) | 3.60E-04 | 0.008 | 1.237 (1.076-1.423) | 0.003 | 0.272 |
| MSR1 | 1.136 (1.047-1.233) | 0.002 | 0.025 | 1.108 (1.005-1.221) | 0.039 | 0.182 | 1.210 (1.041-1.406) | 0.013 | 0.468 |
| MVK | 1.167 (1.081-1.258) | 6.80E-05 | 0.002 | 1.189 (1.086-1.301) | 0.000 | 0.005 | 1.115 (0.970-1.282) | 0.127 | 0.870 |
| MYBPC2 | 1.126 (1.049-1.210) | 0.001 | 0.015 | 1.098 (1.004-1.201) | 0.041 | 0.188 | 1.185 (1.052-1.335) | 0.005 | 0.382 |
| NAGPA | 1.133 (1.041-1.232) | 0.004 | 0.036 | 1.113 (1.006-1.232) | 0.038 | 0.180 | 1.179 (1.014-1.372) | 0.032 | 0.627 |
| NCR3LG1 | 1.145 (1.052-1.246) | 0.002 | 0.02 | 1.132 (1.022-1.253) | 0.017 | 0.118 | 1.172 (1.008-1.363) | 0.039 | 0.644 |
| NCS1 | 1.147 (1.058-1.243) | 0.001 | 0.013 | 1.113 (1.011-1.226) | 0.029 | 0.155 | 1.228 (1.060-1.422) | 0.006 | 0.394 |
| NDUFA5 | 1.128 (1.040-1.223) | 0.004 | 0.036 | 1.159 (1.052-1.278) | 0.003 | 0.037 | 1.059 (0.914-1.225) | 0.447 | 0.992 |
| NECTIN2 | 1.177 (1.095-1.264) | 8.98E-06 | 0.001 | 1.236 (1.136-1.344) | 0.000 | 1.94E-04 | 1.044 (0.910-1.198) | 0.539 | 0.992 |
| NHLRC3 | 1.202 (1.102-1.312) | 3.51E-05 | 0.001 | 1.229 (1.107-1.364) | 0.000 | 0.004 | 1.141 (0.972-1.338) | 0.106 | 0.838 |
| NID1 | 1.147 (1.065-1.234) | 2.76E-04 | 0.006 | 1.115 (1.021-1.218) | 0.015 | 0.109 | 1.219 (1.066-1.394) | 0.004 | 0.317 |
| NPC2 | 1.150 (1.062-1.245) | 0.001 | 0.009 | 1.174 (1.066-1.294) | 0.001 | 0.021 | 1.097 (0.952-1.263) | 0.201 | 0.936 |
| NPDC1 | 1.131 (1.052-1.215) | 0.001 | 0.013 | 1.144 (1.050-1.247) | 0.002 | 0.031 | 1.101 (0.963-1.258) | 0.160 | 0.897 |
| NT5C1A | 1.124 (1.048-1.205) | 0.001 | 0.015 | 1.140 (1.050-1.237) | 0.002 | 0.027 | 1.081 (0.946-1.235) | 0.255 | 0.992 |
| NTproBNP | 1.144 (1.060-1.235) | 0.001 | 0.01 | 1.108 (1.010-1.215) | 0.030 | 0.157 | 1.224 (1.069-1.402) | 0.003 | 0.295 |
| NTRK3 | 0.866 (0.802-0.936) | 3.02E-04 | 0.006 | 0.865 (0.789-0.949) | 0.002 | 0.030 | 0.869 (0.752-1.003) | 0.055 | 0.693 |
| NUCB2 | 1.112 (1.032-1.199) | 0.005 | 0.046 | 1.117 (1.022-1.221) | 0.015 | 0.109 | 1.099 (0.958-1.261) | 0.179 | 0.916 |
| OCLN | 1.210 (1.114-1.313) | 5.52E-06 | 3.93E-04 | 1.225 (1.110-1.351) | 5.10E-05 | 0.002 | 1.176 (1.012-1.367) | 0.035 | 0.628 |
| OLR1 | 1.131 (1.058-1.210) | 3.05E-04 | 0.006 | 1.137 (1.048-1.233) | 0.002 | 0.028 | 1.126 (0.999-1.270) | 0.053 | 0.693 |
| OMG | 0.878 (0.814-0.948) | 0.001 | 0.013 | 0.845 (0.771-0.926) | 0.000 | 0.007 | 0.963 (0.837-1.108) | 0.596 | 0.992 |
| OSCAR | 1.118 (1.036-1.207) | 0.004 | 0.039 | 1.127 (1.030-1.234) | 0.009 | 0.080 | 1.096 (0.950-1.265) | 0.208 | 0.936 |
| OSM | 1.162 (1.077-1.253) | 1.12E-04 | 0.003 | 1.144 (1.045-1.252) | 0.004 | 0.043 | 1.212 (1.053-1.394) | 0.007 | 0.406 |
| OSMR | 1.165 (1.082-1.253) | 4.55E-05 | 0.002 | 1.146 (1.050-1.250) | 0.002 | 0.031 | 1.213 (1.059-1.391) | 0.005 | 0.382 |
| OXCT1 | 1.138 (1.054-1.230) | 0.001 | 0.014 | 1.173 (1.069-1.286) | 0.001 | 0.014 | 1.060 (0.919-1.223) | 0.423 | 0.992 |
| PAG1 | 1.146 (1.068-1.229) | 1.49E-04 | 0.004 | 1.190 (1.097-1.291) | 0.000 | 0.002 | 1.042 (0.907-1.196) | 0.562 | 0.992 |
| PALM2 | 1.177 (1.080-1.282) | 1.98E-04 | 0.005 | 1.181 (1.066-1.309) | 0.001 | 0.025 | 1.163 (0.996-1.359) | 0.057 | 0.704 |
| PDZK1 | 1.142 (1.059-1.231) | 0.001 | 0.009 | 1.165 (1.070-1.269) | 0.000 | 0.010 | 1.081 (0.930-1.257) | 0.312 | 0.992 |
| PGF | 1.115 (1.037-1.200) | 0.003 | 0.035 | 1.129 (1.036-1.229) | 0.006 | 0.057 | 1.081 (0.940-1.245) | 0.275 | 0.992 |
| PI3 | 1.143 (1.062-1.229) | 3.62E-04 | 0.007 | 1.125 (1.031-1.227) | 0.008 | 0.073 | 1.188 (1.036-1.362) | 0.014 | 0.470 |
| PIGR | 1.223 (1.136-1.316) | 7.92E-08 | 2.31E-05 | 1.224 (1.123-1.333) | 4.01E-06 | 0.001 | 1.213 (1.053-1.397) | 0.007 | 0.408 |
| PKD1 | 1.141 (1.045-1.246) | 0.003 | 0.033 | 1.154 (1.040-1.282) | 0.007 | 0.068 | 1.105 (0.940-1.299) | 0.226 | 0.957 |
| PLA2G15 | 1.210 (1.125-1.303) | 3.59E-07 | 5.93E-05 | 1.248 (1.146-1.360) | 4.18E-07 | 1.53E-04 | 1.110 (0.964-1.279) | 0.148 | 0.887 |
| PLAUR | 1.264 (1.171-1.364) | 2.04E-09 | 1.41E-06 | 1.259 (1.149-1.379) | 7.82E-07 | 1.94E-04 | 1.275 (1.107-1.468) | 0.001 | 0.124 |
| PLIN3 | 1.128 (1.049-1.214) | 0.001 | 0.016 | 1.140 (1.044-1.245) | 0.004 | 0.042 | 1.105 (0.969-1.261) | 0.137 | 0.882 |
| PLXNB2 | 1.121 (1.042-1.207) | 0.002 | 0.025 | 1.143 (1.048-1.247) | 0.003 | 0.034 | 1.066 (0.928-1.225) | 0.363 | 0.992 |
| POF1B | 1.120 (1.043-1.202) | 0.002 | 0.021 | 1.142 (1.054-1.238) | 0.001 | 0.022 | 1.058 (0.919-1.218) | 0.432 | 0.992 |
| POLR2F | 1.123 (1.051-1.200) | 0.001 | 0.01 | 1.174 (1.092-1.263) | 0.000 | 0.001 | 0.981 (0.844-1.140) | 0.801 | 0.992 |
| PON1 | 0.878 (0.822-0.938) | 1.03E-04 | 0.003 | 0.861 (0.796-0.932) | 0.000 | 0.006 | 0.921 (0.811-1.046) | 0.203 | 0.936 |
| PRAP1 | 1.173 (1.068-1.289) | 0.001 | 0.013 | 1.221 (1.090-1.369) | 0.001 | 0.012 | 1.076 (0.910-1.272) | 0.392 | 0.992 |
| PRSS8 | 1.190 (1.096-1.292) | 3.59E-05 | 0.001 | 1.173 (1.064-1.294) | 0.001 | 0.023 | 1.228 (1.053-1.432) | 0.009 | 0.410 |
| PRTN3 | 1.144 (1.076-1.216) | 1.81E-05 | 0.001 | 1.153 (1.071-1.240) | 0.000 | 0.005 | 1.128 (1.008-1.264) | 0.036 | 0.639 |
| PSAP | 1.128 (1.054-1.207) | 0.001 | 0.009 | 1.166 (1.072-1.269) | 0.000 | 0.008 | 1.061 (0.937-1.203) | 0.350 | 0.992 |
| PTPRF | 0.885 (0.820-0.956) | 0.002 | 0.022 | 0.878 (0.802-0.962) | 0.005 | 0.054 | 0.900 (0.781-1.038) | 0.147 | 0.887 |
| PTX3 | 1.151 (1.060-1.250) | 0.001 | 0.013 | 1.119 (1.015-1.234) | 0.024 | 0.138 | 1.227 (1.052-1.430) | 0.009 | 0.410 |
| QPCT | 1.117 (1.036-1.204) | 0.004 | 0.038 | 1.149 (1.052-1.255) | 0.002 | 0.029 | 1.043 (0.905-1.203) | 0.557 | 0.992 |
| QSOX1 | 1.095 (1.032-1.162) | 0.003 | 0.03 | 1.074 (0.995-1.159) | 0.067 | 0.248 | 1.140 (1.035-1.256) | 0.008 | 0.410 |
| RAB6A | 0.896 (0.831-0.966) | 0.004 | 0.038 | 0.911 (0.834-0.996) | 0.041 | 0.189 | 0.857 (0.746-0.985) | 0.030 | 0.619 |
| RARRES2 | 1.165 (1.076-1.262) | 1.57E-04 | 0.004 | 1.184 (1.077-1.303) | 0.000 | 0.011 | 1.122 (0.970-1.298) | 0.120 | 0.863 |
| RBFOX3 | 1.199 (1.105-1.300) | 1.20E-05 | 0.001 | 1.235 (1.123-1.359) | 0.000 | 0.001 | 1.113 (0.951-1.302) | 0.182 | 0.916 |
| RBP5 | 1.142 (1.055-1.237) | 0.001 | 0.015 | 1.148 (1.043-1.263) | 0.005 | 0.052 | 1.132 (0.979-1.308) | 0.095 | 0.838 |
| REN | 1.143 (1.065-1.227) | 2.19E-04 | 0.005 | 1.207 (1.111-1.311) | 0.000 | 0.001 | 0.994 (0.865-1.141) | 0.928 | 0.993 |
| RNASE1 | 1.128 (1.047-1.214) | 0.001 | 0.018 | 1.112 (1.015-1.218) | 0.022 | 0.136 | 1.160 (1.019-1.322) | 0.025 | 0.586 |
| RNASE4 | 1.131 (1.050-1.219) | 0.001 | 0.016 | 1.147 (1.043-1.260) | 0.005 | 0.050 | 1.112 (0.979-1.263) | 0.103 | 0.838 |
| RNASE6 | 1.157 (1.083-1.235) | 1.37E-05 | 0.001 | 1.164 (1.077-1.259) | 0.000 | 0.005 | 1.148 (1.017-1.296) | 0.026 | 0.590 |
| RNASET2 | 1.151 (1.074-1.234) | 7.25E-05 | 0.002 | 1.179 (1.089-1.276) | 0.000 | 0.002 | 1.077 (0.938-1.236) | 0.292 | 0.992 |
| RNF149 | 1.155 (1.066-1.251) | 4.19E-04 | 0.008 | 1.172 (1.064-1.291) | 0.001 | 0.023 | 1.114 (0.965-1.286) | 0.141 | 0.883 |
| RPS10 | 0.854 (0.775-0.941) | 0.001 | 0.018 | 0.772 (0.682-0.873) | 0.000 | 0.002 | 1.022 (0.887-1.177) | 0.764 | 0.992 |
| RRM2 | 1.105 (1.041-1.173) | 0.001 | 0.015 | 1.108 (1.034-1.188) | 0.004 | 0.044 | 1.098 (0.974-1.237) | 0.126 | 0.868 |
| SCARB2 | 1.206 (1.118-1.302) | 1.54E-06 | 1.54E-04 | 1.207 (1.103-1.321) | 4.23E-05 | 0.002 | 1.202 (1.039-1.391) | 0.013 | 0.468 |
| SCARF1 | 1.123 (1.044-1.207) | 0.002 | 0.022 | 1.162 (1.065-1.267) | 0.001 | 0.014 | 1.034 (0.905-1.181) | 0.621 | 0.992 |
| SCGB1A1 | 0.853 (0.793-0.918) | 2.38E-05 | 0.001 | 0.836 (0.766-0.912) | 0.000 | 0.002 | 0.895 (0.779-1.027) | 0.114 | 0.856 |
| SCGN | 1.129 (1.049-1.215) | 0.001 | 0.016 | 1.137 (1.042-1.241) | 0.004 | 0.044 | 1.110 (0.968-1.273) | 0.136 | 0.882 |
| SCLY | 1.122 (1.039-1.212) | 0.003 | 0.035 | 1.140 (1.039-1.250) | 0.005 | 0.056 | 1.083 (0.940-1.248) | 0.272 | 0.992 |
| SELENOP | 0.869 (0.800-0.943) | 0.001 | 0.012 | 0.829 (0.751-0.916) | 0.000 | 0.006 | 0.961 (0.831-1.110) | 0.586 | 0.992 |
| SELP | 1.130 (1.050-1.215) | 0.001 | 0.015 | 1.130 (1.036-1.233) | 0.006 | 0.059 | 1.125 (0.983-1.286) | 0.086 | 0.820 |
| SERPINA11 | 1.138 (1.048-1.236) | 0.002 | 0.025 | 1.192 (1.080-1.316) | 0.000 | 0.011 | 1.021 (0.880-1.186) | 0.781 | 0.992 |
| SERPINA3 | 1.137 (1.064-1.214) | 1.37E-04 | 0.004 | 1.106 (1.010-1.211) | 0.030 | 0.159 | 1.170 (1.075-1.273) | 2.69E-04 | 0.066 |
| SIAE | 1.137 (1.055-1.225) | 0.001 | 0.012 | 1.165 (1.067-1.272) | 0.001 | 0.013 | 1.062 (0.922-1.224) | 0.403 | 0.992 |
| SIGLEC1 | 1.179 (1.091-1.275) | 3.54E-05 | 0.001 | 1.183 (1.079-1.298) | 0.000 | 0.008 | 1.166 (1.008-1.349) | 0.039 | 0.644 |
| SIGLEC10 | 1.164 (1.082-1.252) | 4.34E-05 | 0.002 | 1.163 (1.067-1.268) | 0.001 | 0.013 | 1.164 (1.016-1.334) | 0.029 | 0.599 |
| SIGLEC6 | 1.113 (1.038-1.194) | 0.003 | 0.029 | 1.131 (1.040-1.230) | 0.004 | 0.046 | 1.078 (0.946-1.229) | 0.261 | 0.992 |
| SIT1 | 1.131 (1.046-1.223) | 0.002 | 0.024 | 1.168 (1.065-1.281) | 0.001 | 0.019 | 1.045 (0.902-1.211) | 0.557 | 0.992 |
| SLAMF8 | 1.143 (1.055-1.237) | 0.001 | 0.014 | 1.126 (1.026-1.235) | 0.012 | 0.100 | 1.192 (1.023-1.388) | 0.024 | 0.573 |
| SMPD1 | 1.149 (1.068-1.236) | 2.02E-04 | 0.005 | 1.178 (1.079-1.286) | 0.000 | 0.007 | 1.082 (0.946-1.237) | 0.251 | 0.987 |
| SOD2 | 1.121 (1.044-1.203) | 0.002 | 0.02 | 1.094 (1.003-1.194) | 0.042 | 0.192 | 1.175 (1.041-1.326) | 0.009 | 0.410 |
| SORCS2 | 1.117 (1.034-1.206) | 0.005 | 0.043 | 1.157 (1.056-1.268) | 0.002 | 0.027 | 1.029 (0.892-1.187) | 0.696 | 0.992 |
| SORT1 | 1.116 (1.036-1.202) | 0.004 | 0.037 | 1.153 (1.056-1.259) | 0.001 | 0.024 | 1.027 (0.892-1.182) | 0.714 | 0.992 |
| SPINK1 | 1.113 (1.037-1.195) | 0.003 | 0.032 | 1.090 (1.000-1.189) | 0.050 | 0.209 | 1.163 (1.028-1.315) | 0.016 | 0.508 |
| SPINK4 | 1.116 (1.037-1.202) | 0.004 | 0.036 | 1.140 (1.044-1.244) | 0.003 | 0.041 | 1.058 (0.921-1.216) | 0.425 | 0.992 |
| SPON2 | 1.124 (1.040-1.214) | 0.003 | 0.032 | 1.181 (1.077-1.294) | 0.000 | 0.009 | 0.999 (0.866-1.153) | 0.994 | 1.000 |
| TF | 1.137 (1.050-1.232) | 0.002 | 0.019 | 1.150 (1.045-1.266) | 0.004 | 0.049 | 1.108 (0.960-1.279) | 0.161 | 0.897 |
| TFF2 | 1.151 (1.069-1.240) | 2.13E-04 | 0.005 | 1.162 (1.064-1.270) | 0.001 | 0.017 | 1.124 (0.978-1.292) | 0.100 | 0.838 |
| TFF3 | 1.143 (1.073-1.218) | 3.62E-05 | 0.001 | 1.130 (1.046-1.220) | 0.002 | 0.028 | 1.172 (1.048-1.311) | 0.006 | 0.382 |
| TGFA | 1.243 (1.158-1.334) | 1.74E-09 | 1.41E-06 | 1.276 (1.174-1.387) | 9.60E-09 | 7.00E-06 | 1.158 (1.013-1.324) | 0.031 | 0.620 |
| TGFB1 | 1.150 (1.072-1.234) | 9.88E-05 | 0.003 | 1.154 (1.062-1.253) | 0.001 | 0.014 | 1.138 (0.996-1.299) | 0.057 | 0.704 |
| THBS2 | 1.142 (1.066-1.223) | 1.46E-04 | 0.004 | 1.155 (1.065-1.252) | 0.000 | 0.011 | 1.112 (0.978-1.265) | 0.104 | 0.838 |
| TIMP1 | 1.199 (1.113-1.292) | 1.82E-06 | 1.60E-04 | 1.200 (1.097-1.313) | 7.06E-05 | 0.003 | 1.199 (1.048-1.371) | 0.008 | 0.410 |
| TMED10 | 0.848 (0.756-0.952) | 0.005 | 0.044 | 0.844 (0.735-0.969) | 0.016 | 0.112 | 0.861 (0.698-1.061) | 0.160 | 0.897 |
| TMED4 | 0.860 (0.780-0.949) | 0.003 | 0.029 | 0.860 (0.764-0.968) | 0.013 | 0.101 | 0.862 (0.722-1.028) | 0.099 | 0.838 |
| TMSB10 | 1.151 (1.065-1.245) | 3.79E-04 | 0.007 | 1.209 (1.102-1.326) | 0.000 | 0.003 | 1.027 (0.890-1.186) | 0.711 | 0.992 |
| TNC | 1.122 (1.044-1.207) | 0.002 | 0.022 | 1.108 (1.014-1.210) | 0.023 | 0.136 | 1.151 (1.012-1.309) | 0.033 | 0.627 |
| TNF | 1.120 (1.063-1.180) | 2.08E-05 | 0.001 | 1.114 (1.049-1.184) | 0.000 | 0.010 | 1.134 (1.022-1.259) | 0.018 | 0.549 |
| TNFRSF10A | 1.224 (1.145-1.308) | 2.42E-09 | 1.41E-06 | 1.250 (1.162-1.344) | 2.24E-09 | 2.18E-06 | 1.137 (0.986-1.312) | 0.077 | 0.812 |
| TNFRSF10B | 1.161 (1.113-1.211) | 5.60E-12 | 8.17E-09 | 1.163 (1.109-1.221) | 6.38E-10 | 9.31E-07 | 1.152 (1.050-1.264) | 0.003 | 0.272 |
| TNFRSF11A | 1.154 (1.071-1.244) | 1.82E-04 | 0.005 | 1.177 (1.078-1.286) | 0.000 | 0.007 | 1.095 (0.951-1.262) | 0.206 | 0.936 |
| TNFRSF11B | 1.160 (1.072-1.255) | 2.28E-04 | 0.005 | 1.159 (1.056-1.272) | 0.002 | 0.028 | 1.158 (0.999-1.343) | 0.051 | 0.693 |
| TNFRSF12A | 1.144 (1.058-1.237) | 0.001 | 0.012 | 1.195 (1.091-1.309) | 0.000 | 0.005 | 1.027 (0.886-1.192) | 0.722 | 0.992 |
| TNFRSF14 | 1.178 (1.096-1.267) | 8.82E-06 | 0.001 | 1.210 (1.111-1.317) | 0.000 | 0.001 | 1.100 (0.958-1.263) | 0.176 | 0.916 |
| TNFRSF1A | 1.220 (1.130-1.317) | 3.66E-07 | 5.93E-05 | 1.248 (1.141-1.365) | 1.28E-06 | 2.34E-04 | 1.152 (0.994-1.334) | 0.060 | 0.721 |
| TNFRSF1B | 1.152 (1.095-1.213) | 6.22E-08 | 2.01E-05 | 1.163 (1.096-1.235) | 7.82E-07 | 1.94E-04 | 1.123 (1.016-1.241) | 0.023 | 0.567 |
| TNFRSF4 | 1.138 (1.058-1.224) | 0.001 | 0.009 | 1.206 (1.108-1.313) | 0.000 | 0.001 | 0.978 (0.847-1.128) | 0.758 | 0.992 |
| TNFRSF6B | 1.151 (1.068-1.241) | 2.47E-04 | 0.006 | 1.194 (1.093-1.304) | 0.000 | 0.003 | 1.053 (0.912-1.215) | 0.481 | 0.992 |
| TNFRSF8 | 1.138 (1.059-1.223) | 4.56E-04 | 0.008 | 1.184 (1.088-1.288) | 0.000 | 0.003 | 1.027 (0.894-1.180) | 0.707 | 0.992 |
| TNFRSF9 | 1.140 (1.060-1.226) | 4.22E-04 | 0.008 | 1.184 (1.091-1.286) | 0.000 | 0.003 | 1.017 (0.877-1.180) | 0.821 | 0.992 |
| TNFSF11 | 0.827 (0.766-0.892) | 9.93E-07 | 1.11E-04 | 0.828 (0.756-0.906) | 4.13E-05 | 0.002 | 0.827 (0.718-0.952) | 0.008 | 0.410 |
| TNFSF13 | 1.191 (1.104-1.285) | 6.21E-06 | 4.09E-04 | 1.163 (1.063-1.273) | 1.06E-03 | 0.020 | 1.255 (1.091-1.444) | 0.001 | 0.179 |
| TNFSF13B | 1.123 (1.041-1.212) | 0.003 | 0.028 | 1.107 (1.012-1.212) | 0.027 | 0.149 | 1.166 (1.012-1.343) | 0.033 | 0.628 |
| TNFSF14 | 1.119 (1.038-1.206) | 0.003 | 0.033 | 1.101 (1.007-1.204) | 0.034 | 0.167 | 1.160 (1.011-1.331) | 0.035 | 0.628 |
| TNR | 0.874 (0.808-0.945) | 0.001 | 0.012 | 0.894 (0.814-0.981) | 0.019 | 0.123 | 0.829 (0.718-0.957) | 0.010 | 0.433 |
| TP53INP1 | 0.852 (0.776-0.934) | 0.001 | 0.011 | 0.841 (0.753-0.940) | 0.002 | 0.031 | 0.878 (0.741-1.039) | 0.129 | 0.870 |
| TPP1 | 1.118 (1.043-1.197) | 0.002 | 0.019 | 1.149 (1.062-1.244) | 0.001 | 0.012 | 1.030 (0.898-1.182) | 0.673 | 0.992 |
| TYMP | 1.164 (1.079-1.255) | 8.47E-05 | 0.003 | 1.185 (1.082-1.297) | 0.000 | 0.006 | 1.113 (0.969-1.278) | 0.129 | 0.870 |
| UBE2L6 | 1.127 (1.039-1.223) | 0.004 | 0.038 | 1.126 (1.021-1.242) | 0.017 | 0.119 | 1.124 (0.970-1.303) | 0.119 | 0.861 |
| UMOD | 0.837 (0.779-0.900) | 1.34E-06 | 1.45E-04 | 0.829 (0.762-0.902) | 1.45E-05 | 0.001 | 0.862 (0.752-0.987) | 0.032 | 0.627 |
| VCAM1 | 1.144 (1.065-1.228) | 2.33E-04 | 0.005 | 1.125 (1.033-1.226) | 0.007 | 0.066 | 1.185 (1.040-1.349) | 0.011 | 0.433 |
| VEGFA | 1.117 (1.038-1.202) | 0.003 | 0.032 | 1.100 (1.007-1.201) | 0.035 | 0.168 | 1.155 (1.012-1.318) | 0.033 | 0.627 |
| VNN2 | 1.168 (1.079-1.265) | 1.21E-04 | 0.003 | 1.111 (1.012-1.219) | 0.027 | 0.149 | 1.323 (1.138-1.538) | 2.72E-04 | 0.066 |
| VSIG2 | 1.217 (1.127-1.314) | 5.17E-07 | 7.18E-05 | 1.219 (1.111-1.337) | 2.64E-05 | 0.002 | 1.210 (1.053-1.389) | 0.007 | 0.405 |
| VSIG4 | 1.198 (1.108-1.296) | 6.16E-06 | 4.09E-04 | 1.162 (1.057-1.277) | 1.78E-03 | 0.027 | 1.283 (1.115-1.477) | 0.001 | 0.104 |
| VSIR | 1.108 (1.032-1.189) | 0.005 | 0.042 | 1.117 (1.027-1.214) | 0.010 | 0.083 | 1.082 (0.948-1.235) | 0.243 | 0.977 |
| VWA1 | 1.121 (1.038-1.210) | 0.004 | 0.036 | 1.149 (1.049-1.259) | 0.003 | 0.037 | 1.054 (0.914-1.216) | 0.466 | 0.992 |
| VWF | 1.115 (1.035-1.201) | 0.004 | 0.04 | 1.157 (1.060-1.265) | 0.001 | 0.022 | 1.015 (0.884-1.166) | 0.827 | 0.993 |
| WFDC2 | 1.181 (1.092-1.277) | 3.03E-05 | 0.001 | 1.215 (1.108-1.334) | 0.000 | 0.002 | 1.108 (0.956-1.283) | 0.173 | 0.915 |
| WFIKKN1 | 0.863 (0.796-0.935) | 3.35E-04 | 0.007 | 0.839 (0.763-0.922) | 0.000 | 0.007 | 0.924 (0.795-1.075) | 0.307 | 0.992 |
| WNT9A | 1.122 (1.038-1.214) | 0.004 | 0.037 | 1.125 (1.026-1.234) | 0.012 | 0.098 | 1.114 (0.959-1.293) | 0.158 | 0.897 |
| XCL1 | 1.130 (1.056-1.210) | 4.31E-04 | 0.008 | 1.179 (1.091-1.273) | 0.000 | 0.002 | 1.005 (0.874-1.155) | 0.947 | 0.993 |
| YAP1 | 1.166 (1.069-1.271) | 0.001 | 0.009 | 1.162 (1.046-1.291) | 0.005 | 0.055 | 1.168 (1.003-1.360) | 0.046 | 0.693 |
| ZNRD2 | 1.124 (1.040-1.215) | 0.003 | 0.032 | 1.133 (1.032-1.244) | 0.009 | 0.078 | 1.106 (0.961-1.272) | 0.159 | 0.897 |

| **Supplementary Table 31. Consistency of protein effect estimates across primary and sensitivity analyses.** | | | | | |
| --- | --- | --- | --- | --- | --- |
| **Association** | **Sensitivity analysis** | **N** | **Pearson *r*** | **Directional concordance (%)** | **Remained significant at FDR<0.05** |
| Workplace noise–protein | Lipid-lowering medication users | 3122 | 0.911 | 98.3 | 21/229 (9.2%) |
|  | No lipid-lowering medications | 15214 | 0.984 | 100 | 106/229 (46.3%) |
|  | Psychotropic medication users | 1892 | 0.764 | 90.0 | 0/229 (0%) |
|  | No psychotropic medications | 16444 | 0.996 | 100 | 166/229 (72.5%) |
|  | Additional adjustment for hypertension, diabetes mellitus, CVD, and CKD | 18359 | 1.000 | 100 | 222/229 (96.9%) |
| Protein–anxiety disorder | Lipid-lowering medication users | 2647 | 0.848 | 99.6 | 40/239 (16.7%) |
|  | No lipid-lowering medications | 13209 | 0.975 | 100 | 32/239 (13.4%) |
|  | Psychotropic medication users | 897 | 0.516 | 89.1 | 1/239 (0.4%) |
|  | No psychotropic medications | 14959 | 0.917 | 100 | 22/239 (9.2%) |
|  | Additional adjustment for hypertension, diabetes mellitus, CVD, and CKD | 15876 | 0.992 | 100 | 130/239 (54.4%) |
| Protein–MDD | Lipid-lowering medication users | 2645 | 0.789 | 97.1 | 16/343 (4.7%) |
|  | No lipid-lowering medications | 13016 | 0.973 | 100 | 150/343 (43.7%) |
|  | Psychotropic medication users | 816 | 0.567 | 89.8 | 0/343 (0%) |
|  | No psychotropic medications | 14845 | 0.971 | 100 | 105/343 (30.6%) |
|  | Additional adjustment for hypertension, diabetes mellitus, CVD, and CKD | 15680 | 0.992 | 100 | 237/343 (69.1%) |

| **Supplementary Table 32. Detailed associations between workplace noise exposure and plasma proteins in the primary and sensitivity analyses.** | | | | | | | | | | | | |
| --- | --- | --- | --- | --- | --- | --- | --- | --- | --- | --- | --- | --- |
| **Protein** | **Primary analysis** | | **Lipid-lowering medication users** | | **No lipid-lowering medications** | | **Psychotropic medication users** | | **No psychotropic medications** | | **Additional adjustment** | |
|  | **Beta** | **FDR** | **Beta** | **FDR** | **Beta** | **FDR** | **Beta** | **FDR** | **Beta** | **FDR** | **Beta** | **FDR** |
| AARSD1 | -0.022 | 0.046 | -0.045 | 0.195 | -0.015 | 0.410 | 0.012 | 1.000 | -0.026 | 0.025 | -0.022 | 0.050 |
| ACE2 | 0.024 | 0.014 | 0.035 | 0.364 | 0.017 | 0.287 | 0.009 | 1.000 | 0.024 | 0.022 | 0.022 | 0.021 |
| ACHE | -0.027 | 0.015 | -0.053 | 0.112 | -0.020 | 0.270 | 0.008 | 1.000 | -0.032 | 0.008 | -0.028 | 0.014 |
| ACP1 | -0.029 | 0.016 | -0.058 | 0.080 | -0.017 | 0.400 | 0.006 | 1.000 | -0.032 | 0.011 | -0.028 | 0.020 |
| ACP5 | 0.033 | 0.001 | 0.044 | 0.214 | 0.032 | 0.009 | 0.004 | 1.000 | 0.036 | 0.001 | 0.035 | 3.10E-04 |
| ACRV1 | 0.020 | 0.007 | 0.020 | 0.555 | 0.019 | 0.064 | 0.050 | 0.896 | 0.017 | 0.055 | 0.020 | 0.008 |
| ADA2 | 0.026 | 0.013 | 0.041 | 0.254 | 0.019 | 0.245 | 0.001 | 1.000 | 0.028 | 0.014 | 0.026 | 0.015 |
| ADAM22 | -0.035 | 3.33E-04 | -0.028 | 0.515 | -0.038 | 0.002 | -0.039 | 1.000 | -0.035 | 0.001 | -0.034 | 4.89E-04 |
| ADGRG1 | 0.027 | 0.005 | 0.033 | 0.367 | 0.017 | 0.308 | -0.003 | 1.000 | 0.030 | 0.003 | 0.026 | 0.007 |
| AFP | -0.032 | 0.001 | -0.046 | 0.170 | -0.027 | 0.053 | 0.001 | 1.000 | -0.036 | 0.001 | -0.032 | 0.001 |
| ALDH3A1 | 0.032 | 0.001 | 0.026 | 0.545 | 0.034 | 0.007 | -0.007 | 1.000 | 0.036 | 0.001 | 0.032 | 0.001 |
| ALPP | 0.026 | 0.007 | 0.014 | 0.759 | 0.029 | 0.011 | 0.053 | 0.896 | 0.020 | 0.078 | 0.026 | 0.007 |
| AMBP | 0.028 | 0.003 | 0.033 | 0.416 | 0.025 | 0.053 | 0.001 | 1.000 | 0.031 | 0.002 | 0.029 | 0.001 |
| AMPD3 | -0.029 | 0.015 | -0.078 | 0.006 | -0.014 | 0.518 | -0.021 | 1.000 | -0.030 | 0.022 | -0.028 | 0.017 |
| ANGPTL4 | 0.024 | 0.024 | 0.024 | 0.581 | 0.022 | 0.140 | 0.041 | 1.000 | 0.021 | 0.089 | 0.024 | 0.022 |
| ANXA10 | 0.035 | 4.28E-04 | 0.031 | 0.451 | 0.033 | 0.009 | 0.008 | 1.000 | 0.036 | 0.001 | 0.034 | 0.001 |
| AREG | 0.031 | 0.002 | 0.036 | 0.367 | 0.025 | 0.093 | 0.026 | 1.000 | 0.030 | 0.010 | 0.030 | 0.003 |
| ARG1 | -0.028 | 0.008 | -0.052 | 0.094 | -0.021 | 0.218 | -0.039 | 1.000 | -0.027 | 0.022 | -0.028 | 0.008 |
| ARSA | 0.030 | 0.003 | 0.015 | 0.766 | 0.036 | 0.003 | 0.022 | 1.000 | 0.031 | 0.004 | 0.030 | 0.002 |
| ART3 | -0.029 | 0.001 | -0.030 | 0.435 | -0.029 | 0.011 | -0.038 | 1.000 | -0.028 | 0.006 | -0.028 | 0.002 |
| ASGR1 | 0.026 | 0.007 | 0.033 | 0.417 | 0.021 | 0.172 | 0.028 | 1.000 | 0.024 | 0.026 | 0.026 | 0.005 |
| BGLAP | 0.027 | 0.018 | 0.053 | 0.105 | 0.021 | 0.243 | 0.006 | 1.000 | 0.030 | 0.013 | 0.027 | 0.017 |
| BOC | -0.022 | 0.046 | -0.009 | 0.886 | -0.027 | 0.060 | 0.001 | 1.000 | -0.025 | 0.033 | -0.022 | 0.050 |
| BPIFA2 | -0.026 | 0.023 | -0.045 | 0.231 | -0.021 | 0.259 | -0.018 | 1.000 | -0.026 | 0.043 | -0.025 | 0.031 |
| BPIFB2 | 0.023 | 0.031 | 0.040 | 0.281 | 0.022 | 0.151 | 0.024 | 1.000 | 0.023 | 0.062 | 0.024 | 0.024 |
| BST2 | 0.049 | 6.35E-08 | 0.076 | 0.002 | 0.041 | 4.58E-04 | 0.053 | 0.987 | 0.047 | 1.53E-06 | 0.049 | 5.74E-08 |
| C1R | 0.030 | 0.008 | 0.033 | 0.435 | 0.033 | 0.020 | 0.025 | 1.000 | 0.030 | 0.015 | 0.031 | 0.006 |
| C1RL | 0.024 | 0.039 | 0.018 | 0.732 | 0.029 | 0.048 | 0.015 | 1.000 | 0.024 | 0.066 | 0.025 | 0.031 |
| C2 | 0.023 | 0.030 | 0.026 | 0.545 | 0.022 | 0.147 | 0.025 | 1.000 | 0.023 | 0.059 | 0.024 | 0.028 |
| C3 | 0.028 | 0.015 | 0.040 | 0.309 | 0.025 | 0.147 | 0.060 | 0.975 | 0.024 | 0.078 | 0.028 | 0.017 |
| C9orf40 | -0.028 | 0.019 | -0.065 | 0.039 | -0.016 | 0.422 | -0.006 | 1.000 | -0.031 | 0.017 | -0.027 | 0.022 |
| CA14 | -0.023 | 0.019 | 0.003 | 0.965 | -0.028 | 0.017 | -0.006 | 1.000 | -0.024 | 0.024 | -0.021 | 0.037 |
| CA6 | -0.033 | 4.28E-04 | -0.055 | 0.039 | -0.025 | 0.066 | -0.034 | 1.000 | -0.031 | 0.002 | -0.032 | 0.001 |
| CAT | -0.026 | 0.031 | -0.060 | 0.055 | -0.015 | 0.471 | -0.010 | 1.000 | -0.027 | 0.035 | -0.025 | 0.037 |
| CCL17 | 0.023 | 0.034 | 0.029 | 0.505 | 0.023 | 0.139 | 0.023 | 1.000 | 0.022 | 0.078 | 0.024 | 0.031 |
| CCL18 | 0.041 | 8.39E-06 | 0.037 | 0.337 | 0.044 | 6.87E-05 | 0.046 | 1.000 | 0.040 | 7.46E-05 | 0.042 | 4.98E-06 |
| CCL20 | 0.045 | 5.74E-07 | 0.056 | 0.046 | 0.040 | 0.001 | 0.020 | 1.000 | 0.048 | 1.07E-06 | 0.045 | 9.11E-07 |
| CCL21 | 0.027 | 0.009 | 0.030 | 0.461 | 0.024 | 0.104 | 0.029 | 1.000 | 0.026 | 0.028 | 0.027 | 0.009 |
| CCL22 | 0.030 | 0.002 | 0.037 | 0.334 | 0.029 | 0.026 | 0.029 | 1.000 | 0.029 | 0.008 | 0.030 | 0.002 |
| CCL5 | 0.029 | 0.006 | 0.034 | 0.418 | 0.028 | 0.034 | 0.008 | 1.000 | 0.031 | 0.006 | 0.029 | 0.005 |
| CD14 | 0.033 | 0.001 | 0.064 | 0.025 | 0.025 | 0.077 | 0.007 | 1.000 | 0.036 | 0.001 | 0.035 | 3.65E-04 |
| CD163 | 0.024 | 0.021 | 0.030 | 0.461 | 0.024 | 0.097 | 0.048 | 1.000 | 0.020 | 0.109 | 0.024 | 0.019 |
| CD27 | 0.024 | 0.024 | 0.029 | 0.463 | 0.020 | 0.236 | 0.034 | 1.000 | 0.021 | 0.084 | 0.025 | 0.016 |
| CD300LG | -0.024 | 0.023 | -0.028 | 0.496 | -0.021 | 0.184 | -0.007 | 1.000 | -0.026 | 0.020 | -0.021 | 0.047 |
| CD302 | 0.029 | 0.002 | 0.031 | 0.433 | 0.027 | 0.028 | 0.008 | 1.000 | 0.030 | 0.003 | 0.030 | 0.001 |
| CD72 | 0.024 | 0.037 | 0.038 | 0.356 | 0.019 | 0.283 | 0.025 | 1.000 | 0.024 | 0.073 | 0.025 | 0.031 |
| CD80 | 0.030 | 0.005 | 0.058 | 0.066 | 0.022 | 0.200 | 0.032 | 1.000 | 0.029 | 0.016 | 0.031 | 0.004 |
| CD83 | 0.034 | 3.36E-04 | 0.041 | 0.230 | 0.031 | 0.013 | 0.013 | 1.000 | 0.035 | 0.001 | 0.035 | 1.91E-04 |
| CD99L2 | 0.037 | 6.37E-05 | 0.038 | 0.296 | 0.041 | 2.52E-04 | 0.054 | 0.975 | 0.034 | 0.001 | 0.040 | 1.40E-05 |
| CDCP1 | 0.086 | 2.97E-32 | 0.112 | 2.08E-09 | 0.081 | 1.74E-21 | 0.061 | 0.896 | 0.088 | 2.43E-30 | 0.086 | 3.46E-32 |
| CDHR2 | -0.019 | 0.026 | -0.028 | 0.435 | -0.014 | 0.283 | 0.007 | 1.000 | -0.022 | 0.014 | -0.018 | 0.036 |
| CEACAM5 | 0.025 | 0.021 | -0.004 | 0.944 | 0.034 | 0.007 | 0.036 | 1.000 | 0.022 | 0.089 | 0.025 | 0.021 |
| CELA2A | -0.026 | 0.031 | -0.058 | 0.075 | -0.014 | 0.505 | -0.038 | 1.000 | -0.023 | 0.090 | -0.025 | 0.037 |
| CFH | 0.022 | 0.046 | 0.056 | 0.068 | 0.016 | 0.395 | 0.036 | 1.000 | 0.021 | 0.109 | 0.023 | 0.041 |
| CHGA | 0.033 | 0.002 | 0.021 | 0.664 | 0.033 | 0.016 | 0.024 | 1.000 | 0.033 | 0.006 | 0.032 | 0.002 |
| CHI3L1 | 0.024 | 0.018 | 0.032 | 0.444 | 0.020 | 0.205 | 0.038 | 1.000 | 0.022 | 0.072 | 0.025 | 0.017 |
| CHIT1 | 0.028 | 0.008 | 0.044 | 0.195 | 0.023 | 0.147 | 0.037 | 1.000 | 0.027 | 0.022 | 0.027 | 0.008 |
| CILP | -0.027 | 0.016 | -0.036 | 0.417 | -0.026 | 0.092 | -0.045 | 1.000 | -0.025 | 0.048 | -0.027 | 0.016 |
| CLEC4A | -0.033 | 0.001 | -0.040 | 0.289 | -0.030 | 0.023 | 0.003 | 1.000 | -0.037 | 0.001 | -0.032 | 0.001 |
| CNTN1 | -0.023 | 0.022 | -0.035 | 0.309 | -0.019 | 0.221 | -0.045 | 1.000 | -0.019 | 0.102 | -0.023 | 0.024 |
| CNTN5 | -0.034 | 1.77E-04 | -0.033 | 0.367 | -0.031 | 0.008 | -0.030 | 1.000 | -0.034 | 0.001 | -0.033 | 2.76E-04 |
| COL18A1 | 0.022 | 0.031 | 0.040 | 0.251 | 0.014 | 0.400 | -0.021 | 1.000 | 0.027 | 0.012 | 0.023 | 0.022 |
| COL1A1 | -0.023 | 0.035 | -0.006 | 0.921 | -0.028 | 0.041 | -0.041 | 1.000 | -0.021 | 0.108 | -0.023 | 0.041 |
| COL28A1 | -0.029 | 0.012 | -0.050 | 0.189 | -0.022 | 0.243 | -0.035 | 1.000 | -0.028 | 0.039 | -0.029 | 0.015 |
| COX5B | 0.024 | 0.034 | 0.027 | 0.555 | 0.023 | 0.147 | 0.030 | 1.000 | 0.023 | 0.078 | 0.024 | 0.028 |
| CRHBP | 0.024 | 0.018 | 0.038 | 0.301 | 0.021 | 0.182 | 0.042 | 1.000 | 0.021 | 0.083 | 0.025 | 0.012 |
| CTHRC1 | -0.033 | 3.36E-04 | -0.035 | 0.367 | -0.033 | 0.005 | -0.026 | 1.000 | -0.034 | 0.001 | -0.033 | 3.98E-04 |
| CTSD | 0.031 | 0.002 | 0.019 | 0.687 | 0.033 | 0.007 | 0.040 | 1.000 | 0.028 | 0.013 | 0.031 | 0.002 |
| CXCL12 | 0.024 | 0.034 | 0.037 | 0.363 | 0.022 | 0.182 | 0.024 | 1.000 | 0.024 | 0.061 | 0.024 | 0.033 |
| CXCL14 | 0.044 | 8.03E-07 | 0.019 | 0.672 | 0.050 | 1.52E-06 | 0.046 | 1.000 | 0.044 | 5.58E-06 | 0.044 | 9.11E-07 |
| CXCL17 | 0.039 | 5.74E-07 | 0.045 | 0.083 | 0.036 | 2.52E-04 | 0.002 | 1.000 | 0.042 | 3.15E-07 | 0.038 | 9.11E-07 |
| DCBLD2 | 0.027 | 0.008 | 0.026 | 0.545 | 0.019 | 0.241 | 0.026 | 1.000 | 0.026 | 0.023 | 0.025 | 0.016 |
| DLK1 | -0.022 | 0.041 | -0.006 | 0.919 | -0.026 | 0.053 | -0.043 | 1.000 | -0.020 | 0.113 | -0.021 | 0.062 |
| DNAJA4 | -0.035 | 0.002 | -0.070 | 0.025 | -0.024 | 0.167 | -0.003 | 1.000 | -0.039 | 0.001 | -0.035 | 0.002 |
| DPP7 | 0.030 | 0.003 | 0.033 | 0.429 | 0.032 | 0.013 | 0.012 | 1.000 | 0.032 | 0.004 | 0.031 | 0.002 |
| F7 | 0.022 | 0.046 | 0.026 | 0.545 | 0.023 | 0.118 | 0.019 | 1.000 | 0.021 | 0.082 | 0.024 | 0.022 |
| FCAR | 0.027 | 0.012 | 0.030 | 0.463 | 0.025 | 0.093 | 0.013 | 1.000 | 0.028 | 0.017 | 0.027 | 0.010 |
| FETUB | 0.029 | 0.004 | 0.055 | 0.049 | 0.023 | 0.127 | 0.025 | 1.000 | 0.028 | 0.011 | 0.029 | 0.003 |
| FGA | -0.026 | 0.007 | -0.035 | 0.316 | -0.024 | 0.077 | -0.043 | 1.000 | -0.024 | 0.026 | -0.025 | 0.009 |
| FGF23 | 0.033 | 4.32E-04 | 0.030 | 0.444 | 0.033 | 0.006 | 0.064 | 0.896 | 0.027 | 0.012 | 0.033 | 3.98E-04 |
| FRZB | 0.022 | 0.043 | 0.023 | 0.619 | 0.023 | 0.114 | 0.018 | 1.000 | 0.023 | 0.056 | 0.022 | 0.040 |
| GALNT2 | -0.026 | 0.013 | -0.022 | 0.647 | -0.025 | 0.088 | -0.014 | 1.000 | -0.027 | 0.017 | -0.025 | 0.020 |
| GAST | 0.045 | 6.91E-06 | 0.063 | 0.039 | 0.033 | 0.014 | 0.041 | 1.000 | 0.044 | 8.21E-05 | 0.043 | 1.48E-05 |
| GDF15 | 0.033 | 4.73E-05 | 0.028 | 0.451 | 0.030 | 0.005 | 0.021 | 1.000 | 0.033 | 2.82E-04 | 0.032 | 6.28E-05 |
| GDF2 | 0.026 | 0.007 | 0.034 | 0.367 | 0.025 | 0.056 | 0.008 | 1.000 | 0.028 | 0.007 | 0.027 | 0.004 |
| GFRAL | -0.038 | 3.33E-04 | -0.023 | 0.647 | -0.040 | 0.003 | -0.068 | 0.896 | -0.033 | 0.006 | -0.037 | 4.11E-04 |
| GGH | 0.024 | 0.021 | 0.041 | 0.235 | 0.019 | 0.218 | 0.022 | 1.000 | 0.023 | 0.054 | 0.024 | 0.019 |
| GIMAP7 | -0.024 | 0.048 | -0.024 | 0.629 | -0.023 | 0.205 | -0.011 | 1.000 | -0.026 | 0.062 | -0.024 | 0.060 |
| GLA | 0.029 | 0.009 | 0.020 | 0.704 | 0.030 | 0.031 | 0.027 | 1.000 | 0.028 | 0.023 | 0.029 | 0.009 |
| GP2 | -0.031 | 0.002 | -0.052 | 0.083 | -0.026 | 0.077 | -0.044 | 1.000 | -0.029 | 0.012 | -0.030 | 0.003 |
| GPNMB | 0.034 | 0.001 | 0.050 | 0.112 | 0.030 | 0.023 | 0.031 | 1.000 | 0.035 | 0.001 | 0.035 | 4.86E-04 |
| GPR15L | 0.035 | 4.83E-04 | 0.037 | 0.367 | 0.035 | 0.007 | 0.031 | 1.000 | 0.036 | 0.001 | 0.036 | 4.44E-04 |
| GPR37 | 0.033 | 0.001 | 0.033 | 0.429 | 0.030 | 0.018 | 0.006 | 1.000 | 0.035 | 0.001 | 0.033 | 0.001 |
| GUSB | 0.027 | 0.003 | 0.035 | 0.326 | 0.029 | 0.009 | -0.002 | 1.000 | 0.030 | 0.002 | 0.028 | 0.002 |
| GZMB | -0.023 | 0.033 | -0.015 | 0.759 | -0.024 | 0.115 | 0.006 | 1.000 | -0.026 | 0.025 | -0.023 | 0.043 |
| HAGH | -0.027 | 0.009 | -0.050 | 0.105 | -0.020 | 0.225 | -0.019 | 1.000 | -0.028 | 0.016 | -0.026 | 0.012 |
| HAVCR1 | 0.025 | 0.009 | 0.037 | 0.311 | 0.018 | 0.220 | 0.001 | 1.000 | 0.026 | 0.013 | 0.025 | 0.007 |
| HAVCR2 | 0.031 | 0.001 | 0.036 | 0.326 | 0.028 | 0.022 | 0.027 | 1.000 | 0.030 | 0.004 | 0.032 | 4.44E-04 |
| HBQ1 | -0.025 | 0.019 | -0.051 | 0.094 | -0.016 | 0.389 | -0.009 | 1.000 | -0.027 | 0.022 | -0.025 | 0.022 |
| HHEX | 0.026 | 0.024 | 0.036 | 0.418 | 0.025 | 0.119 | 0.024 | 1.000 | 0.026 | 0.055 | 0.027 | 0.019 |
| HMBS | -0.025 | 0.019 | -0.048 | 0.130 | -0.018 | 0.310 | -0.005 | 1.000 | -0.027 | 0.019 | -0.025 | 0.022 |
| HYAL1 | 0.034 | 0.001 | 0.023 | 0.621 | 0.033 | 0.010 | -0.002 | 1.000 | 0.037 | 0.001 | 0.033 | 0.001 |
| ICAM1 | 0.033 | 0.001 | 0.029 | 0.501 | 0.036 | 0.003 | 0.031 | 1.000 | 0.033 | 0.002 | 0.034 | 4.44E-04 |
| ICAM5 | 0.029 | 0.003 | 0.039 | 0.254 | 0.026 | 0.061 | 0.015 | 1.000 | 0.030 | 0.005 | 0.029 | 0.004 |
| IDUA | 0.032 | 0.001 | 0.036 | 0.367 | 0.031 | 0.019 | -0.013 | 1.000 | 0.037 | 0.001 | 0.032 | 0.001 |
| IFI30 | 0.024 | 0.036 | 0.010 | 0.879 | 0.027 | 0.066 | 0.053 | 1.000 | 0.018 | 0.216 | 0.023 | 0.040 |
| IGDCC4 | -0.025 | 0.024 | -0.043 | 0.254 | -0.017 | 0.335 | -0.029 | 1.000 | -0.024 | 0.064 | -0.024 | 0.037 |
| IL12RB1 | 0.025 | 0.023 | 0.046 | 0.189 | 0.017 | 0.334 | 0.010 | 1.000 | 0.026 | 0.027 | 0.025 | 0.020 |
| IL18 | 0.024 | 0.026 | 0.010 | 0.859 | 0.030 | 0.020 | 0.007 | 1.000 | 0.025 | 0.037 | 0.025 | 0.020 |
| IL18BP | 0.031 | 0.001 | 0.040 | 0.254 | 0.028 | 0.028 | 0.007 | 1.000 | 0.033 | 0.002 | 0.032 | 0.001 |
| IL1RL2 | -0.024 | 0.021 | -0.012 | 0.818 | -0.027 | 0.039 | -0.008 | 1.000 | -0.026 | 0.021 | -0.024 | 0.022 |
| IL1RN | 0.025 | 0.007 | 0.032 | 0.345 | 0.021 | 0.118 | 0.012 | 1.000 | 0.025 | 0.013 | 0.024 | 0.009 |
| IL7R | -0.023 | 0.041 | -0.020 | 0.661 | -0.020 | 0.224 | -0.022 | 1.000 | -0.021 | 0.092 | -0.021 | 0.071 |
| INSL3 | -0.017 | 0.003 | -0.016 | 0.619 | -0.016 | 0.024 | -0.019 | 1.000 | -0.017 | 0.010 | -0.017 | 0.004 |
| INSR | 0.029 | 0.012 | 0.018 | 0.737 | 0.034 | 0.017 | 0.057 | 1.000 | 0.025 | 0.066 | 0.030 | 0.010 |
| ITGA5 | 0.026 | 0.009 | 0.020 | 0.649 | 0.029 | 0.021 | 0.025 | 1.000 | 0.025 | 0.022 | 0.026 | 0.008 |
| ITIH3 | 0.025 | 0.015 | 0.017 | 0.725 | 0.024 | 0.079 | 0.016 | 1.000 | 0.025 | 0.023 | 0.024 | 0.020 |
| ITIH4 | 0.040 | 7.70E-05 | 0.044 | 0.235 | 0.038 | 0.003 | 0.068 | 0.896 | 0.036 | 0.001 | 0.039 | 1.11E-04 |
| KEL | -0.022 | 0.046 | -0.042 | 0.251 | -0.015 | 0.417 | 0.011 | 1.000 | -0.027 | 0.019 | -0.021 | 0.057 |
| KITLG | -0.024 | 0.026 | 0.001 | 0.991 | -0.033 | 0.009 | -0.032 | 1.000 | -0.021 | 0.090 | -0.023 | 0.040 |
| KLK4 | 0.028 | 0.004 | 0.057 | 0.039 | 0.014 | 0.417 | 0.045 | 1.000 | 0.025 | 0.022 | 0.028 | 0.005 |
| KYNU | 0.023 | 0.030 | 0.033 | 0.425 | 0.023 | 0.119 | 0.008 | 1.000 | 0.024 | 0.036 | 0.023 | 0.026 |
| L1CAM | 0.023 | 0.033 | 0.015 | 0.759 | 0.029 | 0.022 | 0.009 | 1.000 | 0.025 | 0.035 | 0.024 | 0.021 |
| LAMP3 | 0.035 | 6.14E-05 | 0.036 | 0.296 | 0.037 | 0.001 | 0.013 | 1.000 | 0.037 | 1.24E-04 | 0.036 | 5.34E-05 |
| LEPR | 0.030 | 0.002 | 0.026 | 0.539 | 0.031 | 0.010 | 0.028 | 1.000 | 0.031 | 0.004 | 0.030 | 0.002 |
| LGALS3 | 0.027 | 0.007 | 0.023 | 0.617 | 0.028 | 0.028 | 0.044 | 1.000 | 0.024 | 0.040 | 0.027 | 0.006 |
| LGALS3BP | 0.036 | 4.28E-04 | 0.037 | 0.367 | 0.035 | 0.008 | 0.040 | 1.000 | 0.035 | 0.002 | 0.036 | 0.001 |
| LGALS4 | 0.026 | 0.010 | 0.020 | 0.649 | 0.019 | 0.241 | 0.003 | 1.000 | 0.027 | 0.014 | 0.024 | 0.019 |
| LGALS9 | 0.033 | 1.54E-04 | 0.055 | 0.039 | 0.027 | 0.023 | 0.018 | 1.000 | 0.033 | 0.001 | 0.033 | 9.55E-05 |
| LILRB4 | 0.031 | 0.001 | 0.041 | 0.254 | 0.027 | 0.034 | 0.025 | 1.000 | 0.030 | 0.004 | 0.031 | 0.001 |
| LRRC25 | 0.027 | 0.008 | 0.023 | 0.619 | 0.030 | 0.019 | 0.041 | 1.000 | 0.024 | 0.043 | 0.028 | 0.005 |
| LTA4H | 0.024 | 0.024 | 0.013 | 0.796 | 0.029 | 0.022 | 0.035 | 1.000 | 0.022 | 0.080 | 0.024 | 0.022 |
| LTBP3 | 0.024 | 0.023 | 0.041 | 0.254 | 0.020 | 0.218 | 0.009 | 1.000 | 0.025 | 0.033 | 0.025 | 0.017 |
| MANSC4 | 0.054 | 1.22E-07 | 0.043 | 0.296 | 0.058 | 1.04E-06 | -0.003 | 1.000 | 0.061 | 5.99E-09 | 0.054 | 8.25E-08 |
| MEGF9 | 0.024 | 0.024 | 0.043 | 0.212 | 0.023 | 0.143 | 0.030 | 1.000 | 0.023 | 0.057 | 0.026 | 0.015 |
| MEP1B | -0.025 | 0.020 | -0.007 | 0.919 | -0.027 | 0.053 | 0.003 | 1.000 | -0.028 | 0.016 | -0.024 | 0.025 |
| MFAP4 | -0.026 | 0.031 | -0.019 | 0.732 | -0.027 | 0.106 | -0.018 | 1.000 | -0.027 | 0.050 | -0.026 | 0.035 |
| MILR1 | 0.031 | 0.003 | 0.046 | 0.180 | 0.025 | 0.093 | 0.042 | 1.000 | 0.029 | 0.015 | 0.031 | 0.003 |
| MMP12 | 0.040 | 2.65E-06 | 0.055 | 0.039 | 0.032 | 0.007 | 0.019 | 1.000 | 0.042 | 5.58E-06 | 0.039 | 4.65E-06 |
| MMP7 | 0.025 | 0.020 | 0.041 | 0.259 | 0.016 | 0.352 | 0.026 | 1.000 | 0.023 | 0.064 | 0.025 | 0.018 |
| MRC1 | 0.034 | 0.001 | 0.030 | 0.512 | 0.035 | 0.006 | 0.033 | 1.000 | 0.033 | 0.003 | 0.033 | 0.001 |
| MSLN | 0.022 | 0.034 | 0.009 | 0.886 | 0.027 | 0.037 | 0.024 | 1.000 | 0.020 | 0.100 | 0.022 | 0.033 |
| MSR1 | 0.042 | 5.74E-07 | 0.063 | 0.024 | 0.038 | 0.001 | 0.010 | 1.000 | 0.046 | 3.15E-07 | 0.043 | 4.94E-07 |
| MSTN | -0.025 | 0.010 | -0.013 | 0.792 | -0.028 | 0.022 | -0.025 | 1.000 | -0.023 | 0.036 | -0.024 | 0.017 |
| MZB1 | 0.043 | 1.73E-06 | 0.040 | 0.254 | 0.043 | 1.39E-04 | 0.033 | 1.000 | 0.044 | 4.81E-06 | 0.043 | 1.47E-06 |
| NAPRT | -0.027 | 0.022 | -0.061 | 0.049 | -0.016 | 0.438 | 0.021 | 1.000 | -0.032 | 0.011 | -0.027 | 0.024 |
| NCS1 | 0.022 | 0.031 | 0.039 | 0.269 | 0.015 | 0.367 | 0.011 | 1.000 | 0.021 | 0.065 | 0.022 | 0.025 |
| NHLRC3 | 0.035 | 0.001 | 0.063 | 0.039 | 0.024 | 0.147 | 0.062 | 0.920 | 0.031 | 0.011 | 0.035 | 0.001 |
| NOMO1 | 0.028 | 0.005 | 0.057 | 0.043 | 0.022 | 0.147 | 0.068 | 0.896 | 0.022 | 0.060 | 0.029 | 0.003 |
| NOTCH3 | -0.021 | 0.044 | -0.029 | 0.451 | -0.019 | 0.216 | -0.035 | 1.000 | -0.019 | 0.122 | -0.021 | 0.051 |
| NPL | 0.027 | 0.008 | 0.027 | 0.555 | 0.029 | 0.024 | 0.032 | 1.000 | 0.026 | 0.020 | 0.027 | 0.006 |
| NTRK3 | -0.036 | 6.63E-05 | -0.044 | 0.164 | -0.030 | 0.014 | -0.037 | 1.000 | -0.035 | 0.001 | -0.034 | 1.74E-04 |
| OCLN | 0.028 | 0.008 | 0.039 | 0.318 | 0.023 | 0.162 | 0.061 | 0.920 | 0.022 | 0.089 | 0.028 | 0.008 |
| OMD | -0.036 | 1.38E-04 | -0.014 | 0.784 | -0.040 | 4.58E-04 | -0.055 | 0.975 | -0.033 | 0.002 | -0.035 | 2.26E-04 |
| OSCAR | 0.027 | 0.009 | 0.028 | 0.505 | 0.026 | 0.066 | 0.029 | 1.000 | 0.026 | 0.031 | 0.028 | 0.006 |
| OSMR | 0.036 | 1.38E-04 | 0.045 | 0.164 | 0.034 | 0.005 | 0.014 | 1.000 | 0.038 | 2.20E-04 | 0.036 | 1.11E-04 |
| PI16 | -0.025 | 0.031 | -0.027 | 0.555 | -0.023 | 0.192 | -0.045 | 1.000 | -0.023 | 0.092 | -0.023 | 0.053 |
| PIGR | 0.046 | 1.87E-07 | 0.033 | 0.401 | 0.047 | 1.35E-05 | 0.031 | 1.000 | 0.046 | 1.46E-06 | 0.046 | 3.02E-07 |
| PILRA | 0.030 | 0.002 | 0.048 | 0.134 | 0.025 | 0.087 | 0.028 | 1.000 | 0.030 | 0.007 | 0.031 | 0.001 |
| PILRB | 0.029 | 0.005 | 0.051 | 0.098 | 0.021 | 0.205 | 0.035 | 1.000 | 0.027 | 0.018 | 0.029 | 0.004 |
| PKLR | -0.026 | 0.018 | -0.047 | 0.176 | -0.019 | 0.256 | 0.001 | 1.000 | -0.029 | 0.013 | -0.025 | 0.020 |
| PLA2G15 | 0.027 | 0.008 | 0.018 | 0.696 | 0.031 | 0.013 | 0.007 | 1.000 | 0.029 | 0.010 | 0.027 | 0.007 |
| PLA2G2A | 0.024 | 0.020 | 0.037 | 0.296 | 0.021 | 0.184 | 0.039 | 1.000 | 0.022 | 0.067 | 0.024 | 0.020 |
| PLAUR | 0.035 | 1.08E-04 | 0.022 | 0.619 | 0.037 | 0.001 | 0.017 | 1.000 | 0.035 | 4.67E-04 | 0.035 | 8.95E-05 |
| PLTP | -0.027 | 0.007 | -0.037 | 0.296 | -0.022 | 0.125 | -0.044 | 1.000 | -0.024 | 0.038 | -0.026 | 0.011 |
| PNLIPRP2 | -0.025 | 0.024 | -0.048 | 0.154 | -0.018 | 0.301 | -0.039 | 1.000 | -0.024 | 0.060 | -0.025 | 0.022 |
| PON1 | -0.024 | 0.046 | -0.022 | 0.649 | -0.021 | 0.241 | 0.020 | 1.000 | -0.029 | 0.022 | -0.023 | 0.069 |
| PON2 | 0.025 | 0.023 | 0.024 | 0.619 | 0.028 | 0.048 | 0.051 | 1.000 | 0.021 | 0.109 | 0.026 | 0.017 |
| PRCP | 0.024 | 0.018 | 0.031 | 0.446 | 0.024 | 0.088 | -0.013 | 1.000 | 0.028 | 0.010 | 0.025 | 0.015 |
| PRDX2 | -0.032 | 0.005 | -0.067 | 0.039 | -0.021 | 0.258 | -0.009 | 1.000 | -0.034 | 0.005 | -0.031 | 0.006 |
| PRKAR1A | 0.023 | 0.048 | 0.033 | 0.435 | 0.021 | 0.218 | 0.013 | 1.000 | 0.024 | 0.062 | 0.023 | 0.041 |
| PRSS8 | 0.037 | 1.28E-05 | 0.053 | 0.049 | 0.028 | 0.017 | 0.038 | 1.000 | 0.035 | 1.48E-04 | 0.036 | 1.48E-05 |
| PTPRF | -0.032 | 0.001 | -0.015 | 0.756 | -0.032 | 0.009 | -0.039 | 1.000 | -0.030 | 0.006 | -0.030 | 0.002 |
| PVR | 0.023 | 0.036 | 0.020 | 0.652 | 0.022 | 0.167 | 0.031 | 1.000 | 0.022 | 0.089 | 0.023 | 0.037 |
| RARRES2 | 0.034 | 1.08E-04 | 0.047 | 0.118 | 0.031 | 0.008 | 0.003 | 1.000 | 0.037 | 1.27E-04 | 0.035 | 7.76E-05 |
| REG3A | 0.028 | 0.005 | 0.017 | 0.725 | 0.029 | 0.021 | 0.010 | 1.000 | 0.029 | 0.008 | 0.028 | 0.004 |
| REG4 | 0.027 | 0.008 | 0.005 | 0.941 | 0.030 | 0.018 | 0.011 | 1.000 | 0.027 | 0.013 | 0.026 | 0.008 |
| REN | 0.037 | 4.93E-05 | 0.018 | 0.710 | 0.037 | 0.002 | 0.020 | 1.000 | 0.038 | 1.19E-04 | 0.035 | 1.01E-04 |
| RGMA | -0.027 | 0.009 | -0.019 | 0.696 | -0.029 | 0.023 | -0.059 | 0.896 | -0.022 | 0.078 | -0.026 | 0.014 |
| RGS8 | 0.029 | 0.006 | 0.025 | 0.617 | 0.032 | 0.017 | 0.053 | 1.000 | 0.026 | 0.033 | 0.030 | 0.004 |
| ROBO1 | 0.028 | 0.005 | 0.048 | 0.112 | 0.022 | 0.153 | 0.034 | 1.000 | 0.027 | 0.018 | 0.028 | 0.005 |
| SCGB1A1 | -0.035 | 2.02E-04 | -0.030 | 0.444 | -0.036 | 0.003 | -0.034 | 1.000 | -0.034 | 0.001 | -0.035 | 2.37E-04 |
| SCGN | 0.024 | 0.030 | 0.032 | 0.435 | 0.017 | 0.323 | 0.024 | 1.000 | 0.023 | 0.064 | 0.024 | 0.031 |
| SCT | -0.041 | 1.38E-04 | -0.045 | 0.254 | -0.038 | 0.005 | -0.036 | 1.000 | -0.040 | 0.001 | -0.039 | 2.74E-04 |
| SERPIND1 | 0.029 | 0.008 | 0.041 | 0.294 | 0.023 | 0.167 | 0.016 | 1.000 | 0.029 | 0.015 | 0.028 | 0.009 |
| SIGLEC1 | 0.023 | 0.024 | 0.031 | 0.444 | 0.022 | 0.140 | 0.033 | 1.000 | 0.021 | 0.089 | 0.024 | 0.020 |
| SIGLEC7 | 0.026 | 0.009 | 0.040 | 0.254 | 0.024 | 0.097 | 0.046 | 1.000 | 0.023 | 0.057 | 0.027 | 0.008 |
| SIGLEC9 | 0.039 | 4.73E-05 | 0.068 | 0.012 | 0.033 | 0.008 | 0.040 | 1.000 | 0.039 | 2.06E-04 | 0.040 | 3.49E-05 |
| SIRPB1 | 0.024 | 0.026 | 0.038 | 0.301 | 0.018 | 0.281 | -0.003 | 1.000 | 0.026 | 0.026 | 0.025 | 0.017 |
| SIT1 | 0.023 | 0.031 | 0.058 | 0.039 | 0.011 | 0.586 | 0.009 | 1.000 | 0.024 | 0.052 | 0.022 | 0.047 |
| SLAMF7 | 0.023 | 0.033 | 0.039 | 0.296 | 0.018 | 0.281 | 0.014 | 1.000 | 0.025 | 0.044 | 0.024 | 0.029 |
| SLITRK2 | 0.023 | 0.030 | 0.030 | 0.444 | 0.020 | 0.192 | 0.003 | 1.000 | 0.026 | 0.022 | 0.023 | 0.024 |
| SMPD1 | 0.023 | 0.040 | 0.006 | 0.921 | 0.028 | 0.039 | 0.007 | 1.000 | 0.024 | 0.054 | 0.023 | 0.039 |
| SOD2 | 0.024 | 0.024 | 0.030 | 0.461 | 0.022 | 0.162 | 0.010 | 1.000 | 0.025 | 0.035 | 0.024 | 0.022 |
| SPINK6 | 0.032 | 0.001 | 0.031 | 0.444 | 0.034 | 0.005 | 0.033 | 1.000 | 0.032 | 0.002 | 0.033 | 3.98E-04 |
| SPINT3 | -0.018 | 0.001 | -0.011 | 0.721 | -0.020 | 0.004 | -0.003 | 1.000 | -0.020 | 0.001 | -0.018 | 0.001 |
| SPTLC1 | 0.025 | 0.046 | 0.021 | 0.694 | 0.026 | 0.119 | 0.032 | 1.000 | 0.024 | 0.089 | 0.025 | 0.043 |
| SSC4D | -0.026 | 0.003 | -0.016 | 0.739 | -0.027 | 0.017 | -0.039 | 1.000 | -0.024 | 0.016 | -0.025 | 0.005 |
| SUSD5 | -0.027 | 0.012 | < 0.001 | 0.997 | -0.033 | 0.009 | -0.019 | 1.000 | -0.027 | 0.018 | -0.024 | 0.022 |
| TCN2 | 0.027 | 0.009 | 0.017 | 0.719 | 0.027 | 0.042 | 0.045 | 1.000 | 0.023 | 0.060 | 0.027 | 0.009 |
| TFF1 | 0.057 | 1.45E-11 | 0.052 | 0.075 | 0.056 | 5.40E-08 | 0.038 | 1.000 | 0.058 | 1.98E-10 | 0.057 | 6.80E-12 |
| TFF2 | 0.038 | 2.84E-05 | 0.029 | 0.460 | 0.038 | 0.001 | 0.017 | 1.000 | 0.039 | 7.46E-05 | 0.039 | 1.76E-05 |
| TFF3 | 0.022 | 0.047 | 0.017 | 0.718 | 0.020 | 0.218 | 0.023 | 1.000 | 0.020 | 0.117 | 0.022 | 0.043 |
| TG | -0.036 | 0.001 | -0.028 | 0.568 | -0.039 | 0.004 | -0.087 | 0.896 | -0.030 | 0.016 | -0.036 | 0.001 |
| TGFBR3 | -0.022 | 0.046 | -0.034 | 0.418 | -0.019 | 0.253 | -0.035 | 1.000 | -0.022 | 0.084 | -0.021 | 0.065 |
| TNC | 0.024 | 0.024 | 0.029 | 0.477 | 0.024 | 0.118 | 0.023 | 1.000 | 0.024 | 0.056 | 0.025 | 0.022 |
| TNF | 0.025 | 0.018 | 0.033 | 0.431 | 0.022 | 0.162 | 0.006 | 1.000 | 0.028 | 0.017 | 0.026 | 0.017 |
| TNFRSF10A | 0.024 | 0.015 | 0.021 | 0.638 | 0.020 | 0.189 | 0.044 | 1.000 | 0.020 | 0.095 | 0.024 | 0.016 |
| TNFRSF10B | 0.036 | 4.73E-05 | 0.033 | 0.411 | 0.035 | 0.003 | 0.051 | 0.975 | 0.032 | 0.001 | 0.036 | 3.93E-05 |
| TNFRSF12A | 0.022 | 0.030 | 0.034 | 0.378 | 0.016 | 0.311 | 0.015 | 1.000 | 0.022 | 0.059 | 0.023 | 0.019 |
| TNFRSF1B | 0.022 | 0.039 | 0.029 | 0.485 | 0.018 | 0.282 | 0.018 | 1.000 | 0.021 | 0.086 | 0.023 | 0.026 |
| TNFSF11 | -0.028 | 0.006 | 0.001 | 0.990 | -0.032 | 0.012 | -0.006 | 1.000 | -0.030 | 0.008 | -0.027 | 0.012 |
| TNFSF13B | 0.026 | 0.009 | 0.010 | 0.858 | 0.029 | 0.019 | 0.017 | 1.000 | 0.026 | 0.023 | 0.025 | 0.012 |
| TNFSF8 | 0.029 | 0.010 | 0.028 | 0.555 | 0.032 | 0.028 | 0.057 | 1.000 | 0.025 | 0.062 | 0.030 | 0.008 |
| TNR | -0.037 | 6.37E-05 | -0.046 | 0.153 | -0.035 | 0.004 | -0.041 | 1.000 | -0.036 | 0.001 | -0.037 | 7.76E-05 |
| TP53BP1 | -0.028 | 0.016 | -0.059 | 0.070 | -0.020 | 0.307 | -0.021 | 1.000 | -0.030 | 0.021 | -0.028 | 0.016 |
| TPP1 | 0.023 | 0.033 | 0.003 | 0.961 | 0.030 | 0.020 | 0.008 | 1.000 | 0.024 | 0.042 | 0.023 | 0.031 |
| TRAF3 | -0.029 | 0.014 | -0.049 | 0.201 | -0.023 | 0.218 | -0.015 | 1.000 | -0.032 | 0.013 | -0.029 | 0.016 |
| TREM2 | 0.052 | 2.68E-10 | 0.048 | 0.109 | 0.053 | 5.40E-08 | 0.035 | 1.000 | 0.053 | 1.28E-09 | 0.052 | 1.08E-10 |
| TXNDC15 | 0.034 | 0.001 | 0.033 | 0.435 | 0.036 | 0.004 | 0.017 | 1.000 | 0.035 | 0.001 | 0.034 | 0.001 |
| TYRP1 | 0.032 | 0.004 | 0.043 | 0.282 | 0.028 | 0.079 | 0.046 | 1.000 | 0.030 | 0.019 | 0.032 | 0.004 |
| UBAC1 | -0.026 | 0.015 | -0.047 | 0.155 | -0.020 | 0.246 | -0.040 | 1.000 | -0.024 | 0.048 | -0.026 | 0.017 |
| UHRF2 | 0.026 | 0.039 | 0.050 | 0.198 | 0.018 | 0.368 | 0.039 | 1.000 | 0.024 | 0.094 | 0.025 | 0.043 |
| ULBP2 | 0.025 | 0.019 | 0.027 | 0.528 | 0.022 | 0.153 | 0.029 | 1.000 | 0.023 | 0.058 | 0.025 | 0.013 |
| VIT | -0.029 | 0.005 | -0.013 | 0.807 | -0.031 | 0.011 | -0.075 | 0.896 | -0.023 | 0.064 | -0.028 | 0.006 |
| VSIG2 | 0.034 | 0.001 | 0.013 | 0.829 | 0.037 | 0.005 | 0.051 | 1.000 | 0.030 | 0.015 | 0.033 | 0.001 |
| VWC2 | 0.034 | 2.31E-04 | 0.034 | 0.399 | 0.032 | 0.008 | 0.035 | 1.000 | 0.033 | 0.001 | 0.034 | 1.91E-04 |
| WFDC12 | 0.025 | 0.008 | 0.022 | 0.619 | 0.026 | 0.033 | 0.005 | 1.000 | 0.027 | 0.007 | 0.025 | 0.006 |
| WFDC2 | 0.030 | 0.001 | 0.039 | 0.251 | 0.023 | 0.095 | 0.003 | 1.000 | 0.031 | 0.002 | 0.030 | 0.001 |
| XPNPEP2 | -0.025 | 0.022 | -0.019 | 0.672 | -0.025 | 0.105 | 0.023 | 1.000 | -0.030 | 0.007 | -0.024 | 0.028 |

| **Supplementary Table 33. Detailed protein associations with anxiety disorder in the primary and sensitivity analyses.** | | | | | | | | | | | | |
| --- | --- | --- | --- | --- | --- | --- | --- | --- | --- | --- | --- | --- |
| **Protein** | **Primary analysis** | | **Lipid-lowering medication users** | | **No lipid-lowering medications** | | **Psychotropic medication users** | | **No psychotropic medications** | | **Additional adjustment** | |
|  | **HR (95% CI)** | **FDR** | **HR (95% CI)** | **FDR** | **HR (95% CI)** | **FDR** | **HR (95% CI)** | **FDR** | **HR (95% CI)** | **FDR** | **HR (95% CI)** | **FDR** |
| ACE2 | 1.136 (1.057-1.220) | 0.012 | 1.101 (0.963-1.259) | 0.447 | 1.129 (1.037-1.231) | 0.156 | 0.988 (0.880-1.110) | 0.998 | 1.075 (0.983-1.175) | 0.411 | 1.114 (1.037-1.198) | 0.067 |
| ACVRL1 | 1.121 (1.045-1.204) | 0.027 | 1.198 (1.053-1.363) | 0.106 | 1.092 (1.004-1.187) | 0.356 | 1.046 (0.924-1.185) | 0.998 | 1.087 (0.998-1.184) | 0.328 | 1.096 (1.020-1.177) | 0.122 |
| ADAM9 | 1.132 (1.054-1.216) | 0.014 | 1.195 (1.035-1.379) | 0.159 | 1.110 (1.023-1.205) | 0.235 | 1.291 (1.129-1.475) | 0.073 | 1.043 (0.958-1.136) | 0.626 | 1.113 (1.036-1.195) | 0.067 |
| ADAMTSL2 | 1.143 (1.063-1.230) | 0.009 | 1.169 (1.013-1.348) | 0.219 | 1.122 (1.030-1.224) | 0.199 | 1.090 (0.960-1.238) | 0.924 | 1.118 (1.024-1.221) | 0.203 | 1.127 (1.047-1.213) | 0.036 |
| ADGRG1 | 1.125 (1.053-1.201) | 0.011 | 1.212 (1.089-1.348) | 0.029 | 1.074 (0.986-1.170) | 0.506 | 1.025 (0.907-1.158) | 0.998 | 1.130 (1.046-1.222) | 0.095 | 1.104 (1.033-1.181) | 0.069 |
| ADM | 1.165 (1.075-1.263) | 0.007 | 1.187 (0.999-1.411) | 0.268 | 1.148 (1.048-1.257) | 0.110 | 1.114 (0.960-1.293) | 0.908 | 1.097 (0.999-1.205) | 0.321 | 1.133 (1.045-1.228) | 0.055 |
| AGRN | 1.120 (1.047-1.197) | 0.019 | 1.097 (0.968-1.244) | 0.430 | 1.117 (1.032-1.209) | 0.167 | 1.006 (0.890-1.136) | 0.998 | 1.091 (1.007-1.182) | 0.288 | 1.094 (1.023-1.171) | 0.107 |
| ANGPT2 | 1.147 (1.073-1.225) | 0.003 | 1.163 (1.002-1.349) | 0.261 | 1.136 (1.054-1.224) | 0.059 | 1.024 (0.901-1.164) | 0.998 | 1.163 (1.076-1.257) | 0.029 | 1.125 (1.052-1.202) | 0.024 |
| APOM | 0.903 (0.843-0.966) | 0.043 | 0.792 (0.685-0.916) | 0.065 | 0.949 (0.877-1.026) | 0.624 | 0.971 (0.850-1.108) | 0.998 | 0.889 (0.822-0.963) | 0.128 | 0.921 (0.860-0.986) | 0.146 |
| AREG | 1.145 (1.079-1.216) | 0.001 | 1.161 (1.015-1.328) | 0.211 | 1.140 (1.066-1.220) | 0.036 | 1.025 (0.913-1.150) | 0.998 | 1.121 (1.043-1.205) | 0.092 | 1.122 (1.056-1.193) | 0.014 |
| ASAH1 | 1.117 (1.047-1.192) | 0.017 | 1.264 (1.110-1.439) | 0.029 | 1.080 (1.002-1.164) | 0.374 | 1.014 (0.883-1.163) | 0.998 | 1.131 (1.050-1.217) | 0.076 | 1.116 (1.046-1.191) | 0.030 |
| ASGR1 | 1.185 (1.104-1.272) | 3.57E-04 | 1.208 (1.050-1.390) | 0.125 | 1.162 (1.069-1.262) | 0.040 | 1.115 (0.980-1.269) | 0.821 | 1.096 (1.006-1.194) | 0.296 | 1.160 (1.080-1.246) | 0.005 |
| ATRAID | 1.130 (1.061-1.204) | 0.005 | 1.196 (1.105-1.295) | 0.003 | 1.067 (0.981-1.160) | 0.570 | 1.140 (1.051-1.235) | 0.241 | 1.055 (0.969-1.148) | 0.527 | 1.104 (1.037-1.176) | 0.048 |
| B2M | 1.131 (1.080-1.184) | 5.31E-05 | 1.269 (1.167-1.380) | 8.13E-05 | 1.088 (1.022-1.157) | 0.186 | 1.111 (0.996-1.240) | 0.750 | 1.125 (1.065-1.188) | 0.012 | 1.116 (1.063-1.172) | 0.003 |
| B4GALT1 | 1.150 (1.077-1.228) | 0.002 | 1.228 (1.076-1.402) | 0.073 | 1.120 (1.039-1.207) | 0.110 | 1.090 (0.963-1.234) | 0.924 | 1.098 (1.016-1.186) | 0.234 | 1.130 (1.058-1.206) | 0.016 |
| BMPER | 1.125 (1.055-1.199) | 0.009 | 1.310 (1.143-1.501) | 0.014 | 1.071 (0.991-1.158) | 0.466 | 1.111 (0.979-1.260) | 0.821 | 1.115 (1.033-1.204) | 0.152 | 1.112 (1.043-1.187) | 0.034 |
| BST2 | 1.128 (1.061-1.200) | 0.005 | 1.144 (0.994-1.318) | 0.288 | 1.117 (1.041-1.197) | 0.089 | 1.069 (0.960-1.192) | 0.974 | 1.081 (1.001-1.167) | 0.304 | 1.120 (1.052-1.192) | 0.017 |
| BTN3A2 | 1.158 (1.083-1.239) | 0.001 | 1.212 (1.047-1.404) | 0.136 | 1.141 (1.057-1.231) | 0.054 | 1.023 (0.901-1.161) | 0.998 | 1.149 (1.061-1.244) | 0.059 | 1.142 (1.067-1.222) | 0.010 |
| C1QBP | 1.087 (1.028-1.150) | 0.045 | 1.168 (1.036-1.317) | 0.144 | 1.073 (1.006-1.144) | 0.316 | 1.166 (1.044-1.303) | 0.408 | 1.068 (0.997-1.144) | 0.340 | 1.088 (1.029-1.151) | 0.066 |
| C7 | 1.117 (1.037-1.203) | 0.045 | 1.123 (0.955-1.319) | 0.447 | 1.108 (1.019-1.206) | 0.262 | 1.123 (0.977-1.291) | 0.821 | 1.097 (1.005-1.197) | 0.299 | 1.101 (1.022-1.186) | 0.120 |
| CA14 | 0.857 (0.797-0.922) | 0.002 | 0.871 (0.746-1.017) | 0.329 | 0.871 (0.801-0.947) | 0.072 | 1.234 (1.081-1.408) | 0.271 | 0.796 (0.731-0.868) | 2.98E-04 | 0.855 (0.795-0.920) | 0.003 |
| CA6 | 0.883 (0.823-0.947) | 0.012 | 0.901 (0.768-1.058) | 0.492 | 0.880 (0.814-0.952) | 0.080 | 0.922 (0.804-1.056) | 0.974 | 0.907 (0.836-0.985) | 0.244 | 0.889 (0.828-0.954) | 0.032 |
| CCER2 | 1.121 (1.046-1.202) | 0.022 | 1.221 (1.059-1.408) | 0.106 | 1.097 (1.014-1.187) | 0.285 | 1.216 (1.083-1.365) | 0.204 | 1.014 (0.932-1.104) | 0.891 | 1.110 (1.035-1.189) | 0.065 |
| CCL15 | 1.100 (1.032-1.173) | 0.044 | 1.121 (0.980-1.282) | 0.351 | 1.085 (1.009-1.168) | 0.310 | 1.097 (0.971-1.240) | 0.875 | 1.076 (0.998-1.161) | 0.330 | 1.077 (1.010-1.149) | 0.174 |
| CCL19 | 1.102 (1.039-1.169) | 0.022 | 1.015 (0.881-1.169) | 0.922 | 1.124 (1.053-1.199) | 0.040 | 1.054 (0.943-1.178) | 0.998 | 1.098 (1.025-1.177) | 0.169 | 1.096 (1.033-1.162) | 0.051 |
| CD274 | 1.146 (1.078-1.220) | 0.001 | 1.226 (1.084-1.386) | 0.055 | 1.131 (1.053-1.214) | 0.054 | 1.042 (0.924-1.175) | 0.998 | 1.144 (1.063-1.230) | 0.047 | 1.139 (1.070-1.212) | 0.005 |
| CD276 | 1.142 (1.067-1.223) | 0.005 | 1.143 (0.999-1.308) | 0.270 | 1.135 (1.049-1.229) | 0.087 | 0.941 (0.828-1.070) | 0.998 | 1.173 (1.082-1.271) | 0.028 | 1.129 (1.054-1.208) | 0.022 |
| CD302 | 1.122 (1.048-1.202) | 0.020 | 1.171 (1.033-1.328) | 0.153 | 1.099 (1.014-1.192) | 0.287 | 0.970 (0.856-1.098) | 0.998 | 1.082 (0.995-1.176) | 0.345 | 1.093 (1.019-1.171) | 0.127 |
| CD4 | 1.088 (1.031-1.149) | 0.034 | 1.252 (1.100-1.424) | 0.039 | 1.055 (0.988-1.127) | 0.528 | 1.039 (0.929-1.161) | 0.998 | 1.078 (1.009-1.152) | 0.265 | 1.080 (1.021-1.143) | 0.099 |
| CD40 | 1.109 (1.040-1.182) | 0.028 | 1.182 (1.025-1.363) | 0.189 | 1.086 (1.010-1.167) | 0.300 | 0.938 (0.828-1.061) | 0.998 | 1.140 (1.058-1.229) | 0.059 | 1.103 (1.034-1.176) | 0.061 |
| CD79B | 1.108 (1.036-1.184) | 0.038 | 1.254 (1.109-1.419) | 0.029 | 1.058 (0.979-1.144) | 0.588 | 1.126 (1.001-1.266) | 0.739 | 1.053 (0.972-1.141) | 0.518 | 1.088 (1.018-1.164) | 0.130 |
| CD80 | 1.144 (1.065-1.229) | 0.007 | 1.262 (1.085-1.469) | 0.075 | 1.109 (1.023-1.203) | 0.235 | 1.160 (1.013-1.329) | 0.671 | 1.094 (1.005-1.191) | 0.300 | 1.135 (1.056-1.219) | 0.023 |
| CD83 | 1.163 (1.087-1.244) | 0.001 | 1.271 (1.121-1.441) | 0.022 | 1.116 (1.032-1.208) | 0.172 | 1.071 (0.956-1.200) | 0.974 | 1.116 (1.029-1.211) | 0.169 | 1.145 (1.070-1.225) | 0.008 |
| CD99L2 | 1.144 (1.069-1.224) | 0.004 | 1.236 (1.070-1.427) | 0.084 | 1.130 (1.046-1.220) | 0.089 | 1.059 (0.940-1.193) | 0.998 | 1.122 (1.036-1.216) | 0.151 | 1.137 (1.063-1.217) | 0.014 |
| CDCP1 | 1.117 (1.038-1.203) | 0.044 | 1.193 (1.022-1.392) | 0.201 | 1.090 (1.002-1.186) | 0.375 | 0.992 (0.871-1.130) | 0.998 | 1.079 (0.988-1.179) | 0.377 | 1.106 (1.027-1.191) | 0.100 |
| CEACAM5 | 1.132 (1.058-1.211) | 0.009 | 1.213 (1.047-1.405) | 0.136 | 1.110 (1.028-1.198) | 0.184 | 1.113 (0.979-1.266) | 0.821 | 1.103 (1.018-1.195) | 0.224 | 1.124 (1.050-1.203) | 0.027 |
| CEMIP2 | 1.100 (1.043-1.161) | 0.011 | 1.178 (1.037-1.338) | 0.145 | 1.083 (1.019-1.152) | 0.221 | 1.019 (0.884-1.175) | 0.998 | 1.117 (1.052-1.185) | 0.047 | 1.103 (1.045-1.164) | 0.017 |
| CES3 | 0.909 (0.854-0.968) | 0.040 | 0.848 (0.732-0.982) | 0.206 | 0.924 (0.863-0.990) | 0.298 | 0.857 (0.755-0.973) | 0.577 | 0.955 (0.891-1.024) | 0.505 | 0.911 (0.856-0.970) | 0.068 |
| CHAD | 0.900 (0.838-0.966) | 0.044 | 0.942 (0.806-1.101) | 0.691 | 0.886 (0.818-0.960) | 0.110 | 0.980 (0.860-1.116) | 0.998 | 0.896 (0.824-0.973) | 0.176 | 0.904 (0.842-0.970) | 0.079 |
| CHCHD10 | 1.179 (1.098-1.267) | 0.001 | 1.301 (1.175-1.441) | 0.001 | 1.100 (1.007-1.202) | 0.333 | 1.145 (1.026-1.278) | 0.549 | 1.097 (1.002-1.201) | 0.304 | 1.150 (1.070-1.235) | 0.010 |
| CHCHD6 | 1.101 (1.042-1.164) | 0.015 | 1.133 (1.010-1.271) | 0.219 | 1.085 (1.018-1.157) | 0.235 | 1.103 (1.025-1.187) | 0.429 | 1.054 (0.978-1.137) | 0.479 | 1.095 (1.034-1.159) | 0.044 |
| CHGA | 1.172 (1.101-1.247) | 1.59E-04 | 1.213 (1.085-1.357) | 0.042 | 1.149 (1.065-1.240) | 0.039 | 1.052 (0.947-1.167) | 0.998 | 1.123 (1.039-1.215) | 0.128 | 1.147 (1.077-1.223) | 0.003 |
| CHRDL1 | 1.134 (1.052-1.223) | 0.021 | 1.149 (0.983-1.343) | 0.329 | 1.129 (1.036-1.230) | 0.160 | 1.010 (0.885-1.153) | 0.998 | 1.157 (1.059-1.264) | 0.079 | 1.116 (1.035-1.203) | 0.075 |
| CKAP4 | 1.109 (1.038-1.185) | 0.032 | 1.191 (1.058-1.341) | 0.084 | 1.080 (0.999-1.168) | 0.394 | 1.038 (0.920-1.172) | 0.998 | 1.081 (0.999-1.170) | 0.321 | 1.087 (1.017-1.162) | 0.133 |
| CLMP | 1.123 (1.038-1.215) | 0.047 | 1.161 (1.006-1.339) | 0.248 | 1.097 (1.000-1.203) | 0.384 | 1.132 (0.981-1.305) | 0.818 | 1.067 (0.972-1.171) | 0.479 | 1.095 (1.012-1.185) | 0.174 |
| CLPP | 1.113 (1.041-1.189) | 0.027 | 1.183 (1.018-1.375) | 0.210 | 1.094 (1.016-1.179) | 0.267 | 0.955 (0.838-1.088) | 0.998 | 1.149 (1.063-1.241) | 0.056 | 1.116 (1.044-1.192) | 0.034 |
| COL18A1 | 1.161 (1.081-1.246) | 0.002 | 1.156 (1.005-1.330) | 0.253 | 1.145 (1.054-1.243) | 0.079 | 1.072 (0.939-1.223) | 0.998 | 1.144 (1.052-1.244) | 0.092 | 1.129 (1.051-1.212) | 0.030 |
| COL4A1 | 1.139 (1.067-1.216) | 0.004 | 1.283 (1.119-1.472) | 0.029 | 1.100 (1.020-1.185) | 0.240 | 1.055 (0.943-1.181) | 0.998 | 1.116 (1.031-1.206) | 0.163 | 1.127 (1.056-1.203) | 0.017 |
| COL5A1 | 1.112 (1.037-1.193) | 0.042 | 1.207 (1.050-1.389) | 0.127 | 1.077 (0.993-1.168) | 0.432 | 1.013 (0.890-1.153) | 0.998 | 1.137 (1.047-1.234) | 0.098 | 1.104 (1.029-1.184) | 0.085 |
| COL6A3 | 1.140 (1.062-1.223) | 0.009 | 1.159 (1.017-1.320) | 0.205 | 1.119 (1.028-1.218) | 0.205 | 1.016 (0.896-1.151) | 0.998 | 1.140 (1.047-1.240) | 0.102 | 1.102 (1.026-1.185) | 0.100 |
| COLEC12 | 1.131 (1.050-1.220) | 0.024 | 1.148 (0.997-1.323) | 0.277 | 1.111 (1.017-1.214) | 0.271 | 1.080 (0.945-1.235) | 0.984 | 1.095 (1.001-1.197) | 0.307 | 1.099 (1.019-1.186) | 0.133 |
| CPM | 1.162 (1.082-1.248) | 0.002 | 1.206 (1.035-1.404) | 0.162 | 1.134 (1.045-1.231) | 0.101 | 1.009 (0.895-1.138) | 0.998 | 1.073 (0.985-1.170) | 0.402 | 1.149 (1.069-1.234) | 0.011 |
| CREG1 | 1.107 (1.035-1.183) | 0.041 | 1.253 (1.094-1.435) | 0.055 | 1.096 (1.015-1.183) | 0.268 | 1.049 (0.935-1.177) | 0.998 | 1.086 (1.003-1.176) | 0.304 | 1.115 (1.043-1.191) | 0.036 |
| CRELD1 | 1.165 (1.081-1.257) | 0.003 | 1.224 (1.046-1.433) | 0.145 | 1.144 (1.050-1.246) | 0.092 | 1.080 (0.943-1.237) | 0.984 | 1.117 (1.021-1.220) | 0.219 | 1.146 (1.063-1.236) | 0.018 |
| CRIM1 | 1.116 (1.046-1.191) | 0.018 | 1.138 (0.985-1.314) | 0.329 | 1.113 (1.035-1.197) | 0.124 | 1.023 (0.907-1.155) | 0.998 | 1.120 (1.038-1.208) | 0.127 | 1.100 (1.031-1.174) | 0.070 |
| CRTAC1 | 0.881 (0.823-0.944) | 0.009 | 0.925 (0.794-1.078) | 0.591 | 0.874 (0.809-0.944) | 0.054 | 0.893 (0.784-1.018) | 0.818 | 0.903 (0.833-0.979) | 0.206 | 0.889 (0.830-0.952) | 0.027 |
| CSF1 | 1.148 (1.074-1.227) | 0.002 | 1.230 (1.066-1.419) | 0.092 | 1.128 (1.046-1.217) | 0.087 | 1.012 (0.899-1.139) | 0.998 | 1.125 (1.039-1.218) | 0.128 | 1.130 (1.057-1.209) | 0.017 |
| CST3 | 1.113 (1.035-1.197) | 0.050 | 1.164 (1.018-1.331) | 0.205 | 1.081 (0.992-1.178) | 0.448 | 1.005 (0.881-1.147) | 0.998 | 1.122 (1.029-1.222) | 0.172 | 1.078 (1.001-1.160) | 0.238 |
| CSTB | 1.132 (1.058-1.210) | 0.009 | 1.156 (0.999-1.338) | 0.268 | 1.120 (1.038-1.208) | 0.116 | 0.997 (0.868-1.145) | 0.998 | 1.134 (1.049-1.226) | 0.088 | 1.118 (1.045-1.196) | 0.033 |
| CTSF | 1.128 (1.054-1.207) | 0.012 | 1.067 (0.917-1.243) | 0.655 | 1.135 (1.052-1.225) | 0.069 | 1.097 (0.962-1.251) | 0.920 | 1.061 (0.979-1.149) | 0.452 | 1.125 (1.051-1.204) | 0.026 |
| CTSL | 1.127 (1.053-1.207) | 0.013 | 1.294 (1.123-1.492) | 0.029 | 1.083 (1.002-1.170) | 0.375 | 1.047 (0.930-1.177) | 0.998 | 1.067 (0.983-1.157) | 0.419 | 1.113 (1.039-1.191) | 0.050 |
| CTSZ | 1.108 (1.034-1.187) | 0.045 | 1.185 (1.027-1.368) | 0.184 | 1.089 (1.007-1.178) | 0.323 | 1.052 (0.924-1.196) | 0.998 | 1.057 (0.975-1.147) | 0.484 | 1.094 (1.022-1.172) | 0.114 |
| CXADR | 1.182 (1.113-1.256) | 3.63E-05 | 1.239 (1.083-1.417) | 0.065 | 1.161 (1.084-1.243) | 0.008 | 1.133 (1.015-1.265) | 0.671 | 1.095 (1.016-1.179) | 0.228 | 1.169 (1.100-1.243) | 3.01E-04 |
| CXCL17 | 1.146 (1.062-1.236) | 0.011 | 1.232 (1.042-1.457) | 0.158 | 1.112 (1.021-1.211) | 0.249 | 1.118 (0.973-1.285) | 0.843 | 1.127 (1.030-1.234) | 0.179 | 1.136 (1.053-1.226) | 0.030 |
| CXCL8 | 1.108 (1.040-1.180) | 0.027 | 1.062 (0.922-1.222) | 0.658 | 1.116 (1.039-1.198) | 0.105 | 1.036 (0.916-1.172) | 0.998 | 1.100 (1.020-1.185) | 0.205 | 1.103 (1.036-1.175) | 0.051 |
| DCBLD2 | 1.117 (1.043-1.196) | 0.026 | 1.025 (0.884-1.188) | 0.876 | 1.116 (1.030-1.210) | 0.184 | 0.953 (0.837-1.087) | 0.998 | 1.090 (1.006-1.181) | 0.291 | 1.092 (1.020-1.170) | 0.122 |
| DDAH1 | 1.106 (1.034-1.182) | 0.043 | 1.130 (0.978-1.306) | 0.352 | 1.090 (1.011-1.175) | 0.296 | 1.032 (0.908-1.173) | 0.998 | 1.085 (1.003-1.174) | 0.304 | 1.100 (1.028-1.176) | 0.082 |
| DEFB4A_DEFB4B | 1.167 (1.088-1.252) | 0.001 | 1.201 (1.035-1.395) | 0.163 | 1.159 (1.071-1.255) | 0.037 | 1.073 (0.934-1.232) | 0.998 | 1.161 (1.070-1.260) | 0.047 | 1.164 (1.086-1.249) | 0.003 |
| DPY30 | 1.122 (1.050-1.199) | 0.015 | 1.268 (1.089-1.476) | 0.072 | 1.086 (1.008-1.170) | 0.316 | 1.069 (0.935-1.222) | 0.998 | 1.101 (1.018-1.191) | 0.221 | 1.117 (1.044-1.194) | 0.034 |
| DTNB | 1.131 (1.055-1.212) | 0.012 | 1.241 (1.088-1.416) | 0.058 | 1.089 (1.005-1.180) | 0.343 | 1.083 (0.959-1.222) | 0.936 | 1.099 (1.011-1.195) | 0.265 | 1.114 (1.040-1.194) | 0.050 |
| ECHDC3 | 1.160 (1.084-1.241) | 0.001 | 1.167 (1.003-1.357) | 0.258 | 1.147 (1.063-1.238) | 0.041 | 1.124 (1.000-1.263) | 0.743 | 1.124 (1.037-1.219) | 0.149 | 1.154 (1.079-1.235) | 0.004 |
| ECHS1 | 1.117 (1.037-1.202) | 0.044 | 1.173 (0.988-1.392) | 0.302 | 1.101 (1.014-1.194) | 0.287 | 1.046 (0.910-1.202) | 0.998 | 1.128 (1.035-1.230) | 0.161 | 1.113 (1.034-1.199) | 0.074 |
| EDA2R | 1.159 (1.068-1.258) | 0.010 | 1.206 (1.042-1.395) | 0.145 | 1.135 (1.030-1.251) | 0.225 | 1.018 (0.867-1.194) | 0.998 | 1.173 (1.067-1.291) | 0.074 | 1.119 (1.031-1.215) | 0.099 |
| EDN1 | 1.122 (1.043-1.207) | 0.031 | 1.234 (1.061-1.435) | 0.106 | 1.077 (0.991-1.171) | 0.457 | 1.081 (0.941-1.242) | 0.991 | 1.103 (1.012-1.202) | 0.265 | 1.099 (1.022-1.182) | 0.120 |
| EFCAB14 | 1.110 (1.049-1.175) | 0.009 | 1.187 (1.062-1.325) | 0.073 | 1.085 (1.013-1.163) | 0.275 | 1.226 (1.105-1.360) | 0.059 | 1.050 (0.972-1.134) | 0.531 | 1.093 (1.031-1.160) | 0.062 |
| EFNA1 | 1.118 (1.044-1.196) | 0.025 | 1.204 (1.067-1.358) | 0.075 | 1.081 (0.997-1.171) | 0.399 | 1.135 (1.009-1.276) | 0.683 | 1.047 (0.965-1.137) | 0.579 | 1.089 (1.017-1.166) | 0.133 |
| EFNA4 | 1.144 (1.068-1.225) | 0.005 | 1.202 (1.065-1.358) | 0.078 | 1.109 (1.021-1.205) | 0.246 | 1.053 (0.929-1.194) | 0.998 | 1.110 (1.022-1.206) | 0.205 | 1.112 (1.037-1.193) | 0.061 |
| EGFR | 0.874 (0.819-0.933) | 0.002 | 0.826 (0.721-0.947) | 0.106 | 0.891 (0.827-0.960) | 0.099 | 0.972 (0.856-1.104) | 0.998 | 0.870 (0.806-0.938) | 0.047 | 0.889 (0.833-0.949) | 0.020 |
| ELN | 1.186 (1.082-1.301) | 0.008 | 1.387 (1.163-1.654) | 0.027 | 1.115 (1.002-1.240) | 0.375 | 1.121 (0.945-1.330) | 0.934 | 1.151 (1.033-1.284) | 0.190 | 1.172 (1.070-1.285) | 0.026 |
| ENPP7 | 1.130 (1.056-1.210) | 0.011 | 1.105 (0.946-1.290) | 0.494 | 1.139 (1.056-1.229) | 0.056 | 1.054 (0.934-1.190) | 0.998 | 1.095 (1.010-1.186) | 0.267 | 1.130 (1.056-1.210) | 0.018 |
| EPHA2 | 1.114 (1.040-1.193) | 0.032 | 1.142 (1.013-1.287) | 0.213 | 1.099 (1.012-1.193) | 0.296 | 1.087 (0.959-1.231) | 0.934 | 1.048 (0.964-1.139) | 0.582 | 1.083 (1.011-1.161) | 0.172 |
| ESYT2 | 1.108 (1.034-1.187) | 0.045 | 1.216 (1.040-1.420) | 0.155 | 1.079 (0.999-1.166) | 0.395 | 1.063 (0.932-1.214) | 0.998 | 1.113 (1.027-1.207) | 0.176 | 1.105 (1.032-1.184) | 0.075 |
| F11R | 1.105 (1.036-1.179) | 0.037 | 1.220 (1.052-1.416) | 0.128 | 1.074 (0.999-1.155) | 0.391 | 1.029 (0.909-1.166) | 0.998 | 1.095 (1.015-1.181) | 0.235 | 1.102 (1.032-1.175) | 0.068 |
| FABP3 | 1.159 (1.076-1.248) | 0.004 | 1.255 (1.096-1.437) | 0.050 | 1.113 (1.018-1.216) | 0.268 | 1.114 (0.975-1.272) | 0.836 | 1.129 (1.033-1.235) | 0.169 | 1.134 (1.052-1.222) | 0.032 |
| FABP4 | 1.143 (1.048-1.246) | 0.038 | 1.101 (0.925-1.310) | 0.567 | 1.129 (1.021-1.249) | 0.268 | 0.958 (0.818-1.123) | 0.998 | 1.156 (1.043-1.280) | 0.155 | 1.109 (1.017-1.210) | 0.154 |
| FAM3C | 1.136 (1.060-1.217) | 0.009 | 1.171 (1.031-1.332) | 0.159 | 1.108 (1.022-1.202) | 0.240 | 1.114 (0.985-1.260) | 0.816 | 1.109 (1.022-1.203) | 0.205 | 1.109 (1.035-1.189) | 0.068 |
| FAP | 0.879 (0.822-0.941) | 0.006 | 0.810 (0.703-0.934) | 0.084 | 0.898 (0.832-0.969) | 0.161 | 0.924 (0.818-1.044) | 0.952 | 0.877 (0.811-0.949) | 0.076 | 0.889 (0.831-0.951) | 0.025 |
| FCAMR | 1.128 (1.045-1.218) | 0.032 | 1.151 (0.968-1.368) | 0.375 | 1.114 (1.022-1.215) | 0.242 | 1.098 (0.949-1.271) | 0.955 | 1.077 (0.984-1.180) | 0.402 | 1.118 (1.035-1.208) | 0.075 |
| FGF23 | 1.150 (1.072-1.233) | 0.004 | 1.079 (0.933-1.247) | 0.584 | 1.163 (1.074-1.259) | 0.037 | 1.110 (0.987-1.247) | 0.797 | 1.104 (1.016-1.200) | 0.243 | 1.138 (1.061-1.220) | 0.017 |
| FGFBP3 | 0.898 (0.836-0.964) | 0.041 | 0.938 (0.803-1.096) | 0.670 | 0.887 (0.818-0.961) | 0.115 | 1.004 (0.884-1.140) | 0.998 | 0.867 (0.797-0.943) | 0.074 | 0.902 (0.840-0.968) | 0.075 |
| FGFR4 | 1.171 (1.093-1.254) | 0.001 | 1.188 (1.038-1.361) | 0.149 | 1.147 (1.060-1.242) | 0.054 | 1.119 (0.994-1.258) | 0.754 | 1.103 (1.015-1.199) | 0.243 | 1.153 (1.077-1.236) | 0.005 |
| FKBP4 | 1.125 (1.046-1.209) | 0.025 | 1.287 (1.087-1.524) | 0.083 | 1.091 (1.007-1.182) | 0.320 | 1.017 (0.883-1.171) | 0.998 | 1.128 (1.037-1.227) | 0.151 | 1.126 (1.047-1.211) | 0.035 |
| FN1 | 1.130 (1.046-1.220) | 0.031 | 1.135 (0.965-1.334) | 0.398 | 1.116 (1.022-1.218) | 0.246 | 0.940 (0.808-1.094) | 0.998 | 1.163 (1.063-1.272) | 0.074 | 1.117 (1.034-1.206) | 0.079 |
| FOLR1 | 1.108 (1.033-1.188) | 0.050 | 1.148 (1.009-1.306) | 0.228 | 1.088 (1.003-1.181) | 0.364 | 1.086 (0.958-1.231) | 0.936 | 1.057 (0.973-1.149) | 0.501 | 1.087 (1.013-1.166) | 0.154 |
| FSTL1 | 1.131 (1.059-1.209) | 0.009 | 1.297 (1.108-1.518) | 0.055 | 1.100 (1.020-1.186) | 0.242 | 1.141 (0.996-1.307) | 0.743 | 1.112 (1.028-1.204) | 0.169 | 1.120 (1.047-1.198) | 0.032 |
| FSTL3 | 1.178 (1.096-1.266) | 0.001 | 1.165 (1.018-1.333) | 0.205 | 1.175 (1.079-1.279) | 0.037 | 1.113 (0.979-1.265) | 0.821 | 1.148 (1.053-1.252) | 0.092 | 1.143 (1.063-1.230) | 0.017 |
| GALNT10 | 1.110 (1.039-1.185) | 0.032 | 1.087 (0.938-1.258) | 0.559 | 1.106 (1.027-1.191) | 0.186 | 1.117 (0.983-1.270) | 0.818 | 1.028 (0.950-1.112) | 0.746 | 1.102 (1.032-1.178) | 0.070 |
| GAST | 1.177 (1.102-1.257) | 2.17E-04 | 1.211 (1.068-1.375) | 0.077 | 1.157 (1.071-1.251) | 0.037 | 1.062 (0.943-1.196) | 0.998 | 1.119 (1.031-1.213) | 0.168 | 1.153 (1.079-1.233) | 0.003 |
| GBP1 | 1.127 (1.048-1.213) | 0.024 | 1.174 (0.987-1.396) | 0.304 | 1.119 (1.032-1.214) | 0.173 | 1.115 (0.962-1.293) | 0.904 | 1.114 (1.022-1.214) | 0.211 | 1.127 (1.048-1.213) | 0.035 |
| GDF15 | 1.204 (1.135-1.277) | 9.45E-07 | 1.243 (1.096-1.411) | 0.042 | 1.175 (1.093-1.263) | 0.007 | 1.079 (0.948-1.228) | 0.980 | 1.150 (1.065-1.241) | 0.047 | 1.174 (1.102-1.251) | 3.01E-04 |
| GFRA1 | 1.122 (1.045-1.206) | 0.027 | 1.196 (1.044-1.370) | 0.136 | 1.084 (0.997-1.179) | 0.401 | 1.047 (0.923-1.188) | 0.998 | 1.094 (1.005-1.191) | 0.300 | 1.096 (1.020-1.178) | 0.127 |
| GLA | 1.120 (1.039-1.208) | 0.042 | 0.955 (0.804-1.134) | 0.783 | 1.153 (1.060-1.253) | 0.059 | 1.000 (0.871-1.149) | 0.999 | 1.101 (1.008-1.203) | 0.288 | 1.113 (1.032-1.201) | 0.082 |
| GPNMB | 1.124 (1.049-1.204) | 0.019 | 1.144 (0.983-1.332) | 0.332 | 1.115 (1.031-1.205) | 0.168 | 1.006 (0.884-1.146) | 0.998 | 1.145 (1.056-1.241) | 0.074 | 1.114 (1.039-1.193) | 0.050 |
| GRPEL1 | 1.170 (1.097-1.247) | 2.70E-04 | 1.291 (1.123-1.484) | 0.029 | 1.132 (1.052-1.217) | 0.059 | 1.100 (0.978-1.236) | 0.836 | 1.117 (1.034-1.207) | 0.151 | 1.164 (1.091-1.241) | 0.002 |
| GUCA2A | 1.120 (1.047-1.198) | 0.019 | 1.171 (1.034-1.327) | 0.151 | 1.092 (1.009-1.181) | 0.310 | 1.145 (1.016-1.290) | 0.671 | 1.067 (0.986-1.156) | 0.404 | 1.095 (1.024-1.171) | 0.100 |
| GUSB | 1.145 (1.065-1.231) | 0.008 | 1.169 (1.011-1.353) | 0.227 | 1.143 (1.052-1.243) | 0.085 | 0.947 (0.836-1.073) | 0.998 | 1.144 (1.050-1.247) | 0.098 | 1.142 (1.063-1.228) | 0.017 |
| HAVCR1 | 1.137 (1.058-1.222) | 0.011 | 1.166 (1.018-1.337) | 0.205 | 1.110 (1.021-1.208) | 0.249 | 1.048 (0.923-1.191) | 0.998 | 1.083 (0.994-1.181) | 0.354 | 1.111 (1.034-1.194) | 0.074 |
| HAVCR2 | 1.110 (1.035-1.189) | 0.044 | 1.248 (1.092-1.426) | 0.055 | 1.061 (0.979-1.150) | 0.588 | 1.089 (0.963-1.232) | 0.924 | 1.033 (0.951-1.123) | 0.707 | 1.085 (1.012-1.164) | 0.160 |
| HGF | 1.119 (1.039-1.204) | 0.039 | 1.150 (0.984-1.345) | 0.328 | 1.100 (1.012-1.196) | 0.296 | 0.956 (0.836-1.093) | 0.998 | 1.155 (1.060-1.259) | 0.074 | 1.104 (1.026-1.188) | 0.101 |
| HPGDS | 0.883 (0.825-0.946) | 0.010 | 0.799 (0.687-0.929) | 0.083 | 0.906 (0.839-0.978) | 0.232 | 0.965 (0.850-1.096) | 0.998 | 0.889 (0.821-0.963) | 0.134 | 0.892 (0.833-0.955) | 0.032 |
| HS6ST2 | 1.122 (1.043-1.207) | 0.031 | 1.189 (1.043-1.355) | 0.134 | 1.090 (1.000-1.187) | 0.385 | 1.203 (1.063-1.361) | 0.358 | 1.076 (0.986-1.173) | 0.393 | 1.102 (1.026-1.185) | 0.100 |
| IDUA | 1.105 (1.033-1.182) | 0.047 | 1.126 (0.973-1.303) | 0.377 | 1.093 (1.012-1.179) | 0.288 | 1.050 (0.928-1.189) | 0.998 | 1.092 (1.009-1.182) | 0.274 | 1.102 (1.030-1.179) | 0.077 |
| IFI30 | 1.207 (1.117-1.304) | 2.74E-04 | 1.329 (1.138-1.552) | 0.029 | 1.163 (1.065-1.271) | 0.056 | 1.084 (0.944-1.245) | 0.979 | 1.152 (1.050-1.264) | 0.110 | 1.186 (1.098-1.281) | 0.003 |
| IGF2R | 1.107 (1.033-1.185) | 0.047 | 1.181 (1.009-1.382) | 0.234 | 1.085 (1.006-1.171) | 0.334 | 0.996 (0.877-1.130) | 0.998 | 1.114 (1.028-1.207) | 0.169 | 1.104 (1.031-1.182) | 0.077 |
| IGFBP4 | 1.206 (1.126-1.292) | 4.35E-05 | 1.246 (1.113-1.395) | 0.018 | 1.172 (1.076-1.277) | 0.037 | 1.120 (0.990-1.267) | 0.776 | 1.156 (1.062-1.257) | 0.070 | 1.164 (1.084-1.249) | 0.003 |
| IGFBP7 | 1.119 (1.046-1.197) | 0.021 | 1.127 (0.980-1.295) | 0.346 | 1.112 (1.030-1.201) | 0.177 | 1.029 (0.909-1.166) | 0.998 | 1.143 (1.057-1.235) | 0.068 | 1.098 (1.026-1.175) | 0.094 |
| IGFBPL1 | 1.115 (1.040-1.196) | 0.032 | 1.121 (0.976-1.288) | 0.366 | 1.110 (1.024-1.202) | 0.225 | 0.994 (0.881-1.122) | 0.998 | 1.116 (1.028-1.212) | 0.174 | 1.100 (1.025-1.179) | 0.100 |
| IGSF8 | 1.162 (1.090-1.239) | 0.001 | 1.304 (1.140-1.492) | 0.014 | 1.113 (1.034-1.199) | 0.144 | 1.060 (0.945-1.189) | 0.998 | 1.138 (1.054-1.230) | 0.074 | 1.144 (1.073-1.220) | 0.004 |
| IL10RB | 1.109 (1.041-1.183) | 0.026 | 1.147 (1.002-1.315) | 0.261 | 1.095 (1.017-1.178) | 0.256 | 1.108 (0.988-1.243) | 0.790 | 1.040 (0.961-1.126) | 0.623 | 1.092 (1.023-1.166) | 0.100 |
| IL12RB2 | 1.124 (1.053-1.200) | 0.011 | 1.140 (0.974-1.335) | 0.360 | 1.124 (1.046-1.208) | 0.080 | 1.097 (0.945-1.274) | 0.970 | 1.149 (1.068-1.236) | 0.040 | 1.125 (1.054-1.201) | 0.018 |
| IL15 | 1.108 (1.039-1.182) | 0.030 | 1.167 (1.025-1.328) | 0.179 | 1.088 (1.010-1.172) | 0.299 | 1.140 (1.001-1.299) | 0.739 | 1.071 (0.992-1.156) | 0.366 | 1.098 (1.029-1.171) | 0.077 |
| IL18 | 1.103 (1.034-1.176) | 0.041 | 1.134 (0.977-1.316) | 0.353 | 1.096 (1.020-1.178) | 0.235 | 0.951 (0.834-1.084) | 0.998 | 1.123 (1.042-1.210) | 0.102 | 1.101 (1.032-1.174) | 0.068 |
| IL1R1 | 1.108 (1.039-1.182) | 0.028 | 1.239 (1.090-1.408) | 0.050 | 1.071 (0.994-1.155) | 0.430 | 1.124 (1.004-1.258) | 0.716 | 1.045 (0.967-1.129) | 0.577 | 1.097 (1.029-1.171) | 0.077 |
| IL1RL1 | 1.133 (1.054-1.218) | 0.015 | 1.203 (1.019-1.421) | 0.211 | 1.112 (1.026-1.205) | 0.210 | 1.017 (0.887-1.166) | 0.998 | 1.123 (1.032-1.222) | 0.169 | 1.119 (1.040-1.202) | 0.052 |
| IL4R | 1.107 (1.038-1.180) | 0.031 | 1.154 (1.013-1.315) | 0.218 | 1.085 (1.008-1.168) | 0.312 | 1.086 (0.960-1.227) | 0.929 | 1.052 (0.974-1.135) | 0.507 | 1.089 (1.022-1.161) | 0.105 |
| IMMT | 1.163 (1.094-1.236) | 2.17E-04 | 1.147 (0.992-1.326) | 0.292 | 1.161 (1.085-1.242) | 0.007 | 1.130 (0.994-1.283) | 0.754 | 1.159 (1.079-1.245) | 0.019 | 1.153 (1.085-1.227) | 0.002 |
| ITGA11 | 0.884 (0.826-0.945) | 0.009 | 0.946 (0.821-1.089) | 0.683 | 0.872 (0.808-0.941) | 0.042 | 1.013 (0.897-1.145) | 0.998 | 0.899 (0.830-0.973) | 0.169 | 0.895 (0.837-0.957) | 0.034 |
| ITGAM | 0.864 (0.807-0.926) | 0.002 | 0.944 (0.809-1.101) | 0.699 | 0.843 (0.781-0.910) | 0.007 | 0.904 (0.797-1.026) | 0.850 | 0.899 (0.830-0.974) | 0.174 | 0.870 (0.813-0.932) | 0.007 |
| ITGAV | 0.863 (0.806-0.925) | 0.002 | 0.886 (0.761-1.031) | 0.384 | 0.862 (0.797-0.932) | 0.037 | 0.976 (0.859-1.107) | 0.998 | 0.886 (0.816-0.961) | 0.128 | 0.874 (0.816-0.937) | 0.011 |
| ITGB2 | 0.899 (0.838-0.965) | 0.042 | 0.965 (0.831-1.122) | 0.815 | 0.879 (0.812-0.952) | 0.083 | 0.875 (0.767-0.998) | 0.734 | 0.944 (0.869-1.025) | 0.477 | 0.904 (0.843-0.970) | 0.078 |
| ITGBL1 | 1.149 (1.068-1.235) | 0.006 | 1.201 (1.054-1.367) | 0.106 | 1.119 (1.026-1.220) | 0.228 | 1.041 (0.924-1.172) | 0.998 | 1.171 (1.075-1.277) | 0.047 | 1.133 (1.054-1.218) | 0.027 |
| JAM3 | 1.121 (1.050-1.196) | 0.015 | 1.224 (1.088-1.377) | 0.043 | 1.079 (1.000-1.164) | 0.388 | 1.069 (0.941-1.215) | 0.998 | 1.113 (1.031-1.202) | 0.163 | 1.112 (1.041-1.187) | 0.038 |
| LAIR1 | 1.117 (1.050-1.187) | 0.011 | 1.185 (1.051-1.336) | 0.104 | 1.089 (1.013-1.170) | 0.283 | 1.067 (0.929-1.225) | 0.998 | 1.099 (1.022-1.182) | 0.187 | 1.094 (1.027-1.165) | 0.080 |
| LAMB1 | 1.129 (1.049-1.214) | 0.022 | 1.205 (1.025-1.416) | 0.193 | 1.109 (1.022-1.204) | 0.242 | 1.055 (0.922-1.208) | 0.998 | 1.153 (1.058-1.256) | 0.076 | 1.117 (1.038-1.201) | 0.062 |
| LAYN | 1.117 (1.042-1.197) | 0.029 | 1.157 (1.022-1.311) | 0.189 | 1.093 (1.007-1.187) | 0.325 | 1.060 (0.938-1.197) | 0.998 | 1.057 (0.972-1.149) | 0.505 | 1.087 (1.014-1.166) | 0.151 |
| LETM1 | 1.154 (1.079-1.235) | 0.002 | 1.296 (1.117-1.505) | 0.039 | 1.116 (1.033-1.205) | 0.152 | 1.187 (1.036-1.359) | 0.516 | 1.126 (1.039-1.220) | 0.128 | 1.144 (1.069-1.225) | 0.009 |
| LGALS1 | 1.134 (1.059-1.214) | 0.009 | 1.252 (1.088-1.441) | 0.065 | 1.094 (1.012-1.183) | 0.296 | 1.005 (0.888-1.137) | 0.998 | 1.141 (1.054-1.236) | 0.076 | 1.119 (1.045-1.199) | 0.035 |
| LGALS4 | 1.140 (1.065-1.221) | 0.006 | 1.113 (0.965-1.283) | 0.421 | 1.123 (1.036-1.218) | 0.151 | 1.015 (0.893-1.153) | 0.998 | 1.095 (1.009-1.187) | 0.274 | 1.103 (1.029-1.183) | 0.085 |
| LGALS9 | 1.159 (1.076-1.248) | 0.004 | 1.158 (0.990-1.354) | 0.299 | 1.150 (1.057-1.250) | 0.072 | 1.025 (0.890-1.180) | 0.998 | 1.126 (1.032-1.229) | 0.169 | 1.135 (1.054-1.223) | 0.029 |
| LMNB2 | 1.165 (1.083-1.253) | 0.002 | 1.241 (1.093-1.408) | 0.046 | 1.121 (1.029-1.223) | 0.206 | 1.149 (1.023-1.291) | 0.586 | 1.088 (0.995-1.190) | 0.346 | 1.143 (1.063-1.229) | 0.017 |
| LONP1 | 1.125 (1.047-1.209) | 0.024 | 1.298 (1.103-1.527) | 0.065 | 1.086 (1.002-1.178) | 0.374 | 1.018 (0.885-1.171) | 0.998 | 1.157 (1.064-1.258) | 0.059 | 1.124 (1.046-1.208) | 0.038 |
| LRG1 | 1.122 (1.042-1.208) | 0.033 | 1.093 (0.923-1.293) | 0.584 | 1.121 (1.032-1.217) | 0.173 | 1.149 (0.991-1.333) | 0.766 | 1.082 (0.992-1.179) | 0.359 | 1.117 (1.038-1.203) | 0.065 |
| LTBR | 1.107 (1.036-1.184) | 0.038 | 1.174 (1.041-1.325) | 0.131 | 1.082 (1.001-1.170) | 0.383 | 1.041 (0.922-1.175) | 0.998 | 1.085 (1.002-1.174) | 0.304 | 1.087 (1.017-1.162) | 0.133 |
| MAD1L1 | 1.114 (1.047-1.186) | 0.015 | 1.296 (1.152-1.456) | 0.004 | 1.068 (0.992-1.150) | 0.457 | 1.119 (0.988-1.268) | 0.785 | 1.073 (0.993-1.160) | 0.358 | 1.109 (1.040-1.181) | 0.036 |
| MEP1B | 0.909 (0.852-0.970) | 0.050 | 0.896 (0.772-1.040) | 0.432 | 0.918 (0.854-0.988) | 0.287 | 0.900 (0.784-1.032) | 0.866 | 0.920 (0.853-0.992) | 0.279 | 0.916 (0.858-0.978) | 0.105 |
| MERTK | 1.124 (1.051-1.202) | 0.015 | 1.244 (1.085-1.428) | 0.065 | 1.083 (1.003-1.169) | 0.360 | 1.030 (0.917-1.157) | 0.998 | 1.075 (0.992-1.165) | 0.361 | 1.111 (1.039-1.188) | 0.048 |
| MMP7 | 1.159 (1.078-1.247) | 0.003 | 1.185 (1.022-1.373) | 0.196 | 1.136 (1.045-1.234) | 0.105 | 1.074 (0.936-1.232) | 0.998 | 1.118 (1.027-1.217) | 0.182 | 1.133 (1.054-1.217) | 0.027 |
| MRC1 | 1.136 (1.056-1.221) | 0.014 | 1.093 (0.934-1.279) | 0.560 | 1.136 (1.046-1.233) | 0.101 | 1.071 (0.929-1.235) | 0.998 | 1.115 (1.024-1.215) | 0.203 | 1.129 (1.049-1.214) | 0.033 |
| NAGPA | 1.138 (1.056-1.226) | 0.015 | 1.124 (0.956-1.323) | 0.445 | 1.136 (1.044-1.235) | 0.110 | 1.037 (0.907-1.187) | 0.998 | 1.138 (1.042-1.242) | 0.131 | 1.140 (1.058-1.228) | 0.025 |
| NCR3LG1 | 1.194 (1.110-1.285) | 2.83E-04 | 1.369 (1.200-1.562) | 0.002 | 1.136 (1.043-1.237) | 0.118 | 1.203 (1.060-1.365) | 0.407 | 1.108 (1.014-1.210) | 0.253 | 1.174 (1.092-1.263) | 0.003 |
| NCS1 | 1.126 (1.047-1.210) | 0.023 | 1.113 (0.960-1.289) | 0.443 | 1.114 (1.025-1.209) | 0.225 | 1.034 (0.907-1.180) | 0.998 | 1.036 (0.951-1.128) | 0.688 | 1.104 (1.028-1.187) | 0.096 |
| NECTIN2 | 1.201 (1.127-1.281) | 1.38E-05 | 1.272 (1.133-1.427) | 0.008 | 1.166 (1.080-1.258) | 0.026 | 1.128 (1.003-1.268) | 0.725 | 1.147 (1.061-1.239) | 0.058 | 1.179 (1.105-1.257) | 3.01E-04 |
| NHLRC3 | 1.161 (1.076-1.253) | 0.005 | 1.064 (0.900-1.259) | 0.702 | 1.172 (1.075-1.277) | 0.039 | 1.075 (0.945-1.222) | 0.992 | 1.125 (1.028-1.230) | 0.185 | 1.150 (1.065-1.241) | 0.017 |
| NPC2 | 1.151 (1.074-1.234) | 0.003 | 1.295 (1.125-1.491) | 0.029 | 1.103 (1.016-1.198) | 0.268 | 1.084 (0.944-1.244) | 0.980 | 1.113 (1.023-1.212) | 0.205 | 1.128 (1.050-1.211) | 0.030 |
| NPDC1 | 1.152 (1.080-1.229) | 0.001 | 1.163 (1.038-1.303) | 0.132 | 1.136 (1.051-1.228) | 0.079 | 1.087 (0.962-1.227) | 0.924 | 1.126 (1.041-1.217) | 0.110 | 1.123 (1.051-1.199) | 0.025 |
| NPPC | 1.117 (1.036-1.204) | 0.050 | 1.256 (1.102-1.431) | 0.039 | 1.050 (0.960-1.150) | 0.697 | 1.084 (0.949-1.239) | 0.974 | 1.102 (1.008-1.205) | 0.288 | 1.092 (1.012-1.178) | 0.169 |
| NT5C1A | 1.129 (1.060-1.203) | 0.006 | 1.235 (1.077-1.415) | 0.073 | 1.100 (1.022-1.184) | 0.228 | 1.098 (0.967-1.247) | 0.907 | 1.085 (1.000-1.177) | 0.318 | 1.116 (1.045-1.191) | 0.032 |
| NTproBNP | 1.114 (1.038-1.194) | 0.038 | 1.036 (0.907-1.182) | 0.787 | 1.142 (1.050-1.241) | 0.089 | 0.979 (0.858-1.118) | 0.998 | 1.148 (1.058-1.245) | 0.074 | 1.073 (0.999-1.153) | 0.252 |
| NTRK3 | 0.862 (0.804-0.923) | 0.002 | 0.882 (0.756-1.028) | 0.371 | 0.865 (0.801-0.935) | 0.037 | 0.886 (0.780-1.005) | 0.754 | 0.877 (0.809-0.951) | 0.083 | 0.871 (0.813-0.933) | 0.008 |
| OCLN | 1.202 (1.119-1.291) | 1.35E-04 | 1.240 (1.075-1.430) | 0.080 | 1.177 (1.083-1.278) | 0.034 | 1.165 (1.035-1.312) | 0.512 | 1.110 (1.016-1.213) | 0.246 | 1.173 (1.091-1.260) | 0.003 |
| ODAM | 0.895 (0.832-0.963) | 0.041 | 0.911 (0.773-1.073) | 0.558 | 0.891 (0.821-0.966) | 0.154 | 0.980 (0.878-1.092) | 0.998 | 0.867 (0.795-0.946) | 0.081 | 0.898 (0.836-0.966) | 0.069 |
| OSCAR | 1.148 (1.073-1.229) | 0.003 | 1.140 (0.987-1.315) | 0.315 | 1.150 (1.065-1.243) | 0.040 | 1.094 (0.963-1.241) | 0.921 | 1.106 (1.021-1.197) | 0.205 | 1.133 (1.059-1.213) | 0.017 |
| OXCT1 | 1.124 (1.050-1.203) | 0.017 | 1.260 (1.079-1.472) | 0.083 | 1.091 (1.011-1.178) | 0.296 | 1.068 (0.936-1.218) | 0.998 | 1.116 (1.031-1.209) | 0.168 | 1.123 (1.049-1.203) | 0.030 |
| PALLD | 1.094 (1.031-1.161) | 0.041 | 1.175 (1.019-1.356) | 0.205 | 1.081 (1.012-1.155) | 0.284 | 1.097 (0.958-1.255) | 0.924 | 1.098 (1.026-1.176) | 0.168 | 1.092 (1.029-1.159) | 0.069 |
| PALM2 | 1.177 (1.091-1.269) | 0.002 | 1.249 (1.091-1.431) | 0.056 | 1.132 (1.036-1.238) | 0.172 | 1.072 (0.944-1.219) | 0.998 | 1.119 (1.019-1.229) | 0.234 | 1.149 (1.066-1.239) | 0.017 |
| PENK | 1.136 (1.064-1.213) | 0.005 | 1.164 (1.061-1.278) | 0.059 | 1.099 (1.013-1.193) | 0.294 | 1.107 (1.011-1.212) | 0.671 | 1.094 (1.008-1.187) | 0.283 | 1.100 (1.031-1.174) | 0.070 |
| PGA4 | 1.159 (1.076-1.248) | 0.004 | 1.173 (1.024-1.345) | 0.189 | 1.141 (1.045-1.246) | 0.115 | 1.158 (1.027-1.305) | 0.568 | 1.062 (0.973-1.159) | 0.485 | 1.135 (1.056-1.221) | 0.026 |
| PI3 | 1.158 (1.085-1.236) | 0.001 | 1.175 (1.034-1.335) | 0.151 | 1.150 (1.067-1.240) | 0.037 | 1.027 (0.903-1.167) | 0.998 | 1.178 (1.092-1.271) | 0.012 | 1.139 (1.067-1.217) | 0.009 |
| PIGR | 1.156 (1.081-1.237) | 0.002 | 1.223 (1.066-1.404) | 0.086 | 1.123 (1.039-1.212) | 0.115 | 1.077 (0.947-1.226) | 0.984 | 1.097 (1.011-1.189) | 0.265 | 1.136 (1.061-1.216) | 0.015 |
| PIK3IP1 | 1.114 (1.040-1.194) | 0.032 | 1.203 (1.072-1.351) | 0.065 | 1.065 (0.980-1.157) | 0.587 | 1.171 (1.043-1.316) | 0.408 | 1.039 (0.956-1.130) | 0.647 | 1.079 (1.007-1.157) | 0.198 |
| PILRA | 1.112 (1.036-1.193) | 0.042 | 1.224 (1.053-1.424) | 0.128 | 1.080 (0.998-1.169) | 0.396 | 1.081 (0.944-1.237) | 0.984 | 1.047 (0.965-1.136) | 0.579 | 1.096 (1.022-1.175) | 0.116 |
| PILRB | 1.125 (1.048-1.207) | 0.022 | 1.175 (1.007-1.370) | 0.245 | 1.108 (1.023-1.200) | 0.232 | 1.114 (0.968-1.281) | 0.866 | 1.067 (0.984-1.158) | 0.417 | 1.113 (1.037-1.194) | 0.064 |
| PLA2G15 | 1.165 (1.089-1.245) | 0.001 | 1.215 (1.055-1.400) | 0.114 | 1.150 (1.065-1.241) | 0.039 | 0.952 (0.840-1.079) | 0.998 | 1.155 (1.067-1.251) | 0.047 | 1.154 (1.080-1.234) | 0.003 |
| PLAUR | 1.204 (1.123-1.291) | 5.89E-05 | 1.208 (1.046-1.395) | 0.136 | 1.196 (1.104-1.295) | 0.007 | 1.056 (0.928-1.200) | 0.998 | 1.196 (1.101-1.300) | 0.012 | 1.180 (1.099-1.266) | 0.002 |
| PLIN3 | 1.135 (1.064-1.210) | 0.005 | 1.212 (1.050-1.399) | 0.128 | 1.110 (1.032-1.193) | 0.144 | 0.976 (0.859-1.109) | 0.998 | 1.159 (1.076-1.249) | 0.028 | 1.129 (1.058-1.205) | 0.015 |
| PLSCR3 | 1.110 (1.036-1.190) | 0.043 | 1.279 (1.086-1.507) | 0.081 | 1.074 (0.994-1.160) | 0.427 | 1.135 (0.991-1.298) | 0.766 | 1.095 (1.009-1.188) | 0.274 | 1.107 (1.032-1.187) | 0.075 |
| PLXNB2 | 1.104 (1.032-1.180) | 0.047 | 1.089 (0.948-1.251) | 0.518 | 1.094 (1.013-1.182) | 0.288 | 1.059 (0.933-1.201) | 0.998 | 1.071 (0.989-1.159) | 0.375 | 1.093 (1.022-1.169) | 0.111 |
| PRAP1 | 1.147 (1.057-1.244) | 0.021 | 1.164 (0.976-1.390) | 0.343 | 1.121 (1.022-1.231) | 0.256 | 1.105 (0.953-1.281) | 0.929 | 1.072 (0.975-1.180) | 0.458 | 1.123 (1.035-1.219) | 0.081 |
| PRRT3 | 1.140 (1.063-1.224) | 0.008 | 1.166 (1.018-1.334) | 0.204 | 1.128 (1.040-1.225) | 0.124 | 1.314 (1.161-1.488) | 0.013 | 1.044 (0.959-1.137) | 0.611 | 1.120 (1.043-1.202) | 0.044 |
| PRSS8 | 1.182 (1.098-1.272) | 0.001 | 1.172 (1.000-1.374) | 0.265 | 1.161 (1.066-1.265) | 0.054 | 1.109 (0.973-1.264) | 0.850 | 1.109 (1.016-1.211) | 0.244 | 1.154 (1.072-1.243) | 0.011 |
| PSAP | 1.134 (1.070-1.203) | 0.002 | 1.414 (1.220-1.639) | 0.003 | 1.086 (1.013-1.165) | 0.275 | 1.178 (1.031-1.346) | 0.557 | 1.108 (1.031-1.191) | 0.151 | 1.126 (1.060-1.196) | 0.009 |
| PTPRF | 0.892 (0.833-0.955) | 0.021 | 0.922 (0.796-1.069) | 0.569 | 0.892 (0.825-0.964) | 0.124 | 0.888 (0.779-1.013) | 0.787 | 0.901 (0.832-0.976) | 0.185 | 0.892 (0.833-0.955) | 0.032 |
| PTPRS | 0.898 (0.841-0.960) | 0.026 | 0.964 (0.840-1.105) | 0.781 | 0.888 (0.824-0.957) | 0.089 | 0.939 (0.833-1.059) | 0.998 | 0.913 (0.845-0.986) | 0.245 | 0.895 (0.838-0.956) | 0.030 |
| PVR | 1.153 (1.084-1.227) | 0.001 | 1.188 (1.036-1.361) | 0.152 | 1.141 (1.064-1.223) | 0.037 | 1.148 (1.018-1.295) | 0.659 | 1.115 (1.036-1.200) | 0.128 | 1.147 (1.078-1.220) | 0.003 |
| QSOX1 | 1.082 (1.027-1.141) | 0.043 | 1.142 (0.988-1.319) | 0.312 | 1.076 (1.016-1.139) | 0.235 | 1.158 (1.040-1.288) | 0.408 | 1.064 (0.997-1.135) | 0.339 | 1.079 (1.023-1.138) | 0.078 |
| RAB6A | 0.906 (0.848-0.969) | 0.047 | 0.904 (0.781-1.047) | 0.469 | 0.917 (0.851-0.989) | 0.293 | 0.972 (0.875-1.080) | 0.998 | 0.923 (0.854-0.999) | 0.306 | 0.919 (0.859-0.982) | 0.124 |
| RBFOX3 | 1.178 (1.101-1.259) | 2.73E-04 | 1.272 (1.127-1.436) | 0.014 | 1.138 (1.051-1.232) | 0.080 | 1.093 (0.973-1.229) | 0.869 | 1.129 (1.037-1.229) | 0.151 | 1.149 (1.075-1.229) | 0.005 |
| REN | 1.146 (1.074-1.224) | 0.002 | 1.056 (0.941-1.185) | 0.622 | 1.173 (1.079-1.276) | 0.037 | 0.989 (0.871-1.124) | 0.998 | 1.164 (1.078-1.257) | 0.028 | 1.105 (1.033-1.181) | 0.069 |
| RNASE1 | 1.142 (1.073-1.215) | 0.002 | 1.220 (1.099-1.355) | 0.022 | 1.098 (1.015-1.188) | 0.275 | 1.198 (1.070-1.342) | 0.271 | 1.107 (1.024-1.196) | 0.185 | 1.114 (1.044-1.188) | 0.032 |
| RNASE4 | 1.132 (1.060-1.208) | 0.007 | 1.192 (1.051-1.352) | 0.106 | 1.099 (1.017-1.188) | 0.264 | 1.201 (1.050-1.373) | 0.408 | 1.093 (1.008-1.186) | 0.288 | 1.113 (1.040-1.190) | 0.044 |
| RNASE6 | 1.160 (1.096-1.227) | 7.21E-05 | 1.244 (1.132-1.367) | 0.003 | 1.118 (1.040-1.202) | 0.099 | 1.082 (0.955-1.226) | 0.960 | 1.159 (1.085-1.237) | 0.010 | 1.140 (1.075-1.210) | 0.003 |
| RNASET2 | 1.144 (1.076-1.216) | 0.001 | 1.257 (1.146-1.379) | 0.001 | 1.087 (1.009-1.171) | 0.310 | 1.099 (0.971-1.245) | 0.876 | 1.137 (1.058-1.221) | 0.056 | 1.127 (1.059-1.200) | 0.011 |
| RTBDN | 1.109 (1.038-1.184) | 0.032 | 1.155 (1.000-1.333) | 0.265 | 1.092 (1.013-1.176) | 0.283 | 1.142 (1.007-1.295) | 0.693 | 1.054 (0.974-1.141) | 0.502 | 1.092 (1.022-1.166) | 0.107 |
| SCARB2 | 1.170 (1.090-1.256) | 0.001 | 1.205 (1.066-1.362) | 0.077 | 1.146 (1.052-1.249) | 0.089 | 1.013 (0.884-1.160) | 0.998 | 1.159 (1.065-1.262) | 0.059 | 1.132 (1.053-1.217) | 0.027 |
| SCARF1 | 1.106 (1.037-1.180) | 0.034 | 1.215 (1.057-1.397) | 0.106 | 1.077 (1.001-1.159) | 0.378 | 1.039 (0.917-1.177) | 0.998 | 1.114 (1.033-1.201) | 0.151 | 1.104 (1.034-1.177) | 0.061 |
| SCRG1 | 1.114 (1.035-1.199) | 0.050 | 1.165 (1.009-1.345) | 0.234 | 1.087 (0.997-1.185) | 0.399 | 1.128 (0.967-1.317) | 0.856 | 1.106 (1.015-1.206) | 0.247 | 1.096 (1.017-1.181) | 0.137 |
| SEPTIN8 | 1.121 (1.048-1.199) | 0.018 | 1.262 (1.130-1.409) | 0.008 | 1.065 (0.984-1.152) | 0.551 | 1.141 (1.023-1.273) | 0.577 | 1.065 (0.980-1.157) | 0.436 | 1.102 (1.030-1.178) | 0.077 |
| SHISA5 | 1.145 (1.076-1.218) | 0.001 | 1.183 (1.067-1.312) | 0.060 | 1.117 (1.033-1.207) | 0.153 | 1.129 (0.986-1.293) | 0.791 | 1.122 (1.041-1.210) | 0.110 | 1.118 (1.048-1.192) | 0.026 |
| SIGLEC10 | 1.108 (1.036-1.184) | 0.038 | 1.258 (1.106-1.431) | 0.033 | 1.059 (0.981-1.144) | 0.588 | 1.076 (0.950-1.218) | 0.979 | 1.076 (0.994-1.164) | 0.355 | 1.095 (1.024-1.171) | 0.100 |
| SIGLEC8 | 1.139 (1.059-1.225) | 0.011 | 1.314 (1.118-1.544) | 0.048 | 1.097 (1.011-1.190) | 0.303 | 1.198 (1.053-1.363) | 0.408 | 1.078 (0.989-1.175) | 0.373 | 1.128 (1.049-1.213) | 0.034 |
| SLMAP | 1.119 (1.046-1.197) | 0.022 | 1.147 (0.982-1.338) | 0.332 | 1.107 (1.026-1.194) | 0.199 | 1.047 (0.916-1.197) | 0.998 | 1.128 (1.042-1.221) | 0.110 | 1.114 (1.041-1.192) | 0.044 |
| SMPD1 | 1.156 (1.082-1.234) | 0.001 | 1.195 (1.038-1.375) | 0.150 | 1.147 (1.065-1.235) | 0.038 | 1.008 (0.900-1.130) | 0.998 | 1.109 (1.025-1.200) | 0.180 | 1.145 (1.072-1.222) | 0.005 |
| SNAP25 | 1.127 (1.048-1.211) | 0.022 | 1.339 (1.141-1.572) | 0.029 | 1.070 (0.986-1.161) | 0.510 | 1.150 (1.006-1.315) | 0.699 | 1.093 (1.004-1.190) | 0.304 | 1.122 (1.044-1.206) | 0.044 |
| SORCS2 | 1.146 (1.070-1.227) | 0.004 | 1.197 (1.045-1.370) | 0.132 | 1.115 (1.030-1.206) | 0.180 | 1.094 (0.965-1.241) | 0.920 | 1.117 (1.030-1.211) | 0.169 | 1.114 (1.040-1.193) | 0.050 |
| SPINK1 | 1.123 (1.054-1.196) | 0.009 | 1.133 (1.003-1.278) | 0.255 | 1.107 (1.028-1.193) | 0.184 | 1.101 (0.989-1.224) | 0.787 | 1.070 (0.990-1.157) | 0.372 | 1.092 (1.024-1.165) | 0.099 |
| SPON2 | 1.118 (1.044-1.199) | 0.027 | 1.137 (0.987-1.309) | 0.320 | 1.101 (1.016-1.193) | 0.268 | 1.034 (0.909-1.176) | 0.998 | 1.101 (1.014-1.194) | 0.246 | 1.091 (1.017-1.170) | 0.133 |
| SRPX | 1.164 (1.077-1.258) | 0.005 | 1.300 (1.108-1.526) | 0.056 | 1.126 (1.030-1.231) | 0.204 | 1.132 (0.975-1.314) | 0.821 | 1.196 (1.092-1.310) | 0.028 | 1.153 (1.067-1.246) | 0.017 |
| STX8 | 1.101 (1.035-1.171) | 0.035 | 1.123 (0.976-1.293) | 0.365 | 1.091 (1.018-1.168) | 0.242 | 0.994 (0.880-1.123) | 0.998 | 1.118 (1.041-1.201) | 0.100 | 1.101 (1.035-1.171) | 0.051 |
| SUSD4 | 1.120 (1.050-1.194) | 0.013 | 1.128 (0.981-1.296) | 0.341 | 1.120 (1.042-1.204) | 0.093 | 1.112 (1.002-1.233) | 0.725 | 1.087 (1.004-1.177) | 0.300 | 1.111 (1.042-1.185) | 0.035 |
| SYT1 | 1.112 (1.050-1.178) | 0.009 | 1.181 (1.081-1.289) | 0.024 | 1.075 (1.000-1.156) | 0.385 | 1.138 (1.046-1.239) | 0.330 | 1.074 (0.997-1.157) | 0.339 | 1.100 (1.038-1.167) | 0.036 |
| TAFA5 | 1.120 (1.048-1.197) | 0.017 | 1.137 (1.003-1.288) | 0.257 | 1.100 (1.018-1.189) | 0.260 | 1.098 (0.975-1.236) | 0.851 | 1.106 (1.023-1.197) | 0.202 | 1.091 (1.020-1.166) | 0.120 |
| TCOF1 | 1.116 (1.047-1.189) | 0.017 | 1.162 (0.975-1.384) | 0.346 | 1.106 (1.031-1.186) | 0.144 | 1.256 (1.086-1.454) | 0.280 | 1.081 (0.999-1.168) | 0.321 | 1.116 (1.046-1.191) | 0.030 |
| TFF2 | 1.146 (1.072-1.224) | 0.003 | 1.177 (1.026-1.349) | 0.184 | 1.126 (1.044-1.215) | 0.095 | 1.180 (1.044-1.333) | 0.408 | 1.058 (0.976-1.147) | 0.477 | 1.122 (1.049-1.199) | 0.028 |
| TFF3 | 1.122 (1.062-1.186) | 0.002 | 1.221 (1.074-1.388) | 0.073 | 1.100 (1.033-1.171) | 0.110 | 0.998 (0.900-1.106) | 0.998 | 1.089 (1.015-1.167) | 0.224 | 1.102 (1.041-1.167) | 0.030 |
| TGFB1 | 1.106 (1.036-1.180) | 0.037 | 1.157 (1.017-1.315) | 0.204 | 1.090 (1.012-1.175) | 0.288 | 1.002 (0.879-1.142) | 0.998 | 1.116 (1.035-1.204) | 0.140 | 1.102 (1.032-1.177) | 0.069 |
| TGFBR2 | 1.131 (1.056-1.210) | 0.010 | 1.161 (1.040-1.295) | 0.119 | 1.104 (1.016-1.201) | 0.275 | 1.145 (1.018-1.287) | 0.659 | 1.060 (0.974-1.153) | 0.483 | 1.105 (1.031-1.184) | 0.076 |
| THBS2 | 1.137 (1.067-1.210) | 0.003 | 1.150 (1.018-1.299) | 0.197 | 1.128 (1.048-1.213) | 0.079 | 1.043 (0.939-1.158) | 0.998 | 1.128 (1.046-1.217) | 0.092 | 1.125 (1.056-1.198) | 0.015 |
| THY1 | 1.120 (1.044-1.201) | 0.026 | 1.231 (1.077-1.406) | 0.072 | 1.080 (0.996-1.172) | 0.402 | 1.077 (0.952-1.219) | 0.974 | 1.090 (1.003-1.184) | 0.304 | 1.099 (1.025-1.179) | 0.100 |
| TIMP1 | 1.117 (1.041-1.197) | 0.031 | 1.193 (1.032-1.379) | 0.168 | 1.091 (1.007-1.181) | 0.317 | 1.021 (0.895-1.165) | 0.998 | 1.106 (1.019-1.200) | 0.224 | 1.102 (1.028-1.182) | 0.092 |
| TNFRSF10A | 1.162 (1.089-1.241) | 0.001 | 1.163 (1.013-1.336) | 0.219 | 1.143 (1.058-1.234) | 0.054 | 1.031 (0.906-1.174) | 0.998 | 1.102 (1.016-1.195) | 0.239 | 1.137 (1.063-1.217) | 0.014 |
| TNFRSF10B | 1.153 (1.109-1.198) | 1.78E-09 | 1.167 (1.072-1.269) | 0.029 | 1.144 (1.094-1.196) | 1.15E-05 | 1.110 (1.009-1.222) | 0.671 | 1.139 (1.085-1.196) | 2.98E-04 | 1.149 (1.103-1.196) | 6.20E-08 |
| TNFRSF12A | 1.140 (1.063-1.222) | 0.008 | 1.148 (1.007-1.309) | 0.241 | 1.121 (1.032-1.217) | 0.173 | 1.139 (1.012-1.282) | 0.671 | 1.064 (0.978-1.158) | 0.457 | 1.111 (1.036-1.192) | 0.066 |
| TNFRSF13B | 1.105 (1.038-1.176) | 0.027 | 1.178 (1.038-1.336) | 0.144 | 1.083 (1.008-1.163) | 0.316 | 1.040 (0.917-1.179) | 0.998 | 1.116 (1.040-1.198) | 0.100 | 1.091 (1.024-1.162) | 0.098 |
| TNFRSF14 | 1.180 (1.105-1.259) | 1.59E-04 | 1.250 (1.109-1.410) | 0.027 | 1.146 (1.061-1.237) | 0.046 | 1.072 (0.955-1.203) | 0.974 | 1.157 (1.070-1.251) | 0.047 | 1.156 (1.082-1.234) | 0.003 |
| TNFRSF1A | 1.166 (1.086-1.252) | 0.001 | 1.279 (1.129-1.449) | 0.014 | 1.116 (1.025-1.214) | 0.227 | 1.109 (0.984-1.251) | 0.821 | 1.117 (1.026-1.217) | 0.192 | 1.133 (1.054-1.217) | 0.027 |
| TNFRSF1B | 1.115 (1.056-1.176) | 0.003 | 1.153 (1.053-1.262) | 0.071 | 1.093 (1.023-1.167) | 0.197 | 1.098 (0.979-1.233) | 0.836 | 1.078 (1.006-1.155) | 0.288 | 1.099 (1.039-1.163) | 0.030 |
| TNFRSF6B | 1.114 (1.040-1.193) | 0.032 | 1.114 (0.969-1.282) | 0.403 | 1.101 (1.018-1.192) | 0.260 | 1.028 (0.911-1.160) | 0.998 | 1.104 (1.018-1.198) | 0.225 | 1.097 (1.024-1.174) | 0.102 |
| TNFSF11 | 0.899 (0.840-0.962) | 0.032 | 0.910 (0.779-1.064) | 0.529 | 0.909 (0.842-0.981) | 0.245 | 1.002 (0.887-1.132) | 0.998 | 0.915 (0.845-0.991) | 0.274 | 0.909 (0.850-0.973) | 0.090 |
| TPSG1 | 1.106 (1.040-1.175) | 0.022 | 1.124 (0.979-1.291) | 0.351 | 1.088 (1.014-1.168) | 0.275 | 1.001 (0.882-1.137) | 0.998 | 1.084 (1.006-1.167) | 0.290 | 1.092 (1.025-1.163) | 0.092 |
| TREM2 | 1.123 (1.046-1.206) | 0.026 | 1.036 (0.885-1.213) | 0.823 | 1.114 (1.042-1.191) | 0.080 | 1.023 (0.903-1.159) | 0.998 | 1.097 (1.008-1.194) | 0.288 | 1.109 (1.033-1.191) | 0.075 |
| UBE2L6 | 1.126 (1.048-1.209) | 0.022 | 1.267 (1.071-1.499) | 0.104 | 1.145 (1.057-1.240) | 0.059 | 1.089 (0.945-1.256) | 0.974 | 1.119 (1.030-1.217) | 0.169 | 1.124 (1.047-1.208) | 0.035 |
| ULBP2 | 1.131 (1.058-1.210) | 0.009 | 1.229 (1.075-1.404) | 0.073 | 1.094 (1.010-1.184) | 0.303 | 1.094 (0.976-1.226) | 0.855 | 1.069 (0.987-1.159) | 0.395 | 1.112 (1.040-1.189) | 0.047 |
| VSIG2 | 1.235 (1.154-1.322) | 1.03E-06 | 1.300 (1.155-1.463) | 0.004 | 1.092 (1.011-1.180) | 0.299 | 1.116 (0.994-1.252) | 0.756 | 1.144 (1.051-1.246) | 0.092 | 1.202 (1.122-1.287) | 2.26E-04 |
| VSIG4 | 1.136 (1.057-1.221) | 0.013 | 1.192 (1.041-1.365) | 0.144 | 1.187 (1.092-1.291) | 0.021 | 1.084 (0.948-1.240) | 0.974 | 1.102 (1.012-1.200) | 0.265 | 1.099 (1.022-1.182) | 0.118 |
| WFDC2 | 1.190 (1.108-1.278) | 2.73E-04 | 1.217 (1.075-1.377) | 0.065 | 1.107 (1.017-1.204) | 0.268 | 1.094 (0.961-1.246) | 0.924 | 1.157 (1.061-1.262) | 0.074 | 1.154 (1.073-1.242) | 0.009 |
| WFIKKN1 | 0.823 (0.766-0.884) | 4.35E-05 | 0.797 (0.677-0.939) | 0.111 | 1.169 (1.073-1.273) | 0.040 | 0.897 (0.783-1.028) | 0.846 | 0.838 (0.771-0.912) | 0.016 | 0.829 (0.771-0.890) | 2.88E-04 |
| YAP1 | 1.152 (1.067-1.245) | 0.009 | 1.164 (1.025-1.322) | 0.177 | 0.828 (0.764-0.898) | 0.007 | 1.105 (0.982-1.242) | 0.821 | 1.088 (0.989-1.197) | 0.369 | 1.118 (1.036-1.206) | 0.073 |
| ZNRD2 | 1.109 (1.035-1.188) | 0.044 | 1.102 (0.946-1.283) | 0.501 | 1.129 (1.030-1.239) | 0.214 | 1.093 (0.962-1.241) | 0.924 | 1.092 (1.007-1.185) | 0.288 | 1.108 (1.034-1.187) | 0.068 |
| TPSG1 | 1.106 (1.040-1.175) | 0.022 | 1.036 (0.885-1.213) | 0.823 | 1.103 (1.020-1.192) | 0.242 | 1.023 (0.903-1.159) | 0.998 | 1.097 (1.008-1.194) | 0.288 | 1.109 (1.033-1.191) | 0.075 |

| **Supplementary Table 34. Detailed protein associations with MDD in the primary and sensitivity analyses.** | | | | | | | | | | | | |
| --- | --- | --- | --- | --- | --- | --- | --- | --- | --- | --- | --- | --- |
| **Protein** | **Primary analysis** | | **Lipid-lowering medication users** | | **No lipid-lowering medications** | | **Psychotropic medication users** | | **No psychotropic medications** | | **Additional adjustment** | |
|  | **HR (95% CI)** | **FDR** | **HR (95% CI)** | **FDR** | **HR (95% CI)** | **FDR** | **HR (95% CI)** | **FDR** | **HR (95% CI)** | **FDR** | **HR (95% CI)** | **FDR** |
| A1BG | 1.178 (1.081-1.285) | 0.005 | 1.249 (1.047-1.490) | 0.141 | 1.151 (1.043-1.272) | 0.075 | 1.200 (1.000-1.439) | 0.272 | 1.165 (1.056-1.285) | 0.054 | 1.180 (1.083-1.287) | 0.007 |
| ACAA1 | 1.117 (1.036-1.204) | 0.036 | 1.056 (0.906-1.231) | 0.727 | 1.120 (1.028-1.221) | 0.113 | 1.080 (0.933-1.250) | 0.622 | 1.083 (0.993-1.181) | 0.383 | 1.112 (1.031-1.198) | 0.060 |
| ACE2 | 1.156 (1.069-1.249) | 0.006 | 0.952 (0.819-1.105) | 0.746 | 1.227 (1.120-1.345) | 0.002 | 0.966 (0.836-1.115) | 0.862 | 1.140 (1.041-1.250) | 0.083 | 1.136 (1.050-1.228) | 0.027 |
| ACVRL1 | 1.164 (1.078-1.258) | 0.003 | 1.204 (1.056-1.373) | 0.117 | 1.147 (1.044-1.260) | 0.065 | 1.052 (0.904-1.223) | 0.783 | 1.158 (1.060-1.267) | 0.037 | 1.126 (1.042-1.216) | 0.037 |
| ADA2 | 1.169 (1.085-1.260) | 0.001 | 1.062 (0.919-1.227) | 0.663 | 1.204 (1.105-1.313) | 0.003 | 1.022 (0.878-1.191) | 0.914 | 1.162 (1.067-1.265) | 0.025 | 1.151 (1.068-1.241) | 0.008 |
| ADAM12 | 1.105 (1.041-1.172) | 0.014 | 1.223 (1.050-1.424) | 0.119 | 1.084 (1.011-1.163) | 0.195 | 1.106 (0.932-1.313) | 0.566 | 1.095 (1.022-1.173) | 0.125 | 1.095 (1.030-1.164) | 0.047 |
| ADAMTS13 | 0.890 (0.827-0.958) | 0.023 | 0.950 (0.823-1.097) | 0.724 | 0.864 (0.793-0.942) | 0.027 | 0.845 (0.729-0.979) | 0.223 | 0.918 (0.844-0.999) | 0.309 | 0.894 (0.831-0.962) | 0.037 |
| ADAMTS8 | 0.841 (0.781-0.906) | 3.60E-04 | 0.896 (0.770-1.042) | 0.383 | 0.830 (0.763-0.904) | 0.003 | 0.887 (0.764-1.030) | 0.388 | 0.853 (0.784-0.928) | 0.015 | 0.847 (0.786-0.912) | 0.001 |
| ADAMTSL2 | 1.157 (1.068-1.254) | 0.007 | 1.101 (0.951-1.275) | 0.450 | 1.155 (1.049-1.273) | 0.058 | 1.041 (0.895-1.212) | 0.843 | 1.156 (1.054-1.269) | 0.054 | 1.139 (1.050-1.235) | 0.028 |
| ADGRF5 | 1.144 (1.053-1.243) | 0.019 | 1.259 (1.058-1.498) | 0.119 | 1.111 (1.011-1.222) | 0.218 | 1.252 (1.054-1.487) | 0.205 | 1.107 (1.008-1.217) | 0.259 | 1.143 (1.052-1.242) | 0.028 |
| ADGRG1 | 1.114 (1.040-1.194) | 0.025 | 1.032 (0.917-1.163) | 0.796 | 1.154 (1.059-1.257) | 0.029 | 1.054 (0.921-1.205) | 0.734 | 1.103 (1.018-1.197) | 0.183 | 1.094 (1.019-1.174) | 0.102 |
| ADM | 1.158 (1.057-1.269) | 0.020 | 1.152 (0.966-1.373) | 0.333 | 1.149 (1.033-1.278) | 0.118 | 0.935 (0.787-1.112) | 0.737 | 1.193 (1.075-1.324) | 0.033 | 1.115 (1.018-1.221) | 0.128 |
| AGR2 | 1.139 (1.059-1.226) | 0.008 | 1.190 (1.026-1.381) | 0.156 | 1.116 (1.025-1.214) | 0.122 | 1.100 (0.944-1.282) | 0.533 | 1.094 (1.005-1.190) | 0.268 | 1.121 (1.042-1.207) | 0.034 |
| AIFM1 | 1.149 (1.065-1.239) | 0.006 | 1.228 (1.051-1.434) | 0.120 | 1.121 (1.027-1.222) | 0.115 | 1.225 (1.054-1.423) | 0.193 | 1.097 (1.006-1.196) | 0.266 | 1.151 (1.067-1.241) | 0.009 |
| ALDH3A1 | 1.119 (1.047-1.196) | 0.013 | 1.094 (0.952-1.259) | 0.458 | 1.118 (1.036-1.206) | 0.065 | 0.957 (0.822-1.115) | 0.827 | 1.118 (1.036-1.207) | 0.078 | 1.108 (1.037-1.185) | 0.035 |
| AMBP | 1.141 (1.054-1.236) | 0.015 | 1.179 (1.020-1.364) | 0.168 | 1.102 (1.002-1.212) | 0.281 | 0.894 (0.764-1.045) | 0.457 | 1.152 (1.052-1.262) | 0.054 | 1.103 (1.018-1.194) | 0.116 |
| AMIGO2 | 1.122 (1.042-1.208) | 0.025 | 1.291 (1.117-1.491) | 0.056 | 1.078 (0.989-1.175) | 0.407 | 1.096 (0.941-1.276) | 0.559 | 1.115 (1.025-1.213) | 0.133 | 1.127 (1.047-1.213) | 0.027 |
| ANGPT2 | 1.119 (1.038-1.206) | 0.035 | 1.184 (1.022-1.372) | 0.164 | 1.085 (0.994-1.185) | 0.356 | 0.992 (0.852-1.154) | 0.967 | 1.137 (1.043-1.239) | 0.070 | 1.089 (1.009-1.175) | 0.161 |
| ANXA10 | 1.129 (1.051-1.213) | 0.013 | 1.131 (0.982-1.304) | 0.283 | 1.119 (1.029-1.217) | 0.104 | 1.060 (0.922-1.218) | 0.712 | 1.097 (1.009-1.194) | 0.240 | 1.112 (1.035-1.195) | 0.047 |
| APBB1IP | 1.106 (1.031-1.187) | 0.043 | 1.155 (1.001-1.333) | 0.208 | 1.092 (1.008-1.184) | 0.231 | 1.120 (0.958-1.310) | 0.448 | 1.110 (1.026-1.202) | 0.123 | 1.110 (1.034-1.191) | 0.047 |
| APLP1 | 0.875 (0.807-0.949) | 0.017 | 0.801 (0.680-0.945) | 0.118 | 0.902 (0.822-0.990) | 0.223 | 0.897 (0.761-1.059) | 0.509 | 0.872 (0.796-0.957) | 0.073 | 0.870 (0.802-0.944) | 0.018 |
| APOM | 0.884 (0.820-0.953) | 0.017 | 0.999 (0.863-1.156) | 0.995 | 0.865 (0.791-0.947) | 0.038 | 0.942 (0.808-1.099) | 0.738 | 0.883 (0.811-0.962) | 0.079 | 0.909 (0.843-0.980) | 0.104 |
| AREG | 1.179 (1.103-1.262) | 1.54E-04 | 1.180 (1.025-1.359) | 0.156 | 1.175 (1.087-1.269) | 0.004 | 1.048 (0.910-1.207) | 0.783 | 1.157 (1.069-1.251) | 0.017 | 1.153 (1.076-1.235) | 0.004 |
| ARSB | 1.123 (1.043-1.208) | 0.023 | 1.275 (1.098-1.481) | 0.076 | 1.084 (0.996-1.180) | 0.335 | 1.253 (1.074-1.463) | 0.175 | 1.074 (0.987-1.169) | 0.457 | 1.129 (1.049-1.215) | 0.023 |
| ASAH1 | 1.112 (1.033-1.198) | 0.043 | 1.200 (1.039-1.387) | 0.140 | 1.087 (0.996-1.185) | 0.328 | 1.148 (0.972-1.355) | 0.376 | 1.092 (1.002-1.189) | 0.306 | 1.112 (1.033-1.198) | 0.055 |
| ASGR1 | 1.193 (1.103-1.291) | 0.001 | 1.153 (0.998-1.333) | 0.216 | 1.184 (1.077-1.303) | 0.018 | 1.003 (0.849-1.184) | 0.990 | 1.177 (1.076-1.288) | 0.020 | 1.159 (1.070-1.255) | 0.009 |
| ASGR2 | 1.130 (1.045-1.222) | 0.025 | 1.091 (0.939-1.266) | 0.519 | 1.143 (1.042-1.253) | 0.066 | 1.000 (0.841-1.190) | 0.999 | 1.126 (1.030-1.231) | 0.117 | 1.108 (1.025-1.198) | 0.086 |
| B2M | 1.111 (1.046-1.180) | 0.011 | 1.081 (0.930-1.256) | 0.576 | 1.116 (1.045-1.191) | 0.028 | 1.175 (0.997-1.386) | 0.284 | 1.091 (1.013-1.175) | 0.199 | 1.095 (1.027-1.168) | 0.060 |
| BAIAP2 | 1.169 (1.086-1.259) | 0.001 | 1.138 (0.984-1.317) | 0.268 | 1.167 (1.071-1.272) | 0.017 | 1.093 (0.949-1.258) | 0.530 | 1.125 (1.032-1.227) | 0.106 | 1.162 (1.079-1.251) | 0.004 |
| BAX | 1.121 (1.039-1.208) | 0.032 | 1.207 (1.035-1.407) | 0.143 | 1.094 (1.003-1.194) | 0.269 | 1.333 (1.135-1.566) | 0.122 | 1.058 (0.971-1.154) | 0.612 | 1.125 (1.043-1.213) | 0.033 |
| BCAN | 0.835 (0.770-0.904) | 0.001 | 0.816 (0.699-0.951) | 0.119 | 0.851 (0.775-0.934) | 0.024 | 0.931 (0.789-1.099) | 0.701 | 0.828 (0.757-0.907) | 0.006 | 0.836 (0.771-0.905) | 0.001 |
| BCAT1 | 1.117 (1.035-1.205) | 0.042 | 1.094 (0.930-1.287) | 0.546 | 1.123 (1.031-1.224) | 0.101 | 1.173 (1.028-1.337) | 0.215 | 1.063 (0.971-1.164) | 0.596 | 1.113 (1.032-1.201) | 0.060 |
| BST2 | 1.189 (1.115-1.267) | 3.06E-05 | 1.085 (0.936-1.258) | 0.546 | 1.210 (1.127-1.299) | 9.47E-05 | 1.100 (0.962-1.257) | 0.460 | 1.165 (1.080-1.256) | 0.007 | 1.179 (1.106-1.258) | 1.62E-04 |
| BTN3A2 | 1.187 (1.102-1.279) | 4.09E-04 | 1.228 (1.056-1.428) | 0.117 | 1.171 (1.075-1.277) | 0.014 | 1.072 (0.923-1.246) | 0.677 | 1.178 (1.081-1.283) | 0.014 | 1.165 (1.081-1.255) | 0.004 |
| C1RL | 1.172 (1.076-1.276) | 0.006 | 1.229 (1.033-1.462) | 0.152 | 1.161 (1.053-1.281) | 0.051 | 1.059 (0.889-1.263) | 0.787 | 1.157 (1.051-1.274) | 0.063 | 1.171 (1.076-1.275) | 0.008 |
| C7 | 1.140 (1.048-1.239) | 0.025 | 1.110 (0.941-1.310) | 0.469 | 1.140 (1.033-1.258) | 0.109 | 1.189 (1.008-1.402) | 0.256 | 1.102 (1.001-1.212) | 0.313 | 1.125 (1.034-1.224) | 0.065 |
| C9 | 1.181 (1.083-1.288) | 0.004 | 1.022 (0.880-1.187) | 0.899 | 1.268 (1.142-1.408) | 0.002 | 1.112 (0.937-1.318) | 0.538 | 1.194 (1.082-1.319) | 0.022 | 1.185 (1.087-1.292) | 0.006 |
| CA12 | 1.140 (1.061-1.226) | 0.007 | 1.192 (1.045-1.359) | 0.118 | 1.115 (1.023-1.216) | 0.134 | 1.092 (0.951-1.254) | 0.524 | 1.106 (1.017-1.203) | 0.196 | 1.116 (1.037-1.200) | 0.041 |
| CA14 | 0.871 (0.802-0.945) | 0.013 | 1.046 (0.898-1.219) | 0.778 | 0.834 (0.757-0.918) | 0.010 | 1.221 (1.032-1.445) | 0.217 | 0.836 (0.762-0.918) | 0.014 | 0.864 (0.797-0.938) | 0.012 |
| CA6 | 0.812 (0.750-0.878) | 4.15E-05 | 0.764 (0.650-0.897) | 0.067 | 0.834 (0.761-0.913) | 0.006 | 0.894 (0.757-1.056) | 0.493 | 0.819 (0.749-0.896) | 0.002 | 0.817 (0.755-0.885) | 1.62E-04 |
| CALCA | 1.136 (1.039-1.242) | 0.044 | 1.120 (0.936-1.339) | 0.472 | 1.139 (1.027-1.263) | 0.134 | 1.165 (0.997-1.360) | 0.284 | 1.086 (0.979-1.205) | 0.507 | 1.109 (1.014-1.214) | 0.148 |
| CANT1 | 1.118 (1.036-1.208) | 0.040 | 1.192 (1.027-1.383) | 0.153 | 1.094 (1.000-1.197) | 0.296 | 1.088 (0.930-1.272) | 0.613 | 1.084 (0.992-1.183) | 0.389 | 1.107 (1.025-1.195) | 0.084 |
| CBLN4 | 0.891 (0.827-0.960) | 0.025 | 0.936 (0.811-1.081) | 0.629 | 0.871 (0.798-0.949) | 0.039 | 0.897 (0.771-1.044) | 0.456 | 0.908 (0.834-0.988) | 0.225 | 0.896 (0.832-0.965) | 0.045 |
| CCL15 | 1.120 (1.043-1.203) | 0.021 | 1.167 (1.022-1.332) | 0.158 | 1.092 (1.003-1.189) | 0.268 | 1.168 (1.011-1.350) | 0.246 | 1.094 (1.008-1.188) | 0.246 | 1.090 (1.014-1.171) | 0.127 |
| CCL3 | 1.084 (1.026-1.145) | 0.038 | 1.066 (0.962-1.181) | 0.483 | 1.087 (1.019-1.160) | 0.125 | 1.127 (1.016-1.251) | 0.217 | 1.047 (0.978-1.120) | 0.601 | 1.077 (1.018-1.139) | 0.084 |
| CCL5 | 1.120 (1.040-1.205) | 0.028 | 1.248 (1.081-1.441) | 0.094 | 1.077 (0.989-1.174) | 0.407 | 1.162 (0.994-1.358) | 0.290 | 1.088 (1.000-1.183) | 0.315 | 1.122 (1.043-1.208) | 0.032 |
| CCL7 | 1.162 (1.087-1.242) | 0.001 | 1.075 (0.920-1.255) | 0.625 | 1.186 (1.102-1.276) | 0.001 | 1.102 (0.973-1.249) | 0.404 | 1.140 (1.054-1.233) | 0.036 | 1.157 (1.082-1.238) | 0.002 |
| CD163 | 1.119 (1.038-1.207) | 0.035 | 1.138 (0.975-1.329) | 0.309 | 1.113 (1.020-1.214) | 0.150 | 1.054 (0.898-1.238) | 0.787 | 1.088 (0.998-1.187) | 0.337 | 1.112 (1.031-1.199) | 0.061 |
| CD27 | 1.125 (1.044-1.212) | 0.023 | 1.137 (0.998-1.295) | 0.217 | 1.115 (1.019-1.220) | 0.161 | 1.131 (0.974-1.313) | 0.376 | 1.091 (1.001-1.190) | 0.310 | 1.086 (1.007-1.170) | 0.170 |
| CD274 | 1.150 (1.073-1.233) | 0.003 | 1.188 (1.046-1.348) | 0.118 | 1.147 (1.056-1.245) | 0.029 | 1.171 (1.022-1.342) | 0.217 | 1.111 (1.024-1.205) | 0.139 | 1.137 (1.060-1.219) | 0.010 |
| CD276 | 1.176 (1.089-1.269) | 0.001 | 1.182 (1.033-1.352) | 0.143 | 1.158 (1.056-1.270) | 0.039 | 1.071 (0.916-1.254) | 0.697 | 1.163 (1.066-1.269) | 0.027 | 1.154 (1.070-1.246) | 0.008 |
| CD300E | 1.186 (1.097-1.283) | 0.001 | 1.168 (1.014-1.347) | 0.177 | 1.196 (1.090-1.313) | 0.009 | 1.094 (0.939-1.276) | 0.566 | 1.169 (1.069-1.279) | 0.027 | 1.155 (1.068-1.249) | 0.009 |
| CD302 | 1.193 (1.108-1.283) | 2.06E-04 | 1.191 (1.047-1.355) | 0.118 | 1.192 (1.091-1.303) | 0.007 | 1.029 (0.891-1.188) | 0.886 | 1.162 (1.065-1.267) | 0.027 | 1.154 (1.070-1.243) | 0.008 |
| CD34 | 0.897 (0.832-0.967) | 0.042 | 0.955 (0.824-1.106) | 0.761 | 0.892 (0.818-0.974) | 0.119 | 0.959 (0.823-1.117) | 0.837 | 0.880 (0.808-0.959) | 0.070 | 0.901 (0.836-0.972) | 0.067 |
| CD36 | 1.123 (1.037-1.217) | 0.042 | 1.299 (1.106-1.526) | 0.076 | 1.071 (0.976-1.175) | 0.520 | 1.215 (1.027-1.436) | 0.217 | 1.081 (0.987-1.185) | 0.444 | 1.122 (1.036-1.216) | 0.055 |
| CD4 | 1.111 (1.048-1.178) | 0.008 | 1.154 (1.006-1.324) | 0.194 | 1.102 (1.029-1.179) | 0.074 | 1.106 (0.989-1.238) | 0.328 | 1.083 (1.007-1.166) | 0.250 | 1.098 (1.032-1.168) | 0.039 |
| CD5 | 1.111 (1.036-1.192) | 0.033 | 1.106 (0.957-1.277) | 0.412 | 1.106 (1.019-1.199) | 0.147 | 1.059 (0.914-1.228) | 0.735 | 1.097 (1.010-1.190) | 0.234 | 1.097 (1.021-1.179) | 0.097 |
| CD72 | 1.154 (1.061-1.256) | 0.013 | 1.156 (0.984-1.357) | 0.260 | 1.151 (1.043-1.270) | 0.075 | 1.135 (0.961-1.340) | 0.421 | 1.134 (1.030-1.249) | 0.131 | 1.140 (1.049-1.240) | 0.033 |
| CD74 | 1.155 (1.069-1.248) | 0.006 | 1.227 (1.056-1.425) | 0.117 | 1.131 (1.033-1.237) | 0.096 | 1.036 (0.884-1.214) | 0.874 | 1.149 (1.052-1.254) | 0.054 | 1.127 (1.043-1.219) | 0.035 |
| CD79B | 1.141 (1.061-1.228) | 0.008 | 1.205 (1.058-1.372) | 0.116 | 1.117 (1.023-1.220) | 0.133 | 1.075 (0.928-1.245) | 0.651 | 1.128 (1.037-1.227) | 0.084 | 1.113 (1.034-1.198) | 0.053 |
| CD80 | 1.187 (1.097-1.284) | 0.001 | 1.265 (1.085-1.475) | 0.098 | 1.162 (1.060-1.274) | 0.033 | 1.176 (1.002-1.380) | 0.269 | 1.149 (1.048-1.259) | 0.063 | 1.173 (1.084-1.270) | 0.004 |
| CD83 | 1.173 (1.088-1.264) | 0.001 | 1.204 (1.055-1.375) | 0.117 | 1.150 (1.051-1.258) | 0.046 | 1.100 (0.959-1.262) | 0.478 | 1.129 (1.034-1.232) | 0.101 | 1.147 (1.064-1.236) | 0.010 |
| CDCP1 | 1.226 (1.134-1.326) | 5.48E-05 | 1.232 (1.057-1.436) | 0.117 | 1.219 (1.113-1.335) | 0.003 | 1.096 (0.939-1.280) | 0.563 | 1.203 (1.099-1.316) | 0.007 | 1.211 (1.120-1.310) | 3.59E-04 |
| CDH2 | 1.121 (1.034-1.215) | 0.049 | 0.995 (0.854-1.159) | 0.978 | 1.155 (1.051-1.269) | 0.051 | 0.961 (0.827-1.117) | 0.844 | 1.155 (1.053-1.267) | 0.054 | 1.108 (1.022-1.202) | 0.102 |
| CEACAM1 | 1.133 (1.049-1.224) | 0.019 | 1.254 (1.069-1.470) | 0.117 | 1.097 (1.004-1.199) | 0.264 | 0.990 (0.856-1.144) | 0.960 | 1.130 (1.035-1.234) | 0.098 | 1.138 (1.053-1.229) | 0.023 |
| CEACAM5 | 1.158 (1.074-1.249) | 0.004 | 1.191 (1.025-1.384) | 0.158 | 1.143 (1.048-1.248) | 0.049 | 1.134 (0.989-1.301) | 0.314 | 1.132 (1.037-1.236) | 0.092 | 1.148 (1.064-1.238) | 0.010 |
| CEACAM6 | 1.170 (1.080-1.268) | 0.003 | 1.226 (1.039-1.446) | 0.143 | 1.153 (1.051-1.265) | 0.048 | 1.184 (1.012-1.385) | 0.246 | 1.156 (1.054-1.268) | 0.054 | 1.168 (1.078-1.266) | 0.007 |
| CEACAM8 | 1.131 (1.053-1.215) | 0.012 | 1.225 (1.058-1.419) | 0.117 | 1.100 (1.013-1.195) | 0.191 | 1.152 (0.987-1.345) | 0.317 | 1.128 (1.040-1.224) | 0.073 | 1.119 (1.041-1.203) | 0.033 |
| CFB | 1.137 (1.040-1.244) | 0.043 | 1.128 (0.939-1.354) | 0.447 | 1.121 (1.012-1.243) | 0.221 | 0.971 (0.811-1.162) | 0.907 | 1.183 (1.068-1.310) | 0.037 | 1.139 (1.041-1.245) | 0.053 |
| CFH | 1.198 (1.088-1.318) | 0.005 | 1.169 (0.959-1.424) | 0.344 | 1.193 (1.068-1.332) | 0.039 | 0.911 (0.741-1.121) | 0.689 | 1.273 (1.144-1.418) | 0.002 | 1.204 (1.094-1.325) | 0.006 |
| CFHR5 | 1.163 (1.068-1.267) | 0.009 | 1.148 (0.970-1.358) | 0.322 | 1.182 (1.070-1.306) | 0.029 | 0.986 (0.832-1.169) | 0.951 | 1.171 (1.062-1.291) | 0.044 | 1.164 (1.068-1.268) | 0.014 |
| CHCHD10 | 1.156 (1.064-1.256) | 0.010 | 1.145 (0.985-1.331) | 0.262 | 1.151 (1.042-1.272) | 0.078 | 1.196 (1.006-1.423) | 0.260 | 1.101 (0.998-1.215) | 0.337 | 1.128 (1.038-1.226) | 0.054 |
| CHGA | 1.193 (1.113-1.279) | 7.91E-05 | 1.152 (1.022-1.299) | 0.153 | 1.201 (1.102-1.308) | 0.003 | 1.060 (0.934-1.204) | 0.677 | 1.166 (1.073-1.268) | 0.018 | 1.164 (1.085-1.250) | 0.002 |
| CHI3L1 | 1.184 (1.099-1.275) | 4.78E-04 | 1.152 (1.003-1.324) | 0.203 | 1.184 (1.085-1.292) | 0.009 | 1.114 (0.965-1.286) | 0.430 | 1.160 (1.065-1.263) | 0.026 | 1.167 (1.083-1.257) | 0.004 |
| CKAP4 | 1.138 (1.059-1.222) | 0.008 | 1.192 (1.056-1.346) | 0.112 | 1.119 (1.025-1.222) | 0.128 | 1.069 (0.923-1.239) | 0.682 | 1.124 (1.035-1.221) | 0.090 | 1.105 (1.028-1.189) | 0.069 |
| CLEC4D | 1.240 (1.152-1.335) | 5.51E-06 | 1.314 (1.136-1.519) | 0.046 | 1.207 (1.108-1.315) | 0.003 | 1.167 (0.997-1.367) | 0.284 | 1.256 (1.155-1.366) | 4.92E-05 | 1.222 (1.135-1.316) | 8.33E-05 |
| CLEC5A | 1.113 (1.033-1.199) | 0.043 | 1.211 (1.053-1.392) | 0.117 | 1.078 (0.987-1.177) | 0.415 | 1.073 (0.924-1.246) | 0.671 | 1.108 (1.018-1.207) | 0.189 | 1.099 (1.020-1.184) | 0.104 |
| CLEC6A | 1.126 (1.045-1.213) | 0.021 | 1.176 (1.014-1.363) | 0.178 | 1.103 (1.012-1.203) | 0.208 | 1.085 (0.929-1.266) | 0.623 | 1.118 (1.027-1.217) | 0.130 | 1.113 (1.033-1.199) | 0.055 |
| CLEC7A | 1.135 (1.042-1.236) | 0.035 | 1.241 (1.052-1.463) | 0.123 | 1.095 (0.994-1.206) | 0.349 | 1.106 (0.931-1.314) | 0.566 | 1.127 (1.024-1.241) | 0.164 | 1.110 (1.022-1.206) | 0.104 |
| CNTN1 | 0.872 (0.805-0.945) | 0.012 | 0.953 (0.816-1.114) | 0.765 | 0.844 (0.769-0.926) | 0.014 | 1.015 (0.865-1.189) | 0.946 | 0.851 (0.777-0.932) | 0.024 | 0.874 (0.807-0.946) | 0.020 |
| CNTN5 | 0.851 (0.784-0.925) | 0.004 | 0.797 (0.675-0.941) | 0.117 | 0.883 (0.803-0.972) | 0.119 | 0.910 (0.774-1.069) | 0.566 | 0.833 (0.757-0.915) | 0.013 | 0.866 (0.797-0.940) | 0.015 |
| COL18A1 | 1.132 (1.044-1.226) | 0.027 | 1.096 (0.949-1.266) | 0.469 | 1.125 (1.022-1.239) | 0.151 | 0.974 (0.828-1.147) | 0.907 | 1.153 (1.052-1.263) | 0.054 | 1.093 (1.009-1.184) | 0.167 |
| COL4A1 | 1.145 (1.064-1.231) | 0.006 | 1.092 (0.945-1.262) | 0.496 | 1.163 (1.068-1.265) | 0.018 | 1.088 (0.957-1.236) | 0.509 | 1.108 (1.017-1.207) | 0.194 | 1.128 (1.049-1.213) | 0.023 |
| COL5A1 | 1.129 (1.045-1.221) | 0.025 | 1.060 (0.908-1.237) | 0.700 | 1.151 (1.051-1.261) | 0.046 | 1.042 (0.899-1.208) | 0.834 | 1.140 (1.043-1.247) | 0.077 | 1.118 (1.034-1.209) | 0.055 |
| COL6A3 | 1.151 (1.064-1.244) | 0.008 | 1.179 (1.035-1.343) | 0.141 | 1.120 (1.017-1.234) | 0.180 | 1.051 (0.907-1.218) | 0.780 | 1.140 (1.041-1.249) | 0.081 | 1.102 (1.018-1.194) | 0.116 |
| CPM | 1.162 (1.072-1.260) | 0.006 | 1.190 (1.017-1.393) | 0.173 | 1.127 (1.025-1.239) | 0.132 | 0.861 (0.736-1.007) | 0.291 | 1.170 (1.066-1.285) | 0.033 | 1.148 (1.059-1.245) | 0.019 |
| CRYBB2 | 1.092 (1.028-1.160) | 0.040 | 1.100 (0.989-1.224) | 0.262 | 1.082 (1.004-1.166) | 0.261 | 1.150 (1.027-1.287) | 0.214 | 1.066 (0.991-1.148) | 0.423 | 1.087 (1.023-1.156) | 0.068 |
| CSF1 | 1.171 (1.086-1.261) | 0.001 | 1.153 (0.993-1.338) | 0.231 | 1.184 (1.086-1.291) | 0.008 | 1.053 (0.908-1.220) | 0.769 | 1.155 (1.060-1.259) | 0.034 | 1.144 (1.061-1.234) | 0.012 |
| CST3 | 1.126 (1.039-1.221) | 0.038 | 1.183 (1.034-1.353) | 0.142 | 1.083 (0.980-1.196) | 0.466 | 1.045 (0.889-1.229) | 0.837 | 1.132 (1.032-1.241) | 0.116 | 1.081 (0.996-1.173) | 0.244 |
| CTSL | 1.172 (1.086-1.264) | 0.001 | 1.232 (1.064-1.426) | 0.116 | 1.154 (1.056-1.261) | 0.036 | 1.073 (0.925-1.245) | 0.665 | 1.136 (1.041-1.240) | 0.078 | 1.151 (1.067-1.242) | 0.009 |
| CTSO | 1.134 (1.054-1.220) | 0.012 | 1.067 (0.926-1.231) | 0.629 | 1.143 (1.048-1.246) | 0.047 | 0.999 (0.863-1.157) | 0.998 | 1.137 (1.045-1.236) | 0.061 | 1.113 (1.033-1.198) | 0.055 |
| CTSZ | 1.147 (1.063-1.238) | 0.008 | 1.151 (0.995-1.331) | 0.225 | 1.152 (1.054-1.260) | 0.041 | 1.022 (0.876-1.193) | 0.914 | 1.132 (1.037-1.235) | 0.088 | 1.128 (1.046-1.218) | 0.031 |
| CXADR | 1.161 (1.082-1.245) | 0.001 | 1.131 (0.974-1.312) | 0.317 | 1.163 (1.074-1.260) | 0.010 | 1.109 (0.957-1.285) | 0.473 | 1.106 (1.019-1.201) | 0.172 | 1.145 (1.066-1.229) | 0.008 |
| CXCL11 | 1.116 (1.039-1.200) | 0.029 | 1.175 (1.014-1.361) | 0.178 | 1.105 (1.017-1.201) | 0.166 | 1.079 (0.929-1.254) | 0.636 | 1.108 (1.021-1.203) | 0.163 | 1.116 (1.038-1.199) | 0.037 |
| CXCL8 | 1.112 (1.037-1.191) | 0.030 | 0.999 (0.864-1.155) | 0.996 | 1.145 (1.058-1.239) | 0.026 | 1.141 (0.999-1.304) | 0.281 | 1.068 (0.983-1.160) | 0.507 | 1.105 (1.031-1.184) | 0.055 |
| CXCL9 | 1.187 (1.103-1.278) | 3.58E-04 | 1.118 (0.961-1.300) | 0.379 | 1.202 (1.105-1.308) | 0.003 | 1.075 (0.919-1.259) | 0.677 | 1.172 (1.077-1.275) | 0.015 | 1.164 (1.081-1.254) | 0.004 |
| DBI | 1.118 (1.035-1.207) | 0.042 | 1.243 (1.057-1.463) | 0.118 | 1.080 (0.989-1.179) | 0.402 | 1.168 (0.998-1.367) | 0.283 | 1.085 (0.994-1.184) | 0.377 | 1.116 (1.033-1.205) | 0.058 |
| DCBLD2 | 1.116 (1.034-1.204) | 0.042 | 1.004 (0.864-1.167) | 0.982 | 1.114 (1.016-1.223) | 0.183 | 0.979 (0.838-1.144) | 0.915 | 1.089 (0.998-1.188) | 0.337 | 1.082 (1.002-1.168) | 0.201 |
| DDAH1 | 1.161 (1.079-1.249) | 0.002 | 1.178 (1.019-1.363) | 0.170 | 1.145 (1.052-1.246) | 0.039 | 1.201 (1.043-1.384) | 0.206 | 1.104 (1.014-1.203) | 0.210 | 1.155 (1.073-1.243) | 0.006 |
| DEFB4A_DEFB4B | 1.166 (1.078-1.261) | 0.003 | 1.209 (1.036-1.412) | 0.143 | 1.157 (1.056-1.267) | 0.039 | 1.263 (1.067-1.494) | 0.191 | 1.102 (1.007-1.206) | 0.257 | 1.164 (1.076-1.259) | 0.006 |
| DLK1 | 0.890 (0.823-0.961) | 0.032 | 1.013 (0.874-1.175) | 0.940 | 0.845 (0.772-0.925) | 0.012 | 1.005 (0.865-1.166) | 0.984 | 0.857 (0.784-0.936) | 0.026 | 0.883 (0.818-0.954) | 0.028 |
| DLL1 | 1.136 (1.053-1.225) | 0.014 | 1.178 (1.034-1.342) | 0.141 | 1.108 (1.012-1.214) | 0.211 | 1.043 (0.891-1.222) | 0.843 | 1.115 (1.022-1.216) | 0.160 | 1.105 (1.024-1.192) | 0.086 |
| DPP7 | 1.178 (1.093-1.269) | 0.001 | 1.222 (1.053-1.417) | 0.118 | 1.171 (1.074-1.277) | 0.015 | 1.130 (0.975-1.310) | 0.376 | 1.154 (1.060-1.257) | 0.034 | 1.176 (1.092-1.266) | 0.002 |
| DPY30 | 1.134 (1.054-1.221) | 0.012 | 1.337 (1.149-1.556) | 0.046 | 1.078 (0.990-1.175) | 0.401 | 1.259 (1.093-1.451) | 0.154 | 1.063 (0.974-1.160) | 0.586 | 1.126 (1.046-1.213) | 0.029 |
| DSC2 | 1.119 (1.037-1.207) | 0.036 | 1.163 (1.015-1.334) | 0.173 | 1.093 (0.999-1.196) | 0.307 | 1.103 (0.949-1.281) | 0.510 | 1.085 (0.994-1.184) | 0.370 | 1.087 (1.008-1.174) | 0.170 |
| ECHDC3 | 1.168 (1.083-1.260) | 0.002 | 1.060 (0.903-1.245) | 0.712 | 1.188 (1.089-1.295) | 0.007 | 1.173 (1.012-1.360) | 0.243 | 1.142 (1.046-1.247) | 0.064 | 1.163 (1.078-1.254) | 0.005 |
| ECHS1 | 1.131 (1.040-1.230) | 0.039 | 1.152 (0.966-1.375) | 0.334 | 1.122 (1.020-1.235) | 0.165 | 1.213 (1.029-1.430) | 0.217 | 1.097 (0.997-1.207) | 0.344 | 1.129 (1.038-1.228) | 0.054 |
| EDN1 | 1.140 (1.050-1.238) | 0.022 | 1.158 (0.987-1.360) | 0.252 | 1.122 (1.018-1.236) | 0.170 | 1.197 (1.024-1.400) | 0.217 | 1.082 (0.983-1.190) | 0.489 | 1.113 (1.024-1.208) | 0.094 |
| EFNA1 | 1.114 (1.033-1.202) | 0.046 | 1.189 (1.049-1.347) | 0.117 | 1.074 (0.978-1.178) | 0.497 | 1.078 (0.931-1.249) | 0.636 | 1.088 (0.996-1.188) | 0.355 | 1.073 (0.995-1.158) | 0.254 |
| EFNA4 | 1.206 (1.121-1.297) | 7.18E-05 | 1.231 (1.090-1.391) | 0.066 | 1.179 (1.077-1.291) | 0.015 | 1.069 (0.918-1.245) | 0.700 | 1.198 (1.101-1.303) | 0.004 | 1.159 (1.075-1.249) | 0.006 |
| EGFL7 | 1.120 (1.038-1.208) | 0.034 | 1.164 (1.012-1.338) | 0.179 | 1.096 (1.003-1.197) | 0.272 | 1.032 (0.872-1.222) | 0.894 | 1.128 (1.036-1.229) | 0.092 | 1.109 (1.028-1.196) | 0.074 |
| EGFR | 0.834 (0.776-0.896) | 8.42E-05 | 0.932 (0.809-1.074) | 0.591 | 0.807 (0.743-0.877) | 2.16E-04 | 0.907 (0.777-1.058) | 0.525 | 0.835 (0.770-0.906) | 0.003 | 0.852 (0.792-0.916) | 0.002 |
| ELN | 1.190 (1.073-1.321) | 0.014 | 1.199 (0.988-1.454) | 0.239 | 1.188 (1.051-1.342) | 0.080 | 1.267 (1.043-1.540) | 0.215 | 1.115 (0.988-1.258) | 0.399 | 1.168 (1.053-1.296) | 0.042 |
| EPHA2 | 1.164 (1.081-1.252) | 0.002 | 1.158 (1.026-1.307) | 0.144 | 1.165 (1.064-1.276) | 0.029 | 1.129 (0.973-1.309) | 0.383 | 1.118 (1.025-1.219) | 0.139 | 1.125 (1.044-1.213) | 0.032 |
| EPPK1 | 1.131 (1.049-1.220) | 0.017 | 1.144 (0.975-1.341) | 0.303 | 1.124 (1.031-1.226) | 0.098 | 1.201 (1.031-1.399) | 0.217 | 1.080 (0.989-1.178) | 0.423 | 1.128 (1.046-1.217) | 0.030 |
| ESAM | 1.108 (1.030-1.191) | 0.049 | 1.225 (1.082-1.387) | 0.076 | 1.059 (0.971-1.156) | 0.585 | 1.207 (1.045-1.394) | 0.205 | 1.052 (0.967-1.144) | 0.650 | 1.089 (1.013-1.170) | 0.138 |
| FABP1 | 1.163 (1.077-1.257) | 0.004 | 1.098 (0.941-1.283) | 0.498 | 1.148 (1.047-1.259) | 0.055 | 1.084 (0.926-1.269) | 0.636 | 1.158 (1.060-1.265) | 0.037 | 1.127 (1.042-1.218) | 0.038 |
| FAM3C | 1.124 (1.041-1.214) | 0.030 | 1.193 (1.049-1.357) | 0.117 | 1.072 (0.976-1.178) | 0.525 | 1.125 (0.966-1.310) | 0.409 | 1.100 (1.007-1.202) | 0.258 | 1.087 (1.006-1.174) | 0.176 |
| FAP | 0.879 (0.815-0.948) | 0.012 | 0.917 (0.794-1.060) | 0.502 | 0.869 (0.796-0.949) | 0.040 | 0.890 (0.768-1.032) | 0.398 | 0.889 (0.816-0.969) | 0.105 | 0.890 (0.826-0.960) | 0.035 |
| FAS | 1.101 (1.031-1.175) | 0.037 | 1.021 (0.891-1.171) | 0.896 | 1.133 (1.051-1.220) | 0.029 | 1.095 (0.938-1.280) | 0.566 | 1.094 (1.015-1.179) | 0.189 | 1.085 (1.015-1.159) | 0.116 |
| FCN1 | 1.138 (1.040-1.245) | 0.043 | 1.134 (0.944-1.363) | 0.421 | 1.129 (1.019-1.252) | 0.173 | 1.057 (0.874-1.279) | 0.825 | 1.154 (1.042-1.278) | 0.096 | 1.131 (1.034-1.237) | 0.068 |
| FCRLB | 1.123 (1.049-1.202) | 0.013 | 1.040 (0.899-1.202) | 0.796 | 1.150 (1.064-1.243) | 0.017 | 1.007 (0.851-1.190) | 0.981 | 1.144 (1.061-1.233) | 0.022 | 1.115 (1.041-1.195) | 0.031 |
| FETUB | 1.138 (1.049-1.234) | 0.022 | 1.365 (1.146-1.626) | 0.055 | 1.082 (0.987-1.187) | 0.413 | 0.925 (0.795-1.075) | 0.631 | 1.148 (1.046-1.260) | 0.073 | 1.137 (1.048-1.233) | 0.031 |
| FGF12 | 1.156 (1.061-1.261) | 0.014 | 1.111 (0.934-1.321) | 0.496 | 1.159 (1.049-1.281) | 0.059 | 1.150 (0.949-1.393) | 0.447 | 1.166 (1.057-1.285) | 0.054 | 1.153 (1.058-1.257) | 0.023 |
| FGFBP3 | 0.870 (0.802-0.943) | 0.012 | 0.926 (0.790-1.086) | 0.607 | 0.855 (0.778-0.940) | 0.029 | 0.919 (0.785-1.076) | 0.615 | 0.866 (0.789-0.950) | 0.055 | 0.876 (0.808-0.950) | 0.027 |
| FGFR4 | 1.148 (1.064-1.239) | 0.007 | 1.182 (1.024-1.364) | 0.158 | 1.118 (1.022-1.224) | 0.145 | 1.031 (0.887-1.199) | 0.886 | 1.126 (1.031-1.230) | 0.112 | 1.126 (1.043-1.215) | 0.035 |
| FGR | 1.106 (1.033-1.183) | 0.037 | 1.221 (1.070-1.394) | 0.100 | 1.074 (0.991-1.164) | 0.398 | 1.165 (1.025-1.324) | 0.217 | 1.086 (1.003-1.176) | 0.294 | 1.106 (1.032-1.184) | 0.049 |
| FN1 | 1.248 (1.144-1.361) | 7.91E-05 | 1.087 (0.919-1.286) | 0.591 | 1.289 (1.165-1.427) | 3.69E-04 | 1.096 (0.909-1.320) | 0.654 | 1.253 (1.136-1.382) | 0.002 | 1.232 (1.129-1.344) | 4.54E-04 |
| FOXO3 | 1.142 (1.062-1.229) | 0.007 | 1.253 (1.088-1.442) | 0.081 | 1.110 (1.019-1.208) | 0.151 | 1.212 (1.051-1.398) | 0.193 | 1.103 (1.014-1.201) | 0.211 | 1.148 (1.067-1.234) | 0.008 |
| FSTL1 | 1.149 (1.065-1.238) | 0.006 | 1.117 (0.947-1.318) | 0.437 | 1.158 (1.065-1.258) | 0.020 | 1.305 (1.098-1.551) | 0.154 | 1.104 (1.011-1.207) | 0.235 | 1.135 (1.052-1.226) | 0.023 |
| FSTL3 | 1.188 (1.096-1.287) | 0.001 | 1.164 (1.014-1.336) | 0.174 | 1.189 (1.078-1.311) | 0.019 | 1.133 (0.969-1.324) | 0.391 | 1.162 (1.059-1.275) | 0.044 | 1.142 (1.053-1.238) | 0.026 |
| GAST | 1.216 (1.131-1.306) | 2.64E-05 | 1.213 (1.067-1.380) | 0.100 | 1.204 (1.103-1.314) | 0.003 | 1.124 (0.977-1.294) | 0.373 | 1.169 (1.073-1.273) | 0.020 | 1.191 (1.108-1.282) | 4.54E-04 |
| GCNT1 | 1.132 (1.050-1.220) | 0.016 | 1.138 (0.976-1.327) | 0.306 | 1.125 (1.032-1.226) | 0.096 | 1.064 (0.908-1.246) | 0.732 | 1.119 (1.027-1.219) | 0.130 | 1.125 (1.043-1.213) | 0.034 |
| GDF15 | 1.261 (1.187-1.340) | 1.37E-10 | 1.203 (1.056-1.371) | 0.117 | 1.257 (1.171-1.349) | 7.61E-07 | 1.150 (0.989-1.337) | 0.311 | 1.229 (1.142-1.323) | 2.36E-05 | 1.229 (1.152-1.312) | 7.26E-07 |
| GFER | 1.129 (1.046-1.218) | 0.021 | 1.219 (1.040-1.428) | 0.142 | 1.102 (1.010-1.201) | 0.220 | 1.305 (1.119-1.523) | 0.136 | 1.059 (0.970-1.156) | 0.611 | 1.131 (1.048-1.221) | 0.027 |
| GFRA1 | 1.198 (1.108-1.295) | 3.85E-04 | 1.184 (1.032-1.358) | 0.143 | 1.187 (1.081-1.305) | 0.015 | 1.138 (0.976-1.326) | 0.365 | 1.170 (1.070-1.280) | 0.026 | 1.159 (1.072-1.253) | 0.008 |
| GGH | 1.147 (1.061-1.240) | 0.010 | 1.190 (1.011-1.400) | 0.185 | 1.126 (1.029-1.232) | 0.114 | 1.060 (0.895-1.256) | 0.773 | 1.140 (1.044-1.246) | 0.073 | 1.143 (1.057-1.236) | 0.018 |
| GGT1 | 1.132 (1.046-1.225) | 0.023 | 1.087 (0.929-1.271) | 0.567 | 1.132 (1.032-1.241) | 0.103 | 0.963 (0.830-1.117) | 0.853 | 1.116 (1.018-1.224) | 0.196 | 1.128 (1.042-1.221) | 0.039 |
| GIPC2 | 1.150 (1.068-1.239) | 0.005 | 1.116 (0.964-1.292) | 0.372 | 1.155 (1.059-1.260) | 0.029 | 1.177 (1.004-1.380) | 0.263 | 1.122 (1.029-1.222) | 0.117 | 1.133 (1.052-1.221) | 0.021 |
| GKN1 | 1.146 (1.070-1.228) | 0.003 | 1.176 (1.027-1.347) | 0.148 | 1.127 (1.040-1.221) | 0.058 | 1.119 (0.993-1.261) | 0.307 | 1.117 (1.030-1.211) | 0.105 | 1.139 (1.063-1.220) | 0.008 |
| GOLM2 | 1.107 (1.032-1.188) | 0.042 | 1.023 (0.886-1.180) | 0.894 | 1.129 (1.038-1.228) | 0.066 | 1.042 (0.901-1.205) | 0.828 | 1.091 (1.006-1.183) | 0.262 | 1.099 (1.023-1.180) | 0.086 |
| GPKOW | 1.111 (1.049-1.176) | 0.006 | 1.177 (1.076-1.287) | 0.046 | 1.083 (1.009-1.163) | 0.208 | 1.131 (1.023-1.251) | 0.214 | 1.081 (1.005-1.162) | 0.262 | 1.109 (1.048-1.174) | 0.011 |
| GPNMB | 1.128 (1.045-1.219) | 0.024 | 1.200 (1.032-1.394) | 0.144 | 1.102 (1.008-1.205) | 0.236 | 1.038 (0.881-1.221) | 0.874 | 1.132 (1.037-1.236) | 0.089 | 1.113 (1.030-1.202) | 0.067 |
| GRN | 1.118 (1.037-1.205) | 0.035 | 1.165 (1.003-1.354) | 0.203 | 1.087 (0.996-1.186) | 0.329 | 1.024 (0.886-1.184) | 0.906 | 1.107 (1.016-1.206) | 0.200 | 1.095 (1.016-1.180) | 0.122 |
| GRPEL1 | 1.205 (1.123-1.293) | 4.15E-05 | 1.212 (1.045-1.405) | 0.126 | 1.194 (1.101-1.294) | 0.003 | 1.143 (0.989-1.321) | 0.313 | 1.169 (1.077-1.268) | 0.014 | 1.200 (1.118-1.288) | 1.62E-04 |
| GSN | 0.915 (0.869-0.963) | 0.012 | 0.975 (0.835-1.138) | 0.888 | 0.912 (0.864-0.963) | 0.026 | 0.929 (0.782-1.103) | 0.701 | 0.922 (0.867-0.982) | 0.133 | 0.913 (0.866-0.962) | 0.016 |
| GUSB | 1.121 (1.034-1.215) | 0.049 | 1.207 (1.044-1.395) | 0.126 | 1.101 (1.000-1.213) | 0.299 | 0.959 (0.827-1.111) | 0.827 | 1.130 (1.029-1.242) | 0.131 | 1.120 (1.033-1.214) | 0.061 |
| HAVCR1 | 1.207 (1.118-1.304) | 1.54E-04 | 1.215 (1.063-1.389) | 0.112 | 1.177 (1.072-1.294) | 0.023 | 1.017 (0.870-1.189) | 0.930 | 1.212 (1.109-1.325) | 0.003 | 1.173 (1.086-1.267) | 0.004 |
| HAVCR2 | 1.165 (1.080-1.257) | 0.003 | 1.164 (1.009-1.342) | 0.185 | 1.158 (1.059-1.267) | 0.033 | 1.110 (0.957-1.289) | 0.471 | 1.122 (1.027-1.227) | 0.131 | 1.131 (1.047-1.221) | 0.030 |
| HDGF | 1.138 (1.055-1.228) | 0.013 | 1.153 (0.985-1.351) | 0.259 | 1.136 (1.041-1.238) | 0.063 | 1.184 (1.005-1.394) | 0.261 | 1.137 (1.043-1.239) | 0.071 | 1.143 (1.059-1.233) | 0.015 |
| HEG1 | 1.130 (1.037-1.232) | 0.048 | 1.083 (0.910-1.290) | 0.628 | 1.132 (1.025-1.251) | 0.140 | 1.132 (0.959-1.337) | 0.430 | 1.111 (1.007-1.226) | 0.266 | 1.128 (1.034-1.230) | 0.065 |
| HEPACAM2 | 0.891 (0.821-0.967) | 0.049 | 0.858 (0.731-1.008) | 0.234 | 0.917 (0.834-1.009) | 0.377 | 0.961 (0.809-1.142) | 0.873 | 0.882 (0.804-0.967) | 0.106 | 0.896 (0.826-0.972) | 0.078 |
| HGF | 1.251 (1.156-1.354) | 9.04E-06 | 1.206 (1.035-1.406) | 0.143 | 1.254 (1.143-1.376) | 0.001 | 1.030 (0.882-1.203) | 0.891 | 1.301 (1.189-1.424) | 1.16E-05 | 1.234 (1.141-1.336) | 9.34E-05 |
| HHEX | 1.128 (1.038-1.226) | 0.043 | 1.338 (1.126-1.589) | 0.066 | 1.075 (0.976-1.184) | 0.510 | 1.239 (1.041-1.475) | 0.214 | 1.063 (0.966-1.170) | 0.621 | 1.129 (1.039-1.228) | 0.053 |
| HMOX2 | 1.104 (1.031-1.182) | 0.042 | 1.188 (1.051-1.344) | 0.117 | 1.076 (0.992-1.167) | 0.381 | 1.248 (1.087-1.432) | 0.154 | 1.048 (0.964-1.138) | 0.677 | 1.106 (1.033-1.184) | 0.049 |
| HPGDS | 0.878 (0.813-0.948) | 0.013 | 0.821 (0.703-0.959) | 0.137 | 0.899 (0.823-0.982) | 0.165 | 0.818 (0.699-0.957) | 0.208 | 0.933 (0.856-1.018) | 0.507 | 0.890 (0.825-0.961) | 0.039 |
| HPSE | 1.141 (1.049-1.241) | 0.025 | 1.249 (1.046-1.490) | 0.141 | 1.112 (1.010-1.224) | 0.227 | 1.274 (1.069-1.519) | 0.191 | 1.089 (0.990-1.197) | 0.407 | 1.144 (1.051-1.244) | 0.030 |
| HYAL1 | 1.111 (1.034-1.195) | 0.039 | 1.153 (0.984-1.351) | 0.262 | 1.074 (0.987-1.168) | 0.419 | 1.008 (0.859-1.183) | 0.971 | 1.075 (0.989-1.168) | 0.430 | 1.093 (1.016-1.176) | 0.118 |
| ICAM1 | 1.183 (1.100-1.272) | 4.09E-04 | 1.270 (1.098-1.468) | 0.076 | 1.150 (1.056-1.252) | 0.033 | 1.093 (0.941-1.270) | 0.564 | 1.158 (1.064-1.259) | 0.026 | 1.178 (1.095-1.267) | 0.001 |
| IDUA | 1.147 (1.064-1.238) | 0.007 | 1.151 (0.992-1.335) | 0.236 | 1.137 (1.041-1.242) | 0.065 | 1.071 (0.922-1.243) | 0.682 | 1.139 (1.045-1.242) | 0.066 | 1.143 (1.059-1.232) | 0.014 |
| IFI30 | 1.209 (1.108-1.318) | 0.001 | 1.082 (0.901-1.300) | 0.650 | 1.241 (1.124-1.371) | 0.003 | 1.031 (0.864-1.231) | 0.902 | 1.202 (1.088-1.328) | 0.018 | 1.190 (1.091-1.299) | 0.005 |
| IGF2R | 1.131 (1.047-1.221) | 0.021 | 1.185 (1.012-1.389) | 0.182 | 1.111 (1.017-1.213) | 0.169 | 1.110 (0.953-1.293) | 0.483 | 1.114 (1.020-1.216) | 0.175 | 1.127 (1.043-1.217) | 0.034 |
| IGFBP4 | 1.204 (1.116-1.299) | 1.60E-04 | 1.191 (1.055-1.344) | 0.112 | 1.196 (1.085-1.317) | 0.014 | 1.165 (1.007-1.348) | 0.258 | 1.160 (1.060-1.270) | 0.038 | 1.144 (1.059-1.237) | 0.016 |
| IGSF8 | 1.179 (1.098-1.265) | 4.02E-04 | 1.307 (1.139-1.501) | 0.046 | 1.123 (1.031-1.223) | 0.098 | 1.088 (0.955-1.240) | 0.511 | 1.157 (1.064-1.259) | 0.027 | 1.154 (1.076-1.239) | 0.004 |
| IL10 | 1.151 (1.082-1.224) | 0.001 | 1.115 (0.966-1.286) | 0.363 | 1.158 (1.081-1.241) | 0.003 | 1.228 (1.094-1.378) | 0.122 | 1.110 (1.029-1.197) | 0.101 | 1.144 (1.074-1.218) | 0.002 |
| IL10RB | 1.126 (1.049-1.208) | 0.015 | 1.141 (0.996-1.307) | 0.225 | 1.116 (1.026-1.214) | 0.115 | 1.105 (0.956-1.278) | 0.483 | 1.091 (1.003-1.186) | 0.292 | 1.101 (1.024-1.184) | 0.083 |
| IL15 | 1.152 (1.073-1.237) | 0.003 | 1.058 (0.919-1.217) | 0.676 | 1.191 (1.097-1.294) | 0.003 | 1.183 (1.027-1.362) | 0.217 | 1.121 (1.033-1.218) | 0.098 | 1.137 (1.059-1.220) | 0.011 |
| IL17F | 1.145 (1.075-1.219) | 0.001 | 1.113 (0.954-1.298) | 0.412 | 1.157 (1.080-1.239) | 0.003 | 1.193 (1.045-1.361) | 0.195 | 1.137 (1.058-1.222) | 0.022 | 1.147 (1.077-1.221) | 0.002 |
| IL18 | 1.153 (1.073-1.238) | 0.003 | 1.196 (1.029-1.390) | 0.152 | 1.143 (1.053-1.241) | 0.033 | 1.036 (0.885-1.212) | 0.874 | 1.159 (1.069-1.258) | 0.020 | 1.151 (1.071-1.236) | 0.006 |
| IL18BP | 1.192 (1.109-1.282) | 1.84E-04 | 1.243 (1.088-1.420) | 0.076 | 1.171 (1.074-1.277) | 0.015 | 1.158 (1.001-1.341) | 0.271 | 1.148 (1.055-1.250) | 0.040 | 1.166 (1.084-1.255) | 0.003 |
| IL18R1 | 1.206 (1.119-1.300) | 1.11E-04 | 1.115 (0.963-1.292) | 0.377 | 1.223 (1.121-1.335) | 0.001 | 0.872 (0.747-1.018) | 0.336 | 1.264 (1.161-1.376) | 3.75E-05 | 1.193 (1.107-1.287) | 0.001 |
| IL19 | 1.146 (1.066-1.232) | 0.005 | 1.158 (1.012-1.324) | 0.178 | 1.139 (1.044-1.241) | 0.055 | 1.127 (0.973-1.305) | 0.385 | 1.127 (1.037-1.226) | 0.084 | 1.128 (1.049-1.213) | 0.023 |
| IL1RL1 | 1.161 (1.071-1.260) | 0.006 | 1.281 (1.083-1.516) | 0.109 | 1.119 (1.020-1.229) | 0.159 | 1.058 (0.897-1.248) | 0.774 | 1.154 (1.052-1.266) | 0.056 | 1.144 (1.055-1.241) | 0.023 |
| IL1RN | 1.195 (1.111-1.286) | 1.58E-04 | 1.267 (1.091-1.472) | 0.085 | 1.163 (1.068-1.267) | 0.018 | 0.971 (0.828-1.140) | 0.900 | 1.218 (1.121-1.323) | 0.001 | 1.185 (1.101-1.277) | 0.001 |
| IL2RA | 1.166 (1.085-1.254) | 0.001 | 1.124 (0.974-1.296) | 0.322 | 1.187 (1.091-1.292) | 0.005 | 1.103 (0.954-1.275) | 0.490 | 1.144 (1.052-1.244) | 0.044 | 1.145 (1.065-1.232) | 0.009 |
| IL4R | 1.217 (1.137-1.303) | 5.71E-06 | 1.133 (0.994-1.292) | 0.233 | 1.241 (1.146-1.345) | 9.47E-05 | 1.096 (0.950-1.263) | 0.520 | 1.209 (1.118-1.307) | 0.001 | 1.195 (1.116-1.279) | 1.44E-04 |
| IL6 | 1.143 (1.066-1.226) | 0.004 | 1.126 (0.978-1.296) | 0.302 | 1.152 (1.063-1.249) | 0.020 | 0.977 (0.817-1.168) | 0.917 | 1.164 (1.078-1.257) | 0.010 | 1.138 (1.061-1.221) | 0.009 |
| ILKAP | 1.111 (1.035-1.192) | 0.035 | 1.200 (1.036-1.389) | 0.143 | 1.089 (1.004-1.182) | 0.261 | 1.284 (1.091-1.510) | 0.154 | 1.075 (0.991-1.168) | 0.411 | 1.116 (1.040-1.198) | 0.033 |
| IMMT | 1.168 (1.089-1.253) | 0.001 | 1.042 (0.881-1.232) | 0.822 | 1.191 (1.103-1.286) | 0.002 | 1.259 (1.076-1.473) | 0.175 | 1.136 (1.046-1.233) | 0.057 | 1.157 (1.078-1.242) | 0.004 |
| INSL3 | 0.778 (0.670-0.903) | 0.014 | 0.747 (0.568-0.981) | 0.182 | 0.804 (0.672-0.962) | 0.157 | 0.893 (0.647-1.232) | 0.764 | 0.773 (0.653-0.916) | 0.063 | 0.781 (0.673-0.907) | 0.023 |
| ITGA11 | 0.875 (0.812-0.943) | 0.009 | 0.963 (0.835-1.110) | 0.798 | 0.856 (0.784-0.935) | 0.019 | 1.079 (0.934-1.247) | 0.621 | 0.859 (0.788-0.937) | 0.025 | 0.886 (0.822-0.955) | 0.028 |
| ITGA5 | 1.116 (1.033-1.206) | 0.047 | 1.170 (1.004-1.363) | 0.199 | 1.090 (0.996-1.193) | 0.326 | 1.005 (0.870-1.162) | 0.983 | 1.113 (1.018-1.216) | 0.196 | 1.107 (1.025-1.196) | 0.086 |
| ITGAV | 0.844 (0.781-0.911) | 0.001 | 0.885 (0.760-1.031) | 0.338 | 0.842 (0.770-0.921) | 0.009 | 1.054 (0.900-1.235) | 0.782 | 0.831 (0.761-0.907) | 0.004 | 0.857 (0.793-0.926) | 0.005 |
| ITGBL1 | 1.159 (1.071-1.255) | 0.006 | 1.132 (0.985-1.301) | 0.267 | 1.157 (1.050-1.275) | 0.055 | 1.069 (0.934-1.222) | 0.650 | 1.158 (1.055-1.272) | 0.054 | 1.141 (1.053-1.235) | 0.023 |
| ITIH3 | 1.165 (1.074-1.263) | 0.005 | 1.111 (0.939-1.316) | 0.478 | 1.166 (1.062-1.279) | 0.031 | 1.112 (0.934-1.324) | 0.551 | 1.164 (1.062-1.276) | 0.036 | 1.149 (1.060-1.246) | 0.018 |
| KIR3DL1 | 1.109 (1.031-1.192) | 0.049 | 1.056 (0.913-1.221) | 0.705 | 1.130 (1.039-1.230) | 0.066 | 1.218 (1.047-1.417) | 0.205 | 1.081 (0.994-1.175) | 0.371 | 1.109 (1.032-1.193) | 0.057 |
| KLK4 | 1.138 (1.055-1.226) | 0.012 | 1.076 (0.935-1.239) | 0.572 | 1.163 (1.063-1.273) | 0.028 | 1.002 (0.848-1.183) | 0.993 | 1.151 (1.057-1.253) | 0.037 | 1.112 (1.031-1.199) | 0.061 |
| KLRB1 | 1.140 (1.060-1.228) | 0.008 | 1.103 (0.962-1.264) | 0.395 | 1.142 (1.046-1.246) | 0.054 | 0.984 (0.842-1.150) | 0.932 | 1.158 (1.066-1.259) | 0.025 | 1.114 (1.034-1.200) | 0.053 |
| KRT18 | 1.117 (1.034-1.208) | 0.045 | 1.012 (0.863-1.188) | 0.949 | 1.137 (1.039-1.244) | 0.073 | 1.062 (0.910-1.239) | 0.735 | 1.095 (1.001-1.199) | 0.313 | 1.104 (1.020-1.194) | 0.106 |
| KRT19 | 1.186 (1.103-1.275) | 2.82E-04 | 1.158 (1.008-1.331) | 0.189 | 1.180 (1.084-1.285) | 0.008 | 1.069 (0.918-1.245) | 0.700 | 1.168 (1.075-1.269) | 0.015 | 1.158 (1.076-1.245) | 0.005 |
| LAIR1 | 1.132 (1.059-1.211) | 0.006 | 1.121 (0.987-1.273) | 0.264 | 1.131 (1.045-1.225) | 0.046 | 1.058 (0.889-1.258) | 0.791 | 1.125 (1.043-1.213) | 0.054 | 1.103 (1.029-1.182) | 0.060 |
| LAMP3 | 1.147 (1.059-1.242) | 0.012 | 1.167 (0.997-1.365) | 0.219 | 1.144 (1.043-1.254) | 0.065 | 1.162 (1.001-1.348) | 0.271 | 1.091 (0.995-1.195) | 0.361 | 1.133 (1.047-1.227) | 0.032 |
| LEP | 1.311 (1.145-1.501) | 0.003 | 1.453 (1.098-1.923) | 0.118 | 1.245 (1.066-1.454) | 0.078 | 0.989 (0.758-1.289) | 0.979 | 1.334 (1.144-1.556) | 0.015 | 1.275 (1.114-1.460) | 0.012 |
| LETM1 | 1.129 (1.044-1.221) | 0.025 | 1.241 (1.060-1.454) | 0.117 | 1.091 (0.995-1.195) | 0.335 | 1.267 (1.067-1.505) | 0.191 | 1.085 (0.991-1.188) | 0.399 | 1.120 (1.035-1.211) | 0.055 |
| LGALS4 | 1.220 (1.133-1.314) | 3.17E-05 | 1.187 (1.031-1.368) | 0.144 | 1.191 (1.086-1.306) | 0.010 | 1.035 (0.890-1.202) | 0.874 | 1.207 (1.108-1.315) | 0.003 | 1.174 (1.088-1.267) | 0.003 |
| LGALS9 | 1.196 (1.101-1.299) | 0.001 | 1.072 (0.911-1.261) | 0.656 | 1.231 (1.119-1.355) | 0.003 | 1.061 (0.899-1.252) | 0.760 | 1.174 (1.068-1.291) | 0.033 | 1.161 (1.068-1.261) | 0.012 |
| LILRA5 | 1.193 (1.102-1.291) | 0.001 | 1.228 (1.055-1.430) | 0.118 | 1.170 (1.066-1.283) | 0.027 | 1.051 (0.886-1.245) | 0.825 | 1.210 (1.107-1.324) | 0.004 | 1.182 (1.092-1.279) | 0.003 |
| LMNB2 | 1.159 (1.067-1.259) | 0.008 | 1.124 (0.966-1.307) | 0.355 | 1.162 (1.054-1.282) | 0.049 | 1.121 (0.956-1.315) | 0.453 | 1.125 (1.022-1.239) | 0.177 | 1.139 (1.049-1.237) | 0.032 |
| LRG1 | 1.158 (1.065-1.259) | 0.010 | 1.107 (0.931-1.316) | 0.511 | 1.165 (1.058-1.282) | 0.039 | 1.105 (0.919-1.329) | 0.611 | 1.149 (1.045-1.263) | 0.076 | 1.154 (1.062-1.254) | 0.018 |
| LRIG1 | 1.148 (1.066-1.237) | 0.006 | 1.222 (1.049-1.424) | 0.123 | 1.129 (1.036-1.230) | 0.078 | 1.013 (0.865-1.186) | 0.954 | 1.144 (1.051-1.245) | 0.049 | 1.134 (1.053-1.222) | 0.020 |
| LRRN1 | 0.857 (0.794-0.925) | 0.002 | 0.956 (0.820-1.114) | 0.777 | 0.832 (0.762-0.909) | 0.004 | 0.872 (0.745-1.021) | 0.347 | 0.883 (0.809-0.963) | 0.085 | 0.867 (0.803-0.936) | 0.008 |
| LTBP3 | 1.177 (1.105-1.254) | 6.60E-05 | 1.243 (1.105-1.398) | 0.046 | 1.159 (1.076-1.248) | 0.007 | 1.136 (0.983-1.313) | 0.339 | 1.176 (1.095-1.264) | 0.002 | 1.171 (1.098-1.249) | 3.20E-04 |
| MAD1L1 | 1.140 (1.068-1.216) | 0.002 | 1.287 (1.137-1.457) | 0.046 | 1.104 (1.022-1.192) | 0.128 | 1.211 (1.076-1.361) | 0.154 | 1.085 (1.000-1.177) | 0.319 | 1.133 (1.060-1.210) | 0.008 |
| MAP1LC3A | 0.881 (0.812-0.956) | 0.026 | 0.979 (0.829-1.157) | 0.911 | 0.853 (0.777-0.937) | 0.027 | 0.928 (0.782-1.100) | 0.697 | 0.878 (0.801-0.964) | 0.097 | 0.882 (0.813-0.957) | 0.035 |
| MARCO | 1.135 (1.050-1.227) | 0.017 | 1.113 (0.952-1.301) | 0.422 | 1.138 (1.040-1.245) | 0.068 | 1.023 (0.869-1.206) | 0.914 | 1.129 (1.034-1.233) | 0.101 | 1.131 (1.046-1.222) | 0.031 |
| MDK | 1.143 (1.065-1.227) | 0.005 | 1.229 (1.077-1.403) | 0.091 | 1.104 (1.014-1.201) | 0.181 | 1.086 (0.932-1.264) | 0.611 | 1.124 (1.036-1.220) | 0.085 | 1.129 (1.051-1.213) | 0.020 |
| MEP1B | 0.861 (0.801-0.926) | 0.002 | 0.817 (0.705-0.947) | 0.117 | 0.882 (0.811-0.959) | 0.055 | 0.787 (0.671-0.925) | 0.170 | 0.889 (0.819-0.965) | 0.084 | 0.870 (0.809-0.935) | 0.007 |
| MERTK | 1.132 (1.050-1.221) | 0.016 | 1.054 (0.911-1.220) | 0.715 | 1.151 (1.054-1.256) | 0.039 | 1.053 (0.914-1.215) | 0.754 | 1.093 (1.002-1.193) | 0.306 | 1.114 (1.033-1.201) | 0.055 |
| METAP1D | 1.115 (1.034-1.204) | 0.044 | 1.171 (1.002-1.368) | 0.206 | 1.098 (1.006-1.199) | 0.251 | 1.131 (0.971-1.318) | 0.386 | 1.091 (1.000-1.191) | 0.319 | 1.123 (1.041-1.213) | 0.038 |
| MILR1 | 1.131 (1.051-1.217) | 0.014 | 1.135 (0.982-1.311) | 0.281 | 1.134 (1.042-1.234) | 0.060 | 1.128 (0.978-1.301) | 0.367 | 1.101 (1.011-1.198) | 0.228 | 1.121 (1.041-1.207) | 0.034 |
| MME | 1.130 (1.050-1.217) | 0.016 | 0.999 (0.859-1.162) | 0.995 | 1.156 (1.061-1.259) | 0.027 | 0.928 (0.797-1.080) | 0.653 | 1.141 (1.049-1.242) | 0.054 | 1.122 (1.042-1.209) | 0.035 |
| MMP3 | 1.152 (1.052-1.261) | 0.025 | 1.073 (0.903-1.276) | 0.671 | 1.179 (1.061-1.310) | 0.044 | 1.220 (1.027-1.449) | 0.217 | 1.101 (0.991-1.224) | 0.389 | 1.115 (1.019-1.220) | 0.122 |
| MMP7 | 1.142 (1.052-1.239) | 0.018 | 1.153 (0.992-1.342) | 0.237 | 1.118 (1.016-1.231) | 0.185 | 1.071 (0.904-1.269) | 0.722 | 1.113 (1.014-1.221) | 0.217 | 1.108 (1.022-1.202) | 0.102 |
| MMP9 | 1.137 (1.053-1.227) | 0.015 | 1.077 (0.919-1.262) | 0.620 | 1.146 (1.049-1.251) | 0.048 | 1.071 (0.911-1.259) | 0.707 | 1.182 (1.083-1.290) | 0.014 | 1.130 (1.046-1.220) | 0.031 |
| MPO | 1.123 (1.049-1.202) | 0.013 | 1.152 (0.997-1.330) | 0.218 | 1.114 (1.030-1.204) | 0.092 | 1.120 (0.972-1.289) | 0.390 | 1.121 (1.036-1.212) | 0.079 | 1.118 (1.044-1.197) | 0.027 |
| MRC1 | 1.213 (1.118-1.315) | 2.58E-04 | 1.147 (0.978-1.346) | 0.290 | 1.223 (1.113-1.344) | 0.003 | 1.114 (0.935-1.329) | 0.542 | 1.203 (1.097-1.319) | 0.009 | 1.205 (1.112-1.307) | 0.001 |
| MSR1 | 1.136 (1.047-1.233) | 0.025 | 1.083 (0.922-1.272) | 0.594 | 1.152 (1.048-1.267) | 0.057 | 1.065 (0.909-1.249) | 0.726 | 1.111 (1.012-1.220) | 0.232 | 1.118 (1.030-1.213) | 0.073 |
| MVK | 1.167 (1.081-1.258) | 0.002 | 1.106 (0.952-1.286) | 0.436 | 1.160 (1.061-1.269) | 0.029 | 1.213 (1.051-1.400) | 0.193 | 1.111 (1.017-1.213) | 0.196 | 1.156 (1.071-1.247) | 0.008 |
| MYBPC2 | 1.126 (1.049-1.210) | 0.015 | 1.169 (1.006-1.358) | 0.196 | 1.118 (1.029-1.214) | 0.104 | 1.142 (0.954-1.367) | 0.437 | 1.132 (1.044-1.226) | 0.058 | 1.118 (1.040-1.202) | 0.035 |
| NAGPA | 1.133 (1.041-1.232) | 0.036 | 1.167 (0.988-1.378) | 0.246 | 1.121 (1.017-1.236) | 0.180 | 1.124 (0.952-1.327) | 0.470 | 1.102 (1.001-1.213) | 0.311 | 1.134 (1.042-1.234) | 0.043 |
| NCR3LG1 | 1.145 (1.052-1.246) | 0.020 | 1.188 (1.017-1.389) | 0.173 | 1.130 (1.023-1.249) | 0.153 | 1.152 (0.971-1.366) | 0.376 | 1.095 (0.993-1.207) | 0.377 | 1.123 (1.032-1.222) | 0.068 |
| NCS1 | 1.147 (1.058-1.243) | 0.013 | 1.180 (1.018-1.368) | 0.171 | 1.112 (1.010-1.223) | 0.223 | 1.047 (0.889-1.233) | 0.832 | 1.089 (0.992-1.195) | 0.385 | 1.117 (1.031-1.211) | 0.068 |
| NDUFA5 | 1.128 (1.040-1.223) | 0.036 | 1.235 (1.048-1.455) | 0.128 | 1.100 (1.002-1.207) | 0.286 | 1.046 (0.883-1.240) | 0.844 | 1.126 (1.027-1.235) | 0.139 | 1.124 (1.037-1.219) | 0.053 |
| NECTIN2 | 1.177 (1.095-1.264) | 0.001 | 1.224 (1.086-1.380) | 0.066 | 1.147 (1.049-1.253) | 0.048 | 1.134 (0.983-1.306) | 0.336 | 1.130 (1.038-1.229) | 0.081 | 1.146 (1.066-1.232) | 0.008 |
| NHLRC3 | 1.202 (1.102-1.312) | 0.001 | 1.090 (0.916-1.298) | 0.595 | 1.214 (1.097-1.343) | 0.009 | 1.102 (0.940-1.292) | 0.549 | 1.173 (1.061-1.296) | 0.049 | 1.189 (1.090-1.298) | 0.005 |
| NID1 | 1.147 (1.065-1.234) | 0.006 | 1.261 (1.086-1.464) | 0.093 | 1.111 (1.020-1.210) | 0.147 | 1.200 (1.035-1.391) | 0.214 | 1.120 (1.030-1.219) | 0.113 | 1.145 (1.063-1.232) | 0.010 |
| NPC2 | 1.150 (1.062-1.245) | 0.009 | 1.177 (1.007-1.376) | 0.193 | 1.128 (1.027-1.239) | 0.126 | 1.145 (0.966-1.357) | 0.392 | 1.108 (1.008-1.218) | 0.254 | 1.119 (1.032-1.215) | 0.068 |
| NPDC1 | 1.131 (1.052-1.215) | 0.013 | 1.133 (1.005-1.278) | 0.192 | 1.120 (1.023-1.226) | 0.139 | 1.026 (0.878-1.198) | 0.907 | 1.134 (1.045-1.232) | 0.060 | 1.091 (1.013-1.174) | 0.135 |
| NT5C1A | 1.124 (1.048-1.205) | 0.015 | 1.183 (1.007-1.390) | 0.192 | 1.109 (1.023-1.203) | 0.126 | 1.298 (1.116-1.511) | 0.136 | 1.051 (0.959-1.153) | 0.693 | 1.113 (1.036-1.197) | 0.045 |
| NTproBNP | 1.144 (1.060-1.235) | 0.010 | 1.026 (0.897-1.173) | 0.863 | 1.203 (1.095-1.322) | 0.008 | 1.183 (1.026-1.365) | 0.217 | 1.104 (1.010-1.206) | 0.239 | 1.090 (1.008-1.179) | 0.167 |
| NTRK3 | 0.866 (0.802-0.936) | 0.006 | 0.907 (0.776-1.059) | 0.473 | 0.873 (0.797-0.955) | 0.055 | 0.935 (0.799-1.094) | 0.701 | 0.861 (0.788-0.941) | 0.033 | 0.880 (0.814-0.951) | 0.026 |
| NUCB2 | 1.112 (1.032-1.199) | 0.046 | 1.256 (1.083-1.457) | 0.095 | 1.067 (0.978-1.163) | 0.519 | 1.099 (0.941-1.283) | 0.550 | 1.089 (1.000-1.187) | 0.320 | 1.100 (1.021-1.186) | 0.100 |
| OCLN | 1.210 (1.114-1.313) | 3.93E-04 | 1.185 (1.010-1.390) | 0.186 | 1.203 (1.092-1.326) | 0.009 | 1.193 (1.018-1.398) | 0.234 | 1.142 (1.037-1.258) | 0.104 | 1.179 (1.085-1.280) | 0.005 |
| OLR1 | 1.131 (1.058-1.210) | 0.006 | 1.113 (0.964-1.284) | 0.374 | 1.134 (1.051-1.224) | 0.031 | 1.149 (0.990-1.334) | 0.309 | 1.141 (1.058-1.231) | 0.026 | 1.131 (1.057-1.209) | 0.010 |
| OMG | 0.878 (0.814-0.948) | 0.013 | 0.818 (0.704-0.950) | 0.118 | 0.901 (0.825-0.985) | 0.180 | 0.995 (0.846-1.170) | 0.983 | 0.855 (0.784-0.933) | 0.022 | 0.883 (0.818-0.953) | 0.026 |
| OSCAR | 1.118 (1.036-1.207) | 0.039 | 1.148 (0.993-1.329) | 0.234 | 1.112 (1.017-1.216) | 0.172 | 1.065 (0.916-1.238) | 0.711 | 1.093 (1.002-1.193) | 0.301 | 1.099 (1.019-1.186) | 0.110 |
| OSM | 1.162 (1.077-1.253) | 0.003 | 1.198 (1.024-1.401) | 0.163 | 1.141 (1.046-1.245) | 0.055 | 1.017 (0.866-1.194) | 0.932 | 1.231 (1.129-1.342) | 0.001 | 1.154 (1.069-1.245) | 0.008 |
| OSMR | 1.165 (1.082-1.253) | 0.002 | 1.371 (1.172-1.604) | 0.046 | 1.112 (1.022-1.211) | 0.136 | 1.157 (0.979-1.367) | 0.343 | 1.138 (1.047-1.237) | 0.055 | 1.163 (1.080-1.251) | 0.004 |
| OXCT1 | 1.138 (1.054-1.230) | 0.014 | 1.344 (1.145-1.578) | 0.046 | 1.085 (0.992-1.186) | 0.371 | 1.238 (1.058-1.450) | 0.193 | 1.090 (0.998-1.191) | 0.337 | 1.141 (1.055-1.232) | 0.020 |
| PAG1 | 1.146 (1.068-1.229) | 0.004 | 1.369 (1.202-1.560) | 0.007 | 1.077 (0.991-1.170) | 0.394 | 1.174 (1.027-1.341) | 0.217 | 1.118 (1.030-1.213) | 0.106 | 1.140 (1.062-1.223) | 0.009 |
| PALM2 | 1.177 (1.080-1.282) | 0.005 | 1.053 (0.886-1.250) | 0.776 | 1.209 (1.095-1.335) | 0.009 | 1.107 (0.928-1.321) | 0.575 | 1.143 (1.034-1.264) | 0.116 | 1.153 (1.058-1.256) | 0.023 |
| PDZK1 | 1.142 (1.059-1.231) | 0.009 | 1.052 (0.902-1.228) | 0.747 | 1.165 (1.069-1.271) | 0.019 | 1.149 (1.004-1.316) | 0.263 | 1.098 (1.003-1.202) | 0.292 | 1.130 (1.048-1.218) | 0.027 |
| PGF | 1.115 (1.037-1.200) | 0.035 | 1.173 (1.018-1.352) | 0.171 | 1.092 (1.000-1.192) | 0.301 | 1.034 (0.877-1.220) | 0.886 | 1.122 (1.033-1.218) | 0.097 | 1.082 (1.003-1.168) | 0.199 |
| PI3 | 1.143 (1.062-1.229) | 0.007 | 1.201 (1.064-1.357) | 0.100 | 1.114 (1.019-1.218) | 0.163 | 1.055 (0.906-1.229) | 0.765 | 1.153 (1.061-1.253) | 0.030 | 1.120 (1.041-1.206) | 0.035 |
| PIGR | 1.223 (1.136-1.316) | 2.31E-05 | 1.222 (1.065-1.401) | 0.112 | 1.201 (1.101-1.309) | 0.003 | 1.078 (0.920-1.262) | 0.669 | 1.203 (1.105-1.309) | 0.003 | 1.197 (1.111-1.290) | 4.19E-04 |
| PKD1 | 1.141 (1.045-1.246) | 0.033 | 1.146 (0.965-1.361) | 0.341 | 1.131 (1.021-1.253) | 0.163 | 1.223 (1.032-1.448) | 0.217 | 1.083 (0.980-1.198) | 0.506 | 1.128 (1.033-1.231) | 0.072 |
| PLA2G15 | 1.210 (1.125-1.303) | 5.93E-05 | 1.220 (1.060-1.404) | 0.117 | 1.205 (1.106-1.314) | 0.003 | 1.018 (0.881-1.176) | 0.920 | 1.191 (1.093-1.297) | 0.007 | 1.197 (1.113-1.287) | 3.20E-04 |
| PLAUR | 1.264 (1.171-1.364) | 1.41E-06 | 1.296 (1.126-1.492) | 0.046 | 1.244 (1.136-1.363) | 0.001 | 1.203 (1.035-1.399) | 0.214 | 1.232 (1.127-1.346) | 0.001 | 1.229 (1.137-1.328) | 9.34E-05 |
| PLIN3 | 1.128 (1.049-1.214) | 0.016 | 1.308 (1.132-1.512) | 0.046 | 1.068 (0.981-1.163) | 0.494 | 1.170 (1.015-1.349) | 0.240 | 1.080 (0.992-1.175) | 0.396 | 1.120 (1.041-1.205) | 0.035 |
| PLXNB2 | 1.121 (1.042-1.207) | 0.025 | 1.162 (1.020-1.325) | 0.164 | 1.078 (0.986-1.178) | 0.424 | 1.023 (0.879-1.191) | 0.913 | 1.112 (1.022-1.209) | 0.158 | 1.106 (1.027-1.191) | 0.075 |
| POF1B | 1.120 (1.043-1.202) | 0.021 | 1.073 (0.919-1.253) | 0.631 | 1.127 (1.040-1.221) | 0.059 | 1.060 (0.896-1.254) | 0.770 | 1.101 (1.013-1.195) | 0.210 | 1.104 (1.027-1.186) | 0.068 |
| POLR2F | 1.123 (1.051-1.200) | 0.010 | 1.207 (1.052-1.385) | 0.117 | 1.103 (1.021-1.191) | 0.129 | 1.189 (1.065-1.328) | 0.154 | 1.081 (0.996-1.174) | 0.358 | 1.115 (1.041-1.194) | 0.030 |
| PON1 | 0.878 (0.822-0.938) | 0.003 | 0.834 (0.731-0.951) | 0.117 | 0.896 (0.828-0.969) | 0.080 | 0.865 (0.763-0.979) | 0.217 | 0.889 (0.823-0.960) | 0.059 | 0.885 (0.827-0.947) | 0.011 |
| PRAP1 | 1.173 (1.068-1.289) | 0.013 | 1.055 (0.879-1.267) | 0.777 | 1.177 (1.053-1.316) | 0.064 | 1.016 (0.849-1.217) | 0.948 | 1.168 (1.049-1.301) | 0.082 | 1.143 (1.040-1.255) | 0.060 |
| PRSS8 | 1.190 (1.096-1.292) | 0.001 | 1.153 (0.981-1.356) | 0.272 | 1.157 (1.048-1.278) | 0.063 | 1.109 (0.947-1.300) | 0.509 | 1.141 (1.037-1.255) | 0.100 | 1.153 (1.061-1.253) | 0.019 |
| PRTN3 | 1.144 (1.076-1.216) | 0.001 | 1.192 (1.069-1.328) | 0.077 | 1.126 (1.047-1.211) | 0.033 | 1.178 (1.031-1.345) | 0.214 | 1.132 (1.054-1.215) | 0.026 | 1.141 (1.073-1.214) | 0.002 |
| PSAP | 1.128 (1.054-1.207) | 0.009 | 1.215 (1.030-1.434) | 0.154 | 1.108 (1.025-1.198) | 0.113 | 1.227 (1.034-1.455) | 0.217 | 1.103 (1.017-1.196) | 0.185 | 1.116 (1.041-1.197) | 0.032 |
| PTPRF | 0.885 (0.820-0.956) | 0.022 | 0.918 (0.791-1.065) | 0.525 | 0.889 (0.813-0.973) | 0.120 | 0.895 (0.760-1.054) | 0.487 | 0.895 (0.821-0.977) | 0.149 | 0.886 (0.821-0.957) | 0.031 |
| PTX3 | 1.151 (1.060-1.250) | 0.013 | 1.159 (0.975-1.379) | 0.299 | 1.151 (1.048-1.264) | 0.057 | 1.011 (0.850-1.202) | 0.965 | 1.198 (1.089-1.317) | 0.014 | 1.140 (1.051-1.238) | 0.029 |
| QPCT | 1.117 (1.036-1.204) | 0.038 | 1.219 (1.061-1.401) | 0.116 | 1.077 (0.985-1.178) | 0.432 | 1.043 (0.901-1.208) | 0.827 | 1.123 (1.030-1.224) | 0.113 | 1.099 (1.019-1.186) | 0.107 |
| QSOX1 | 1.095 (1.032-1.162) | 0.030 | 1.203 (1.057-1.369) | 0.116 | 1.073 (1.001-1.151) | 0.287 | 1.160 (1.009-1.333) | 0.252 | 1.083 (1.010-1.161) | 0.217 | 1.091 (1.027-1.158) | 0.055 |
| RAB6A | 0.896 (0.831-0.966) | 0.038 | 1.064 (0.932-1.215) | 0.623 | 0.853 (0.781-0.932) | 0.017 | 1.059 (0.946-1.186) | 0.636 | 0.858 (0.786-0.937) | 0.026 | 0.911 (0.845-0.981) | 0.108 |
| RARRES2 | 1.165 (1.076-1.262) | 0.004 | 1.265 (1.083-1.477) | 0.100 | 1.122 (1.023-1.231) | 0.146 | 1.004 (0.849-1.187) | 0.988 | 1.164 (1.064-1.274) | 0.033 | 1.149 (1.061-1.244) | 0.016 |
| RBFOX3 | 1.199 (1.105-1.300) | 0.001 | 1.185 (1.013-1.386) | 0.181 | 1.195 (1.086-1.315) | 0.013 | 1.174 (0.996-1.382) | 0.284 | 1.145 (1.039-1.262) | 0.097 | 1.167 (1.076-1.267) | 0.008 |
| RBP5 | 1.142 (1.055-1.237) | 0.015 | 0.993 (0.846-1.165) | 0.972 | 1.172 (1.068-1.285) | 0.026 | 0.934 (0.791-1.102) | 0.716 | 1.166 (1.064-1.277) | 0.034 | 1.118 (1.032-1.212) | 0.064 |
| REN | 1.143 (1.065-1.227) | 0.005 | 1.072 (0.957-1.201) | 0.495 | 1.138 (1.034-1.252) | 0.101 | 0.938 (0.807-1.091) | 0.709 | 1.172 (1.081-1.270) | 0.011 | 1.091 (1.015-1.174) | 0.126 |
| RNASE1 | 1.128 (1.047-1.214) | 0.018 | 1.064 (0.920-1.230) | 0.654 | 1.138 (1.044-1.241) | 0.055 | 1.140 (0.955-1.362) | 0.438 | 1.119 (1.028-1.219) | 0.120 | 1.095 (1.014-1.182) | 0.132 |
| RNASE4 | 1.131 (1.050-1.219) | 0.016 | 1.081 (0.928-1.260) | 0.584 | 1.133 (1.039-1.236) | 0.067 | 1.102 (0.913-1.331) | 0.632 | 1.132 (1.040-1.231) | 0.077 | 1.109 (1.026-1.198) | 0.080 |
| RNASE6 | 1.157 (1.083-1.235) | 0.001 | 1.085 (0.942-1.250) | 0.523 | 1.173 (1.089-1.264) | 0.003 | 1.091 (0.926-1.285) | 0.617 | 1.157 (1.074-1.246) | 0.011 | 1.131 (1.055-1.213) | 0.014 |
| RNASET2 | 1.151 (1.074-1.234) | 0.002 | 1.228 (1.092-1.381) | 0.064 | 1.109 (1.019-1.208) | 0.153 | 1.132 (0.976-1.313) | 0.372 | 1.137 (1.050-1.231) | 0.044 | 1.127 (1.050-1.209) | 0.020 |
| RNF149 | 1.155 (1.066-1.251) | 0.008 | 1.114 (0.968-1.282) | 0.359 | 1.165 (1.057-1.284) | 0.042 | 1.175 (1.013-1.363) | 0.243 | 1.103 (1.005-1.211) | 0.280 | 1.126 (1.039-1.220) | 0.047 |
| RPS10 | 0.854 (0.775-0.941) | 0.018 | 0.897 (0.741-1.086) | 0.531 | 0.838 (0.749-0.938) | 0.044 | 0.889 (0.724-1.093) | 0.580 | 0.858 (0.769-0.958) | 0.098 | 0.854 (0.775-0.941) | 0.027 |
| RRM2 | 1.105 (1.041-1.173) | 0.015 | 1.099 (0.988-1.222) | 0.271 | 1.100 (1.022-1.184) | 0.122 | 1.164 (1.008-1.344) | 0.254 | 1.071 (0.995-1.154) | 0.376 | 1.103 (1.038-1.172) | 0.028 |
| SCARB2 | 1.206 (1.118-1.302) | 1.54E-04 | 1.147 (1.005-1.309) | 0.196 | 1.232 (1.121-1.352) | 0.002 | 1.000 (0.845-1.182) | 0.999 | 1.218 (1.116-1.330) | 0.002 | 1.153 (1.066-1.247) | 0.011 |
| SCARF1 | 1.123 (1.044-1.207) | 0.022 | 1.301 (1.132-1.495) | 0.046 | 1.064 (0.977-1.159) | 0.531 | 1.176 (1.014-1.365) | 0.241 | 1.092 (1.005-1.187) | 0.268 | 1.120 (1.042-1.205) | 0.033 |
| SCGB1A1 | 0.853 (0.793-0.918) | 0.001 | 0.843 (0.729-0.974) | 0.153 | 0.858 (0.787-0.934) | 0.017 | 0.843 (0.725-0.979) | 0.224 | 0.861 (0.791-0.937) | 0.024 | 0.839 (0.780-0.903) | 4.54E-04 |
| SCGN | 1.129 (1.049-1.215) | 0.016 | 1.169 (1.023-1.337) | 0.158 | 1.100 (1.008-1.202) | 0.236 | 1.093 (0.937-1.276) | 0.575 | 1.108 (1.019-1.206) | 0.180 | 1.107 (1.028-1.192) | 0.069 |
| SCLY | 1.122 (1.039-1.212) | 0.035 | 0.982 (0.837-1.151) | 0.918 | 1.148 (1.050-1.255) | 0.046 | 1.010 (0.867-1.177) | 0.963 | 1.130 (1.035-1.235) | 0.100 | 1.122 (1.039-1.212) | 0.042 |
| SELENOP | 0.869 (0.800-0.943) | 0.012 | 0.826 (0.702-0.973) | 0.156 | 0.881 (0.801-0.968) | 0.106 | 0.950 (0.802-1.126) | 0.815 | 0.896 (0.815-0.984) | 0.202 | 0.871 (0.803-0.945) | 0.020 |
| SELP | 1.130 (1.050-1.215) | 0.015 | 1.315 (1.135-1.523) | 0.046 | 1.076 (0.989-1.171) | 0.408 | 1.240 (1.069-1.438) | 0.175 | 1.087 (1.000-1.182) | 0.319 | 1.132 (1.053-1.218) | 0.019 |
| SERPINA11 | 1.138 (1.048-1.236) | 0.025 | 0.990 (0.847-1.157) | 0.956 | 1.207 (1.096-1.330) | 0.008 | 1.012 (0.856-1.197) | 0.959 | 1.170 (1.065-1.285) | 0.035 | 1.133 (1.044-1.230) | 0.037 |
| SERPINA3 | 1.137 (1.064-1.214) | 0.004 | 1.172 (1.019-1.349) | 0.169 | 1.137 (1.054-1.227) | 0.027 | 1.078 (0.900-1.292) | 0.712 | 1.144 (1.065-1.230) | 0.015 | 1.135 (1.062-1.213) | 0.008 |
| SIAE | 1.137 (1.055-1.225) | 0.012 | 1.282 (1.096-1.499) | 0.085 | 1.098 (1.008-1.197) | 0.235 | 1.117 (0.959-1.301) | 0.448 | 1.107 (1.016-1.206) | 0.196 | 1.136 (1.054-1.225) | 0.019 |
| SIGLEC1 | 1.179 (1.091-1.275) | 0.001 | 1.196 (1.020-1.402) | 0.171 | 1.177 (1.076-1.288) | 0.016 | 1.050 (0.901-1.224) | 0.795 | 1.157 (1.058-1.266) | 0.040 | 1.160 (1.073-1.254) | 0.008 |
| SIGLEC10 | 1.164 (1.082-1.252) | 0.002 | 1.210 (1.053-1.389) | 0.117 | 1.144 (1.050-1.246) | 0.044 | 1.161 (1.004-1.343) | 0.263 | 1.121 (1.030-1.220) | 0.112 | 1.147 (1.066-1.235) | 0.008 |
| SIGLEC6 | 1.113 (1.038-1.194) | 0.029 | 1.159 (1.004-1.338) | 0.198 | 1.096 (1.010-1.189) | 0.212 | 0.988 (0.854-1.143) | 0.951 | 1.130 (1.044-1.223) | 0.057 | 1.107 (1.031-1.188) | 0.055 |
| SIT1 | 1.131 (1.046-1.223) | 0.024 | 1.253 (1.073-1.464) | 0.112 | 1.079 (0.985-1.182) | 0.430 | 1.038 (0.890-1.209) | 0.862 | 1.136 (1.038-1.242) | 0.089 | 1.119 (1.034-1.210) | 0.055 |
| SLAMF8 | 1.143 (1.055-1.237) | 0.014 | 1.029 (0.880-1.203) | 0.875 | 1.180 (1.075-1.294) | 0.018 | 1.041 (0.891-1.218) | 0.850 | 1.165 (1.064-1.276) | 0.034 | 1.130 (1.044-1.223) | 0.035 |
| SMPD1 | 1.149 (1.068-1.236) | 0.005 | 1.086 (0.939-1.256) | 0.533 | 1.174 (1.078-1.278) | 0.010 | 0.984 (0.855-1.133) | 0.929 | 1.131 (1.039-1.231) | 0.080 | 1.137 (1.057-1.223) | 0.015 |
| SOD2 | 1.121 (1.044-1.203) | 0.020 | 1.083 (0.943-1.244) | 0.523 | 1.124 (1.035-1.222) | 0.078 | 1.178 (1.024-1.356) | 0.217 | 1.063 (0.978-1.156) | 0.556 | 1.114 (1.037-1.196) | 0.039 |
| SORCS2 | 1.117 (1.034-1.206) | 0.043 | 1.141 (0.990-1.314) | 0.245 | 1.090 (0.995-1.195) | 0.338 | 1.071 (0.917-1.250) | 0.697 | 1.100 (1.007-1.202) | 0.257 | 1.075 (0.995-1.161) | 0.256 |
| SORT1 | 1.116 (1.036-1.202) | 0.037 | 1.172 (1.011-1.359) | 0.182 | 1.099 (1.008-1.198) | 0.235 | 1.126 (0.974-1.303) | 0.383 | 1.101 (1.011-1.199) | 0.235 | 1.118 (1.038-1.205) | 0.040 |
| SPINK1 | 1.113 (1.037-1.195) | 0.032 | 1.103 (0.973-1.249) | 0.348 | 1.099 (1.007-1.198) | 0.237 | 1.039 (0.910-1.187) | 0.825 | 1.101 (1.013-1.197) | 0.210 | 1.069 (0.994-1.150) | 0.261 |
| SPINK4 | 1.116 (1.037-1.202) | 0.036 | 1.191 (1.043-1.360) | 0.122 | 1.064 (0.973-1.164) | 0.554 | 1.035 (0.894-1.198) | 0.871 | 1.091 (1.001-1.188) | 0.309 | 1.090 (1.011-1.174) | 0.146 |
| SPON2 | 1.124 (1.040-1.214) | 0.032 | 1.008 (0.868-1.171) | 0.967 | 1.153 (1.052-1.264) | 0.046 | 1.043 (0.895-1.216) | 0.837 | 1.106 (1.012-1.208) | 0.232 | 1.089 (1.007-1.177) | 0.170 |
| TF | 1.137 (1.050-1.232) | 0.019 | 1.144 (0.980-1.337) | 0.285 | 1.123 (1.023-1.233) | 0.140 | 1.154 (0.990-1.346) | 0.307 | 1.106 (1.009-1.212) | 0.245 | 1.132 (1.045-1.226) | 0.034 |
| TFF2 | 1.151 (1.069-1.240) | 0.005 | 1.150 (0.999-1.325) | 0.215 | 1.138 (1.042-1.243) | 0.062 | 1.108 (0.949-1.293) | 0.505 | 1.118 (1.026-1.218) | 0.134 | 1.119 (1.038-1.207) | 0.042 |
| TFF3 | 1.143 (1.073-1.218) | 0.001 | 1.281 (1.127-1.456) | 0.046 | 1.101 (1.021-1.188) | 0.129 | 1.047 (0.924-1.187) | 0.753 | 1.110 (1.027-1.199) | 0.113 | 1.114 (1.042-1.190) | 0.027 |
| TGFA | 1.243 (1.158-1.334) | 1.41E-06 | 1.299 (1.132-1.491) | 0.046 | 1.218 (1.121-1.323) | 0.001 | 1.077 (0.923-1.257) | 0.665 | 1.282 (1.184-1.389) | 2.96E-06 | 1.226 (1.142-1.317) | 1.89E-05 |
| TGFB1 | 1.150 (1.072-1.234) | 0.003 | 1.191 (1.057-1.343) | 0.112 | 1.133 (1.041-1.232) | 0.060 | 1.182 (1.010-1.383) | 0.252 | 1.125 (1.037-1.219) | 0.080 | 1.146 (1.068-1.231) | 0.007 |
| THBS2 | 1.142 (1.066-1.223) | 0.004 | 1.129 (0.999-1.276) | 0.214 | 1.142 (1.051-1.240) | 0.039 | 1.079 (0.961-1.213) | 0.509 | 1.113 (1.025-1.208) | 0.131 | 1.129 (1.054-1.210) | 0.014 |
| TIMP1 | 1.199 (1.113-1.292) | 1.60E-04 | 1.244 (1.080-1.433) | 0.094 | 1.177 (1.078-1.285) | 0.012 | 1.189 (1.021-1.386) | 0.225 | 1.167 (1.071-1.272) | 0.022 | 1.181 (1.096-1.273) | 0.001 |
| TMED10 | 0.848 (0.756-0.952) | 0.044 | 0.972 (0.814-1.162) | 0.893 | 0.794 (0.687-0.917) | 0.039 | 0.910 (0.742-1.116) | 0.677 | 0.831 (0.725-0.952) | 0.106 | 0.847 (0.755-0.951) | 0.055 |
| TMED4 | 0.860 (0.780-0.949) | 0.029 | 0.766 (0.606-0.969) | 0.168 | 0.885 (0.794-0.986) | 0.208 | 0.744 (0.588-0.941) | 0.208 | 0.883 (0.793-0.985) | 0.223 | 0.861 (0.780-0.950) | 0.037 |
| TMSB10 | 1.151 (1.065-1.245) | 0.007 | 1.316 (1.120-1.545) | 0.066 | 1.104 (1.010-1.207) | 0.218 | 1.196 (1.016-1.408) | 0.241 | 1.111 (1.017-1.214) | 0.197 | 1.147 (1.061-1.240) | 0.014 |
| TNC | 1.122 (1.044-1.207) | 0.022 | 1.147 (0.997-1.320) | 0.220 | 1.127 (1.035-1.227) | 0.078 | 1.051 (0.900-1.228) | 0.792 | 1.117 (1.028-1.213) | 0.116 | 1.121 (1.043-1.206) | 0.031 |
| TNF | 1.120 (1.063-1.180) | 0.001 | 1.073 (0.954-1.208) | 0.502 | 1.125 (1.062-1.193) | 0.005 | 1.103 (1.012-1.202) | 0.223 | 1.095 (1.026-1.170) | 0.098 | 1.111 (1.053-1.173) | 0.006 |
| TNFRSF10A | 1.224 (1.145-1.308) | 1.41E-06 | 1.196 (1.043-1.371) | 0.124 | 1.211 (1.118-1.311) | 0.001 | 1.132 (0.968-1.325) | 0.396 | 1.177 (1.086-1.276) | 0.007 | 1.194 (1.113-1.281) | 1.91E-04 |
| TNFRSF10B | 1.161 (1.113-1.211) | 8.17E-09 | 1.159 (1.054-1.275) | 0.093 | 1.156 (1.102-1.214) | 7.12E-06 | 1.096 (0.971-1.236) | 0.425 | 1.155 (1.099-1.214) | 1.16E-05 | 1.155 (1.103-1.208) | 7.26E-07 |
| TNFRSF11A | 1.154 (1.071-1.244) | 0.005 | 1.148 (1.012-1.301) | 0.177 | 1.136 (1.034-1.248) | 0.098 | 1.084 (0.936-1.256) | 0.600 | 1.140 (1.045-1.244) | 0.066 | 1.112 (1.031-1.200) | 0.063 |
| TNFRSF11B | 1.160 (1.072-1.255) | 0.005 | 1.116 (0.954-1.306) | 0.409 | 1.173 (1.071-1.285) | 0.020 | 1.027 (0.877-1.203) | 0.903 | 1.150 (1.050-1.258) | 0.057 | 1.148 (1.061-1.243) | 0.016 |
| TNFRSF12A | 1.144 (1.058-1.237) | 0.012 | 1.168 (1.020-1.337) | 0.164 | 1.114 (1.012-1.225) | 0.209 | 1.103 (0.949-1.282) | 0.509 | 1.102 (1.006-1.208) | 0.266 | 1.103 (1.020-1.193) | 0.109 |
| TNFRSF14 | 1.178 (1.096-1.267) | 0.001 | 1.233 (1.089-1.396) | 0.066 | 1.144 (1.048-1.250) | 0.050 | 1.145 (1.002-1.310) | 0.269 | 1.134 (1.041-1.235) | 0.074 | 1.143 (1.063-1.230) | 0.009 |
| TNFRSF1A | 1.220 (1.130-1.317) | 5.93E-05 | 1.243 (1.092-1.414) | 0.066 | 1.200 (1.092-1.318) | 0.009 | 1.129 (0.975-1.308) | 0.376 | 1.197 (1.095-1.309) | 0.008 | 1.172 (1.084-1.267) | 0.004 |
| TNFRSF1B | 1.152 (1.095-1.213) | 2.01E-05 | 1.110 (0.998-1.235) | 0.220 | 1.163 (1.096-1.233) | 2.38E-04 | 1.138 (1.030-1.258) | 0.206 | 1.125 (1.055-1.201) | 0.020 | 1.136 (1.076-1.199) | 0.001 |
| TNFRSF4 | 1.138 (1.058-1.224) | 0.009 | 1.128 (0.988-1.287) | 0.258 | 1.135 (1.040-1.238) | 0.067 | 1.043 (0.905-1.202) | 0.816 | 1.113 (1.023-1.212) | 0.152 | 1.105 (1.026-1.189) | 0.076 |
| TNFRSF6B | 1.151 (1.068-1.241) | 0.006 | 1.153 (1.003-1.325) | 0.203 | 1.140 (1.043-1.246) | 0.063 | 1.101 (0.952-1.273) | 0.507 | 1.127 (1.033-1.229) | 0.101 | 1.129 (1.047-1.217) | 0.028 |
| TNFRSF8 | 1.138 (1.059-1.223) | 0.008 | 1.159 (1.003-1.340) | 0.200 | 1.138 (1.047-1.237) | 0.046 | 1.132 (0.981-1.306) | 0.348 | 1.105 (1.016-1.201) | 0.196 | 1.121 (1.042-1.205) | 0.032 |
| TNFRSF9 | 1.140 (1.060-1.226) | 0.008 | 1.120 (0.977-1.284) | 0.314 | 1.143 (1.049-1.246) | 0.046 | 1.008 (0.866-1.174) | 0.968 | 1.130 (1.039-1.229) | 0.080 | 1.110 (1.031-1.196) | 0.061 |
| TNFSF11 | 0.827 (0.766-0.892) | 1.11E-04 | 0.881 (0.752-1.031) | 0.334 | 0.829 (0.759-0.906) | 0.003 | 0.901 (0.773-1.050) | 0.486 | 0.848 (0.778-0.925) | 0.015 | 0.838 (0.776-0.904) | 0.001 |
| TNFSF13 | 1.191 (1.104-1.285) | 4.09E-04 | 1.225 (1.060-1.416) | 0.117 | 1.163 (1.064-1.272) | 0.027 | 1.149 (0.989-1.336) | 0.312 | 1.150 (1.053-1.255) | 0.049 | 1.164 (1.080-1.255) | 0.004 |
| TNFSF13B | 1.123 (1.041-1.212) | 0.028 | 1.107 (0.954-1.284) | 0.423 | 1.119 (1.024-1.223) | 0.130 | 1.066 (0.927-1.226) | 0.682 | 1.087 (0.994-1.188) | 0.370 | 1.109 (1.028-1.195) | 0.071 |
| TNFSF14 | 1.119 (1.038-1.206) | 0.033 | 1.100 (0.940-1.288) | 0.496 | 1.120 (1.028-1.220) | 0.109 | 1.150 (0.979-1.350) | 0.347 | 1.113 (1.023-1.212) | 0.150 | 1.126 (1.044-1.213) | 0.031 |
| TNR | 0.874 (0.808-0.945) | 0.012 | 0.868 (0.743-1.015) | 0.258 | 0.877 (0.801-0.960) | 0.067 | 0.826 (0.705-0.968) | 0.215 | 0.905 (0.828-0.989) | 0.235 | 0.876 (0.810-0.947) | 0.020 |
| TP53INP1 | 0.852 (0.776-0.934) | 0.011 | 0.778 (0.633-0.957) | 0.144 | 0.873 (0.787-0.967) | 0.112 | 0.830 (0.684-1.008) | 0.291 | 0.842 (0.757-0.937) | 0.044 | 0.854 (0.779-0.937) | 0.019 |
| TPP1 | 1.118 (1.043-1.197) | 0.019 | 1.140 (0.996-1.305) | 0.225 | 1.109 (1.023-1.202) | 0.126 | 1.032 (0.890-1.197) | 0.880 | 1.109 (1.024-1.200) | 0.133 | 1.113 (1.039-1.192) | 0.035 |
| TYMP | 1.164 (1.079-1.255) | 0.003 | 1.177 (1.005-1.378) | 0.196 | 1.154 (1.058-1.258) | 0.030 | 1.253 (1.080-1.453) | 0.157 | 1.114 (1.022-1.214) | 0.163 | 1.162 (1.077-1.254) | 0.005 |
| UBE2L6 | 1.127 (1.039-1.223) | 0.038 | 1.272 (1.071-1.512) | 0.117 | 1.091 (0.994-1.198) | 0.349 | 1.340 (1.131-1.588) | 0.136 | 1.053 (0.958-1.157) | 0.687 | 1.125 (1.037-1.221) | 0.054 |
| UMOD | 0.837 (0.779-0.900) | 1.45E-04 | 0.790 (0.690-0.905) | 0.065 | 0.864 (0.793-0.941) | 0.026 | 0.882 (0.759-1.025) | 0.372 | 0.838 (0.772-0.910) | 0.004 | 0.858 (0.798-0.923) | 0.003 |
| VCAM1 | 1.144 (1.065-1.228) | 0.005 | 1.129 (0.978-1.302) | 0.301 | 1.150 (1.059-1.249) | 0.027 | 1.141 (0.977-1.331) | 0.358 | 1.124 (1.036-1.220) | 0.085 | 1.124 (1.046-1.208) | 0.027 |
| VEGFA | 1.117 (1.038-1.202) | 0.032 | 1.315 (1.146-1.508) | 0.046 | 1.047 (0.960-1.142) | 0.697 | 1.058 (0.909-1.232) | 0.746 | 1.104 (1.015-1.201) | 0.199 | 1.109 (1.031-1.193) | 0.060 |
| VNN2 | 1.168 (1.079-1.265) | 0.003 | 1.288 (1.092-1.518) | 0.095 | 1.132 (1.034-1.239) | 0.093 | 1.147 (0.969-1.358) | 0.385 | 1.187 (1.085-1.299) | 0.014 | 1.165 (1.077-1.261) | 0.007 |
| VSIG2 | 1.217 (1.127-1.314) | 7.18E-05 | 1.189 (1.044-1.354) | 0.119 | 1.207 (1.097-1.329) | 0.008 | 1.034 (0.893-1.198) | 0.873 | 1.189 (1.086-1.302) | 0.014 | 1.181 (1.093-1.276) | 0.002 |
| VSIG4 | 1.198 (1.108-1.296) | 4.09E-04 | 1.164 (1.011-1.341) | 0.181 | 1.199 (1.091-1.317) | 0.009 | 1.193 (1.021-1.393) | 0.225 | 1.162 (1.061-1.273) | 0.037 | 1.147 (1.060-1.241) | 0.016 |
| VSIR | 1.108 (1.032-1.189) | 0.042 | 1.208 (1.054-1.384) | 0.117 | 1.073 (0.987-1.165) | 0.423 | 1.153 (1.008-1.319) | 0.254 | 1.068 (0.983-1.160) | 0.507 | 1.107 (1.032-1.188) | 0.055 |
| VWA1 | 1.121 (1.038-1.210) | 0.036 | 1.053 (0.897-1.235) | 0.753 | 1.145 (1.048-1.251) | 0.049 | 1.076 (0.925-1.251) | 0.662 | 1.096 (1.004-1.198) | 0.287 | 1.124 (1.041-1.214) | 0.038 |
| VWF | 1.115 (1.035-1.201) | 0.040 | 1.283 (1.104-1.491) | 0.070 | 1.065 (0.977-1.161) | 0.530 | 1.125 (0.972-1.304) | 0.388 | 1.079 (0.991-1.175) | 0.403 | 1.111 (1.031-1.197) | 0.061 |
| WFDC2 | 1.181 (1.092-1.277) | 0.001 | 1.179 (1.039-1.338) | 0.124 | 1.169 (1.060-1.289) | 0.039 | 1.128 (0.966-1.318) | 0.406 | 1.148 (1.047-1.258) | 0.068 | 1.133 (1.045-1.227) | 0.034 |
| WFIKKN1 | 0.863 (0.796-0.935) | 0.007 | 0.920 (0.781-1.084) | 0.585 | 0.849 (0.773-0.931) | 0.019 | 0.902 (0.758-1.074) | 0.566 | 0.888 (0.810-0.973) | 0.131 | 0.871 (0.804-0.944) | 0.019 |
| WNT9A | 1.122 (1.038-1.214) | 0.037 | 1.254 (1.099-1.431) | 0.066 | 1.065 (0.968-1.172) | 0.585 | 1.186 (0.986-1.426) | 0.312 | 1.103 (1.008-1.207) | 0.255 | 1.096 (1.013-1.187) | 0.145 |
| XCL1 | 1.130 (1.056-1.210) | 0.008 | 1.099 (0.960-1.259) | 0.411 | 1.139 (1.053-1.233) | 0.030 | 1.037 (0.890-1.209) | 0.865 | 1.148 (1.064-1.238) | 0.020 | 1.115 (1.041-1.195) | 0.031 |
| YAP1 | 1.166 (1.069-1.271) | 0.009 | 1.151 (0.988-1.341) | 0.249 | 1.161 (1.044-1.290) | 0.078 | 1.140 (0.959-1.354) | 0.421 | 1.129 (1.019-1.251) | 0.196 | 1.135 (1.040-1.237) | 0.052 |
| ZNRD2 | 1.124 (1.040-1.215) | 0.032 | 1.259 (1.082-1.465) | 0.100 | 1.077 (0.983-1.180) | 0.454 | 1.175 (1.010-1.366) | 0.250 | 1.092 (0.998-1.194) | 0.337 | 1.125 (1.041-1.215) | 0.039 |

**Supplementary Fig. S1 | Flowchart of participant selection for the four psychiatric outcome analyses.**

**Note:** The four analytic cohorts were not mutually exclusive. For each cohort, the comparison group consisted of participants who did not develop any of the four psychiatric disorders during follow-up. MDD, major depressive disorder.

**Supplementary Fig. S2 | Distributions of WNPRS-Anxiety and WNPRS-MDD.** Density histograms showing the distributions of WNPRS-Anxiety and WNPRS-MDD across participants. Both scores were constructed from the same 44 shared mediator proteins using the corresponding mediation effect estimates as weights. The overlaid curves show the smoothed density distributions.
